# Global analysis of *SBP* gene family in *Brachypodiumdistachyon* reveals its association with spike development

**DOI:** 10.1038/s41598-020-72005-7

**Published:** 2020-09-14

**Authors:** Rajiv K. Tripathi, William Overbeek, Jaswinder Singh

**Affiliations:** Plant Science Department, McGill University, 21111 Rue Lakeshore, Quebec, H9X 3V9 Canada

**Keywords:** Non-coding RNAs, Biological techniques, Computational biology and bioinformatics, Developmental biology, Genetics, Molecular biology, Plant sciences

## Abstract

*SQUAMOSA*-promoter binding like proteins (SBPs/SPLs) are plant specific transcription factors targeted by miR156 and involved in various biological pathways, playing multi-faceted developmental roles. This gene family is not well characterized in *Brachypodium*. We identified a total of 18 *SBP* genes in *B.* *distachyon* genome. Phylogenetic analysis revealed that *SBP* gene family in *Brachypodium* expanded through large scale duplication. A total of 10 *BdSBP* genes were identified as targets of miR156. Transcript cleavage analysis of selected *BdSBPs* by miR156 confirmed their antagonistic connection. Alternative splicing was observed playing an important role in *BdSBPs* and miR156 interaction. Characterization of T-DNA *Bdsbp9* mutant showed reduced plant growth and spike length, reflecting its involvement in the spike development. Expression of a majority of *BdSBPs* elevated during spikelet initiation. Specifically, *BdSBP1* and *BdSBP3* differentially expressed in response to vernalization. Differential transcript abundance of *BdSBP1,* *BdSBP3,* *BdSBP8,* *BdSBP9,* *BdSBP14,* *BdSBP18* and *BdSBP23* genes was observed during the spike development under high temperature. Co-expression network, protein–protein interaction and biological pathway analysis indicate that *BdSBP* genes mainly regulate transcription, hormone, RNA and transport pathways. Our work reveals the multi-layered control of *SBP* genes and demonstrates their association with spike development and temperature sensitivity in *Brachypodium*.

## Introduction

Characterization of various transcription factors has revealed their organism-specific function and a particular class of these transcription factors have been discovered in animals, yeast and plants. *SQUAMOSA*-promoter binding like proteins (SBPs) form a major family of plant-specific transcription factors. SBPs were first identified in *Antirrhinum* *majus* interacting with the promoter sequence of floral meristem gene *SQUAMOSA*^1^*.* SBP-box proteins share a highly conserved 76 amino-acids long DNA binding domain known as SBP domain, which contains two zinc ion binding motifs (Cys2HisCys and Cys3His) and a nuclear localization signal (NLS) sequence^2^. The SBP domain of the SBP-box family members binds to TNCGTACAA consensus sequence present in the promoter regions with GTAC as a core motif^3,4^. Phosphorylation of serine amino acid within the SBP domain has been recently shown to modify DNA binding affinity and immunity in rice^5^. As a multigene family, *SBP* genes have been identified in green moss^6^, algae^7^, *P. trichocarpa*^8^ and angiosperm^9^. There are 16 *SBP* genes in *Arabidopsis* *thaliana*^10^, 19 in rice^11^, 28 in *Populus* *trichocarpa*^8^, 41 in soybean^12^ and 17 in barley^13^. *SBP* genes play key roles in various plant developmental pathways such as flowering time^14^, vegetative to reproductive phase transition^15,16^, plant architecture^17–19^, gibberellic acid biosynthesis^20,21^, anthocyanin biosynthesis^22^, and abiotic stresses^23,24^.

Another layer of gene regulation involves microRNAs (miRNAs), which are single-stranded non-coding RNA molecules of 20–22 nucleotides in length that bind to their complementary sequences present in the messenger RNAs (mRNAs) of their target genes^25,26^. Thus, both miRNAs and their target genes can be manipulated for crop improvement. Out of 16 *SBP* genes in *Arabidopsis,* 10 are known to be negatively regulated by a conserved miRNA, miR156^27^. The role of SBP/miR156 module has been observed in many plant developmental processes such as, vegetative to reproductive phase change, plant architecture and flowering time^14,18^. On the basis of SBP domain, these genes can be classified into five groups in *Arabidopsis* such as *SPL3/4/5*, *SPL9/15*, *SPL2/10/11*, *SPL6* and *SPL13A/B*^6,11^. The miR156 targeted SPLs accelerate the phase transition by positively regulating the expression of *APETALA1* (*AP1*), *FRUITFULL* (*FUL*), and *SUPPRESSOR* *OF* *CONSTANS* *OVEREXPRESSION* *1* (*SOC1*) and *LEAFY* genes^28–30^. In wheat and barley, *VERNALIZATION1* (*VRN1*) is the homolog of *FUL*/*AP1* and acts as both an activator and a target of *VRN3* which is a homolog of *FLOWERING* *LOCUS* *T* (*FT*)^31–33^. Based on the gain or loss of functions, *SPL* genes in *Arabidopsis* can be classified into three groups^30^. Group 1 contains *SPL2*/9/10/11/13/15 genes which promote both the juvenile-to-adult transition (vegetative phase) and the vegetative-to-reproductive phase transition. The *SPL9*/*13/15* genes are central players for these developments as compared to *SPL2/10/11*. Group 2 contains *SPL3*/*4*/*5* genes, which play major roles to accelerate the transition of floral meristem identity. Group 3 contains only *SPL6* which does not have a major role in shoot development but may be key to some other biological pathways.

In monocots, SBP/miR156 module has been anticipated as an important tool-box to genetically enhance crop productivity^34^. Interaction between miR156-SPLs and strigolactone signaling pathway regulates bread wheat tiller branching and spikelet development^35^. In rice, overexpression of *OsmiR156* targets *OsSPL14* and promotes panicle branching and grain yield^18,36^. Likewise, miR156 regulated *OsSPL16* and *OsSPL13* control grain shape, size and quality in rice^37,38^. The *unbranched2* and *unbranched3* members of SBP-box transcription factor family modulate plant architecture and yield in maize^39^. In switchgrass, miR156/SPL4 module regulates aerial axillary bud formation; branching and biomass yield^19^.

*Brachypodium* *distachyon,* the small monocot plant, is an emerging model system ideal for functional genomics research to study complex monocot species, especially the triticeae crops^40,41^. It is extensively being used to study the biology of flowering and vernalization response^42,43^. In *Arabidopsis*, *FLOWERING* *LOCUS* *T* (*FT*) gene is known as a key player of floral signaling processes^44^. In *Brachypodium*, *FT* undergoes age dependent alternative splicing and is regulated by miR5200 and FT/miR5200 module control photoperiod dependent flowering^45–48^. Homoeolog-specific transcriptome changes under heat stress conditions have been examined in *B.* *stacei*, *B.* *distachyon* and *B.* *hybridum*^49^. *Brachypodium* *auxin* *influx* *facilitato*r (*AUX1*) T-DNA mutants showed dwarf phenotype and had aberrant flower development^50^. In the current study, we identified 18 *SBP* genes in *B.* *distachyon* genome and studied their phylogenetic relationship with barley, wheat, rice and *Arabidopsis*. Further, their gene structure, alternative splicing event, gene duplication event, miR156 mediated negative regulation, co-expression and protein–protein interaction network have been investigated systematically. Transcriptional changes of individual *SBP* genes in leaf and spike at different developmental stages and temperature regimes were critically examined. Moreover, *SBP* genes role in early (Bd21) and late (Bd1-1) flowering accessions of *Brachypodium* was verified. Further, *Bdsbp9* T-DNA mutant was characterized to understand its function in spike development.

## Results

### Identification and characterization of *SBP-box* genes in *B. distachyon*

In this study, we identified 18 *SBP* genes in *B.* *distachyon* and designated as *BdSBP.* *BdSBP* family members were named according to the closest homologs present in wheat, barley or rice. Details of *SBP* gene family in *Brachypodium* are given in Table 1. *Brachypodium* *SBP* genes encode proteins ranging from 177 (SPL7) to 1,110 (SPLl4) amino acids (aa) in length and from 122 kda (SBP15) to 12 kda (SBP23A) in molecular weight. The number of exons ranged from 1 to 11 and isoelectric point (pI) was from 5 to 10. The 18 *BdSBP* genes were located on all 5 chromosomes (chr), with maximum number of *BdSBP* genes detected in chr 3 of *B.* *distachyon* (Table 1).

**Table 1:** Characteristics of *SBP* Genes in *Brachypodium* *distachyon.* Asterisks * denotes miR156 Targeted *BdSBPs.*

Gene name^a^	Gene symbol^b^	CDS^c^ length (bP)	Domain^d^	Deduced protein^e^			Chr^f^	Position on genome^g^	Exon^h^ No
				Length (aa)	MW (kDa)	pI			
BdSBP18*	BRADI3G41250	1,278	SBP	425	44.35	7.11	3	3:43,201,676–43,206,673:1	3
BdSBP14*	BRADI3G40030	1,176	SBP	391	40.50	9.16	3	3:42,259,872–42,263,157:− 1	3
*BdSBP8*	BRADI5G24670	1,263	SBP	420	45.81	7.51	5	5:26,221,739–26,225,184:− 1	3
BdSBP3*	BRADI3G03510	1,458	SBP	485	52.24	8.34	3	3:2,279,990–2,284,027:− 1	4
*BdSBP21*	BRADI3G05720	1,452	SBP	484	51.95	6.62	3	3:4,069,051–4,071,726:1	3
*BdSBP7*	BRADI5G17720	567	SBP	188	20.57	10.31	5	5:20,917,607–20,918,254:1	2
*BdSBP22*	BRADI1G31390	1,272	SBP	423	46.46	10.47	1	1:26,879,476–26,882,414:1	3
BdSBP11*	BRADI3G05510	990	SBP	329	35.41	9.31	3	3:3,895,778–3,899,371:1	4
BdSBP23*	BRADI2G59110	1,179	SBP	392	41.66	9.10	2	2:56,854,357–56,858,238:1	3
BdSBP16*	BRADI4G34667	1,266	SBP	421	42.02	8.55	4	4:40,207,721–40,212,788:1	3
BdSBP13*	BRADI1G26720	579	SBP	192	20.04	9.92	1	1:21,747,747–21,750,619:1	2
BdSBP17*	BRADI4G33770	1,224	SBP	407	42.51	7.8	4	4:39,473,148–39,476,188:− 1	3
*BdSBP6*	BRADI1G02760	2,889	SBP, ANK	962	105.09	5.44	1	1:1,858,041–1,863,792:1	11
*BdSBP15*	BRADI3G40240	3,381	SBP, ANK	1,126	122.96	6.92	3	3:42,427,821–42,432,953:1	10
BdSBP1*	BRADI2G11240	2,664	SBP, ANK	887	84.73	9.18	2	2:9,490,127–9,495,150:1	11
*BdSBP9*	BRADI2G25580	2,550	SBP, DEXDC	849	92.84	5.69	2	2:23,666,234–23,676,756	10
*BdSBP23A*	BRADI4G18890	360	SBP	119	12.54	9.12	4	4:21,459,257–21,462,340	1
BdSBP13A*	BRADI4G18900	651	SBP	216	23.24	7.74	4	4:21,464,988:21,467,761	2

### Phylogenetic analysis and gene duplication in *BdSBP* genes

**Figure 1:**
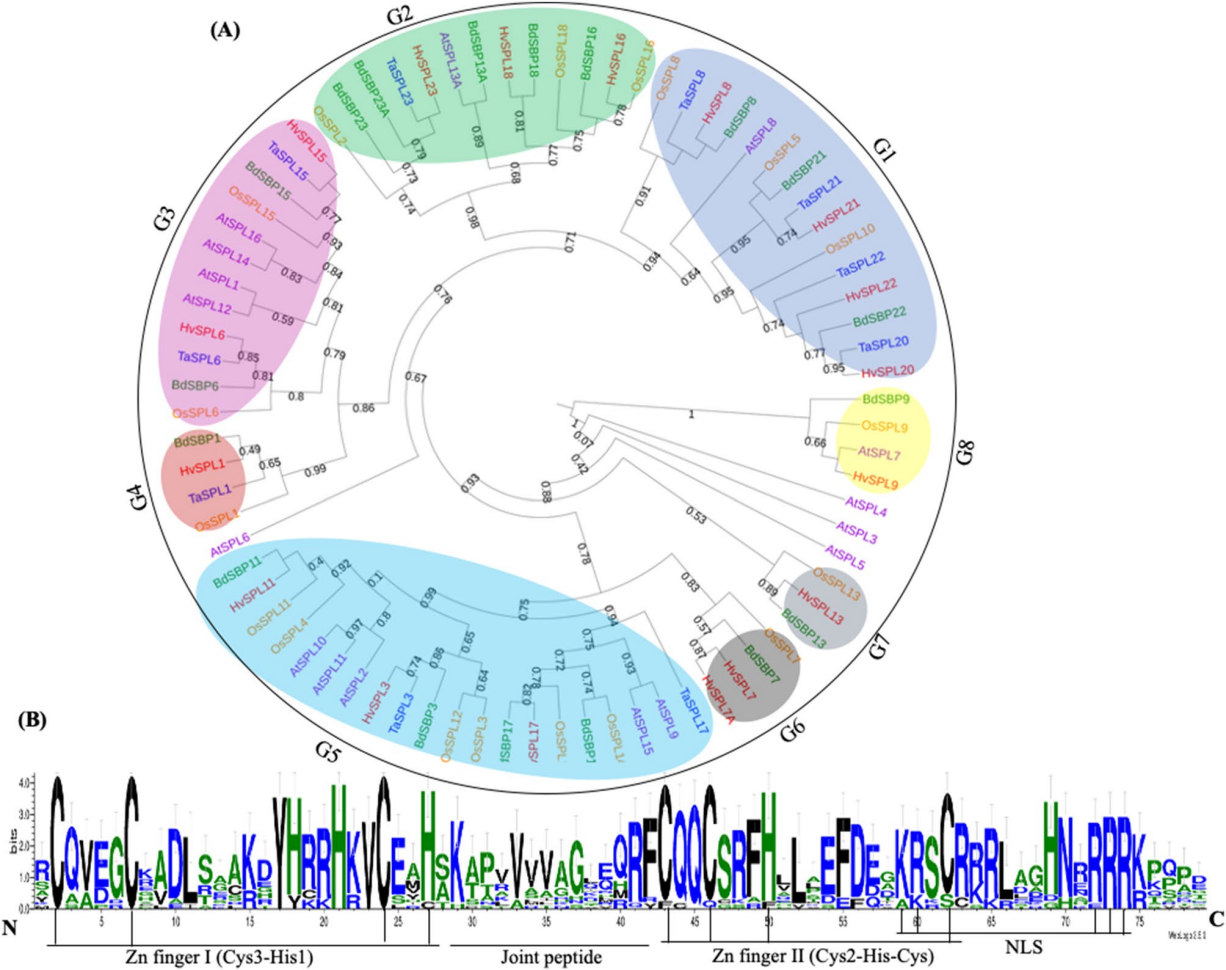
Evolutionary analysis of SBP domain transcription factors. (**A**) Phylogenetic tree of SBP proteins taken from *Brachypodium*, barley, wheat, rice and *Arabidopsis*. The amino acid sequences were aligned using MUSCLE tool and Interative Tree of Life (iTOL) resource was used to annotate the phylogenetic tree. (**B**) Sequence logo of *Brachypodium* SBP domain. The height of amino acid residues shows level of conservation. Two zinc finger motif and nuclear localization signal (NLS) and joint peptide are shown.

**Figure 2:**
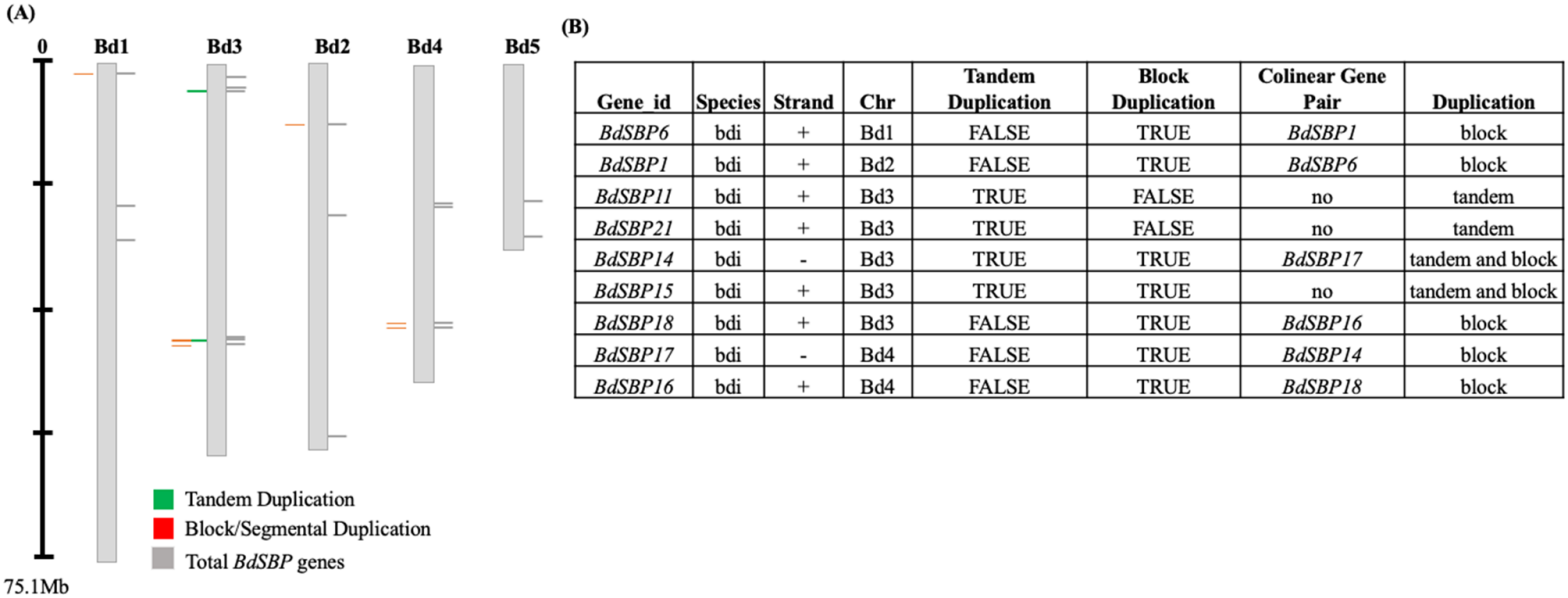
Gene duplication of *SBP* genes in *Brachypodium* genome. (**A**) Segmental and tandem duplications gene pairs located on *Brachypodium* chromosome regions are marked in red and green colours. The gray lines on each chromosome represent the total number of *SBP* genes present in *Brachypodium* genome. (**B**) Summary of *BdSBP* duplicated gene pairs and type of duplication events in *Brachypodium*.

A phylogenetic tree was constructed using conserved SBP domain sequences of SBP proteins from *Brachypodium*, wheat, barley, rice and *Arabidopsis* (Fig. 1A,B). A total of 79 SBP proteins from different plant species including 18 from rice, 10 from wheat, 17 from barley and 16 from *Arabidopsis* were used for phylogenetic analysis. SBPs clustered into 8 groups (G1–G8), with AtSBP3/4/5/6 as ungrouped members. Each group contained at least one SBP protein from *Brachypodium.* As anticipated, BdSBPs exhibited closer relationship with the SBP proteins from barley and wheat as compared to rice and *Arabidopsis*. Group 1 and group 5 contained maximum number of BdSBPs, where SBP proteins from barley and wheat were also grouped. Moreover, gene duplication analysis among *BdSBP* genes identified 9 putative paralogous gene pairs in the *Brachypodium* genome (Fig. 2A,B). Divergence time for duplicated *BdSBP* genes was estimated from Ka and Ks values and their ratios. The dates of duplication events (T) were calculated using Ks values through the formula T = Ks/2λ × 10^−6^ (millions of year, Mya). The λ = 6.5 × 10^−9^ substitutions per synonymous site per year was assumed as universal clock-like rate for *Brachypodium* *distachyon*. For *BdSBP1* and *BdSBP6* gene pair, Ka and Ks values were 0.60 and 2.14, respectively and their ratios 0.28 imply their evolution under purifying selection. Similarly, the ratio (0.30) of Ka and Ks values for *BdSBP16* and *BdSBP18* gene pair highlights purifying selection. Purifying selection also called negative selection, influence genomic diversity in natural populations. It eliminates the changes that produce deleterious effects on the fitness of the host. The frequency distributions indicate that *SBP* genes in *Brachypodium* went through a large-scale duplication event ranging from 55 to 164 million years ago (mya). *SBP* gene paralogs were located on same as well as different chromosomes, indicating that expansion of *Brachypodium* *SBP* genes was both, tandem as well as segmental/block duplication during evolution.

### *BdSBP* genes have diverse gene and protein structures

**Figure 3:**
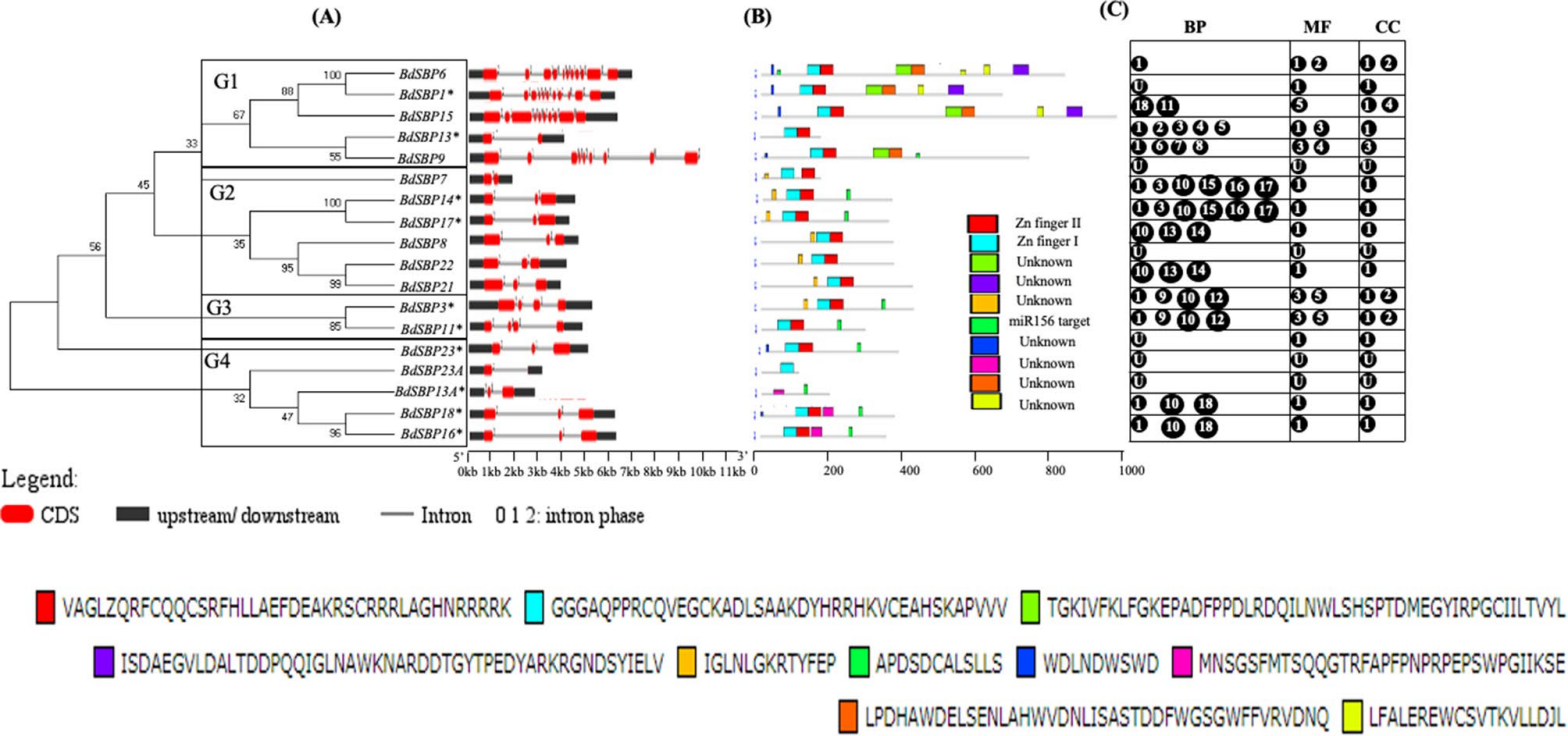
Gene structure and protein motif analysis of BdSBP genes. (**A**) Un-rooted neighbour-joining tree was developed using SBP domain sequences through MEGA6 package. The organizations of exon–intron and intron phases of the BdSBP genes are displayed. Exons, introns and 5′UTR/3′UTR are denoted by red boxes, horizontal black lines and black boxes, respectively. *miR156 targeted BdSBP genes. (**B**) The conserved motifs in BdSBP proteins are shown in different colors. Full length protein sequences of BdSBPs were used to search motif using MEME tool. (**C**) Functions of BdSBP genes are annotated based on Gene ontology. The biological processes (BP), molecular functions (MF), and cellular component (CC) are shown in the box below. Biological Processes (BP): 1. GO:0006355; regulation of transcription, DNA-templated; 2. GO:0010229;inflorescence development; 3. GO:0010228;vegetative to reproductive phase transition of meristem; 4. GO:0010321;regulation of vegetative phase change; 5. GO:0009911;positive regulation of flower development; 6. GO:0055070;copper ion homeostasis; 7. GO:0048638;regulation of developmental growth; 8. GO:0035874;cellular response to copper ion starvation; 9. GO:0048510;regulation of timing of transition from vegetative to reproductive phase; 10. GO:0048653;anther development; 11. GO:0045893;positive regulation of transcription, DNA-templated 12. GO:0010358;leaf shaping 13. GO:0009556;microsporogenesis; 14. GO:0009554;megasporogenesis; 15. GO:0042127;regulation of cell proliferation; 16. GO:2000025;regulation of leaf formation; 17. GO:0008361;regulation of cell size;18. GO:0042742;defense response to bacterium; 19. Unknown (U). Molecular function (MF): 1.GO:0003677; DNA binding; 2. GO:0005515;protein binding; 3. GO:0003700; transcription factor activity, sequence-specific DNA binding; 3. GO:0043565; sequence-specific DNA binding; 4. GO:0042803;protein homodimerization activity; 5. GO:0044212; transcription regulatory region DNA binding; 6. unknown (U). Cellular Component (CC): 1. GO:0005634;Nucleus; 2. GO:0009941;chloroplast envelope 3. GO:0016607;nuclear speck; 4. GO:0005886;plasma membrane 5. Unknown (U).

Gene structure and genetic diversity analyses (Fig. 3A–C) in *Brachypodium* *SBP* gene family revealed that the *BdSBP* genes contain at least one intron; however genes in group 1–3 have the largest number (10–11) of exons (Fig. 3A). Other *BdSBP* genes possess only 2–4 exons. Interestingly, five sister gene pairs (SBP1/6; SBP14/17; SBP21/22; SBP3/11 and SBP16/18) have similar exon/intron numbers but intron phases with variable lengths. Conserved motif sequence database search identified a total of 10 motifs, which were designated as motif 1–10 (Fig. 3B). Gene pairs (*SBP1/6/15*; *SBP14/17*; *SBP8/21/22* and *SBP16/18*) shared a similar type of motif structure. Some motifs were found to be specific to one or two groups of BdSBP proteins. Motif 6 that encodes miR156 target sequence was present in all miR156 targeted BdSBP proteins. Whereas motif 10 and motif 4 were found in group 1 BdSBP and group 3 and 4 BdSBP proteins respectively. To predict possible functions of *BdSBP* genes, we also performed gene ontology (GO) term enrichment analysis (Fig. 3C). Most of the *BdSBP* genes with similar gene structure and motifs (*BdSBP1/6*; *BdSBP3/11*; *BdSBP13A/23A*; *BdSBP14/17*; and *BdSBP16/18*) were predicted for their similar biological processes (BP), molecular function (MF) and cellular component (CC).

### Higher transcript abundance of *BdSBP* genes correlates with early inflorescence development

**Figure 4:**
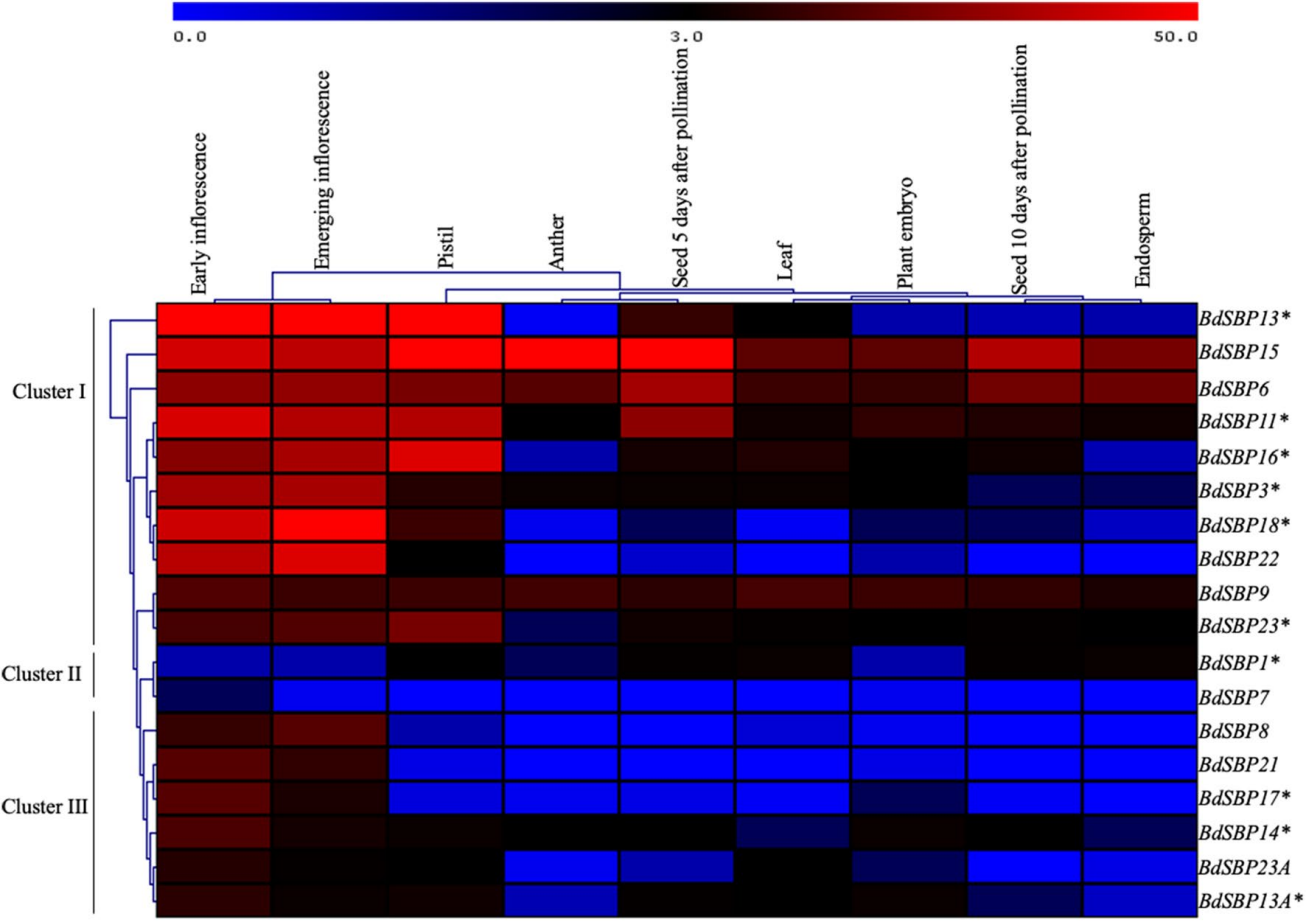
Expression pattern of *BdSBP* genes in nine different tissues. The log2 transformed FPKM values are represented as a color scale bar on the top of heatmap which shows high and low expression. Details of tissues utilized for expression analysis are shown on the top of map. The genes are mentioned on the right side of the map and *denotes miR156 targeted *BdSBPs*.

RNA-seq data of *B.* *distachyon* acc. Bd21(https://www.ebi.ac.uk/gxa/experiments/E-MTAB-4401/Results) from 9 different tissues and organs (leaf, early inflorescence, emerging inflorescence, anther, pistil, seed 5 days after pollination, seed 10 days after pollination, plant embryo and endosperm) was mined to understand the dynamics of *BdSBP* genes expression (Fig. 4). The expression profile of *BdSBPs* was grouped into three clusters. Higher expression of *BdSBP* genes of cluster 1 was observed in early inflorescence, emerging inflorescence and pistil tissues. Many *BdSBP* genes of cluster 1 were also expressed in anther, plant embryo, developing seeds (5 and 10 days after pollination), leaf, and endosperm tissues, implying their significant role throughout the *Brachypodium* plant development, especially in spike architecture. The *BdSBP* genes of cluster 2 (*BdSBP7* and *BdSBP1*) either lacked expression in any tissue (*BdSBP7*) or poorly expressed (*BdSBP1*) in pistil, leaf, and developing seeds (seed 5 and 10 days after pollination), and endosperm. The *BdSBP* genes from cluster 3 were found to be expressed mainly in early and emerging inflorescence. Expression profiles of *BdSBP* genes indicate their involvement in the reproductive units of *B.* *distachyon.*

### Post-transcription of *BdSBP* genes is regulated by miR156 and alternative splicing (AS)

**Figure 5:**
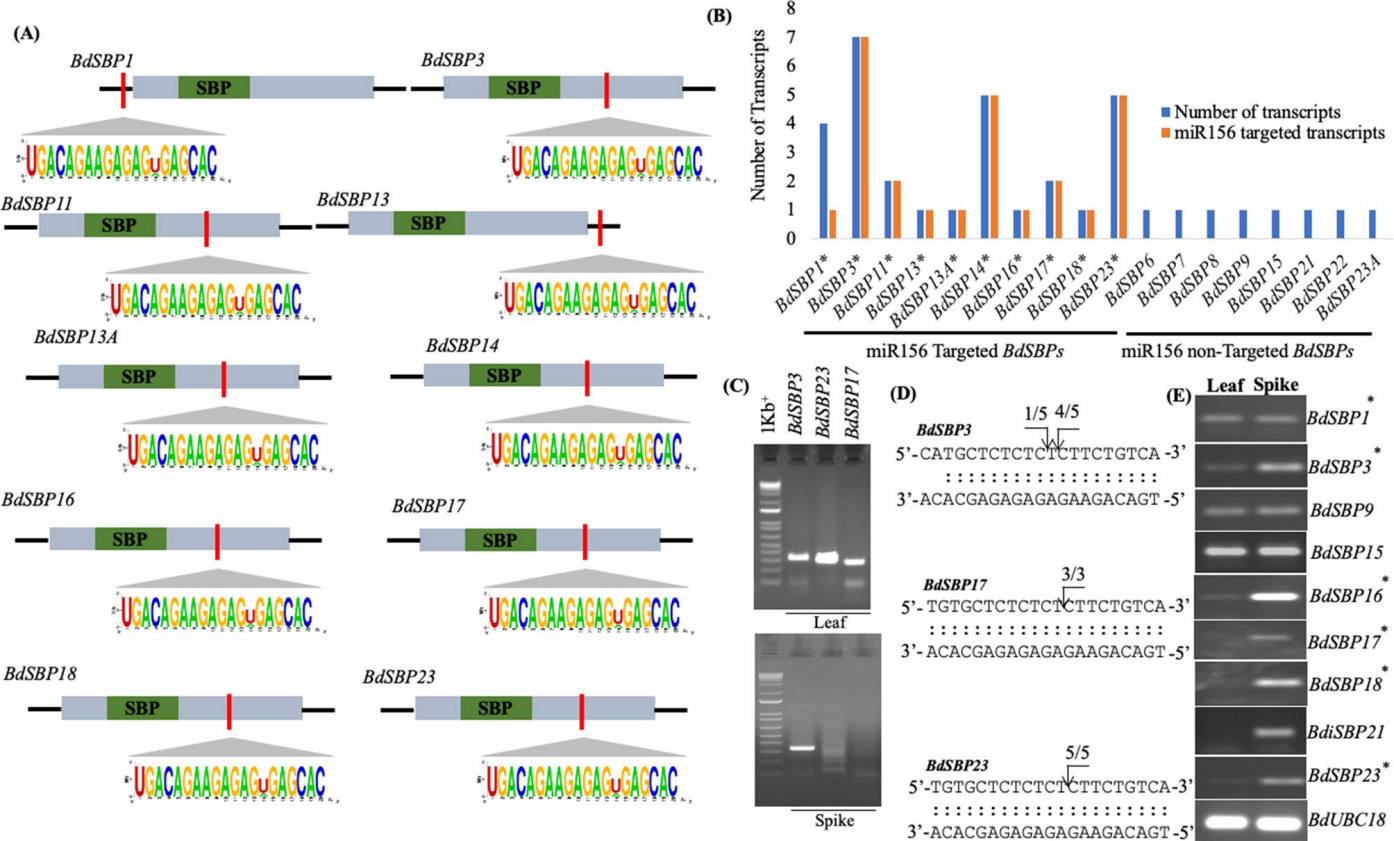
Analysis of post-transcriptional regulation of *BdSBP* genes by miR156 and AS. (**A**) Prediction of miR156 target site in *BdSBPs* transcripts. The SBP domain is represented in green. miR156 complementary sequences of *BdSBPs* are indicated in red. 5′ and 3′-UTRs are indicated in black horizontal lines. (**B**) Details of AS events in miR156 targeted and non-targeted *BdSBP* genes. X-axis shows *BdSBP* genes and Y-axis indicates number of transcripts. (**C**) Agarose gel image showing product of 5′-RACE PCR to measure transcript cleavage of *BdSBP* genes by miR156. (**D**) miR156 mediated cleavage site mapping in *BdSBP3*, *BdSBP17* and *BdSBP23* genes. The arrow indicates exact cleavage site and number indicates clones used for confirmation. (**E**) Semi-quantitative RT-PCR analysis of *BdSBP* genes in leaf and spike tissues. *BdUBC18* was used as an internal control. miR156 targeted *BdSBP* genes expressed poorly in leaf and higher in spike.

The cDNA sequences of *BdSBP* genes were searched for putative target sites of *Brachypodium* miRNAs (Fig. 5A). Ten *BdSBP* genes are found to be the target of miR156. Out of these, 8 contain miR156 complementary sequences in their coding regions. However, in other two genes, *BdSBP1* and *BdSBP13*, miR156 target site was found in their 3′-UTR and 5′-UTR regions, respectively. The *BdSBP* gene family undergoes AS which specifically targets miR156 regulated *BdSBP* (Fig. 5B). The number of splice isoforms for each *BdSBP* genes was derived from plant Ensembl database. Splice variants from Ensembl gene are compared to generate an inclusive list of elementary alternative splicing events. The range of splice isoforms produced by *BdSBPs* was between 2 and 7. Most of the splice variants of *BdSBP* genes possess miR156 target site except *BdSBP1,* which has 4 splice variants and only one contains miR156 target site.

### Organ specific differential accumulation of *BdSBP* genes is regulated by miR156

Three *BdSBP* genes (*BdSBP3*, *BdSBP17* and *BdSBP23*) were analyzed for miR156 mediated transcript degradation by 5′-RLM-RACE (Fig. 5C,D). Additionally, to observe miR156 mediated cleavage pattern, we also constructed cDNA libraries from leaf and spike. Interestingly, *BdSBP* genes were highly degraded by miR156 in leaf as compared to spike tissue. We hypothesized that this differential degradation of *BdSBP* genes in leaf and spike tissues might be connected with their expression patterns in these tissues. To validate this, we performed semi-quantitative RT-PCR of several potential *BdSBP* genes on the basis of in silico expression data (Fig. 5E). Our data indicate that miR156 targeted *BdSBP* genes indeed expressed poorly in the leaf and abundantly in the spike, confirming our hypothesis. The miR156 non-targeted genes (*BdSBP9* and *BdSBP15*) expression was constant in both the leaf and the spike, suggesting no effect of miR156 on these genes. Furthermore, to map the miR156 cleavage site in *BdSBPs* transcript, the 5′-RLM-RACE products were cloned and sequenced. Data indicates that miR156 cleaves between 9 and 10th nucleotide of 5′ site of *BdSBPs* transcript, except *BdSBP3* where cleavage site was found between 10 and 11th nucleotides. Collectively, our results suggest multilayered regulation of *BdSBP* genes at the post-transcriptional level.

### *BdSBP* genes are involved in complex regulatory network and pathways

**Figure 6:**
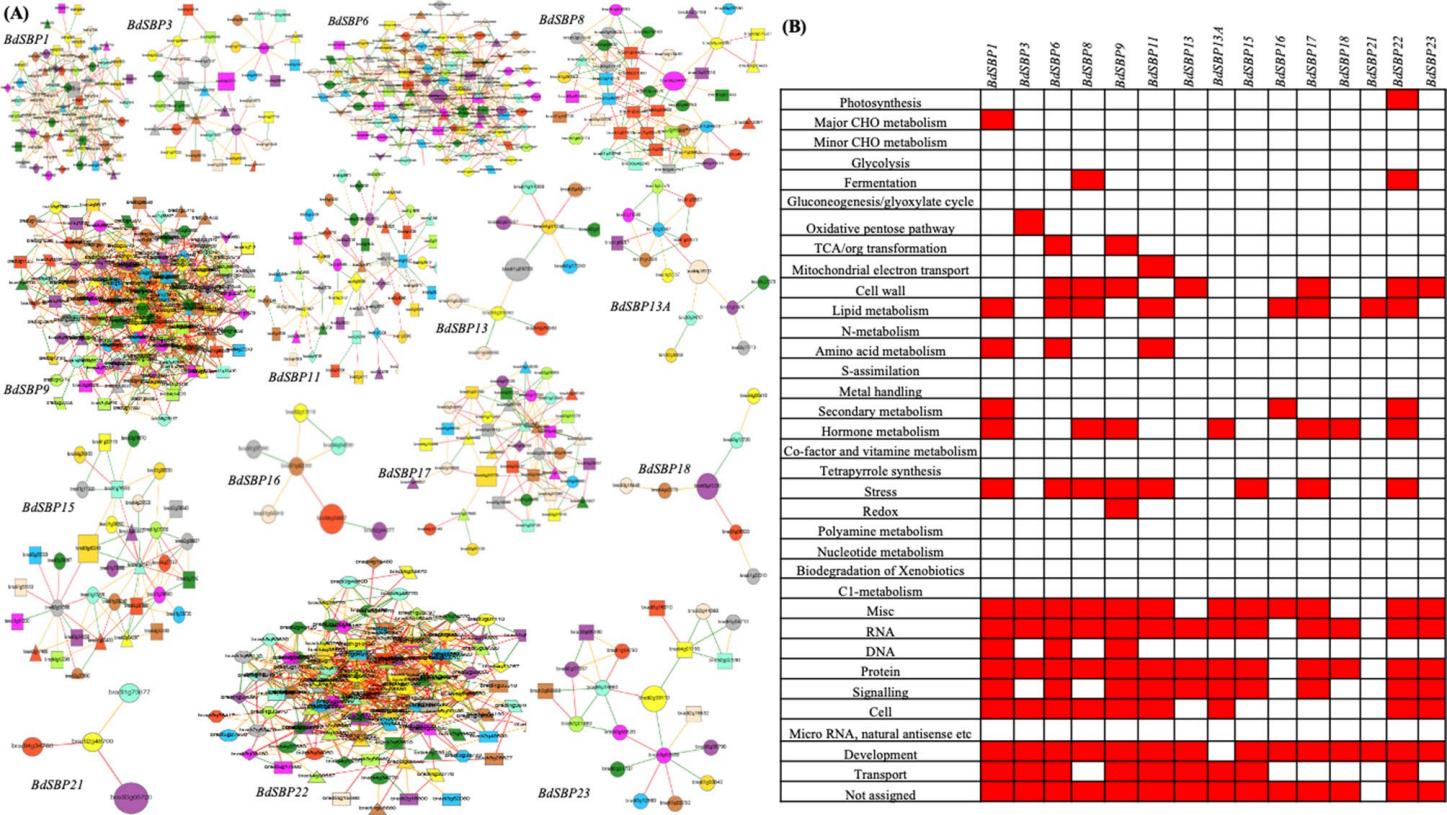
Co-expression and metabolic pathway analysis of *BdSBP* genes: (**A**) the co-expression neighbourhood was analysed using PlaNet tool. The green, oranges and red edge colours shows strong, medium and weak co-expression. Coloured shapes indicate label co-occurrences. The gene annotation of co-expressed genes is available in Supplementary Table S5. (**B**) The co-expressed genes of *BdSBP*s that are enriched for a biological pathway given by MapMan term are shown by red boxes.

*BdSBPs* co-expressed genes were investigated using publicly available large-scale co-expression database (www.gene2function.de), and MapMan (https://mapman.gabipd.org) ontology term enrichment to study their roles in different biological pathways (Fig. 6A–B). Around 710 co-expressed genes were found to be associated with 15 members of *BdSBP* family (Supplementary Table S5). The MapMan ontology of the co-expressed genes suggests that 22 out of 35 of the major biological classes have at least one of the *BdSBP* family members (Fig. 6B). Cell, development, transport, hormone metabolism, secondary metabolism, stress, lipid metabolism, cell wall, DNA, RNA, protein and signaling were major biological processes in which co-expressed genes of *BdSBP* family members were involved. Some other *BdSBP* family members and their co-expressed genes were enriched in photosynthesis, major CHO metabolism, fermentation, oxidative pentose pathway, mitochondrial electron transport and amino acid metabolism. However, *BdSBP* co-expressed genes were not augmented in the C1-metabolism, microRNA, polyamine metabolism, nucleotide metabolism, S-assimilation, N-metabolism, glycolysis and minor CHO metabolic pathways.

**Figure 7:**
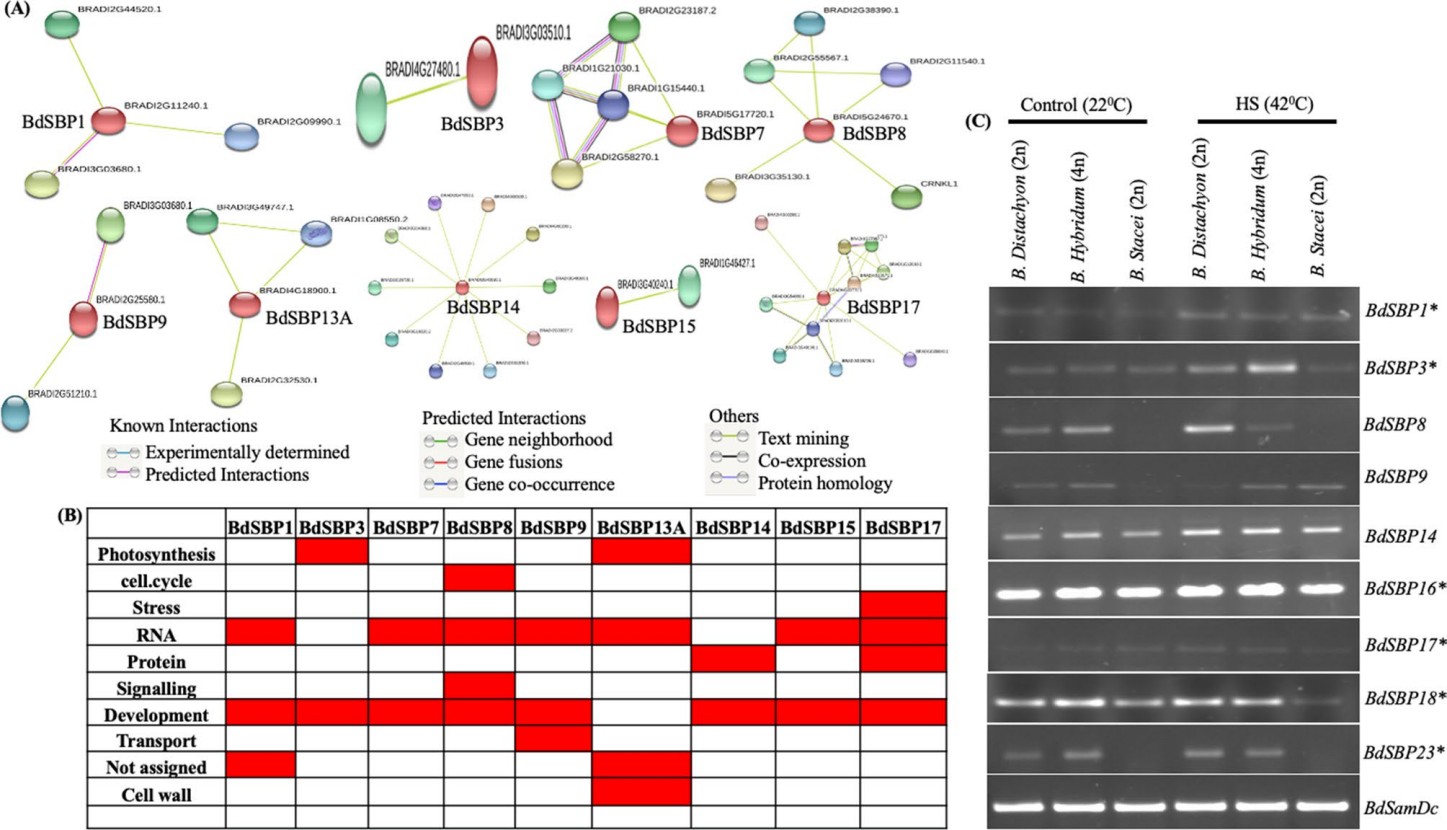
Protein interaction network and pathway analysis of BdSBP proteins. (**A**) The potential interactors for 9 BdSBP proteins were predicted using STRING tool and are shown with different coloured connective lines. (**B**) The biological pathways enriched in MapMan terms in which the interacting partners of BdSBP proteins are involved. (**C**) Effect of heat stress on the expression of *BdSBP* genes in *B.* *distachyon*, *B.* *stacei*, and *B.* *hybridum* grown under normal (22 °C) and heat stress (42 °C) conditions.

In addition, a network of protein–protein interaction of *B.* *distachyon* proteins was developed using STRING database. This database predicts interactions based on experimentally determined, predicted, text mining, co-expression, gene fusion, gene neighbourhood etc. A total of 39 interactive proteins were found (confidence value = 0.5) for 9 of BdSBP proteins, which were based on either predicted interactions or text mining (Fig. 7A, Supplementary Table S6). Protein annotation reveals that BdSBP proteins might interact with MYB33, PHABULOSA (PHB), Homeobox TF family, Growth regulating factor 5 (GRF5), Heat shock TF, ZnF C2H2, F-box TF, NBS-LRR, protein kinase family protein, ankyrin repeat protein, protein kinase family protein, chlorophyll a-b binding protein, DCL1, DCL2 and DCL3 proteins. The BdSBP7 was the only *B.* *distachyon* protein that interacts with DCL2 and DCL3 proteins. The MapMan term ontology of interactive protein partners of the BdSBPs indicates that 10 out of 35 proteins of the major biological terms were enriched by at least one of the BdSBP protein network (Fig. 7B). These biological processes were linked to development, RNA, photosynthesis, cell wall, protein, transport, signaling, cell cycle and stress.

### *BdSBP* genes express differentially during variable temperature conditions

In order to advance our knowledge about the molecular mechanism controlling heat stress in *Brachypodium*, we examined the transcriptional changes in *BdSBP* genes in the spike development under 22 °C and 42 °C in *B.* *distachyon*, *B.* *stacei* and *B.* *hybridum* (Fig. 7C). Transcript abundance of *BdSBP1,* *BdSBP14* and *BdSBP16* was higher at 42 °C among all the accessions, independent of ploidy level and it will be important to functionally validate these key genes in future. Negligible transcript abundance of *BdSBP8,* *BdSBP9* and *BdSBP23* was observed in *B.* *stacei*. However, transcript level of *BdSBP3,* *BdSBP8,* *BdSBP9,* *BdSBP18* and *BdSBP23* was higher in *B.* *distachyon* and *B.* *hybridum* as compared to *B.* *stacei* under both conditions*.* We did not observe any temperature dependent specific expression pattern among miR156 targeted and non-targeted *BdSBP* genes. Our results imply important roles of *BdSBP* genes to beat the heat in the reproductive organs of *Brachypodium* spp.

### *BdSBP* genes regulate spike development and flowering

**Figure 8:**
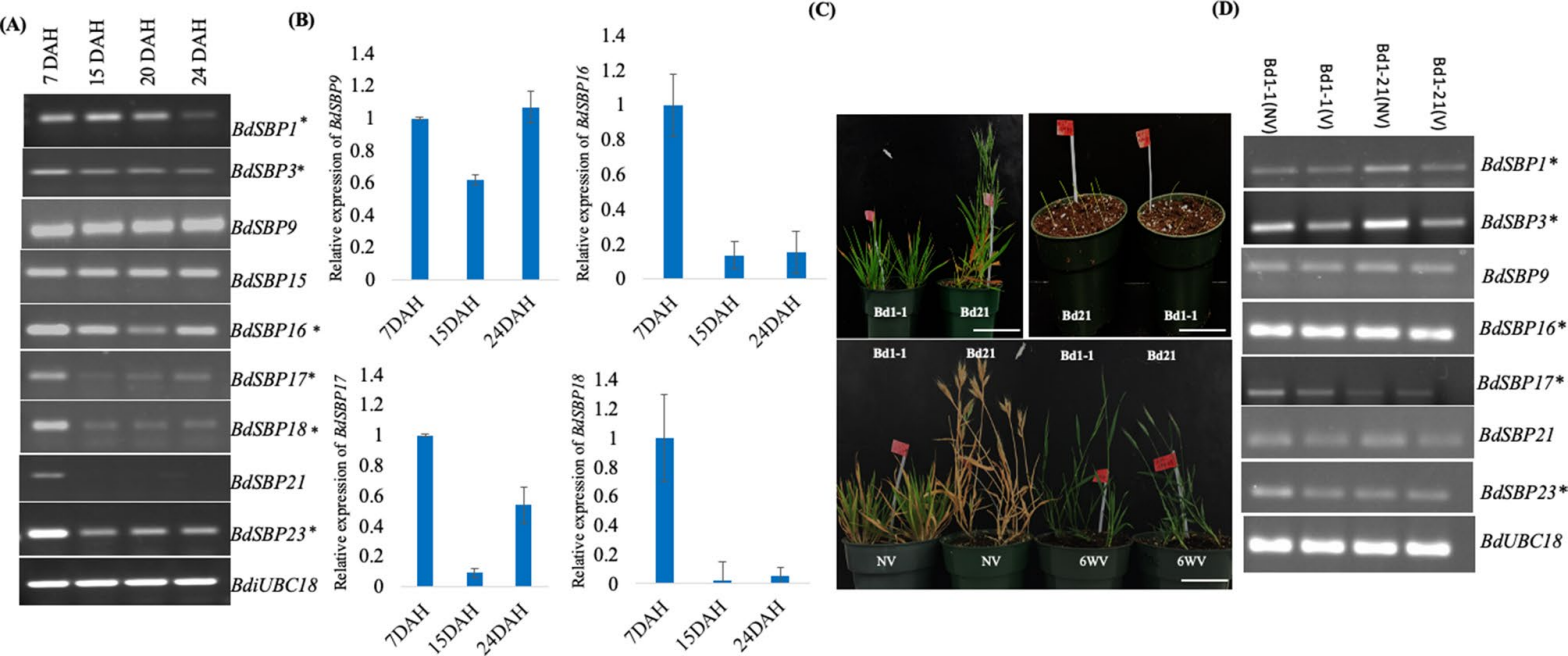
Expression pattern of *BdSBP* genes during spikelet development and flowering. (**A**,**B**) Semi-quantitative RT-PCR and quantitative real time PCR (qRT-PCR) analysis of *BdSBP* genes at 7, 14, 20, and 24 DAH stages of spikelet development. *miR156 targeted *BdSBP* genes. (**C**) *Brachypodium* accessions Bd21 (early flowering) and Bd1-1 (delayed flowering) under vernalization and non-vernalization time course. Bar represents 1 cm. (**D**) Semi-quantitative RT-PCR analysis of *BdSBP* genes in vernalized and non-vernalized *Brachypodium* accessions Bd21 and Bd1-1. BdUBC18 was loaded as internal control.

In silico expression analysis revealed higher expression of *BdSBP* genes during spike emergence and in early inflorescence development (Fig. 4). Therefore, the transcript abundance of 9 *BdSBPs* was examined during different developmental stages [7–24 Days after Heading (DAH) of *Brachypodium* spikelet] (Fig. 8A,B; Supplementary Fig. S3). Five genes including *BdSBP3,* *BdSBP16,* *BdSBP17,* *BdSBP18,* *BdSBP21* and *BdSBP23* were highly abundant during early spikelet development (7 DAH), as compared to mid-phase (15–20 DAH) or maturation phase (24 DAH). However, the transcript level of *BdSBP1* was constant at 7, 15 and 20 DAHs except at 24 DAH. No change in transcript level of *BdSBP15* was observed at any of the above-mentioned developmental stages. Expression pattern of *BdSBP9/16/17/18* was also confirmed by qPCR (Fig. 8B). Expression of *BdSBP9* was slightly lower at 15DAH as compared to 7 and 24DAHs.

To ensure the reproductive success, flowering is the critical stage of plant reproduction, which is mainly regulated by gibberellin, vernalization, photoperiod and autonomous pathways. Vernalization promotes the flowering in alpine species and its molecular mechanism has been investigated in *Arabis* *alpina* and *A.* *thaliana* plants (Bergonzi et al*.*^51^). Therefore, to understand the genetic control of vernalization response in grasses, we analysed the expression pattern of *BdSBP* genes in *Brachypodium*. Transcript abundance of several *BdSBP* genes was compared in the rapid flowering (Bd21) and delayed flowering (Bd1-1) accessions of *Brachypodium* under vernalization and non-vernalization conditions (Fig. 8C,D; Supplementary Fig. S4). We observed that Bd21 accession flowered rapidly under non-vernalised condition, whereas Bd1-1 lacked flowering until maturity. However, both the accessions produced flowers with 6-weeks vernalisation at 4 °C. *BdSBP1* and *BdSBP3* expressed differentially in these accessions following vernalization or non-vernalization. The expression of *BdSBP1* and *BdSBP3* were found to be lower in Bd1-1 as compared to Bd21 under non-vernalized condition. Whereas, under vernalized condition, no change in the transcript level was observed suggesting their possible role in flowering time and spikelet development. However, transcript level of *BdSBP9/16/17/21/23* was not altered significantly under vernalization condition.

**Figure 9:**
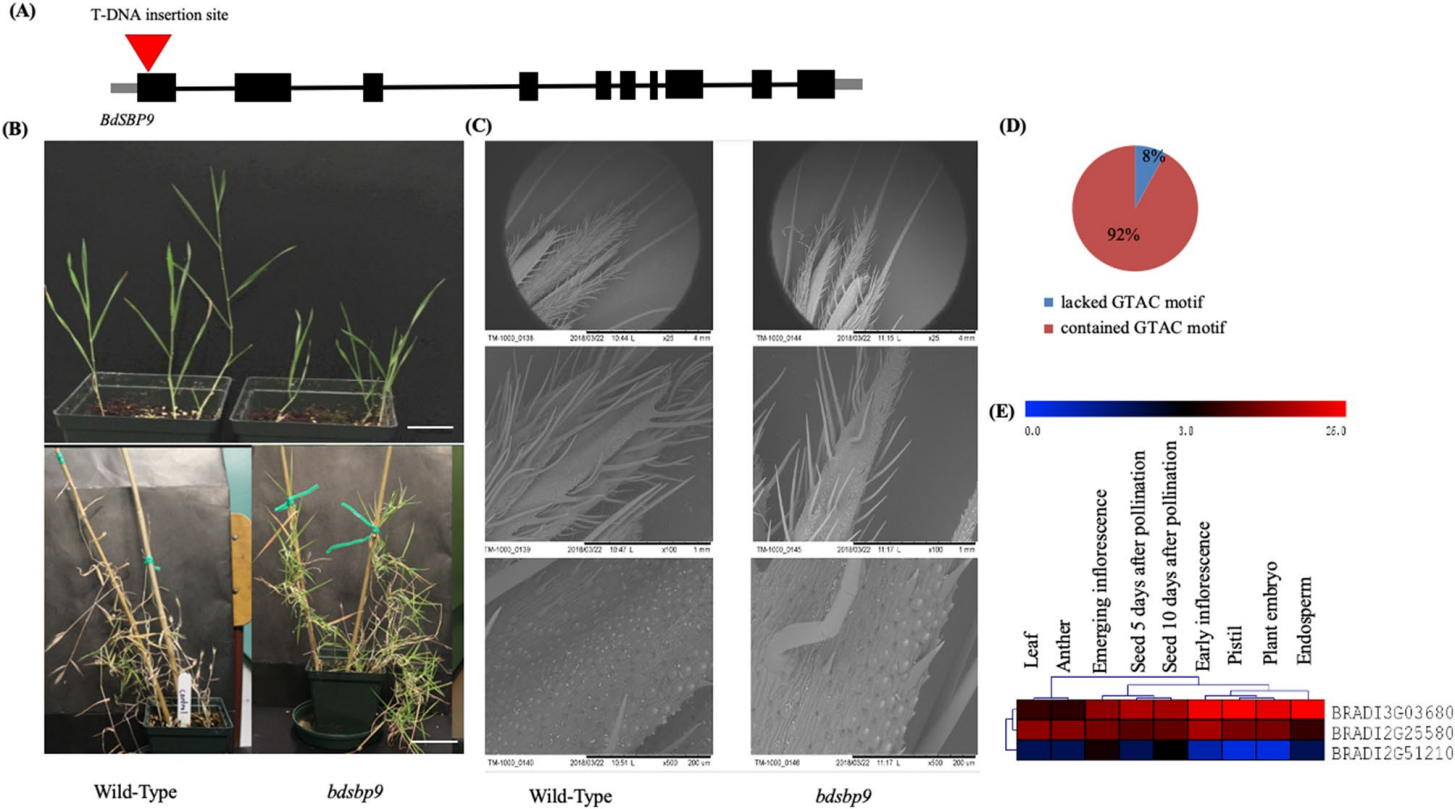
Shoot and spikelet phenotype of *Bdsbp9* mutant. (**A**) Schematic diagram of the T-DNA insertion line for *BdSBP9* gene. The insertion was within first exon of the gene. (**B**) Comparison of shoot growth in Bd21-3 wild-type and *Bdsbp9* mutant at vegetative and reproductive stage. Bar represents 1 cm. (**C**) Scanning electron microscopy (SEM) analysis of the terminal spikelet of Bd21-3 wild-type and *Bdsbp9* mutant. The SEM was performed at three resolutions at ×25, ×100 and ×500. Bar represents 4 mm, 1 mm and 200 µm. (**D**) Promoter regions analysis of BdSBP9 co-expressed genes for GTAC binding motif. (**E**) Heat map based expression analysis of BdSBP9 interactor proteins.

To further confirm the function, T-DNA mutant for *BdSBP9* gene was obtained from JGI (Fig. 9A–C). The *Bdsbp9* mutant has a T-DNA insertion in the first exon of *BdSBP9*. Electron microscopy indicated that different patterns of lignification in the wild type as compared to *Bdsbp9*. Wild-type patterns were straighter, with no circular patches whereas, *Bdsbp9* patterns are less uniform, with some circular patches. Further, we investigated the promoter region (1000 bp upstream of initiation codon) of the co-expressed genes of *BdSBP9* (Fig. 9D). Data indicate that 92% of the co-expressed genes contain GTAC motif, a specific binding site for *SBP* genes. The expression of one of the interacting partners matches with *BdSBP9* expression pattern (Fig. 9E).

## Discussion

SBP/miR156 genetic circuit controls the transition of vegetative to reproductive phase change in *Arabidopsis*^14,52^. Owing to the importance of *SBP* genes, we conducted the first-ever genome-wide identification of this gene family in *Brachypodium* and discovered 18 *BdSBP* genes (Table 1). The number of *SBP* genes in *Brachypodium* were similar to the *SBP* genes in barley (17), *B.* *luminifera* (18), rice (19) and *Arabidopsis* (17), but was smaller in comparison to soybean (41), moso bamboo (32) and *P.* *trichocarpa* (28), suggesting that *SBP* genes were evolved in a species specific manner and underwent different gene duplication events. On the basis of phylogenetic analysis *BdSBP* genes were divided into eight (G1–G8) groups (Fig. 1A). *BdSBP* genes grouped closely with *HvSPLs* and *TaSPLs*, suggesting that these *SBP* genes possibly diverged from a common ancestor. The DNA binding SBP domain binds to the promoter regions of its target genes containing TNCGTACAA consensus nucleotide sequence with GTAC as a core motif^53^. Two zinc ion binding motifs Cys3His1 and Cys2His1Cys1 at N terminus and a nuclear localization signal (NLS) at C terminus were found in BdSBP proteins (Fig. 1B). *SBP* genes share similar gene structures within their same phylogenetic group as mentioned previously in barley^13^, rice^11,54^, and tomato^55^. Gene duplication events are key to evolution and gene expansion which produce many paralogous gene pairs^56^. Additionally, gene duplication also assists organisms to cope up with different environmental conditions during growth and development^57^. In order to study gene duplication, we estimated the Ka/Ks ratio for each duplicated genes using Plant Genome Duplication Database and PLAZA 4.0 (Fig. 2A,B), which suggests that *BdSBP* genes underwent duplication event ~ 74 to 164 mya. The Ka/Ks ratio of > 1 shows that the gene has experienced positive selection, = 1 indicate neutral selection and < 1 indicates purifying selection or negative, respectively. Based on Ka/Ks ratio^56^; the *BdSBP* gene pairs which were ranged from 0.2 to 0.7 suggesting that these genes were duplicated under purifying selection. Also, *BdSBP* genes shared similar intron/exon structures within the same phylogenetic groups (Fig. 3A). Additionally, most of the BdSBPs from the same phylogenetic groups possess similar motifs (Fig. 3B). Consequently, the genes in the same phylogenetic group might have similar roles in *Brachypodium*, which have been supported by gene ontology terms of *BdSBP* genes (Fig. 3C). In addition to conserved BdSBPs motifs, several unique group-specific motifs were observed, such as motif 4, 9 and 10 in group 1 and motif 8 in group 8. These specific motifs might be important for specified roles of *BdSBP* genes, and their functional differentiation could arise during evolution of different lineages.

All *BdSBP* genes expressed substantially during early and emerging inflorescence development except, *BdSBP1* and *BdSBP7*, implying their role in inflorescence development of *Brachypodium* (Fig. 4)*.* Most of the *BdSBP* genes except, *BdSBP7/8*/*17*/*21*, expressed significantly in pistil whereas *BdSBP3*/*6/11/* *14/15*/*16* expressed highly in the anther, suggesting their role during reproduction. However, *BdSBP6/9*/*15* were constitutively expressed in all the 9 tissues. Previously, differences in expression profiles of miR156 targeted and non-targeted *SBP* genes have been reported in barley^13^, *Brassica* *napus*^58^, Betula^59^ and soybean^12^. Importantly, miR156 targeted *BdSBP* genes showed differential expression pattern and most of the miR156 non-targeted *BdSBPs* showed constitutive expression profiles in *Brachypodium*. Post-transcriptional regulation of *SBP* genes through miR156 has been considered as the key process for the functionality of these genes^27,29,60^. A total, 11 of 19 *SBP* genes in rice^11^, 10 of 17 *SBPs* in *Arabidopsis*^52^, 7 of 17 *SBPs* in barley^13^, and 18 of 28 *SBPs* in *Populus*^8^ have been identified as targets of miR156. In our study, miRNA target prediction revealed that 10 *BdSBP* genes are regulated by miR156 (Fig. 5A). A total 8 (*BdSBP3,-11,-13A,-14,-16,-17,-18*, and *23*) of 10 miR156 targeted *BdSBP* genes contained miR156 complementary sequence in their coding region whereas, *BdSBP1* and *BdSBP13* contained target site in 5′ and 3′UTRs, respectively. Thus, miR156 targets *BdSBP1* and *BdSBP13* along with other *BdSBP* genes will be unable to perform downstream roles. This phenomenon of binding of miRNAs to their complementary sequences in the coding sequences or un-translated regions of target genes to inhibit gene function either by transcript cleavage or deadenylating has also been reported elsewhere (Rhoades et al*.*^61^).

In humans ~ 95% and in *Arabidopsis* > 60% of multi-exonic genes undergo AS^62^. Meanwhile, we noticed that miR156 targeted *BdSBP* genes produced different splice variants via*.* AS (Fig. 5B). AS generally produces transcripts with premature stop codon which are degraded in cytoplasm by non-sense-mediated decay (NMD) pathway^63^. Splice variants produced by AS generally exhibit spatiotemporal or environmental condition-specific expression patterns^63^. Our experiments in *Brachypodium* showed that *BdSBP* gene products are degraded at higher level by miR156 in the leaf as compared to the spike (Fig. 5C,D), resulting into higher transcript abundance of miR156 targeted *BdSBP* genes in young spike as compared to leaf (Fig. 5E; Supplementary Fig. S1). However, expression of miR156 non-targeted *BdSBP9* and *BdSBP15* genes was constitutive in these tissues. This confirms that miR156 negatively regulates *SBP* genes in *Brachypodium* and is consistent with previous findings in *Arabidopsis*, rice, tomato and wheat^11,29,35,52,64^. Taken together, these results suggest that miR156 in conjunction with AS regulates the transcriptome dynamics.

*SBP*-correlated gene network and interactome analysis revealed that *SBP* genes function by regulating other families of transcription factors and membrane transport proteins, and are involved in the metabolism of glucose, in-organic salts and ATP production in *Arabidopsis*^65^. Therefore, considering the significance of *Brachypodium* as a model plant for developmental biology of triticeae crops, we examined the co-expression and MapMan biological pathways (Fig. 6A,B; Supplementary Table S5). MapMan terms enrichment analysis showed that *BdSBP* genes perform their function by regulating transcription, protein, signalling, transport and development related biological pathways (Fig. 6B). The co-expression network contains mainly transcription factors, hormones (auxin, brassinosteroid, ethylene and gibberellin) responsive genes, cell wall biogenesis related genes and transporters, implying their roles in development as well as cell wall biogenesis of *Brachypodium*. Existence of CSLF3 and MYB TF indicate that *BdSBP* genes might be involved in secondary wall synthesis in *Brachypodium*^66^. Studying the protein–protein interaction network represents gene functions crucial to plant physiology, pathology, and growth^67^. Protein–protein interaction at the molecular level might be important in transcription regulation, post-transcriptional modification, cytoskeleton assembly, phosphorylation, acetylation, transporter activation and others^68^. Previously, it was found that IPA1 (OsSPL14), an important factor which controls plant architecture interacts with D53 protein (DWARF53) in-vivo and in-vitro^69^. Recently, OsSPL14 protein has been shown to be associated with disease and yield in rice by phosphorylation and non-phosphorylation of Ser^163^ amino acid respectively during *Magnaporthe* *oryzae* fungal infection^5^. In our study 39 interacting proteins with 9 BdSBP proteins were identified (Fig. 7A,B; Supplementary Table S6). These interacting proteins mainly belonged to bZIP, Homeobox, MYB33, ZnF_C2H2, F-box and heat shock transcription factor families, Dicer-like proteins and protein kinases. These interacting protein partners have been involved in the regulation of the biological pathways including development, RNA, protein, stress, photosynthesis and cell wall, implying the diverse roles of BdSBP proteins in *Brachypodium* growth and development.

The grain development and filling of *Brachypodium* spikelet are completed (dry) in 50 days and has been classified into three stages namely-embryo and endosperm development [0–14 days after fertilization (DAF)]; maturation (14–36 DAF) and desiccation (36–50 DAF) stages^70^. Higher expression of *BdSBP1/-3/-16/-17/-18/-21* and *23* at spikelet initiation stage as compared to the maturation stage, might be key to early spikelet development in *Brachypodium* (Fig. 8A,B). Further, *BdSBP9* and *BdSBP15* genes exhibited constitutive expression pattern during embryogenesis and maturation stages, suggesting their importance for these stages. Plants bear flowers at a certain time of reproductive phase which is mainly regulated by SBP/miR156 pathway^29^. As plants grow older, the level of *SBP* genes increases while miR156 abundance declines. Previously, it was reported that higher production of *SBP* genes ensures flowering in response to cold in the model perennial *Arabis* *alpina* accession Pajares^51^. It has been reported in *Cardamine* *flexuosa* that SBP/miR156 pathway plays a key role in flowering through integrating age and vernalization pathway ^71^. The SPL/miR156 module has been known to be a key component for flowering phases^1,52^. Involvement of *SBP* genes in the control of flowering time of *B.* *distachyon* accessions Bd21 and Bd1-1 under vernalization condition (Fig. 8C,D) suggest that *BdSBP1* and *BdSBP3* potentially involved in this. This result positively supports the previous study about sensitivity of certain *SBP* genes to vernalization in older plants^51^. Cereals inflorescence (spike) architecture is one of the main determinants of their yield. In rice, *SBP* genes have been reported as an important regulator of plant architecture. Overexpression of *OsSPL14,* present on the IPA1 (ideal plant architecture)/WFP (wealthy farmer’s panicle) QTL, decreased tiller branching but increased panicle branching and grain weight^18,36^. Likewise, *OsSPL7*, *OsSPL13* *OsSPL16* and *OsSPL17* also regulate grain size, shape and yield in rice^16,37,38^. In our study, the *Bdsbp9* mutant showed abnormal spike and delayed flowering, implying its role in spike development (Fig. 9A–E). In *Arabidopsis*, miR156/SPL module confers thermotolerance at reproductive stage[^24^,^72^). Our study also indicates that *BdSBP* genes contribute thermotolerance during spike development in *Brachypodium.* Interestingly, differential expression of *BdSBP* genes in the developing spike under variable temperatures was not been associated with ploidy level in *Brachypodium* genome as described previously^49^. However, specific expression of these genes in response to high temperature in tetraploid genome, *B.* *hybridum,* probably induced by interaction of *B.* *distachyon* and *B.* *stacei* genomes (Fig. 7C; Supplementary Fig. S2). Overall, our study revealed that altering the expression pattern of *BdSBP* genes may provide an important tool-box for the genetic improvement of the cereal crops.

## Materials and methods

### Identification and annotation of *SBP* genes in *Brachypodiumdistachyon*

To identify *SBP* genes in *Brachypodium* *distachyon* genome, pHMMER search was performed on EnsemblPlants database (https://plants.ensembl.org/Brachypodium_di/Info/Index) using *A.* *thaliana* SBP domain (Pfam: PF03110) sequence as the query^13,73^. Additionally, phytozome (https://phytozome.jgi.doe.gov/pz/portal.html#!info?alias=Org_Bdiulgare_er) database was also mined through TBLASTN using SBP domain amino acid sequences. The accession numbers of putative *BdSBP* genes were taken from databases and were named based on their closest homologs present in barley, wheat and rice. Further, EnsemblPlants database (https://plants.ensembl.org/Brachypodium_di/Info/Index) was used to obtain the genomic sequences (Table S1), coding sequences (Supplementary Table S2) and protein sequences (Supplementary Table S3) of *BdSBP* genes.

### Gene structure and phylogenetic analysis of *BdSBP* genes

The exon/intron structure of each *BdSBP* gene was predicted through gene structure display server program (https://gsds.cbi.pku.edu.cn/index.php) by comparing their coding and genomic sequences. The TAIR (https://www.arabidopsis.org/index.jsp) was used to obtain the *Arabidopsis* *SBP* sequences and rice genome annotation project database was used to obtain the rice *SBP* genes sequences. *SBP* sequences of wheat were obtained from a previous study^74^. SMART tool was used to identify SBP domain sequences from *Brachypodium*, rice, wheat, and *A.* *thaliana* which are presented in Supplementary Table S4. A phylogenetic tree was annotated using the Interactive Tree of Life resource (https://itol.embl.de). SBP domain sequences were aligned using MUSCLE tool followed by Gblocks curation utilities and maximum likelihood method was used to construct the phylogenetic tree using PhyML software (https://www.phylogeny.fr).

### Motif identification, miR156 target site prediction and alternative splicing event analysis

The MEME 4.11.0 tool (https://meme-suite.org/tools/meme;) was used to search for conserved motifs within BdSBP proteins by using default settings, except that the maximum number of motifs to find was 10, the maximum width was 50 and the minimum width was 6. The sequence logo of the *Brachypodium* SBP domain was created with an online available WebLogo3 platform (https://weblogo.threeplusone.com/). The cDNA sequences of *BdSBPs* were subjected to psRNATarget tool (https://plantgrn.noble.org/psRNATarget/?function) to predict the putative target sites of miR156. The Ensemble database (https://plants.ensembl.org/Brachypodium_distachyon/Info/Index) was used to obtain the information on alternative splice events for each *BdSBP* gene (Supplementary Table S5).

### Gene expression analysis of *BdSBPs*

The log2-transformed fragments per kilobase per million fragments measured (FPKM) values were used to study the expression of *BdSBPs* in nine tissues as described^13,74^. A heat map of the expression of *BdSBPs* was generated by the average hierarchical clustering method using the MeV tool (https://www.tm4.org/mev.html).

### Co-expression, protein–protein interaction and gene duplication analysis

The co-expressed genes for *BdSBP* members were identified using online PlaNet (https://aranet.mpimp-golm.mpg.de/index.html) tool^76^. PlaNet uses the Pearson correlation coefficient (PCC) and constructs a co-expression network, with PCC cut-off to 0.7^77^. Further, a highest reciprocal rank (HRR) co-expression network with standard edge cutoff of 30 was used. Additionally, a heuristic cluster chiseling algorithm (HCCA), which is optimized for HRRbased networks was used with standard parameters (stepsize = 3). The protein–protein interaction network was identified using STRING database (https://stringdb.org/cgi/input.pl?sessionId=A92xEG08sQEk&input_page_show_search=on), which contained information from various datasets such as; gene coexistence, protein–protein interactions, gene fusion and co-expressed genes to calculate the semantic links between proteins^78^. The genome-wide genomic duplication files of *B.* *distachyon* were retrieved from the plant genome duplication database (PGDD) (https://chibba.agtec.uga.edu/duplication) and PLAZA4.0 (https://bioinformatics.psb.ugent.be/plaza/versions/plaza_v4_dicots/,^79^. s*The synonymous substitution (Ks) and non-synonymous substitution (Ka) rates were obtained from PGDD and the ratios of Ka/Ks were used to assess the selection pressure for duplicated gene events.

### Plant material and sample preparation

*Brachypodium* seeds from Bd21-3, Bd21, *B.* *hybridum*, *B.* *stacei* and Bd1-1 accessions were obtained from Prof. John Vogel (DOE Joint Genome Institute, CA, 94598 USA). Seeds were imbibed in water overnight, dried and stratified at 4 °C in the dark for 1 week. The daily temperature was 22 °C and the photoperiod was 16-h-light/8-h-dark (long day). The *Bdsbp9* mutant line JJ12467 was obtained from a *Brachypodium* T-DNA insertion library^80^; Prof. John Vogel’s lab; JGI). The T_3_ *Bdsbp9*-mutant seeds were advanced for two further generations using conditions described above and according to method by^81^. For the vernalization experiment, Bd21 and Bd1-1 seeds were vernalized for 6 weeks at 4 °C. For heat stress study, *B.* *distachyon*, *B.hybridum* and *B.* *stacei* seeds were grown under 22 °C and 42 °C for 2 h. Further, immature spikes were collected from each accession to elucidate transcript abundance of *BdSBP* genes. To study the expression pattern of *BdSBP* genes in *Brachypodium* spike development, tissue samples were collected from leaf, and spike tissue at 7, 14, 21, and 25 days after heading (DAH).

### Genomic DNA and RNA isolation

Leaves from *Brachypodium* *distachyon* plants were collected and DNA isolation was performed using cetyl-trimethyl-ammonium bromide-based (CTAB) extraction method as described elsewhere^82^. PCR-based genotyping was performed using primers following recommendations from Joint Genome Institute^80^. The spectrum plant total RNA Kit (Sigma-Aldrich, St. Louis, MO, USA) was used for RNA isolation following the manufacturer’s protocol. The RNA integrity and purity of all samples were verified on a Nanodrop ND-1000 (Nanodrop Technologies, Wilmington, DE, USA). Prior to synthesizing cDNA, RNA samples were treated with DNase I to remove genomic DNA contamination (Invitrogen, USA). The reaction mixtures were incubated at 23 °C for 15 min and after that 1 µl of 25 mM EDTA was added to each sample.

### First strand cDNA synthesis and quantitative real-time PCR (qRT-PCR) analysis

First strand cDNA was synthesized from 2 µg total RNA sample using AffinityScript QPCR cDNA Synthesis Kit (Agilent technology, Canada). The qRT-PCR was run on Mx30005p qPCR system (Stratagene, USA) in a 20-μl volume containing 5 µM gene-specific primers, 1 μl diluted cDNA, and 10 μl Brilliant III Ultra-Fast SYBR Green QPCR Master Mix (Agilent, USA). Two biological and three technical replicates were used in all the experiments. The 2^−ΔΔCq^ method was used to quantify the relative level of gene expression (Livak and Schmittgen 2001). The gene-specific primers for *BdSBP* genes used in semi-quantitative RT-PCR and qRT-PCR are listed in Supplementary Table S7. PCR was performed in a 20 μl volume using GoTaq Green master mix (Promega, USA). *BdUBC18* (*Ubiquitin-conjugating* *enzyme* *18*) was used as a reference gene for different developmental stages and *SamDC* (*S-adenosyl* *methionine* *decarboxylase*) was used for heat stress^83^.

### Scanning electron microscopy (SEM)

Immature spikes (14 days after anthesis) were collected from *Bdsbp9*-mutant and control plants. Samples were fixed in 25 mM phosphate buffer (pH 7.0) with 3% glutaraldehyde overnight. Dehydration of the tissue was carried out by increasing the ethanol concentration of the solution every hour (30%, 40%, 50%, 60%, 75%, 90%, 100%), keeping the samples in 100% ethanol for two days. Samples were critically point dried using the Leica EM CPD300 and coated with platinum using the Leica EM ACE200. The samples were visualized using Hitachi TM1000.

### RNA ligase-mediated modified 5′ rapid amplification of cDNA ends (RLM-RACE)

The miR156 mediated cleavage site in the *BdSBP* transcript was mapped by using First-choice RLM-RACE kit (Ambion, Austin, TX, USA). Total RNA was isolated from leaf and 7 days old spike (after heading). Without pre-treatment, 1 μg total RNA was ligated to the 5′ RACE RNA adapters. The M-MLV reverse transcriptase enzyme and 18mer oligo dT were used to reverse transcribe the adapter-ligated RNA. Primary and secondary nested PCR was carried out using 5′ RACE gene-specific outer and 5′-adapter outer primers and 5′ RACE gene-specific inner and 5′-adapter inner primers. The primer sequences used in nested PCR are listed in Supplementary Table S5. The 5′ RACE PCR amplified fragments were gel extracted and cloned into pGEMT^easy^ vector. Further, clones were confirmed by EcoRI restriction analysis and Sanger sequencing.

## Supplementary information

Supplementary Information

## Abbreviations

SBP: *SQUAMOSA*-Promoter binding like transcription factor
miR156: MicroRNA156
qRT-PCR: Quantitative real-time PCR
WT: Wild type
