# Supplementary material for: Global analysis of SBP gene family in Brachypodiumdistachyon reveals its association with spike development
Source: Sci Rep. 2020 Sep 14;10:15032. doi: 10.1038/s41598-020-72005-7 (PMC7490389; doi:10.1038/s41598-020-72005-7)
Supplement: Supplementary file 1 — Supplementary Information [file 41598_2020_72005_MOESM1_ESM.pdf]

Global analysis of *SBP* gene family in *Brachypodium distachyon* reveals its association with spike development

Rajiv K. Tripathi*[1], William Overbeek[1] & Jaswinder Singh*[1]

[1]Plant Science Department, 21111 Rue Lakeshore, McGill University, Quebec, H9X 3V9, Canada

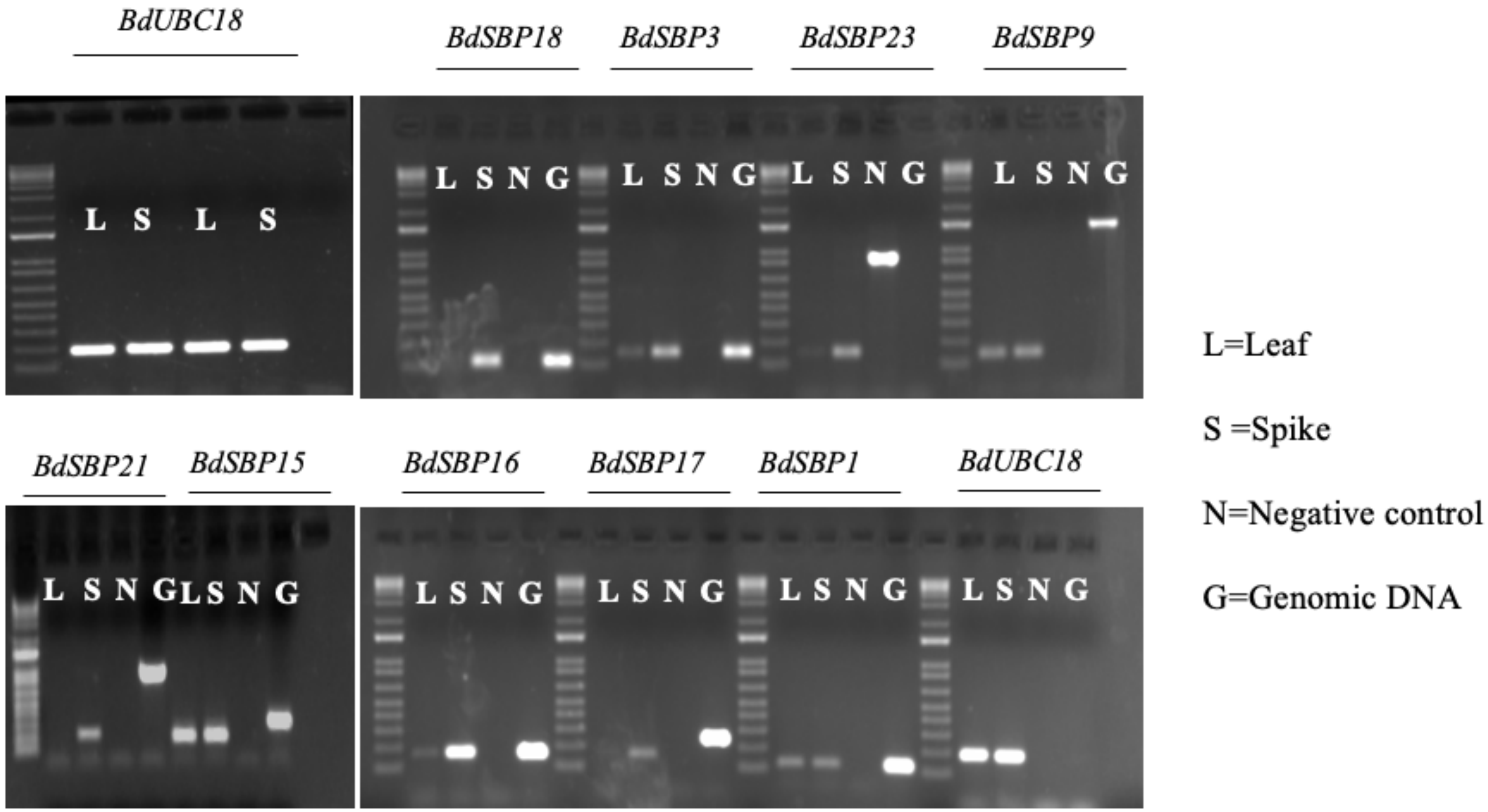

Supplementary Fig. 1. Full length gel image of transcript level of *BdSBP* genes in Leaf and Spike tissues.

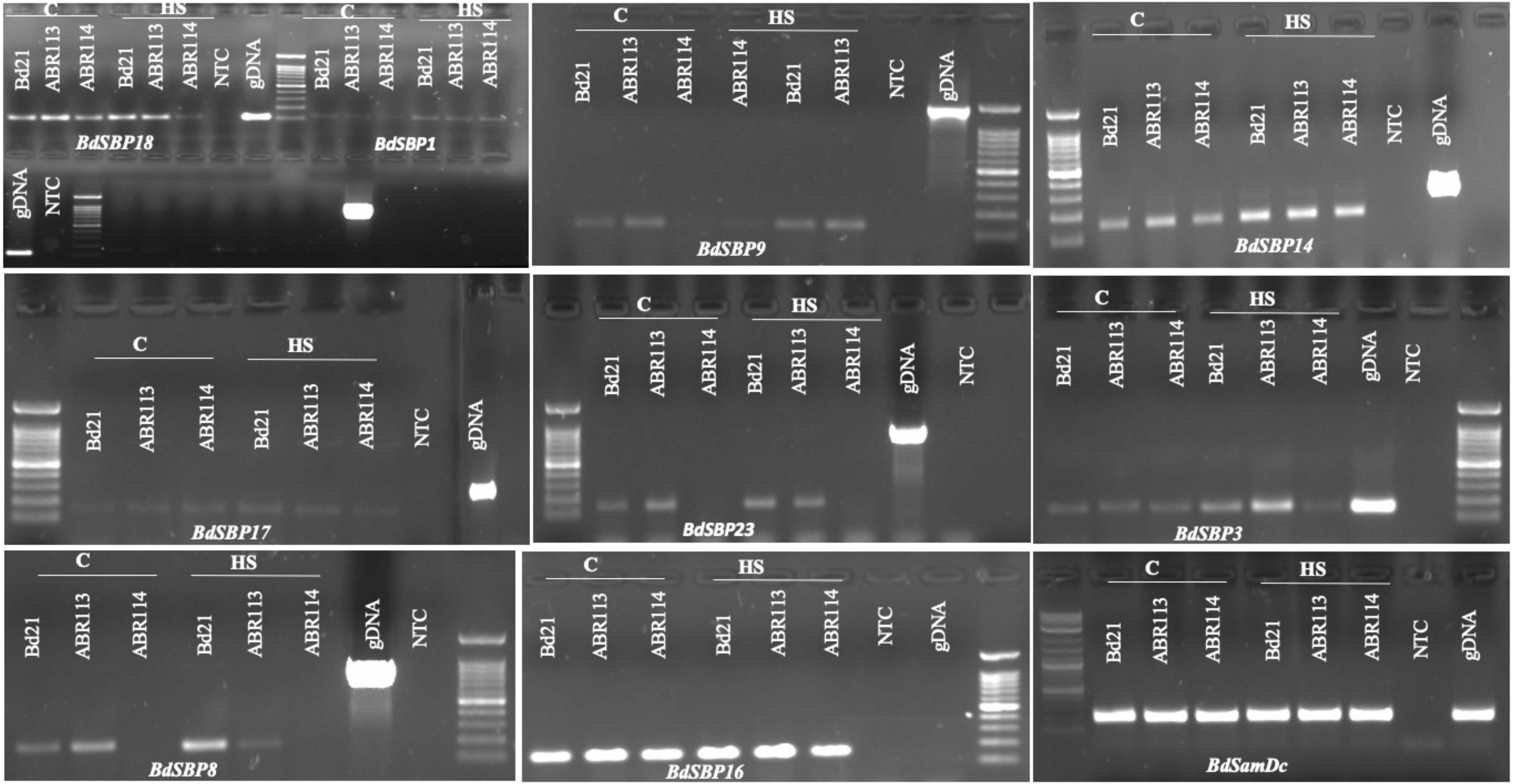

Supplementary Fig. 2. Full length gel image showing transcript level of *BdSBP* genes in *B. distachyon, B. stacei* (ABR114), *B. hybridum* (ABR113) accessions of *Brachypodium* under control and heat stress conditions. C: control; HS; Heat stress.

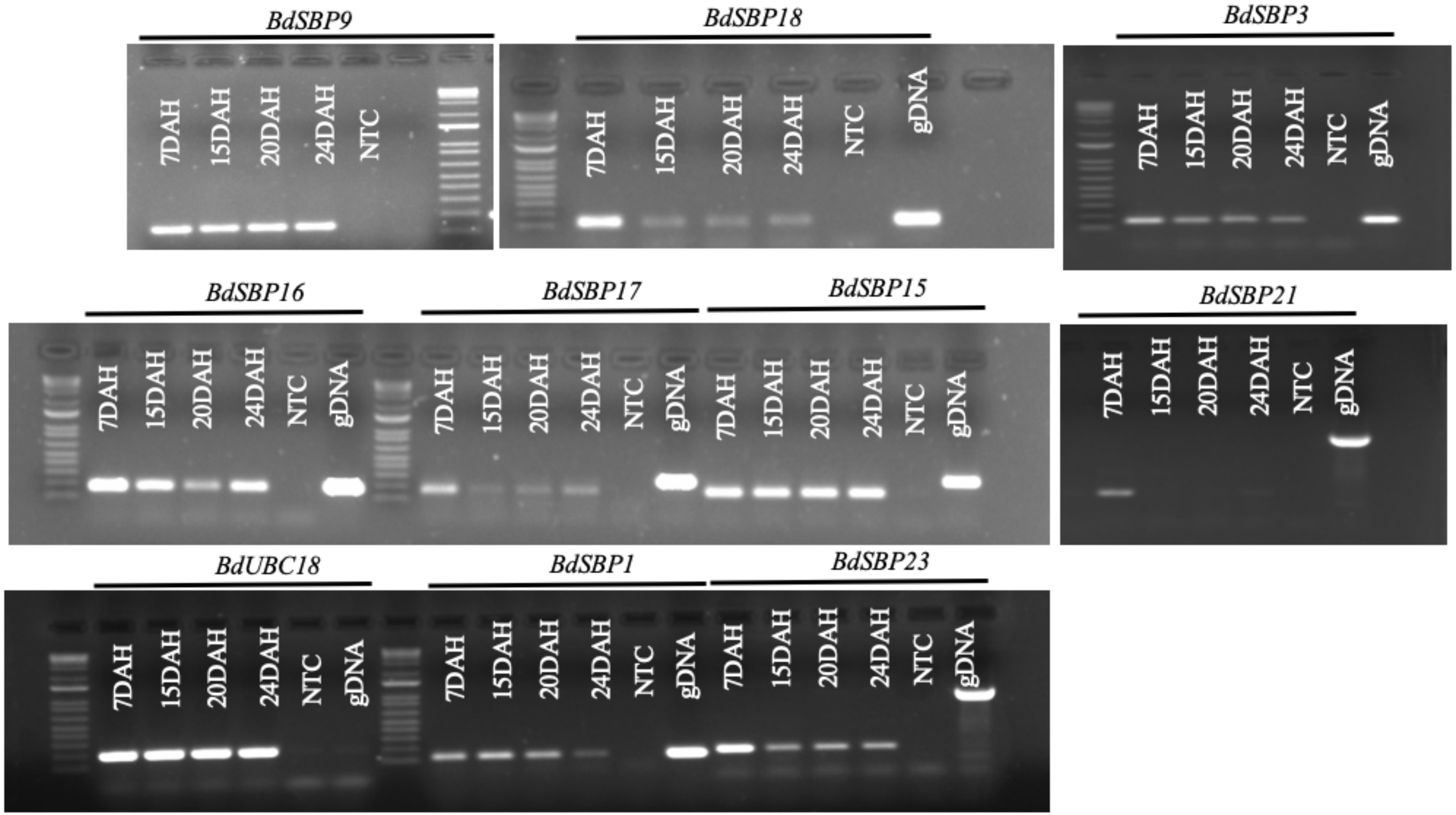

Supplementary Fig. 3. Full length gel image of transcript level of *BdSBP* genes in different stages of spikelet development

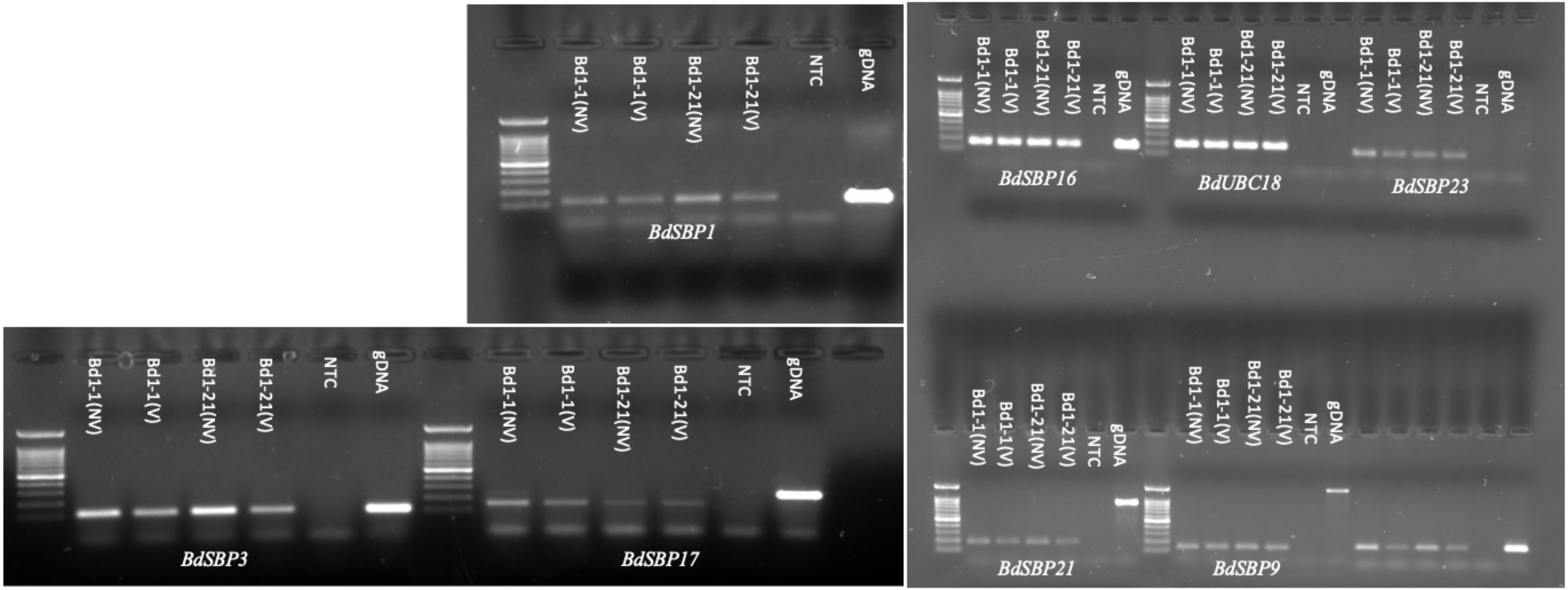

Supplementary Fig. 4. Full length gel image showing transcript level of *BdSBP* genes in Bd1-1 and Bd21 accessions of *Brachypodium* under vernalization and non-vernalization conditions.

Table S1. Genomic sequence of BdSBP genes

>BRADI1G02760
ATTTCAATTTAAACGGGACAACCGTGCCGGGCGCAGTTTGTTGTGTAGCCACACGGTGTG
TCCACACAGAAAATCCGGGGCCCTATTTGTACTAGCCCGTCGCGCTACGGGTCCCCATCG
CCTCCACCATCTTTTGCTCTTCTCTCTCCCTTTCCTCTGTTCTCCCCTCGTCCTCCTTCA
CTCCCACCAGTCCACCATCCCCAACTCCCGAAATTCGCCAACTGCTCTCCGCGGAATTCG
CCCGATTCGGCCCTCGCCGGAGCTCGATTCGTGCCCGAATCCGCCGCCAATTCGCGCCGC
TCCGTTTTGCCGGCTCAGGTGACCCCCGCTTGCCCCCCTCTCTGTTTCGGTCTCCCCGCT
GTGTGATTCCAGCCTCTGCGTGGTTGATTATTCCCCACTGGAGGCGCGAGGGCATGTGGG
TCTCGCTGGTTTGGATCCGTGGCTGCGTGGGCGTCACGTTCGTCGACTGCTTGGGCGGGA
GGGGAGGGGACTAGATCTCTCGGCTCCTGCGGCTGCCGTACATTTTTTTTTGGGAGGGTT
GAACTTGAAGAAGGTTGGTTTGGATAGGAGGTTCTTCTAGGGTTTCGGTGGGGGCGGGCC
ATGGAGGCGGCCGGGGTCGGGTCGCAGAGCCGCCGGCTGTACGGCGGCGGGTTAGGTGAG
CCTGCCCAGGACATGCGTGGGAAGAGGCTGTTTGGCTGGGACCTCAATGACTGGAGCTGG
GACAGCGAGCGCTTTGTCGCCACCCCGGCGCCTGCAGCAGAAAAGGCAAATGGCCTGTCA
CTGAATAGTTCGCCGTCTTCCTCTGAGGAAGCGGACGTCGAGGTGGCTAGGAGTGGTAAC
GTGAGAGGTGATTCTGATAAGAGGAAGCGGGTGGTGGTCATTGATGATGACGGTGATGAC
CAGAAGGATGAGGACCCTGTGGATAACAATGGCAGGGTGCTCAGCTTGAGAATTGGGGGA
GACACTACTGTTGCCGGGGGAGCGGTGGAGGGTGGTGCCGTTAATGAGGAGGATAGAAAT
GGCAAGAAGATCAGGGTGCAGGGGGGAAGCTCAAGTGGCCCAGCTTGTCAGGTGGAGGGC
TGCTGCGCGGACCTTAGTGCGGCGAAGGATTACCATCGCCGGCACAAGGTCTGCGAGATG
CATGCTAAGGCCAACACTGCGGTGGTCGGAAATACTGTCCAGCGCTTCTGCCAGCAATGC
AGTAGGTCAGTTTTACAATTGAGCTGCTATTACCTTTTATTTACATGTAATAATTTTGTT
TGTGTGGGCAATTTATATGTAGAATATGTTATGCAAGTTTTATTTTGTCTTGCCAAAATA
GATTTGATATCTGAGACCATGATGATGTTTTAGGCAATAACATTTGATATCTGAGATTCA
CTATAATCTATGTCGGCAATGTTAATGCAAGTCCTCATTTGTAGACTGAAAAGGCATGTA
GAAATTGTTTTCCAAGAAACTTTTAGTATAAATAGTTTAAAAAGGGACATCCAACTTCCA
TACAGGCTTATGCATGATTTAGTTATGACGTTGTTGCTGGGAGCAAGTGGTCACATAGTC
ATGCTAATAGAGGTTAGTTTTGCGTCAATTCTCTCTTGTGAATACAGCACGTAATTCACT
GTGCACGAAAGGAGGGTGGCGTGGCAAATATGAATCCATATACATACGAATATATTCTGA
AGTGAATTTCTATATCAGGATTTACGAATTTGGTTTATCGAATTTATGCGAGTTAGTATA
AGTTATAACCATTTGCTAAGTAGAAATGCACTGTTATACTCATCAGTTATCCCTTTTCAA
TTCAACAGAATCGATATAATTTTTTTTACTTCGATATGTATGAGGATTAGCTTATGCTGA
TAAACTCGGAAACCTCCGTGGTACTCAGATATTTTTTTTTAAAAAAAAGAAACTTTGGAA
TATGCTGTTCTCAGCAACCAAGCCCGGTTTCCAGAAAGCTTGGTTTTTTGTTTGGTACAT
AATTTTTTTCACAGTCCAAATTTCCAACTACACTGTTAATAATTTTTTCCACACAATGTA
CTTGTTACATCAGCACCTAAATTTACCGCATATATTAGACAGCACATGACCAACAATAAT
GGTCTTTTGTGCCTCACCCTTATTTCGTGTTCTTGGAGAACTTATATCTCTAGAGACACT
GCCCACTTTTAGGAAATACTGAATTCATCCTGGCCTGTGCATAGCCAAGGACTACTTTTG
GTAGCATTTGATTTATATAATTACGTGATGTATTTTTTCTGTTATAATCATAGAGCACAC
ATTGTGTGCATAGCGTATTGTGTGGCTAAAGATATATCTATGGAGCTTTGTTGCTGCTGC
CACTTTATACATACATTTTAAATTTAGCAATCCTCCTGACTACTGTACATGTGCAGATTT
CACCTTCTTCAAGAATTTGATGAAGGAAAGCGAAGCTGTCGTCGGCGTTTAGCAGGTCAT
AATAGACGGAGGAGGAAAACCCGCCCTGAAATTGCTGTTGGTGGGACTCCTATTGAGGAT
AAAGTTGGCAGTTATTTAGTGTTGAGTCTTCTTGGAATATGCGCCAATTTGAACTGTAAG
CAATATTTTGTAGGTTTCAGGCTGACTAATTTACTAGAGTTAAATACACAGCTAACTGGT
CTGCATATAAATGTCATGGTACTTGTATAGCTGATTTCCCCTTAAATGACTTGAAATTAT
GTTGACTTATAGCTTTGACTGTATTTTACTTGGTAGTTAGTTTTAACCGAAAAGAGAACT
TATGATGGTGATCTGATTTTTTTTCTTCTGGTAGATGCAGTTACAACATGAGTGTCAAGG

CTCTTGCTGATTTTTGAGATATGTTGATTGAAATAGTAGCGTTTCTAGGATCAAAAAGGC
TATATAATGCGGTACTGGGCGTAGTTGAAGTTAACTATTGCTTGTCATCCAATTTCTGAA
TTGCTTGCAGGAGGCTCCGCCGCCAGCCTGTGTGACGAGTCCATCATATTTCTTAAATTT
TCACCACCCTTGTAAAATCTTTGTACTTTGCTGGTTATTATCTGTTGTCTATGATAGCTT
ATCTCTTAATTATAGCATAGGAACAGTTTCTTTGCCTGAACTGAACTATTATCAGCATAC
TTAATGCCCTTCAGAACTACTCTTATGGTAATACTCAGGAAGAGACATGACTTTTAGCAT
TGTACTTGCAGCTGAGAATGCTGAGCATTTACAAGGTCAGGAGTTGCTATCCAATCTTTG
GAGAAACCTGGGGACTGTTGCTAAATCATTGGATCCAAAAGAACTCTGTAAACTCCTGGA
GACATGTCAGAGCATGCAAAATGGATCAAATACTGGGACCTCTGAAGCAGCTAACGCTTT
GGTGAATTCTGCTGCAGTAGAGGCTGCAGGACCATCTAATTCTAAGGCGCCTTTTACGAA
TGGTGGTCAACGTGAGCAAACATCATCTGCTGTTATACCGCTACAGTCAAATGCTACCGT
GGTGGCGACTCCTGGTAAGGCATATAACCATTTTAACTCCCAGGAAATGTTTGCCCATGA
AAAAATCAGCTTATATATATTGTGTGTTTACAGAGACTCCAGCATGCAGGATTAGGAATT
TTGATTTAAATGACACTTGTAATGATATGGAAGGCTTTGAGGATGGTTCTAATTGCCCAT
CTGTACAGCAAGATTCTACTCAAAGCCCACCACAGACTAGTGGTAATTCAGATTCAACGT
CAGCTCAGTCATTGTCAAGCTCAAATGGAGATGCTCAGGTTTATTATTTGCTCCTTTCAA
CATGCTCGTACTTAAAAGCTAAAGGTCTAATTTCAAATTATTGTATGACGTATCAGTCTA
CATAGCAGCAACGTTATCCATTAATAACATTGTTTCTCAAAGGGAAACTAGCAACTCAAT
GCCTGCCAATTTGCTATTGATTGTTTTTTCAAATGTTCATTTCCCTCCAAAACATGTGTC
TACCTCTCTTCTCACGTACATTATTATTATTTTGCTTGCAGTGTCGGACTGATAAAATTG
TATTCAAGCTTTTTGACAAAGTTCCTAGTGATTTACCTCCAATTTTGCGATCACAGGTAA
ATATGCATTCTCTTAGCAAATGACAAGAGAGCGTATCTTGAATCTAACACTCATCCTGAA
TTTCAGATTCTTGGTTGGTTGTCCAGTAGCCCTACTGATATAGAGAGCTATATTAGACCT
GGCTGTATTATCCTGACAGTATATCTTCGATTAGTTGACTCTGCATGGAGAGAGGTACCT
CTTTTTCATAGTCATTTGATCATTTCATGTCATGATAGTGATCTTTTTTCTGGCTGGTGG
CTCATATTCCTTTTCTACAGCTCTCTGAGAATATGAGCTTATACCTGGATAAGCTTTTAA
GTAGTTCCACTGATAACTTTTGGGCATCTAGTTTGGTATTTGTGATGGTACGGCATCAAA
TTGTTTTTATGCACAATGGTGAGTCCAAGCGCTTATATATTTTTGCATAACTGTTGAGGT
TTTATTTATGCCTTATGTCAGAGGTCATATTATTGTAGGTCAAGTTATGTTGGACAGACC
ACTGGCACCTAATTCTCATCATTACTGCAAGGTTTTATGTGTTAGTCCAGTTGCTGCTCC
TTCTTCAGCGACAGTTAATTTCAGGGTGGAAGGCTTTAACCTAGTCAGTGCTTCCTCGAG
GTACATTATCATCTACAATTTACATGTGCTCATTCTTGCAGGGGATATGTTGTTCTCTAA
AAATACACGTCATGTTTGCAGGCTAATTTGTTCATTTGAAGGGCGATGCATATTCCAGGA
AGATACAGCTATTGTGGATGATGCTGCTGAGCATGAAGATATTGAGTGTCTCAACATTTG
TTGTTCCCTCCCTGGTTCAAGAGGAAGAGGATTCATAGAGGTACATATACAACTTGTTAG
ATACCTTTTGTTTTGATGTGGTTTTTGTTTTTAAATTGTAAATTTTAACTTCTTTCGGGA
CAAATAATTTTGAACATATTTTAATACATTGAATATCTTTGGCAATTTCAGGTTGAAGAT
AGTGGATTTAGTAATGGATTCTTCCCCTTCATAGTTGCTGAGCAGGATGTATGCTCGGAG
GTTTGTGAGCTGGAGAGCATATTTAAGTCATCCAGTCATGAACAGGCAGACAATGACAAT
GCCAGGAGTCAAGCTTTAGAGTTTCTAAATGAGCTGGGGTGGCTTCTTCATAGAGCAAAC
ATAATTTCTAAGCATGATAAAGTGGAGCTGCCTCTAGCTGCATTTAACCTGCTGAGATTT
AGAAACCTTGGTATATTCGCCATGGAGCGGGAATGGTGTGCTGTGACCAAAGTGCTGTTA
GATTTATTATTTGATGGATTTGTTGATGTTGGGTTGCAGTCACCGAAAGAGGTGGTATTA
TCAGAAAATTTGCTGCACACTGCTGTGCGAGGGAAATCTGTCCGAATGGTTAGATTTCTG
CTGAGATACAAGCCAAGTAAAGACCAGAAGGAAATTGCAGAGTCATACCTATTCCGACCT
GATGCTCGGGGCCCTTCTACATTTACACCCCTCCATATAGCAGCTGCTACTAGTGATGCA
GAGGATGTATTGGATGCACTGACTAGTGACCCTGGACTGGTAGGCCCTCATCTACTTATG
GAATATGATTCAAGTACTTACTTTTTTTGTTCTATGTTGCCTTCAAAGTTTCGTTGCTTT
GAATGTTACAGCCGTACACCAACGTCAACTTATTATTTAAGACTTGTTTTTATCTTTTAA
CTCTAGCACAGAGCGATACCAATTAAATGATGAACATCACGTTAATGATGCTATACCACG

GCATTACTCATCCTCTCAGCCTAGTTTGTTTCTGTTAATTAATTGTGAAGATCCGGTCTA
ATCTCCCTACTGTTGCTTCTCGTAGGTTGGACTCAACGCGTGGAAAAATGCGCGAGATGA
GACAGGCTTCACCCCTGAAGATTACGCTCGCCAGAGAGGCAATGATGCATACATGGATCT
GGTCCAGAAAAAGATTGATAAGAATCTTGGCGAAGGTCATGTTGTCCTTGGTGTTCCCAG
CAGCATGTGCCCTGTACTAACTGATGGGGCGAAGCCTGGTGATATTAGCCTCGAGATCTG
CAAAAGCATGCCAATGGCTCCACAACCTGTGTCGAGGTGCAACATCTGCAGTCGTCAGGC
TCGGATGTACCCCAGTTCCTTTGCAAATACTTTCCTGTACAGGCCAGCAATGTTCACCGT
GATGGGCGTTGCCGTGATCTGCGTCTGTGTCGGCATACTCCTCCATACCCTCCCCAAGGT
TTATGCAGCACCAAATTTCAGATGGGAACTGTTAGAGCGTGGACCAATGTGAGGACTTGG
GGTTGCAATAAAGCGTAGATGTGTTGCTGTTAAATTAAAAGAGTTTTAGTGTGGTGGCTT
GATCAAAGAAACATGTAACTATGTGAAGAAGAAGGCCCTTTGTATCTGAAAGGCTAAAGG
CAGGATTGACTCGCTGCTATAAGCCTTATATACCATCAATAATAATTGATTGTGCTTACA
TCCTAACATACATGTGGACAGAACATATGCCGCCATTCATTTCTCTTTCTGGTTGATAGG
CTGTACAATTCATTCTTTGAGAGCTGTATGTTTAGAAGAGAATCAGAATAAAGCATATTC
TTTTATTTTTGTTTGTAATATGTACATATGTTGAACCTTATTGTTCTCCCCCTGGAGTTC
CTAGGTGTTGAAAATCTGGATGGTGACTGTCTCCGACTGTTTAGACCCACATATCAGTCA
GTTAAAGCTTGCCACATACCTCGAGCTGATGTGGACACAAATGTCCTGTTTCCTGTTCTG
CGTGATGAGTTTCAATATTGATACTGAGCAATCATGCTAGAGCTTCTGCCATTTTTTGCT
GAAAGGCCATCACTTTTCGATCCAACCTATGTTCTATGGATGTTTTCTTTTT
>BRADI1G26720
TCGGTTTTGTCTAGATACACATATAACTACATAACGATGCGCATATATACATATACAAGT
ACTCCCTCCGTTCCTAAATACTTGTCGTGGTTTAGTGTAAAATTTAGGAACGGAGGGAGT
ATTATTTAGAGCCAAAATACAAGTATAGTACTATAGGACGTGAACCCTCGGATAGCCAAA
ACATTCGTTTAAAAAAAATGTAGAACTTCAATCGATCGTCTGATTGAAAAGAACAAGGTA
AAGCAGATGTGAGTACACGTTTCTGTACCCACGTACGTGGACGTTGGATTTGTCGGTTGC
CAGACGTATATTTCCGCTTAACACAAACATATACTAGGACAACACCTCTTGCATACGTAC
GTACGCGCGCACTCTGCCGTACGTATACAAGACCGTATTCTGCAGTGCCGCGCCCACATG
TGCACCCTGCCGCCTCCACACATACGGCACCCGCCTTTATATCCCTTTCGACTCTTTTCT
CCCAGACCCCCCTCTCCTTCTCCTTCCACACTTGTTGATCCACTCATGAGCTAGCTAGCT
AGCTCCATCGCTCCACCTCGCCGTCCGCTTCTCCTTCCACACTTCGTGTCGTCGGACGGC
ATGGACCGGAAGGACAAGTCCCGGAAGTCCTCCTCCTCGGCGGCGTCCATGGCCGCGCTC
GCCGCCGCCGGAGGAGACAGGATGGCGCCGCCTTCTTCCGGCGATGAGGACCAGAAGCCG
AACCTGGTGAACGTGCCCGTGGTCGCCACCGGCGCCTCGAGCTCCTCTGCTGCCGCGGTG
AGGAGGGGCGGCGGCGGCGGTGCTGCTGGTGGGCCCGTCGCGGTGGGCGGGGCCGGCGCG
GGCGGGCCCAGCTGCCAGGCCGAGAGGTGCCCCGCCGACCTCACCGAGGCGAAGCGGTAC
CACCGGAGGCACAAGGTGTGCGAGGCGCACGCCAAGGCCGCCGTCGTGCTCGTCGCCGGC
CTCCGCCAGCGCTTCTGCCAGCAATGCAGCCGGTAAGGCCGTCCATCATGCTCGCCCAAA
CCCTCTGGCTGTGGCTTTTCGCAGTCGCAGCACGCGGCCGTGGTTTGGAATTATTTGGCA
AATTCAAAACCCAAAACCACATCATCTTCTTTAGGATTAATTTGTCTAGTTCTTGGATCC
TGTGCCGCGCGTACGTCTGGCTCTTAACTTTGCAACAAAAGTTCTTGCTACACCTCGCGC
CAATTATTCCTGGATTACCGCTTAACTTGCGGTGCAAATTCTAAGGTTATGTGTTTACAC
GTGCACCGTTAGGTTCGCCTCTGATTTTGTTGGTGACCTCAGTAGTCTAATGCTGTAGCC
TGTGATAAGAGGCCAGGACAGGTCAGGTTAGCCGTAGAGATTGAGATTGTTGCACCCAAC
TCTACTCAAGAATTCAGTGCAAGTTACTGTTCTTGGGTGCTGTACATGTAAGCAACTCTG
TTTGCATTTCCTCCGGCAGGGTTGTAAAGTAAAACGTAGGATAAGGAAACCTAGCTAGGT
TTCACGTCTCATTCTGAGGATTAATTCCTGCTTTGTTAGTGCACGATAGATGGACAAAGA
CTACCATAGTGCACATCATTGTCCGTGGCTCCGTGGCTGTACACGTTCTTTAGGGGACAT
GCACTTTTAACCTGTGTCGGTGGCCGGAGATGATCAGAGCTTGGCCTTGCCTTGCCTTTT
TCTAAAACAGCTTGGTTCTGCTTCTCCAATTGCTGTCTTTGAATGTCTGATGCAAGATGT
AGCCCGTCCTAGATTAGTTCACCTGTGGATCGGTCAAAACTAGTATGGTTGCAGCATGTT

TTGTGATGCGTTATGTTTCTTTTGTGCATGCCATCTTGCTGTTCAGGAGTGCATATATAC
AGTACTGCAATTCTATGTTACAGTGTATAGATGTCATGTTGGCATTTTTCAGCATCAGAT
GTGCCATTCTAGCTAGTTTTTGGATTTCAACATCTCAGACTTTCAAGTTCACGAGCGAAG
GGGTTAATACTCTGTAGCTTTAAGTTAGTGTAGTGCAGCAGTAGTAGTAGCAGTAGTAGT
ACAGGGCTGGCCCTTTTCCAGAGCAGTTGGTCGCTGGCCCTGTTCATGCTGTCCTAGTGC
TGTTTAAGGTTTTGATGAAGACATTGCTACAGCAGTTCTTGATGACTCATAGGTGGGGAA
TTCAAGTGGAACAAACAATTTTTTTTTTGTGGTCCTTCGTTTCAGAATGCAATCATGTTC
TTCATGACCTCTGTAGCGGGAATGCATGGTAGTTTTATCCCAGCCTGCTGTCTGTATGAC
ATGACTTATCTTAGGTCCCTAAGCGCTTTGCTAAGAACATGTTTCTAGAGTATTTTCGGC
AAAAAGAATATGTAGGCATTGACATGTTTCTTTAACAACTCCATCTGGAAAATGATTTAT
CAAAGCAAACTAAATAGTGTTCTGGACAGTAGTGACAGTTCGTTCAGAACCAGACAAGAG
GGAGACAACGTTTCTGTTCTTCAGTTGCTTCATGTTAACCGATCAGTGCAGCGTTGTAAA
TTGCAGTGTTAAAACTTGGGAGATTAATTAATTTAGGGCGTGCAGGATGATTGCTCCTCC
ATGAGCTTTCTTTTTTTTTTACCTGTGGGTGGGGGGATCTTTATACGGACGGAATAGGAC
TAAATACGGCCTGATGGACTGTTGCTTTTTTCTGTCTCTGCTTTTGCATGCGCACATGCA
TTGTGCACAGCACCAGATTCCCAGCAGTACACATCTCCAAACAGCTTCCGATCCGTTGGT
TAATTCGTTTCAACTACACGCAAATTAAATACTGTACTCCAGTCTCCAGCTAAGTTATTG
CTTCGCTTCACGTGATGTATTTTTCATCTCTTGCTTCTTGTTTTACGGTGTTGGTTTCCC
TATTTGTTCTTGTCATCTCTGTCTGAAACTGCTGCTCCTGATCGACATGTACGTACAACG
TGCTGCAGGTTCCACGAACTTCTGGAGTTCGACGACACCAAGCGCAGCTGCCGCCGGCGC
TTGGCCGGTCACAACGAGCGCCGGAGGAAGAGCTCGGCGGACGCCAATGGTGGGGATGGC
TGCCGCCACGTGGACCAGGACGGCCGGAGCAACCCGGGGAACCCGCCGCCGCTGAACCAC
TTCCAGATCAGATAAACCTACCTAAGCATGCTCCCTCTCTTCTGTCATGCAGCCATGCTA
CATCTATTTATGTATCTATATTGGCCTATCTATCCAGTGTACTACTAACTGGCAGTATGT
ATGATCCCCTGTGCTGCTATATATTTTGCACAGGAGGTATATGTGCTAATATCTGCCAAA
AACCTAAGCTAATTGTAACCTGAAAATTAATCAGGTGTTAGAATGATTGTGCGTGCGTGT
GAGGTTTGATGTGTAATTGTATGGCCTGCAGTGCCCAACTAAGACTTTAGCAGGCTGTGT
AGTGTTGCTACTGTATTGGAATAATTGTGGCAATGTATGCTATTTATTCGAGTAATAAGT
GCAGAAGATCACTCATGCTTTTCAAGCCCTATCCTGTTTAGAATGATTCATGTACAAGTG
TCATAAAACCAGTTTAGTACGTTTATTTGAAAAAATATATCAGAAAATTTAAGTCCATCA
GCTTTTGCAGAAAATAAATAAATCAAAGCGTCCAATCATCTAACTCCAGAGGAAACATTA
GGATCACAGTTAGCGTTCAGTTAGGCAAATAGTTCGCTAGACAACCATATTCATCTTGAT
CATTCACGAGTTATAAGTACTTAATTGTAAAAAGGAGAAGAAGAAAGCCTGAATTTGTAC
ATGTCAACCACACATGCCAAGGGGTGAACTACTGATACGGAAAATATATGGAGCAGAATT
GTTTCCAAGCGCGACCTGCTTTGAAGCCATTGCAGGCGCGCGTGGCATAAGTAGCTAGGC
GAACAGCAAGTCGGCGTGTGGGCTGAGGTCCTTGTTGTGCACCTCCCTGATGGTCACCTC
GCTGACGCCGGTGGCCGCAGACACGTCCCGGATGGATTTCCCGGCGCCACCGCGTTGTAT
TGCCATGTAGATGACGGTGGCCGCGATGGAGTCAGGGTTGCGCCTCACGTCCA
>BRADI1G31390
AGATACACATCCAAATTTTGACAAATTTAAAACATCTTTCGTTGGACGGAGAAGTACCAG
TAAAACTAAGAGGCCGGTATAGCTAGGCAGGCTGGCACACGAGCCAACGTTGTGTCCACG
TATGCACATTGCACATATGTGTGTGTCTTGTGATCCGTGGGTTGGGGTGGCATATGTTTC
TCTGTCCAAAACAACCACGGCGACTTGTTGCACCGAAACAGCAAGCAGCTGCAGCCTAGC
AGGCCCCGGCCGGCCGGCCCACAGTCGCCTCCAGTCCAGTACTACTCTACTACTCTCTGT
GCGGTGTGGTGCTCGCTCAGGTCCAGTGAGGCAGCTGACACATGCCACTCCCGGACAAGC
TACACGCCAAGCCTCCATCCCAACGGACACGCCCACGCTCACTCCTCTCTCTCGTACTAC
CATCCTTCTCTCCCCACGCTGCCTTTTTAACCACCCCTGCAGCCTCCACTCCACTCCCCA
CTCCCCACTCTCACTCTCCTCCAGCCACTGAAGCTCCACACTGATCAACCCAGCTAGCTA
TCCACCCACACACACACACAGAGACCTTCTTCTAGCTCTCCTCCATTCTTTGCTTGCCAA
ATGATGAGCGGCAGGATGAACAACTCCACGGGGAGCGACGACTTCCCCTTCGCCCCAACG

CCGCCGCCATCCTACGGCGGCTTCGAGCAGCGAGCACTGTACGACAGCTTCGACTTCGCC
GCCGCCTTCCAGTTCCAGCACCAGCAAGAACACCACCAAATGCTCTCACTCCCCCCCAAC
GCCAACACAAGCAACCTACTCCACCACCCCATGGCTCCTCCCCCACCCCCAGCAGCCATG
TCAATGCAGCTCCCAATCCCCATGCCCCAAATGCACGGCCACGGCGGAGACGCCATGATT
TACCCAGCGCTGGGGATGGCCGTGAAGCGGGAGGGAGAAGTGGCCGAGGGAAGGAACATC
GGGCTGAACCTGGGCCGGCGGACCTACTTCTCCCCCGGGGACATGATGGCCGTGGACCGG
CTGCTGATGCGGTCCAGGCTGGGCGGGGTGTTCGGGCTGGGCTTCGGTGGGCCGGGGGGC
CATGGGCACCACCACCAGCCGCCCCGGTGCCAGGCGGAGGGATGCAAGGCGGATCTCTCC
GGCGCCAAGCACTACCACCGGCGCCACAAGGTCTGCGAGTACCACGCCAAGGCCTCCCTC
GTCTCCGCCGGCGGCAAGCACCAGCGCTTCTGCCAGCAATGCAGCAGGTACTACTGTACA
CAACAAAAACCCAAATCACCATCTTTCCTCTCTGTTGCAAACACTGTAGAGTTTCAGACA
TGGAGTTCTTTGCACCAGCCTAGAGCTAGCTTTAGCCTAGCTTTAGGTATAGCTAGCTCT
AGCTCTAGGTAGCTAGGCTAGAAGCGTAGGGGTAGGGCCAAGTGGAAGCTGTTTGGCTTT
TCAGTTACTCCACTGTCCACTCTCTCTACTGGGTTTCGTGTTGCTTTGTCCGTTGCGGCC
TCCTCGATCGAGCCATGGAAAGGCTGTGCACTTGTGTGTGTGCGTGCAAAAGCAAGAACA
CCAGTCAACGACCAGTCATGTCAAGAGGGAAAACGAAATGTGTATAGACAGACATCCACA
AAAGAACACTGCAATGCTAGCACTAGCACTAGCTAGGGCATGCACCCAGCTAGCTATAGC
AGAGCTTGCTTTCCATGGAAATCCATTGCCTTTTCCTTTCTTTCTTGCTTTCTCTCCGGC
CATGTGTGTTGTCTTCTCTCTTCTGGGGATCGATGGAGACAGACAGACGGACAGGGACAT
GTGTGTGTTGTGTGTGGTGGGAGAGAATCAGACTAGGAGGAAGGAAAGGGTAAAAGGCTG
CGTGCTTTTGCTTTGCTTTGGGACGCCGCGGCCGCTGCAGACTGTTTGCTCCTGCTGCTG
CTGGCCGGCCGCTGGGGGAGGCTGATCCGGTGTCAGGCTTTTGGCCCTGAGCATATCTCC
TTGCTGCTTTTCTTTTTCTTTCTCTCTTTATCCTCCTCCTCGCATGCATTGACCAATGTG
GTTGTGGTAGCAGCTGCAGCTGCAGCGAGACTAGAAAAGACTTTTTGATGCTGTCCTGTG
CCGTTGTTGTTTCTCACTAGACTTCAGTAGTACTGAACATTAGTTGTTGTGCTAGATAGT
ATTTTTCTTGTTAAGTACGTACGTGCACGGACGATTTGGTTTGGAATTCCTGAAGTTTAG
CTTAATTTTGTTGTGTATGTGTATATAGGTTCCACGTGCTCACTGAGTTCGACGAGGCCA
AGAGGAGCTGCCGGAAGCGGCTGGCAGAGCACAACCGTCGCCGGAGGAAGCCGGCGACGA
CCAATGGCACGGCGACATCGGCGGCCAAGGACTCGGCGACGCCACCTTCTTCCAAGAAAC
CCAACAACGGCGCCGGTGGTGCCATCATCGGTTCCTACACTGTCGACAACAAGAGTAAGA
CCTACACCTTATTTTGCTGGTATTTTGCTTGACTAGTTTGCAATGCATCCTAGCGCTAGA
TTCCTATGTAATGCATTTTATTCATCCTTATTGGTCATTTTCACATTAAAATTATGAACT
AATCTTGATTTGCGCATTTTCGTGTGATCGAGCAGCTTTGAGCGCCGCCAAGTCGTCGAC
CATCTCCTCCAACACCAGCGGCATCAGCTGCCTGCAGCAGCAGCAGCAGGACCAAAGCAA
GGCGGCGGCAGCGGCGGCGCTCACCCTCGGCGGATCGCCGCAGGATCAGAGCAACGCCGT
GCACCAGCTCGCCGGCCACGGCCACGGCCACCATCATCAGGAGCAGCACTTCATCACCTC
ACTCTTGCACAACAACAACAACAACAACAACGGCAACAACAACAACATCCTGTCGTGCTC
CTCGGTGTGCTCCAACGCAATGCCGCCGCCTGCGACAGCGAACAACGGCGGTGGCGAGGT
CTCCGACCAGAACAACCACCACGGCAACAACAACAGCAACAACATGCATCTGTTCGAGGT
GGACTTCATGTAGATCGATGCCACATCAACCAATCAAAGCTCTAGCTGCAGTCTGCAGAG
ATCGAAGACCATGCAAGCATGGGATCGAGTACTAGTCCTGTTTAATTGATTTTGTTTGTA
GGTAATTAAAAGAGAAACGAGAGAGACAGAGAGCAAAAGAAGGGTATAATCGTCCAAAGA
GTGTCAAGTGATCAGGTCTCTTCAGCTGCTGCTGCCCCCCAACTTCTTCAAGTGAGTGAA
AAGAAAAGGATAAAGGCTGTGCAGTGTGCTGCCTAGCTTAGCTTGCTTAGCTAAGCAATG
CAAGCCCCTCTTCCCTCTGACCACCCTGCAAGGCATAGCATGCTCCTGTACTGCAAAGCA
AGCATGCCATCCTTCCTGCCTTTCTGTTTCAGTGAGGAAATAATGTTTGTGCTCTTCTTT
TTATCTTCCTTTTCTTTACTTTTCTCTTTTGAAATTAGAGTTGAAGTATCTATGTCTGTC
CCAGTCCTTTGTTGAATCTCCTACAGTTAATTGTTTAACAGAGATAGCTCCAAGAATTAC
TCTACCATTTCATTTCACTGAATGCATGCCCCATGCAGGTGAGGTCCCAATTATTTGCAT
ATTTGTTTCCTTGATGAAAGAAGGTCCCAAAGATGATTCCATTAATGAAACCATGGCCTA

TACATAATAAGTTTTGGTCCCAACTTAGAATTACTCCAGGATCTTTTCGAATCTGCCACA
GCCTCTGTTTCTTTTTTTACTTTGACCATGGATGATGCAAGCTAGCAGCAGGAGGAGGAC
CAGTTAATCTCTGTTCTCCATTTTTACTGGCATATGCATCGCAATTAATTTGGAGGGAGA
TAGATAGAGATCTTGAGGCCATGCATATGAATGTGCAGGAAAATAATTACACATATTAAC
TGCATGTGAAAATATGGCAGCCCCACACGTACAGATCCTGCGTGCCCACCCGGATACATA
ACCTTAAATTCACAACAAGCCTAGCCCTATATACTGTTACTACCAGTACTATACGAACGT
ACATTACGCTAGCTCTTGGAGTTCATCATTAATTATTATTAGTAACTCGAGATCTGCTAA
TTGAATAATGGTTTATGTTGGAGATCTAGGTGCCTCAAGGTACTATACTACTCATGATA
>BRADI2G11240
CAGTAGGTTCATGGGAGATATCAGCAAATGGATTACCCATTTGTACCTATATATATGACA
TTTTGGATCGCTACGAGTGCTGAGTGCTGAACCTAATACTCCTTGAAACATGAACTCGAG
AATGAGATAGAAATTCTACTGAGTTTTCTTTAGAGAGACAGGGAAGCTCTACGGGAGATG
CTCTCCCGCGTCCGGCTCGCCGCAGCTATGCGCACACGTGAGAACCAACAGGAGAGCGCA
CATACCAAAAGACAAAGAAGTGTCCTGATCTTCCAAGGTCCAATAGCCGTGCCGTCACTG
TCTGAGGCGAAGCTGCCAAACGCCAGATAAATGGAGAAGAAAAAGAGGCTCTCATCTCAC
GATGTGAAAGTACATAGCCGGCTAGCGGCACGGCGAGGTCTTGCCTAGTAGTTGCCTTGC
CTTCCTTTCCCCCTCCCCTGGATACTTCGCCCCGGGTTGCCCCAGCCCTCACTCTCCACC
TCCTCTGTTCCAACTAACTCCCACTCCGCCGCCCCCCGTGTCTATGATCGAGGCACGGGA
GATGGTGAGACGGCTCCCTTACCCTGCTCAACTGTTGACTCGACTTACTCTCTCTTCCGT
GAGTACTTTCTCCTTCTCCTCTGTCCCCAATTCTTTTCTAACACATCTCTCCTTTAGCGC
GCTGTCGTGTTGATTCATGTACTATTTTCTTTTTCTTCAGTTCAAGATTGGTTCCAAGCT
AGAAAAGGGGGCTCGGTCCGTGTGTTGATCTGTGTGCGTTTGATCCTGCTGGGATGATGC
ATTTGTACTCGAGGATGTAGTTGAACTGCGCGGGCTTTAGATGTTTGTTGCAACAGTAGG
ATCAAGAACGGCTGAAAGGGAGGGGTTAAATGGAAGCCGGTTTCCGGCGAGAAGGCGGCC
AAACTGGTTTGGCTAATACAATGTCTAGTGAGCTCAGCAAGAACAAGAAGAAGAAGGGCC
TCGAGTGGGATTTGAACGACTGGAGATGGGATGGCAACCTGTTCCTGGCCACACCGTCGT
CGAATGCCGTCGCGGCGCCATCGGGTTGTGGCAGCAGGGAGCTGGGCCGATCTGAGGATG
GGATAGATTTGAGTGTTGCTGACAAGAGGAGGAGAGTTTCACCAGTGGATAACCATGGGG
AGTGCAGCAATGCTGCGATTGCAAATGGAGATCATGACAGGGTTGTTGGTCGGAGAGGGC
AGAGCAGCAGAGAAGGGAGACATGCAAATGCAACAGGTGCATATTCCACTTCTGCTCCAT
ATTGCCAAGTTGACGGCTGCCACGCGGATCTCCGTGACAGCAGGGACTACCATAAGAGGC
ACAAGGTGTGCGAAGTACATACCAAGTCCACTGTGGTTCGTATAAAAAGCATAGAGCATC
GGTTTTGTCAGCAGTGCAGCAGGTTAGTCAGCAATTTCTTTGCGTCAAGTTAACATTGCT
ATCATTGTTACTTGTGCCTTAAAAATTGCTTAATTTTGTACTTTTGTACTTGTCACATAC
TCATATGTTATCCTATTCAAAATGTCTTCATCCAGAGCTGTTCTTTGGCCACAGTTTCAT
TCTGTGTTTTTCTGATTATGTTACTCTCTCGTGAGCTACATCCTGGAATGCACCCTTTTG
CTGTGGGAGCAAAAAAAAGGGAAGCTATTGTTCTGAAATAAATTATGACCAACTGGATGT
GGATATACCTCGTGTTACTCCCTCCAGCGGCTCCACATAAACAAAAGGTGTTCTCTGTAA
ATTTGCACAGTCAACCAATTTTAACTTCTGTAGCATACATATCTGCTATATGGAGATGGT
ATGAATAGATTTTTCTCCTGATTACTATTTTATAAAATTTACCAATATGTTACAGTAAAA
ATTGGTGGCTGACATTGTGGTTCTTGACACCAAGTCGATCCCTAAGCCTTAACTTCTTTG
TGATTGAAGTAGTAATAATTACAGCTCACAGGATAAAGAATAAAACAATAAGGTAGTTGC
TTAAATGGTGGTTAAACTGATGGTTTCAATTATTAATTGAAATTTGTGGAGGGTCAGCCA
TGATCAATACTCTATCTAATTGACAATAAAGGCTCACCTGATCATGATGCATCACAGATT
ATCAGAAATAAGAACATTTCTATATTTCTATAGATAGGTAAATGATATGGCCTTGGGGGC
AACATTGGAAAACATAATACAGCTTGTTCATGTCTTGATTTTTTGTGGTTTAATGATCGA
TAGATCAGGCAAGAATTTTGGTGTAAGCACCAAACTGAACATATATGTGAAAAAAGGAAT
CATGAATATAACTGGAATTCAGATTTTTCACATGCTCAAAATAGTTTTTAATTTTTTGGC
ATATGTTGGCAGGTTTCATCTTCTTCCAGAATTTGATGAAGGGAAGAAGAGCTGTCGCTC
ACGTCTAGCAAAACATAATGGTAGGAGGAGGAAAGCCCCAGCCCAGGCTGGTGCGGCTGG

GAATACCTCAAGTGAAAATCAGTCTTTAACCAACACCTTACTCCTCTTGTTGAAACAACT
TTCAGGGCAAGATCGTAAGTTTTTAGAATTCTTTCTTTCTCTGATTTTTTTTCTACAATT
CTAAATACTGTGGTTTTATGAATCCATACGCAAACAAGCCATAGTCACGAACTCATGATA
CCTACATTATTTTGTTGTGGCAGCTGGAAGCTCATCTGAGCAAAGCAATGGTCCCAACTT
TTTGGTTAATCTTTTGAAGAACCTTGCTGCCATTGCTGGCACACAGGCGTATCAAGATAT
GCTTAAGAATGCAAATTCTGCTTCAATATCATCAAATGATGCTAACTACGTTGTAAATGG
CTTCACAAACGAGCCAACCAGATCACCAATTCCTGTAGGAACTGAATCCTCAGCAGGTAA
AGCTCTTGATTACCTGTTGCTGTTGCTCAAGGCAAGTAAAAGTAAGTAGACTCCTTTTAA
AAAAAATTGATGTGTACATGGTTTGGTATGTCTTGCCTGAAGAGCATACAGTGAAACAGC
ATGTGCAGAATTTTGATCTGAATGATGCTTACGTTGAAGAAGATGAGGTATATTATTTAG
GTTGATTCTTTTAGATTTCATGCTGATGTTAATTCTCTTTTAGCTACATTTTCCATGATG
CTGACGCCCATAGCATTTGCAGAGCCGAACAGATAAAATTGTCTTCAAGCTCTTTGGGAA
AGAACCAAAGGATTTTCCTGTTGATCTACGTGCTCAGGTCATTCGCCATATGCAAACTGG
AATTTTGTTTCTCTTAGAAACTTTATCTCCTTAAGTGTTTTTGATCAAATATTTTTTTAC
AGATGCTAAACTGGTTGTCACATTACCCAAGTGATATGGAAAGCCATATTAGGCCTGGTT
GTGTCATTCTAACTATTTACCTTCGTCTTCCTAATTGGATGTGGGATAAGGTAGTCTTCA
TTATGTGCTCTGGTTTTTAGATGTATATCTCTTATCTATGGTTTTTAGTGAATAAACAGG
ATATTAATGCAAACATAAAAATTCTAATTTATTAATCTTGGATGAAGAAACACACTGTTT
GCTGACTGCTATTTTTCTTTGTCTGAATCTGCCTAAAGCTTAAAGTCAATCCAGCTCCTT
GGATAGAAAACCTTATTAGCATATCCACTGATGGCTTCTGGGAAACAGGATGGTTGTATA
CTAGGTTACAGGACCGCCTGGCATTGAGTTGCAATGGTTTGTTCACCTTATTTTCTTTTG
TACATCCATGTTCCTTACGTATTTTATTTTCATATACCCAACAACCATATCTGATCTTTT
GCGGCCTCCATTATTTTGGCAGGTAGGCTTATGTTAGTGTCTCCCTGGCAACCCTTAATA
GGTGACAAGCATCAGATATTATGTGTAACTCCGATTGCAACTGCTTGTTCTTCAACAGCA
AACTTCTCAGTGAAAGGTTTCAACATAGTTCAACCAACCACAAAGTAGGTATCATGTAAA
ATTCTGCACTGACACGGATTTTGTTTATCTGACAATAATATTGGTATTGATTTCAGATTA
CTTTGTATATTCGGTGGGGAATATTTAATCCAAGAAGCGACACAAATGCTACATGAGGAT
ACTATGATGCAGCAAGGCCCTCAATGTCTGACCTTCTCTTGCTCCTTTCCTAGTATGAGT
GGAAGGGGGTTCATAGAGGTGCATCCTTTGAGTTGTTTCTTCGTGTTTGTTTTCATTTCC
CTTTTGACATTACTTATTTCTGGATTCCTTTTCCAGGTTGAAGATTATGACCAAAGCAGC
CTTTCCTTTCCCATTGTTGTCGCTGAAGAATCTTTATGTTCTGAGATTCGTATGTTGGAG
GATAAATTGAATTTAATTGCATTTGGTGATATTTTGGAAGGAAGAGAGGATCTGATGGCT
TCTCGTGACCAAGCCTTAAACTTTTTACAAGAAATAGGCTGGTTTCTTCAGAGGAGCCAC
AAACGAGCTACAACTGATGCTCCACAATACTGTACTGAGAGTTTTCCTGTTGCAAGATTT
AGATGGCTGCTATCTTTTGCGATTGATCAGGAATGGTGTTCTGTTGTGAAGAAGCTTCTG
GATACATTGTTCCAGGGTAATATTGATGTAGATGTCCGCTCACCATTTGAATTTGTCCTA
GGAGAAGGTTTAGTATTCACCGCGGTCAACAAGCGGGCGAAGCGTTTGGTTGAGTTCCTA
TTACGATACACAACAAATTCTGCACTTGTGGCCCGTGGAGCCGTGTCACCAGTTCGGTTC
TTGTTCACACCTGATATAACTGGTTCATCAAATATTACACCTCTTCATATTGCAGCCAGT
ATGAGTGATGGTGCTGGTGTTTTAGATGCTTTAACTGATGATCCTCAACAGGTACTTTGT
CTTGCCTCCCTTCTTCTAGTATCAAAATTACATCGAAAGCAGCTTGAATGTTTTTAAACC
CTAGTGGGCTAACATTGGTGCCAGAACCTTTTTTTTAATCTGAAATCATGACCATAGGAA
TTCATTTTGTAAATCTGGATAAAAAACCAGTAGGAGTTTTGTCAGAAAATAGTATGTGTG
TTAGTCAAAACCAAAATGTTAGGTACTCAAAACTATGGTTACTGTGTGGCTTCTGCCTGC
AATTTTTCTGCCATATTTTCTTTTCCTTAACGAACTCTGCCAAAATGTCACAGCTTGGAA
TCAAAGCATGGAAGCATGCTCGTGACACTACTGGGTACACTCCAGAGGATTATGCTCAGA
AGAGAGGTCACATATCCTACATCCAGATGGTCGAGAACAAAATCAACAGCAGGTTGCCTA
AAGCTCATGTGTCTGTTTCCATGACTATTAGTCCATCCACCACAGATATCTCTGAAAAGC
ACGCAGGTCGATCAAAGTCTACGAATCAAACCACATTGGATATTGAGAAAAGCCAAAGGA
GCGACAAGAGACCACCAAGCTGCAGACAGTGCGTCCAGCTCCAGCACATTGCTTACCATC

CCCGTCCGAACAGGTTTCTGTCGAACAGGCCTGCGGTGCTCTCCTTGGTCGCCATTGGTG
CAGTCTGTGTCTGCGTAGGATTGATCATGCAGAGCCCGCCGACTGTCGGCGTTGGCATGA
CGGGTCCTTTCCTCTGGAGTTCCCTGAATTACGGTCCCATCTGATAAAAGATTGCCAGGC
GCCGATCAATTATGCATGTCCATATGCTGAAGATGCTTGTGCTGGCATTGATCTGCATAT
TCTTACAAGCTGCAGACCTGTTAAAATGAAGTGCAATTTCTGTATATATTATATATACGG
GCAGTTGAATGCGAACGGCCTGGCTAGAAATTTCAATTGTATATGCTAACTTTTATTTGC
GGGTTGTGGCCCTTGTCTATCGTAGGCCTTTCTGTGATCGAGAGAAACAAGGTGACATAC
GTATTCTCATAGCTTGATGATCCCGCAGGAGTGGCCTGCAGGCAACAGCTCCCAAATACT
ATCTGCTGCAAACACTATCACAGAGTAGGGTACATGCTGAAGTTCTATAACATTCTCAAA
ACAATATTGCGTGTAGATGTGGCAATGCTCTAAATGCAGTTCGGAAAAAGAAAACAAGTT
TAGAACGTCACATTTCATTCCTACATCTCCCAGATGTGATTGAGTTTGTTATTCAAAATT
TGCATGTTTACAAAAAACCAACTCTTCAATATGTTGTTCTTGTCTAGATTTTATTTTTTT
TGGAAACTTGTAACCGTCATTTGAACACACAGTTTAAAACTGTA
>BRADI2G25580
GAAAACTCAATGCTGTGGGAAAAAAAATGTACTAGTAACGGAAGACTAACAAAAAATGCG
AGGCGGTGTCGACATGATGAGACCTATCGGCACATCCGCTCCCGTCCGTCCGCGTCCCAA
TCGCCAAGATATCTAATCGGACGGCTTGTAGCAACAAGATGGGGTCACCATGTGGTCAGC
CGCTCCCACTGACCTGATGGGCCTGCACCGGCGTCGCTGGGGCGTTAAGATTCGCCCACT
CGCCGAACACCGCACTGCGCGAGCACCTGCTCCGGTGCTCCCCGTCACCAACCCCGCACC
GGAATGAGGGCCTACGCGTCATAGAGTGCATCCCCCACCACCTAGGCACCTAGCAACTGG
TACTCGGTCTTCCTACGCCGCTCACCTACGAAGCTTTACCTCTCGACCCCTTCCCCCCAG
CGAGACGACCAATTCATTCGCCCTCGCCGGCTCGCCGGTCGCCTTCTCCCTCCCCTCGTC
TCATTCGCCTCAACCGTAAGAAAAAACCCCGCATCGCGGGAGCCGATCGCGCCACGGGCC
GGGGGTCTAGGGCTAGGGATAGGGCTGAAGGGGGGGGTGGTGGGGGTGGGTTCTCTGGGA
ATGGACGCCTCCGATTCAGGCGGGGCCAGCGCAGCGCCGGACGCCGGCGAGCCAGATTGG
GACTGGAACCACATCCTCGAATTCGCCGTCCGGGGAGACGACTCCCTAATCCTCCCGTGG
GATGACACCCTCGGCACCGCCGAGGCTGGTCCCGCCGAAGGGGCGTTTCTCCCTGCCCCG
TCTCCGGCTCTGCCAGTGGAGGCGGAGCCGGTGGCGCCGCCGCCACCTGTTGAGGCTGGA
GGAAGCAGGTCCGGCGTGAGGAAGCGGGACCCGCGGCTGGTGTGCCCGAACTACCTCGCC
GGGATTGTGCCGTGCGCGTGCCCCGAGTTGGACGAGATGGCGGCTGCTGCGGAGGCCGAG
GAAGTTGCCTCGGAGATGCTGGCTGGCCCGAGGAAGAAGTCGAGGCCTGCCAGCCGGGGC
AACGGAGTGGCAGCCGGAGGTGGTGGTGGTGGTAGTGGAGTAGCTGGCCGTGGGGGAGCA
GTAGAAATGAAGTGCCAAGTCCCAGGTTGCGAGGCAGATATTCGGGAGCTGAAAGGGTAC
CACAAGCGGCACCGGGTGTGTTTGCGTTGCGCGCACGCTACGGCTGTCATGCTCGATGGC
GTCCAGCAGCGCTACTGCCAGCAGTGCGGCAAGTAAGCCACAGTTTGCCCCTTTTTGCTT
GTAGAAGACCCACTGTGATCTTTATGATACATAATTAAATGCTTGGAACTTTTATACAAT
TGTCATTGCCTTGTGATTCTCTGTATTATATATAGTACTTCTATAAGACTCAGCATACTA
TTTCTAATTTCCATCATTCAAACTATTTCACTCCTTTGTCAATGACAGATACATTATCTT
TTAGCGTATTTCCTCGTAAATGTTGTTGCGGCTGGTTCGTAGGAATGTTATTTAATATTT
GGACTGTCATATTTAATTTTGGCTTCAGATCATCTTGAGAAGGTATTTTGCAACACAAGA
TCATAGGTGTAATGAAATAATTACGTGAAAAGCTACCAGCCTATAGCGCTAGATGTTTTT
GTTTCTTGCAGTAGGATATACCAGTACAGTGGCGTGCTATATTTGTTGTACAATCCCCTA
CATTGAAGTGGCAGGTTCAATTGTTGAAATGGTTTGATATGCCAACTATGGGTTATTTAT
TATTTTATTTGGACCTGGCGTCCAGGCATCCTGGTGTATCATCTAGGATTGGCTGGAATT
CACGTAAAACCTCCAGTAAATTACATCTCCATGTAGGGTCAGTATGTACGCTTCTTTATT
TTATTTATTTTTGTCATCATACGGTAGGAATGCATACGCACCACACGTGATCATATCCCC
AAGCACGCGGCCATAATTAATGGAGCCCAGGGATACATCCGCCATCTCACGATGTTCGCA
CTGAGCTTAAGTAGATGCATTAGGATTTGACCCCTGGTGGCTGGAGTCGTACACCCATAA
ATCCACCAAGAGGTGTTTCTGGAGTTAGTATGTTATACATGGTCATTGAACTTTGCATGC
AAATATCATTTCGTGATAATTCTAACGACAATCTATTTTATGGGTCTAGTTAGTTTCCTT

AAAAAGGCACAATTTGGAGTGGTCGTATATGATTTTGACATGACGATGTTGGGCCATTTA
TCAATCACACTGCAATTACTATTGTCCCTGTTCATGCATGCATAACCCTCAGCTGTTCAA
ATTCTTTGCCTTTGACATTTAACAATAGGTACTTCCCCAAACAATCTATTGTTGCCTATT
CTATGGCCAGTCTAACAATTGTGATGTATATGCTGCTCTTGTTGACTTGTAGTAATAGTT
TAGTTTGGTTGCTATAATAAGTTATCTCTCTCTCTCAATATATATTTCTGTTGCAGGTTC
CATGTTTTACTTGATTTTGATGAAGACAAAAGGAGTTGTAGAAGAAAGCTGGAGCGGCAC
AACAAAAGAAGACGAAGAAAACCTGATTCAAAAGGAGCATTTGAGAAAGAGGTAGATGAA
CAATTGGATTTGTCAGCAGATGGTAGCGGTGGCTGTGAACTAAGAGAAGGTAGGACAAAA
CGTCGTTGAACTTTCTAAGGCAACTATTTTGGCCTATTTAGTCATCTTAGCTTAAATATT
TTTTGTAGCTGCTCAGTTGCTAACGTTCCAAAATATTCTAAAGAGATTATTCGTGGCTGA
AAAGAAGTTGTCTCTTAAATAACTTACAAAGGACCAACAATTTCCCTTGATTTTTTTTAG
TCTGTTTGCTCTGGTAGAAGATTTTATGTTCAAGTAGAAAATACAAGTTTTAGTACTTTG
TCTTTAGGCATGAATTTATTGACCAGAATCATAAATATTTGGGAGCGCTTATATCTAATT
AAATGTAGGTTGGTAAATTTGATTTGTTTAGGATACATGTTACTGATAGTTTTGTTAGAG
TTGATATCTGAGTAATGTTGCGGATTTCGATATCAATGTTCTTAATGGTACTCAAAATCT
TCTAGCAGAAGACAAGAACATGCTACAAAATATAGAGCAAAGAAAAATCCCAATTATTGC
AAGCTTTAATGACAAGACAACACCATCTTAATCAATGTCCTAACAGAGGTTAATAGACCT
TAGACTGAAGTCCCATTCCCAGAGCGATGCACCAAAATAGAGATGGCTGGGGATACTGGC
TGTACAATCAGGAGCAAAACTAGGAAATACATTAAAATGCTCTATTTTGAAACATATATA
TTTAAAACCACAGTTTTGGAAAACCCCGGTGTTTTAATCTGCAGGCATGAAAACGTCCCA
ACATCATGATGTCAAAATCACACAGGCCTTTAGTTATAAATGTTTTTAAGGCTCTGTTTT
GATACTAATATTTTTGAAAGATAGTTTTGGAAAAGTCCGGTGATTTTGACATGACGATGG
GGGCTACTGTTACCGCATGTACCCACACAGGGCTTTAGTTACTCAGGGAGTCGGAATCTC
TACTTCCCTAAAGAGCTGATGCTACCCGACCCTCCTCTGGCAATGCTTACCTGAAAATCA
GCATATGCCTCCCTTTACCTCTGTAAGTTGCGGCTTCCGTGGGACCGTGGCAAACTAAAC
AGTCTCAAGACTCAAGAGGCATTCAGAATGTGTCACTGGTAGTTTATTGACACAGTGTTT
CTTTCATATTTGTGTCATCTTGGTTGACATAACAATAACATACAAGAGTGTCCTAGTTAT
AGATATGTCAATATTGTTCAAATTCCTTATCATGCAATTGCTTGGTTTGCAAAGGCAGCG
TATGTTCTGCTTAACACTTTGCAAGTTACTCTCATTTATCGTAACCCCTAACACTTGCTG
TCTTATTTAATAAATAAATAAACCGTGTTACAAATCATTTGTCAACAAATCAATGGAGGC
CGGCTTTTATCTGATGCCCTACTTGACTGAGGAAACCTTTTTTTTAATGGAAACGGGAAG
AGCCCACTAAAAATAGGTTAACATAAAAAGGTTCTGTTTACAGCCACCGATAAAAACATG
AATATAACAAGCTAAACATCATTTGTTTAGAGCATATCGGATCTTTGCGGAATTCTGGTT
TTGATTCGATTGAGTTCCAGTTGCATCAGTCTCTTCCAACTAGGCTTATTTGGTGGGATA
TTTTCTTAAATGTTGTTGTTGCTTTGTGTGGTCCAGTTCCCTTTCTTCTAATGCTCCTTT
TCCGTACACTATGTAAGTCTGTGCTGTATGATGCAGAACATCTTAATTATTATTTTTTGG
TATGCAGAGAATACAGATGGAACTACCTGCGAGATGGTTGAGACTGTCCTTAGTAATAAG
GTTTTGGACAGAGAAACACCTGTGGGGTCTGAAGATGTGCTAAGTGCTCCAACCTGTACA
CAGCCCAGCTTGCAAAATGAACAAAGTAAAAGCGTAGTGACTTTTGCAGCTTCTGTTGAA
GGCTGCCTTGGTACAGAACAGGAAAATGCCAATATTACCAATTCTTCGATGCATGACACC
AAGAGTGTTTATTCATCCTCGGTAAGTTCCTTTTTGTTCTTCCAAATCTCTCTCTCTCTC
TCTCTCTAATGGTTCGTATCACCATTCAGTGTCCCACAGGACGCATTTCATTCAAGTTGT
ATGACTGGAATCCCGCAGAATTTCCTCGACGTCTACGTAACCAAGTATGTAATTGGGTTA
GAGCCTATAAGTTTCTAACATCACATGACATGTCCTGCTCAAACAATTTATCCTAAGTCA
ATACTACACTTGACATATGCAGATATTTGAGTGGCTGTCTAGTATGCCGGTAGAATTGGA
GGGCTACATTCGTCCTGGATGTACAATTTTAACTGTGTTTATTGCAATGCCACAACATAT
GTGGGACCAGGTATGTATAGTTCTCCTAAAGCAAAGTCAGTACAGTCAATTCTTGCTACT
TTTGAGAGTGAGCACTTGAAAAGGAAATAAGCGGACTTGCTGCCAGGAAACTTCATTATA
TCATTACCTTTTGAACTTCTACAAACATATCATTTCTATGTGCATTCTTGTTGACAGTTA
TCAGAGGATGCAGCAAATCTTGTCAGAGACTTGGTAAATGCTCCGAGTAGTCTTCTGTTG

GGTAAAGGGGCTTTCTTCGTGCATGTCAATAACATGATATTTCAAGTATTGAAAGGTGTG
TTCCACACAACTTCTAATTCCCTGGCACATTGAGTTATATTTCTACATCACATTTGAACT
GTGTCGTTCCTACTTCCTACGCACCATATATAGTATGTGCAACACATCGATATCTATCAC
AGACTCACAGCCATAATTAGTGGTTAAAGTTCCAATTGTGCTTGCAAAAAGTGGCATTAT
TTGCATGTGTCACTACATGTAACATGTTGCAGAATCTTTGGATTATGCATCTGCTCAGGA
GAATTCTCATGACTAGATAACCCTTTGTTTGTCAGTTATTCTATTTGAGGATCCTTGTCC
TCTTCGACTGACACACATCGTTTTGGTCTGTACAATAATTATGTCATGTACAAACATTTG
AACAATTCTTAATTAACATGGGAACATTTGCCATTTAAGTGTATGGATTATTGCACCTTT
GTTTTGAACTGTTCATGAAAAATGTTACCTTACCATTAACTGAAATTTGTTATGTCTGTA
CTTAAGATGGGGCCACATTGATGAGTACCAGATTGGAGGTACAAGCCCCCAGGATCCATT
ATGTTCATCCAACATGGTTTGAAGCAGGAAAGCCTGTTGAGCTCCTCCTCTGTGGAAGTT
CCCTTGACCATCCCAAATTCAGGTATGACATGCCGTTGCATCTTCTGGTGATCTCTGGTT
GCTATTTTTCTAGGTTGTGGTACAAAAATTGAGAACATCTATAGCTCTAGCTTTGTGAAG
ACTGAAATTTGATCTTCATCATAGAAGTATAAAATCATGAGATTTGATCATCATTGTGAT
CAAAACTATTCTGTAGCTGTGTTTGGTCTCCCCAAAACCTTGTGTTTTATATTTTGAGTT
TTTTAGTTGGCCAAGTTTTTGAATGACCAGATATTAGCCAACTTCTATCGACTTGGAGAA
ATAACTAAATGAATGTAAGTGGTGGCAAGTGAAAGCCAGTATTTTTTTTATCTCGTTTGG
TCATAGTTCAGGCACAAACTGTGTAGACATAACTAGTAATTTCGGCATTAAGTATCTCTA
TGGGCCAAGCAAAAATAAGTTATTTAGCTGTTCTTTTCATTAATAACTCTATTGAACTTG
TGACACTTTATTAAGCTGAAGGTGGAGCTTAGGGGCTCCCTGTTGCTGTCGCTTTGTTCC
ATTGTATCACCGGGCTGAGCCCTTCTTTTCTTGTTCCTCTGGGCTGTGCCCTTGTAAATA
CTTGGACATAGTTTTTATATGTCTAATAGATTTATTCTGTCGGAGCCTCTCCCGTAGTTT
TTCCCTAAAATATAGCACTTTTTAACCCGGGATCTCTCCCCTTTCCATTACTTGCATAAC
GGAAATACAAATTGTCTTAACGATAAACAGAATGGGGGACGAAAGATTAAGGATGAGAAA
AAACCCAGGAACCAACTACAGACAGGAAACCCACTGACCAGGAGCTGCAATCAGCTCCTG
AGACTGGGACGAAAACCTGCCTCTAACCTAAACCTATCAGAGTTCAAGCGTTTACAGCAT
TTCAAAATAGATGACAGCGGCGCCAAAAGCATAACACAAGCAACACCAGCAACCAGCAAA
AGTGCAGAAGGTCCTTCAGGCATCAGTGAGACGGGCGCTCCCCATGGTGTTGACCGGCAC
AGAGACAGACATAGCGCCCTCCCCCATGCGACGCATCAACTCCTGAGCGTTGGCCACCAG
CTTCTTAGCACCTTCCTTCAGCAGGTCAACATCCGAAAGCTTCTGAAGACCTGCCCAAGA
TAACAGAGAAGCACAGGCAGCAAAGATCACTTCAAACGGAGACCTGAGCAAATATTTGTC
AAAAGTCACTTTATTCCTGCAATTCCGAATTGCCCATATGATAGAAGCAAGACCAACCAC
ATAGAATTTGTTGCGTCCAGGTAAGAAGGCATTCATCCACACAAAGAATTGCCATTAAGA
GGAAGGCACCAGTGTAGTACCAAACAGAGAGCCAACCACTCTCCAGACCACTTTAACAAC
CACACAATTAAAGAATACTCCCTCCGTCCCATAATTCTTGTCGAAATATTACATGTATCT
AGACGCTTTTTAGGAATAGATGCATCCATTTTTGGGCAAATTTGAGACAAGAATTATGGC
ACAGAGGGAGTAGATTATTAGTACTGTGGTATTGCAAGTGCATTTTCTTGTAGTCAAGAA
AAAACTTGCAAATTTAACAGAAAACAGATGGAGTTGAAGCATTTTATGTTGGCTTTGTAA
TACACTGGTGGATGGGTGGTTATACTTTATGCACCTTATGTGCTCATTAATGGTGTTTTG
TTTTCCTAATTCTAACCTTCAGTTTGCGCACCATGGCAGTTGATTGGTAGTGTGCATGAA
GTGAATCCCCATGGAACTGTTCTTGTAGATAAGCTATTTATTAGATGGCAGCTGTTTATG
CTTTTTTAGGATTTTCAATGCTCGATGGATGGCTGATTTGCATTCCATTTATGTTCTTAT
GGCAGATCACTTTTGTCATTTGATGGGGAGTACTTGAAGCATGATTGTTGCCGTTTAACG
TCTCACGAGACCATTGCTTGCGTAAAAAATGCCGCTGCACTTGATTCTCAACATGAAATT
TTCCGGATAAACATCACTCAAACAAAGGCGGATACTCATGGACCTGGGTTTGTGGAAGTA
AGTTAATCTGCCTTTAACAAAACCAATTAATTTTCCTCCATACTTTAGAGCCTGGTGCAT
ATTAATCGGTACCATTTTTTTCTGGAATCCACATGTTTGGCATTATATTGGTAATGATGC
CTTGACAGTCCTATGCACTCCCTCCATTCCTAAATATAATACTCCCTCCGATCCATAGTA
GTTGTCGAAATATTACATGTATCTAGACGCTTTTTACGCATAGATATGTCCATATTTGGA
AAAATTTGAGACAATTAATATGGATCGGAGGGAGTATGTTCTAGCTTTGTCCTGGTACAT

TTTTCTATAAACTTGGTCAAACTCAAAGAAGTTTGACTTACAACAAAGTTAGAACATCTT
ATATTTAGGAGTGGAGGTAGTAGATGGTTGTGTTGTGCACAAAATTATGTTATGCTTGTA
CATCCTTATTAGCCAAGATCAACCTGAAGGATTGTATGAAATTGATAGAACCTGCTTATA
GGGCTTAAGCTTACGCCATCCCGAGCAGCGTGCTTTTGAAGTGGTTCCTTAAATTAGGCA
CCATTACCCAAGACTTATGCCCTCTACTGTACACAGGGATTTGTGTGCACCCACATTGTT
TTTTGAACATCATAGACATTGGATATGTCAGTTATAACATGTAAGGTTGCTAAATTCTGT
GCAAAAATATGATCTTATCTGCACTTTGCATAATGACAAACAAAACAGACTTGTATCACA
TAATATGTGGATTACAAATACTAGATGGTCGAGCTATAAGGAAAATAAGTGAGAAATGGG
GATCTTTTTTGCATTTCTCCTATGATAAAGACACGGTAAACCATTACCCTATCACACGGA
ATACCACATAAGGCATTATTATATTTTGGCTGAATTTCAGCAGAGATGGGATATGCTCTT
TCGACATAATGATATACTGGCTAGTGACCTACTATTGTTATGGCACTAACAGTATAGGTG
TATTAAGACCACATTGTAGAATAACACCTTGAAATAGCAGAATCAAATTCATGCATTTTA
GAGTACTTGCAACGTTTGGGGGTTTGAATTGTGTACTGTTTTAAGAACAACAATTACTAC
TCCCTCCGTCCCATATTAAGTGACTTTCTATTACATGTATCTAGACGCCTTTTAGGCATA
GATACATCCATATTTGGGCAAATTTGAGTCACTTAATATGGGACGGAGGGAGTATTTATC
TAACTGCATGCCTGTTCAGATTAAGTTGTCATTCTTCGGCGCTTCTGTAGCTGTATATGA
TGATAGTTGATCAGTTCTATATGTTCACTTTTGTGATTTATTTCTTAACAGGTGGAAAAC
ATGATTGGGCTATCAAATTTTGTCCCTGTCCTTTTCGGTAGCAAACAGTTGTGTTCTGAA
CTAGAGAGGATACAAGATGCTCTATGCGGTTCTAATGAAAAGTACAAGAGTGTATTTGGA
GAGGTTCCTGGTGCCACTTCTGACCTGTGCGGGCGTCTGGAACTTAAACAAACTGCAATG
TCGGGATTCCTAATAGAGATTGGATGGCTAATTAGGAAGTCTTCTCCAGATGAACTAAAA
AATTTACTGAGTTCAGCAAATATCAAGAGATGGACATCTGTGTTGAAGTTCTTGATACAG
AACGATTTTATCAATGTTCTGGAAATAATTGTCAAGTCTTCAGACAACATCATAGGCTCT
GAGATTCTTTCTAACTTGGAAAGAGGGAGGTTAGAACATCATGTCACGACATTTCTTGGA
TATGTACGGCATGCTCGAAACATTGTTGAAGACAGAGCTAAATATGACAAGCAAACACAG
CTTGAAACAAGGTGGTGTGGTGATAGTGCTTCAAACCAGCCAAACTTGGGCACTTCTGTC
CCATTCGCTAAAGAAGTAAGTGCTATTATGTTACAAGCTAATATTGTAGTGATCATAATT
AAAATAATCGTTAGGTGCTTGAAAAGCCTTCAATAGGGGCATAGCTTTTCAGAATTGAAA
TCTCTGAATTAGGAAATACTAGACACTTCGCTGAACCTTGCAAACATTTCTTTATTGCAC
CGGTTTCATTCTCATGCCCTGGACACCAGGCTGGTCTCCATCCTGCTGCTGGTACATGCA
AGTCTAGTCGTGAGCCTCGTTCACAGCTCTGGAGTGCTCGATTTTGGTTTTCAAGAGAAT
GTAATCATGACTTAGCAGTAGGCTCTAAAATACCATTTGCATGCAAATCATAGTCAATAC
TCAATAGTATCACAGAGAATTGGGGGAAATCATGGTACCGACGACATTGAGTTAAGATAT
ATTTAGGTGTAATGTGTTCTGTTTTGTTTTATTTTAGCTTCTGTTTGTCTATGCTGGTTG
TTGAAAAAGATGACCATTGAACGAACTTAATGCTGCACCTAAAATAGAATTCAAGTCGCT
CAACCATTTGCAGTACCCATGTCAAATCATTTCTATGAATTATTGCATCAACATGAGCTT
GTGTCGGTCTAGAACTGTTGTTGAGTGGATCCTAGGAGTTTCACGGTAGAGGATCTTTTG
CTTGATGAAAGTGTAAGCAGCTTAGTAGCCTTAGTATTGCATTTATCCCTTTACCTATTG
GAGTGGCACATTGTTCAGTCTTTTTGAATCGAACTAAACTACCATGATTCCCTAAAAAAA
ACTAAACTACCATGATCTTTTGTAATATTCTGCAGAATACTGGTGATGGCAGTGAATATG
ATTTGCACCCAACCAATGTTGAGTGTAAAGAGGAAGAAAGGATGCTACTTGTGAGTCCTA
AAGCTGTCTCACACAGGCAATGCTGCAGCCCGGAGATGAATGCTAGATGGCTTAATCCCA
CCTTGGGTGCACCTTTTCCAGGTGGTGCCATGAGAACGCGACTTGTCAAGACTGTGGTAG
TGGCTGCTGTATTGTGCTTCGCTGCTTGTGTTGTCGTTTTCCACCCAGATAGAGTAGGGG
TGCTTGCAGCGCCAGTAAAGAGGTTCTTATTTAGCGACTCCCCATCTAGTTGAACTTAAG
GATAGATTAATTTTGTTTCTATTGCTTTCCTCTGTACCATATCTGCACAATTTGGTTGAG
GGTGTGCACCTATGATCGAATACCTGTTCCACCAAGTGGGCAATGTAAACTTGTATCCTT
TTGTTAGAAACTAGCGTACATGTTCATTCCTGAATAAATGGAGCGGCAAAGATTGGGCTT
CAATTTTTTTGTTCAGTCTCCTGCAGCTGGGCTATTTAGCCTGATATATAATGGGAAGCT
TGGAGGACAAACTGATCTGAATGCAACTTGAACATCTATCTGAATGGCCTGAAGATGCAT

TTGAAAAATACTCGGCATCTTACACTATCAGGTCTGAACGAATACCGATTCTGTCTGTAG
CATCATCTCAGATAGACGATCATTATATCCTTCGCCGCAAGGCATCGAGCTACTTTGTTC
ACATGTGCCTCGTTGCTTGTATCGAGACTCGTTAAGATCTCCAGGCAGTTGCTGACTTGC
TGTGATCCGGTTTGAAGCATAGATGTAACTATGCAAGGGAAGTGTATTCCACGTGATCCT
CTCAAACCAGGCAGCAAAGCACCCACGTAATCAAATCATATACGGAGTAGGAGTACATGG
AAGAACAAATGCATGATAGAAACTGATGTGAAGTGTACTTTATGCTGTCACTGGGTGACA
CTAGGCAATACTAGTCGATAACAGGAATTGTGAAGTGTTGACTGAACAAAAGTGTCGACC
GGAAATGGGCAGTACAGGCGTCGAGGACTTCTGCAATACACATGGTGGCAAAGAAATGCT
GTAGTTGGTGATTTGTTGGTAGTGACACTTAACGACAAGTTAACAACCATCGATAATATA
GGTCCAGCTGGTCAGCACACTCG
>BRADI2G59110
TAAATAGTATATGGATGCATCCATTTCATGATGTGGAACTATACAGAGGTTCGTTTTAAA
AGTGCAAGATGCAATAATCATGAGGAAAAGAGTGGTTGTGATTTGTGAATATCATGTCAG
CTGAAATGAGGCAGCAATGCTCCACGCACACAAATACAAAGTACAAACACAGAAAGCCTC
CGGCAGGCGCGCAGCAGCATCTCTCCTCCATTCACAGTCTCCATGCATCCATCCCTTTCC
AAACTTTTGTACTTTTGCCCTGCCTCCATCCCCCTACAAAAGGTGAACGAAAGGGAGGAC
AAAGCACTGCGCTTGGTCCTTCTCTACATTACAGGCGTTGGTAATTAAAGTGAGTGAACA
GTAGATGAAGCAGTGGAGGAGGAGGTGAGGTGCCCCCCTCTCGGCAATTATTGCCACCTT
TGGAGTTTGGAGTCCTCCAATAGGAGTCTCCTCCTGTGTTGTCTGCAAGGACGCTATCTT
TCTCTCTCCCTCTTCCTTCCTTCTCTTCCCCTCTCTGCCCATCTGCTCTCTCCTCCAGCT
CTGCTCTCTCAGCCTTTTCGAGGTTGGTGCTCTTCTTCACTCTTCACACACCGCTTCTCA
TCATCGCTTTAAAGCCTAAAACCACTGCAGCGAGCTACTCTGTTCTGGTGGGTGTCGTGG
TGGGTTTGGTCCGGTTCCTTGGATTCTTAATTGCATCCTGTTGCTCGCTCCTGTGATCTC
TCTCTCCGTTCTGATAGCTAGCTACATCTCACTCCTTTTTCACTGTCCTGCTTAAGCTTT
TGCTTGCAATCAACTCTTCTTTCTGGGGTGTCTGTACTTGATCTCTTCCTTCTGCTTCTT
CTTCGCTGAGTAGAGAGATCAGGAAAGGTTGAGCCGTTGTTGTCTCAGGCGACGGCTACG
CGCTGCAAGGAAGCCTTTTTAAGCCACACCATTCTCGCTGATCACCACAAGCTTAGCTCC
CCAGCAGCAAGCAAGAAAGCAAGAAATCTACCATGGATTGGGAGGCCAAGATGCCTTCAT
GGGACCTGGGCACGGTGGTGGGGCCAAGCGGCGGCGGGGGCGGGGGGCTGGACCTAAAGC
TCGGGGGCCCGACGAGCTGGAGGCCGGTCCCGGCGGCGACAGCGGCGCCGGTGGCGCCGT
CGTCTTCTCCGACGCCGGCGAAGCGGGCGCGGGCGGGGGCGCCGGCGCCGGCGTGCTCGG
TGGAGGGCTGCGCCGCCGACCTGTCGCGGTGCCGCGAGTACCACCGGCGGCACAAGGTGT
GCGAGGCGCACTCCAAGACCCCCGTCGTCTCCGTCGCCGGCCAGCAGCAGCGCTTCTGCC
AGCAGTGCAGCAGGTATACAATTCCATCCGTATTTGAATCCGTCCTCTCCTCTTTAACAT
TTTTTTGCAAATTCAGACAGTAGCTTTACGCATGCAGCTTCTGTTCTCTTCTCCTGCCGT
TTGACATTTCAAACTTGAGAAGAAGAATATATCAACCGTACTGCTAGATTGGCGTAGTAA
AAAGCACATCCGTGTGTGTGAGTTAGTGAGTACGAGTAGTAGCGGTGTGCGACGACGGTC
GTTTGGCGAAACAACAAGAGCAATTCGCTGTCAGATTGGAGCAGTTCCCTGTCCCTGCGC
CATCATGGCCGCGCCCGTCCGTTAATTTGGCGCTTGCACTTAACTGCACCAATCGATCGA
TCGATTGATTAACCGACGTGCCCCCACACTAAACTCGCGACTACTACTGCGACCCATCTG
CATCTGCTAGTGCTTTCCCACTCATACCGTCAAAAATTGCCACGAGTGATTAAACTAACC
AACCCGCCGTAAAAGCTTGGTAAGCGTAAACCAGTTCGATAATAATCTCCACCTGTCCAA
AAGTCGTTTTTTTTTGCTGGAGCTAGTCCAAAAATCGTTTTCTGATGACGCAGAAAAAGG
TTTCAGTTTGATTTAGCAAAACACACTTCGAAACGCGTGACGAGGAGCAGTCAAATTTAC
CTGGCTGGCTGGCTGGTCCTGAGCGGCTCAAAGGTCTGAACCAGACACGCAGCCAGCAGC
AGGGTTTGACGCGTTGCGCCGCTCCCCCGACCAGACCCGACAGGGACGCCGCGCAGCCAC
CGGAACCAGGCCTTTCCGGGTCGCCGTCGGTGCCTCTCCTTCAGTCCTCGTCTCACGCCT
GCCTGCTACTTCGCTCTCCATCGGCCATCGGAGTATTACTTAAACTTACTGTTGGTCTTT
GGCGCCACGTGACTTCATGTTACCGTCAGGCAGTGGGTACATTTTTTCTGCCTGTTACTT
TCAAAATCGGGGTTACTGTTGATTCTTTTTGTTTTTCCGATACGTCGTGCTCGGCCGGCT

CTGGCCCCTGTCGCATGTTTTCCATGCGCTCCGGCCTCCGGGTCGTCGTCGTCTCCCAGG
CCGGGGAGAAAAGTCGTTCCGATTCTCCTCCCCGTGTGTGGCCTGCGTGCGTGCTGCTTG
TTCTGGAAGACAAGGATCGAGTTGCTTCTTGGAGGAGGATCTTCTGACCGGTCAACGGTG
TGATCTGCGGCCGTGGCCTAGAAAAGGCAAAAAAAAAAAAACTGTGATCTGGCCATGTGAT
TTGAGATTGTGCCGTGATTAAGCTCATGTTCGTCTTCCTGTTAGGCGTGCCAAAAGACTG
GATGAAAACTGCCTGACAGGAAACTCTGTGTTGTTTGTTTTCAGGTTCCATCTGCTCGTT
GAGTTCGACGAGGTGAAGAGGAGCTGCAGGAAGCGGCTCGACGGCCACAACAGGCGCCGG
AGGAAGCAGCAGCCGGACCCCCTCGGCCCTGCCGGCCTCTTCGCTAATCACCACGGTAAA
ACCATCCATCCCTCTCTCTGTGCTGCTAATGTTTTTTACGCCGCATGAAACATAAGCTGA
GATTTTGGCGTTCCCAGTTTGAGTGAGACAGACAAGAGGGAAATTTGGGGACGCATTTGG
TCGATCATGTGCTCACGTTTTTTTAATTTTTAGCAGGACTTTGTACTAGTCCTTTTCCCC
TTTCTGTTGACTGACAGTACTATCCATGGGAACACTTCACACGTGATTGATGGAAAGCAA
AACGAAAGTTCCTTTCCGAAATGTTTTGAGAAAAGGATGTTTGACTCCTACAAGTCCTGA
ACTATCATTATGAAGCATGTCCGCATAATAAGCTGTAAAAGCCAATCTCTTTGATCTGGC
ATTTCCAGGAACTTTGGCCGGCAAGAGAAGATGTCTGCCTGAAATTTCTTCTGAACTTAT
CTGAAACTGCTGTACCTATGTGAAACCAGTTAACGCTGCCACAATGAATTGAACATGCAA
TTATTGGTCTAGCAATAAATATTAGTAACATCAAATAACAAGAAAAACATCCAACTCATG
CCTTACGAAGACCTTACCTGAAAACAGCTGATCTTTAGCTGCAATCAAGTAACAGGGTAG
GGGTAGATGCACTTTAAACTCCTCTGCCAAACAAATTTACAGTTCTTTTTTTCCTTCTGA
AAGATGAATTTGCAGCTTGTCTATGTAGAAATTTCGTATCAACTGGACGACATGCCCAAC
TGTAATGCAGGTTTGCATCAATAAAACATCGAACTCACATTCTCCGCTTTCCATCGTTTT
CAATCAGGAGGAGGAACAAGATTTGTGTCGTACCCGCAAATCTTCTCCACGACGACATCC
ATGGCGCCGGAGCACAAGTGGCCTGGCAGCATCGCCGTCAAGACGGAAGCCGACGCGTTC
CAGGTCGAGCAGTACTACTCGTCACTCCAACTCAACGGTGCCGCCAACGCCGCCGCCTCG
CTCTTCCATGGCAAGGAGAGATCGAAGCGCTTCCCCTTCCTGTCCGATCACCATGGCGAC
ACGGGATTCCAGCAGCAGCCATTTACCATCACACCTTCCTCAGAGAGCAGCAGCAACAGC
AGCAGCAGGCACAGCAACGGCAACAAGATGTTTGCCACCAACGACGGGGGCCCGGATCAC
AACTGTGCTCTCTCTCTTCTGTCAGACAGCCCGACGACACAGGCGGCGCACACCATGATC
ACCGCAGCGGCGCAGCACCTCGGTGGCGCAAGGATACATTACAACGTTGGAGGCGATGGT
GGCGGCGGTGTCTCGCTCACTGGGCTGTCTTACGCCAGTGCCATCATGGGAGACAACAAG
GGCAGCGCACAGGCCGCCATGGCCTCATCTACCAGGCACGCTGGAGCCGTTACTACAGCC
CCCTCTGTTGCTGCCCAGCTACAGCAATACCATGGCTACTACCAGCACCAGGTGAATGGT
GATCAGGGGAACTCAGCTGATGCTGGTGCCTCCATGCAGGCCCTCCCTTTCTCATCATGG
TAGAGAGGCTCGATCCTGAACACTGCTAGCAATATACTATTTGCACGGCGTAACACTTTC
GTCACTGATCAGATGATCAGATCCTTCTGTAGTTCTGCTCCCTCATCAAATATGTCTCTG
ATAATAGTAGCCCTGAATGGTTGATCGATGAGTATGAAAACATCACCAAGATATGTTCGT
TTTCCAATACTTGTCTTGATACTTCTAACTTGGAAGAAAATGCCCCTACTTATGATTCAG
AATTGGATTGAGATTCTACTGCTCTTCGTGTTGGTTCGTTTCTTCGTCAGTTTGGGCATG
CACTTGCGTGGCAAGAGTACGTCAGCAGAAGTACGTAAACTGAGCTGTACTGAACAGTCC
GGTTAGCTGAACAAGATGCAGGTCATTGATTATTGTAATCATGTGGGGCTGCATGTTTTT
CACATTCTGTGCATCAGCAGAGATTTGGTTGTCGAACAATGATTATATTAAGGATTTTGG
ACCACTTCCGTGACCGTTGTTCTACCTATAGGAACCCAAGTTCAGAATTCAGATAGCTAC
ATCCTACTCTGGAATTGTTCCTTTCTGCCCCCTTGCCTTAAAAAATAAGGTGGTGTGTCC
TTGTTTTCTGTCCATCATCTTGGACTGAAGAAATTTGTCGTTCGGCAAAATGACGTGGAA
AGAATTGCCTGTTAATGTTATGGGAAACGTGGAACAAAATAAGCCAGGCGCAACAGGCAG
AATCAGATGCATGTGTCGAAGATGACTAGACAAGGGAGATTA
>BRADI3G03510
GAAACAAAGTTGGCCATCTTGTGTATGTGCGTGTGTGCTGGATTATTGTGAGTCTTGTCT
TTCTTATCTATGTTGCTCTCCTTTCTTGTCTTTTGGATTTTGAGGCCCTCCCTCCTCATC
TTCCTCCCCAGTGCCTGCCAGGCAGAAGGATTCCAAGGAGACAAAGATTCTTCAACAAAA

GGGTGGTACTAGTAAGATTCTTGATTTTTTTTCCCTTTCTCATACAATACAGTAGAGGGG
TAGGAGCACTTGTTCTTTTCTTTCCCCTCCTCTAGATGGGTAAAAAAAAGGGTTCTTCTG
TGCCCCCACAGAAAAGCAACTGACTTGATGTGGTATAGGTTCTCTACCTCCCTGTCCCTC
TGATCTCAAAAAGGATTTAATTTGCATGTGCTGCTGCCACTATTTGCAGGTGCTGGCATG
GAAGAAATAATGCAATCTTGAGGTAGTTTTCTTGGTGGTGGTGAAAGTTTTGGATGGAGA
AAGTGAAACTGGAGGGGTGGTTGCCATGATGTGTAGTAGGGTCCAGCACTGGAGTGGTTG
TTGATGCTGTGGGTTTCAACGCGAGGGGGATAGGGGGGGAAGGGGAACACACAGGTTATA
GATCTCATCAAGCAAGCCAGGTATGAGATTTTAGGAGGGGAAGCAGCGGCCAAGCTTGTG
CTATGTGCTTTAGCTGAGGCTGATTCTCTGGTTGCTGCCTGCCAATTAATTGATGAAGGT
ACCGCCGCCGCCACCGGTCACCGGATCAAGGAAGCTTCATGGTTCTCTGCCGTGCTGCTG
ATTGAGGAGGTGCTCCAGGCTTCCAGGAGGAGGAAGAATCTGCAGCGTTTCCAATATTTC
TGGGAGAGAAGGAAGGGGAAGATGGTTGATCTTTGGTCCCTCAAGGTACTTTCTTCTGTT
TTTTTGTACTTCTTGCTGCTGGAAATGGAATGATGAATTGATGATGGTGGTGCTGTTCTT
GCCATGATTCATGATTCTCCTAGTAAAGGTAGGGAGACTAGTCTGCGTTCTTTCTGTTCT
TTGGTACATGGCATTCATTCATTTGATTCTAGCATCTTGTGTACTTCTCTCTTGCTTCAA
TGATGTATAGGTGAGTGCTCATACGTGTTCTAAATCTGTTCTCGTTGACTTTTGGAAGAA
CAAGTTCTGTCCTAATTTTGCCTTCCATTTTGTTGCTTCAATTGCAGCAGCTTTGACATA
AAAGTAGGAGCAGTACCTGAGATTTTCAATTCTGGCATGGGCTCATTTGGGATGGACTGG
AATCAGAAGAGCTCAGTATTGTGGGATTGGGAGAATTTACTGCCGGCAGGCACTAATGCG
GCTGAGAACCCCAAGAATGGAATGCAGGCTGAGCAAAGGTTTGCAAGTGTAGTGGCTGCC
ATGGCTAATGAATCACGACATTCTTCCGGTAGCAGCGGTACTTTCTCTTCCAACTCGGAG
ATGGGATATGGTTCATCCAAGAGCTCCCTATCCGCTTCAATTGATTCTTCATCCAAGGTA
GGGAACAGCATGGAATTCAGATTTGCAGCTGCCGCAAACCCTGATAGCAACAACAGCAAA
AATACTGAGTTGGGTAAAGTTGATGACACCGGAGCCGGAACATCTCCCTCATCGGTGATA
GCAGTGAGCAGTGGAGAGCCAGTCATTGGCCTGAAACTTGGCAAGAGAACTTACTTTGAA
GATGCCTGCGGAGCACAGAATGTCAAGAACTCACCATTGGGTGCGGGTGCACCAAACCCA
TCTCCTGCTTCGGTCAAGAAGGCAAAGGTGGATCAACAGAAGCCACATAATTCATACTGT
CAAGTTGAAGGCTGCAGAGTTGATCTCTCTTCTGCTAAAGATTACCATCGAAAGCACAGA
GTCTGTGAAGTTCATTCTAAGACTCCCAAAGTTGTCGTCGCTGGTCTGGAGCGACGCTTT
TGCCAACAGTGTAGCAGGTGAGATGCTTTAACTTGTACATGAAGCAGCACCTGTTTCTAT
GAACCAAAGTACCTGTTTGTTTTCTTGAACTCATTATCTTGGCATCCTTGAGTGAGGAAC
AGTGATTCTTTCTGATCTTTTTCTTTTTCTTCTTTGATACTCCACTGGTTTCTAGGTTTC
ATGCTTTAGCTGAGTTTGACCAGATAAAGCGAAGCTGCCGTAGGCGTCTCAACGATCATA
ATCACCGCAGACGGAAGCCACAGCCAGAAGCAATTTCTTTCAGTACATCAAGGCTGTCTA
CGATGTTTTATGGTAGCAGCCTATGTTTCATGGAATTGATGCATTAAGTAGGATTGTTAC
ATCTGCCCTGCTTAAAATTATGCTTTTCCGTACATGCTTAGACTAACTCAGGACAAAGAG
CGTTATTTTTCCCTGACATAGTTGATTCTGTTTATTCAAATCTCATCATTAGTCATAATA
TTTTCAATTTCATGAATTTTAATAATAGTATTATTGTAAGGACTCATTTATATTACTTGA
AACATATTTCAACTTTAGCTGTTATTATGTTATCTTGTCGTGGTTTAAATCTTTAATGTT
TCAGAAAGGAAAAACAAACTGCATAATTGTACAAACATTGATTGATTTGTTTGTGCAATT
ATGCAGTTTGTTTTTCCATAAATGTTTATGTGAGTAACAGTAGGTCGAATTTCCATAAAT
GCATGTTGAGTCCACTATAATATACTAATCAAGATATTTGTTCTTTCCTGAAAATGTTTT
AACTTTAGTGCTCAAATTGTTGTAATGATCCTGGACAGATGCAAGGCAACAGACAAGTCT
TCTATTCAGTGAGGCTCCGTATGTTCAAATGAGAAACTGTGCAAGTTCTTCATGGGATGA
CCCAGGACCAGGAGGCTTCAAGTTCACAGAAACAAAAGCTCCTTGGTTAAAGCCAACAAC
TGCTGCAGGTGTTGATGCGATGCATTTGTCTAGCCAGCAGGTATCGAACAGTATTATGCC
ACACGGTGCACATCATGGTTTTGATGGGTTCATGTCATTCAAGGGAACTGGTATGAAGTT
CCTTAATCAAGGTATTTCCCTGTCTAACTTACAAAATGAACTTCCGAACGAAGTGAAACA
ATAATTATACATAGGGTCATCAGTTGCATGAAAACTGATCTTTCTACTATTCCTAACTAG
CTAGTAGTAGGAAACAAGCATACTTATAATGCTTCTTTCAAGTTATTTACTATCATCATT

TGGAGATATTCAGAAATGAACCACAATTGGCTACTAACATAATTGACATGAAGCCTTTTG
CACTTCCTGGACATATATGCATAAATGCATGGTACTTATTTTTGCAATCTGAAGAGTTGT
TACCATCCTCTTAGCACTAACCTGATGCTGATATCTACCAGCAATTGTGACTAATACCAT
TTGAGCCCATTAAGCTGTGGGGTCCAGAAAAGAATAGTGATGGGTTACCTTATGTCATAA
TCTGGCATGATTGAGCTGCCTAGAAACAGTTGTTCATAGTGTTCTATTTTTGTTTTTTTC
ATGTGGTTTTCTTTTTCTATAATGTTAGACATGGAGGCAAGGTTTGGAGGGAAAAAAGGT
AATCTGGTATAGAATCATCATTACTTATAGTAGAACCAGACTGAACGATCAACTGAAAGT
TTTTTGCACATTTGCACCCTATTCATAGCTACCAGAAAAATAGATGATTTTCTTAAAAGC
ACTGGGACAATTGTTGCAGATGCGGTGATTATAGCCTTCTGAAGTATGTTTCATTAGCAA
TGAACATTAAAACTAAAAGCTGTTCTACTTTTACCTCTTTTCAGGCGTCGAAGCTTCCAC
TGTTGTTTCCGACACCAGTGGAGCCCCAGACCTTCAGCATGCTCTCTCTCTTCTGTCAAA
CAACCCAGCAGGTGCTGGCAATCTCCAGCCAAGTCCCCAGATGCATTCTGGAACTACTGC
AGACATCGCCGGCACTTCAAACCCTGCGATGCATGTGCTGGGCTCGTCGCCAGGGCTCTG
GCTAGACGGCCCGCCCCTCGACAATCATCACCCACGGTTTCAGGCTTTTGATCTCTTGGG
GGGCCACGACAGCACCATGCCGCACGAGCTCCAGCTCCCAAAGCCTTCATATGACCACCA
CGCCGCCTCCCACTTCGACCGGATGCACTGATAATGATAGACGCTCCGATCAGCACCCAC
CGTGCGTGATATCCTGAATTTCTTTGTTTTGATGTTGTTGCCAACCTGCTTAGAAGGGAT
GCAATCTGGTAATGCCAAGAATAAAGGCGCACATGAACAACAGACTTGCAACCAGATTGA
TCTGGTCGTGTTGCCATGGAAAAAAGAACTTCATCTTGCCAGCAGCTACCAATCCCATTA
TCAGCTGTGGCTCTGAATAATTCTAAATTGTTATGTATCTGAATTGCTTCTGCAAGTGAT
GAATCCAGAAGTTGGCAGAACTGCAGACGATTTTAATATTTTGCAAGATCTTGGCGGCTT
GGAGTCAAGGAGGAAAAGCAGTAGATCCTTTTTGTTTTTGTTTGCAAGAGAGACCCTATT
ACCACGCTGGTTAAGACACTGGTTAACATCTAAAAACATGACACTGGCAGGTATGACTAT
TTAAGACTACAGATTAGCACATATGTGGTTAACGACTGCTGCATTTCCAAAACATAATTA
TTTTTCTTCTTATTTTCGAAAAAGGGAACTTTATTAATTCTCAACATCATATCAAGCAAT
GCGCCAGCATAAGCCTCTGCATAGCAAAGATGCACTCATGCACATAGGCAAACAAGAAAG
AACAAACGCACTCAAGCACATAGATACATATAAGAACATTTCGCTCATCAGTAATTTATT
TATTTATTTTACCCTAAAAATGAACAATGGTACTACCATCTTTTAGATAAAGGACCAATC
GTGCCCGGTTTTATACATAAAGCTAAAACCGGCTGCACTTAACCTGCCTTGAGAAGGTAG
GTACCTCTGTAAATTTAACTTCACTTCTGACATGGAACTTGACAAGGATCAGCTAGTATT
GCCCTTCAGGGATTTGGTGTCCAACAAGACTCGGCAGCGGTACTCTCCAAGAGCCCAAAC
AGGAAAGATGTTCCTGTACTGGGAGTAGCTGATCGTGCAGTTTCTGCTGAAGACTCCCAT
TATCTCCTGTGTTACAAAAGGAAAAACCGTTTTTAACTGATAGGAAAACAGAACAGCAAC
TGAATGAAAGTTGCCTGGTCAATCATGTCTTACTTGCTGAGGGAACTCGCCATCCACTAA
TTGCAGGTTAATCAAGAC
>BRADI3G05510
ATTCGACTTCAACACATTCATATTTGTATACGTACTATCCGTTTATCCGTGACTGACTCT
GAGTCCAAAAAAAAGTTATGAAGTCTGTTTTCAAACCTACAATGATGCCAGAGAAAAACC
AAGTAAAATAATGAAAATACATGACATATATATCGTCTTCCCACAAGAGGCCAAACTTTG
GAATTTAGCGGATTATTTTTATATAGCATGTGTTTATGAAAAAAAAGAAAATAATCATAC
TCCACCTATCATCATTATAATAAGCTAGAGTTAAGTAGATAAAGCCTCTTAAATAATAGT
GTAAAAAAGAGCCTCTTGAATAATTTCTCAAGTTTCACAAACTCAACTGCTTTGCAGCTC
TCCACTTCTACTGTTCTAATGAAACCCAAACAGCAAAAAAGAAGATGGAAGTAAAAAACG
AGAAAGGAAAAAAACGAGGGAGAAAGGGACCCAAGACTAAGAAAGACTTGCCTTTTTCTA
TTCCAACTCTCTCTTCCCCCATCTCTGCTTCTCCTTCTTCTCCTGCACAGTCCACACTGT
TCCTCTGCTCCTCCTCTCGATCCCCAAATCCCAAGCTGACCAACCGAGCCCGCGCCCCGC
ATGGAGTGGACGGCCCCGAAGCCCGCCACGTCCCCCCCGACCCACCTCCTCTGGGACTGG
GGCGACTCCGCCGCGCCGGGCTCCTCCGGCGACGCGGCGGGGAGGCGCGGGAAGGAGAAG
CGGGCGAAGGGGGAGGAGGGCGGCGGCGGCGGCGGAGGAGGAGGAGGAGCGGTGGTGAGG
TGCCAGGTGGAGGGCTGCGGGGTGGAGCTCCGCGCCGCCAAGGATTACCACCGGAAGCAC

CGCGTCTGCGAGGCCCACACCAAGTGCCCCCGCGTCGTCGTCGCCGGCCAGGAGCGCCGC
TTCTGCCAGCAGTGCAGCCGGTGGGTGTTTCCTTGATGCTGCGAGATTGGCCGCGGGAGC
TGTGTTATTGTGCTCTCGGTGCTCCTGCTCGTCGGACCTTGTGCTTTTCTTGCCTTCGAT
TGCTCTAAAGTCTAAACTGCTCGCAACTTTCTGCGATTGCTTTTGCGCGACTTTGTGTGT
GCCTTAATTCCGGAAGCGCAAAAGTTCTGCGAGTTCGTGTCTACCGGCAAATTTCTTGCG
CTTTCTTAGGCCAGGATGCATCGCGCTATGCTTTAATTGCGTGATTAGTTAGTCGATTAA
TCCGTGCAAGCTTGTGTTTAATTTGATGTGCTTATTGTTAGTGGGGTTTGGAAATCCTGG
TGCTTTAGCCTTGAGATTATTTTGGGCGAGGGAGTACGGCTTGTTACCAGATTTGGGAAG
AGAAGATGCCTTTTGCACCCACGATTAGTGCAATCTAGTGTCTACCTGTGGAAAATCGTA
CTGCATGGGTGAATGGCTGCGCCATGCATGTTTCCAGTTTCGGCATGTCTGGCCCCTGTC
CTCTATATCGTGCATCTTTGGGAGGAAATTCTCTTGTGCTGTGGCTGTTGTGATTGAATT
ACTGTGTGGAGATCTGTCTTCCGTGCATACTTTTAGATGATTGTTAGTTTGTTACTTTGT
TAGCCTATACAAATAACTGGCTCTGGTTTTGCGGATTGATTGGGTTTTGGTGATCAAGGT
TCCATGCCCTGTCGGAGTTTGATGAGAAGAAGAGGAGCTGCCGGAGGCGACTGTCTGATC
ACAACGCTCGACGACGAAAGCAGCAGCCAGATGCATTCTCCTTCGCACCTGCAAGGCTGC
CATCAACGTTGATATTTGGTAATTTATCCATTAAGTCGTTAATTATGCAAGATTTGAATT
TTCCTTACTCGCAATTTGATTGTCATTGCTCATTACAGATGATAGACGACAAATAAGTTT
TGTCTGGAATAAAGGTCCACCTAGCCATGTAAGGCCTTTCGCATGTTCTCCATGGGAAAG
CCCATCTGAATTCAAGCTCTCACAAGTGAAGAACACGAGAGGAGTGTCAACCAATGGACA
AGTTCATCTGGACAAATCTCATCTATCGAATGCTGTTCCAACACTGAGTCATGACATGGA
TGAGCTGTTCCCAGTAAAAGGTATGATTTCTCAAACATTTTGTAAACTACCATCGATGAA
GAAGATGCTATATGATTTCTTGGTGGCTTAACACATTTATATTTATTAAAAAGAAGTGTT
TTAATCTTTGAAATGTTACTTACGTGGTGTTTGAGATGCCAAACTCTGGCCCTACTCTGG
AAAGTGTAACTGGCATGCTAATATCTCATATATTAACTCATATAGAATAAGTTATGGCTA
ATCATAGCATTATCAAAAGCCGGAGCTCATGCATACTTCAGCATACTGCCAACTGACCAA
CCATAAAAGAGCAGTAATCCCAGTGCTATTTAGCTGTAAAAATGTTGTTTCAGTTCACTC
ACTCGAACCAGTGAGGGAGATACTTCTTTATCTTCAATATTCTCGACTAATTCTTCAACA
TATGGGAATCTTACAGTGAAAGACTTACTTGTAACTCCTGCTCATGGCTGTTTATAGTTT
CCGATAGGATTTTATTATCCTTAATAGAACATTAGGATTGGATAACCATGATTCCATGGC
TTATTTCATATTGGTCTTCTATGCATTAATTGCAGAAATTCTTCTCTTGAAATGAGTCCC
TAGAAATATATTTTATCATTGCTAGATGTATTTGTTGATCTTAGATACAAACACATGATG
CCACTAGGCTTATGAATTTGAGTACATTCATTGTAGATAACGAAATGGAGTGTTATATGA
AAATAAAAAACAAGGAGGTTTTATTCAAGATTTGTACTCCCTCCGATCCATAGTAAGTGT
CTCAGATTCTGTACTAACTTAGTACAAAGTTGTACTAAGCTTGAGACACTTATTATGGAT
CGAAGGGAGTATCAAAATATGTGCTATGTGGAGTTGTGCTGCCTCAATAATGATATACAC
TAATTTTTCCTACTCCTGACACATAAACTTCAGTCTGCTATACTTGGATATGTACATATG
AGAGCTTTATCAAGATGCTTACAACATGACTAAGCAGTACCATGAATATTACATTAGAAC
AACTTGCACCTGCATTGGACTAATTTGGTTTTTAGTATAGCTGATATATACTGTTTCATT
GGTAGATAGCAGCCCTGGCATCTGAAGTAGTAATTATTGTTTCTATTTACCAAAAGTTGA
CACAATAGTGGTCTGAACATCACAAATTTGAAGCTACAATCAATATTGATATTGTGGCTA
CACAGCTAGGTAACGCGTACAAAGTGTTTTTAGATTTAAATGTTTGTTCCATTTAACAAG
ATGATGTCAATGCAACACTCAAGATAATGCACAGACGACAGCTTAGGAACTCCCAAATTC
ACTTTGGGATGTCTAATGTCCAAACTCCTGAATAAAGAATAACAGAGTATTGAGTCTCAT
GCTCCAACCAATTAATCCCAAAGCTTAAGCTGATGAGGCAAGGTGGGCAATGTACTCATC
CTTCAACCTGGAGGCTAGTTACATACTGCGTAAATGCTTGCCAGCAATATCTGCTCAATT
GTTCTTGTCATGTATCTCAAATGCATATGAGCCACAAGATTTTTTTGTTCAGCGTTCTAG
ACATCTCTGCTACCAATATTTGGATGTTCTTGGGTGTCCATACATTGATGAGGGAATCCT
TTGATGGACTGATTGATTGATTCTGCAGGTCCCGATGCATCTTTAGCTGCTTCAAAATTA
GATGGAGCACCGGATCTTCAGCATGCTCTCTCTCTTCTGTCAGCTAGTTCTCGTGGATTA
CCTGATCCTGCACAGCAAGCATCTTGTCTTGTCCAATTCTCCGGTGCCAGCCAAAACAGC

CGGGGCCTTCATTCATCACATGGAGTGAGCTCTGCGTCGGCACCCTGTGCTGAAGGACAG
CCTATGGCGCCGTCGCCTCACCTCGTCCGTTTTACCGTGGATGGCACCAGCAGTGGCTAT
GACACCACATTCTTCGGTCTAAACAAGATAAATTAAGGCGTGAAATGTACCGGCCTTTTT
GGCTATTTTGGATCAATAGCCAAGAGCTTTTGCAAGAACTGCTTCTCTCGACGGTGTAAA
ATCTCACTGTTAATGTTACCTAGCTACATCAGCATTAATCTGTTTTTGTACAACCCTGCT
GTCAACTGTTGGCTCTTCGAAACCATCGGATGAATTTGGACATGATTTTGTTTCACAAAT
TGTGCCTTATATATTTTTCTTTTCTTCTCCCTGTCAAGCCATTACACCTGTAGTTTCCTC
GCATTTTAAAAATAAAGTGCAGTGTCCCTCGCAATTCCATGGCTACATTCTACGCATCAT
CAGTTTTAATAGAAGTACGAAAGTATATTGTGTATGTTATAATCACGGCTAAAGTTTAGA
ACGCAAGACGACTAGTCGATTAATGTTAGCATCGAACTAATTGATTGTTTGAGGTGAAAT
CAGCAATGCATTTTGGAACTAAAATTCAGATTACCTGCCATATGGTGGCCTACACCTGAG
ATGACTGATCATATGTGATGTATATTTTACACAAAGTTGAAGTTCACATGTTGAATTGGT
CAATCGGTTGTTTGCACTGATAAGTCATGGGTATGCCAGGTTATATTAGTAGGTGATTTC
ATGATAATAAATCATAATACTAGATTAAATTTATTAGATTACTCTATCAATACTTGATAA
AAGTTTATATCGCGATATGTTTTTTTGACTTTGTAACGATATTTGATTAGTAAGTGTTTG
GTTCACCATCAGTCATGCATGATGTTAGTAGCGATAACTAAATTTTAAGATCAC
>BRADI3G05720
GGTCTGGAAGGATAAAAATATGAGGCCCTCAGAAGTGTGACATAAAGAGTCGTGAACTAA
AAATGGGAACAACACATGTAATATTGACCATTGTATAGAATATACCTGTGCATAAGCATT
GCCCTTGTCATATTCCTTGACACCATAATTGTAGGTCAACTCCAATACAGCATTTTTGTC
TTCGGGGCCATATCCCATCATAGCAATGGTATACTAAAGAAAGGTAAATTAGAATTTTAA
ATAAAACCCTGAAAGTTTACATGGAAGGGATGATGAATACGCATATTTGGTTGCAAAATA
TTTAAGAGGGAAAATATTTCAGACACAGAGCTGGAAGCTTCTACCAAAGTATAAATAACC
TATATGATACATGTTTGTTATAACTGTAACCAAATGAAAACAATACCGGGTGCCATGAAC
TACAACCCTATGGTGTCACATATATGACTATCTTTAACTTTGCTTTTTTTCTTACTTTCT
GAACATTTNNNNNNNNNNNNNNNNNNNNNNNNNNNNNNNNNNNNNNNNNNNNNNNNNNNN
NNNNNNNNNNNNNNNNNNNNNNNNNNNNNNNNNNNNNNNNNNNNNNNNNCCGATCGAATG
ATGAGCAGCAGGCAGCAGCTGAGCCCCGGCACTGGTACAATGCCACCCATCTCCGGCGAC
GCTGACTTCGGCAGCTGCTACACCAGTACTCATCATCCCTACGCCGCCTTCGACGGCAGT
CCCAGCGCCGCCGTCGACCACCGCCCGCCGCTGCTCCACCACCACCACCAGCAGCTCTAC
GACACCACCGGCCTCGACTACGCCGCATTATTCCCCTTCGCCCCCCAGCAAGATAATAAC
CCCCCCGCTCATCTCTTCCCCAACCAGCTGCCGCCCTTCACCGCCAACAGCACCACGATG
CTTCTCCAGCCGCCGATGCTGACGCCACTCCCCGGCCTGCCGACGTCATCCCCGCCGCCG
GCCCCCGGGGACGCGTACCAGCTGCACCACCCCTTCGGCGGCTTCCAGCTGAAGCGGGAG
AACGAGGGCGGCCTCTTCCCCTTTTCCGACGCCATGGCCGCCTCCGGCGTTAGCGGCGTT
GGCGGAGGCAGCGGAGGGAGGATCGGGCTCAACCTGGGCCGCAGGACCTACTTCTCGCCG
GCGGACGTGCTGGCCGTGGACCGGCTGCTGATGCGGACACGCGGGGGACTGGGCGGCGGC
GGCATGGGGGTTCTGGGGCTGGGGCTGGGAGGCGGCATTATGCAGCAGCCGCCGCGGTGC
CAGGCGGAAGGGTGCAAGGCGGACCTGTCGGCGGCCAAGCACTACCACCGCCGCCACAAG
GTCTGCGAGTACCACGCCAAGGCCGCCGCCGTCGCCGCCAACGGCAAGCAGCAGCGATTC
TGCCAGCAATGCAGCCGGTACGCCCTCCTGTATGCGTAATTTCGAAAATAAATTCCCCAG
TTCGTATATACAAATTGAACAAGGATGCGTGCAGGCATTCCAAGTAAGTAGTTCATCTAG
CTAGCAGACGAAAGAATCGTTGGGGAAAGGCTGCGAGCTTTCGATCTGGCTAGCTAGTAC
ACATATGGAGTATCTATCGATCAATCCGCATCTGCATATGCCAGCTGCTTCAACACCTCA
TTATCGATCCCTAGCTAGCTAGCTGCTGCATGCCTACATTGACCGATGTAGCACACACCC
ATCATTGCGTGCATGCATTACTCCATCTCTACACACAGACATATACCTGGCATATGTATG
CTATACGTACATTAGTATCTAGTACATGCATGTGACATGTATGTCTCGCCGGGAATCGTA
TGAATAATTTCGTTATGGCCGGCCTTATGGGTCGTTTGTGTTTTGCGGGCTAGGTTTCAC
GTGCTTGCGGAGTTTGACGAGGCCAAGAGGAGCTGCCGGAAGCGGCTCACGGAGCACAAC
CGCCGCCGCCGGAAGCCCGTCGGTGTTCAGGGCAAGGACTCGCCCCCGCCGCCGCCTTCC

AAGAAATTAGAAGCCGGCATCACCACCAGCTCATACGCCGGGGATCACACTAGTAAGTCC
ACTCAGCTCTCATACATTTCTAGCTACCTTCAAGATCCAAACGTTGGATAAACTGTGATA
TTCGGAACAAAAAACTCAAGCCGATCTCGACTAATTCAAATTTGTGCCAGTAAGCAGTCG
CATGAGAAAATAAATCAGAAATGTATGTTAGGGGTCTACGAGAATGTTAATGATATATAC
CATGTAGTTAAACACTACAAAAAAAAGATAAAGTACATGATCGAGCAAGAGGCAATTTAA
TTCTATGCGCAAATTAATTTAGCTTCATAAGTGTGCCTCCGTCCCAAAATAAGTGTCGCA
ATTTTGTTTATACACGTTTCACGGCTTAGATTTGCTCATTTTAAAAATAAATCATGACAT
TCATTCCGGGAAGGAGGAAGTACTATTTGACACCGGTAATTCTATGCCCAAAAACGTTTA
CGATGTTGGAGTATACCTAGCTAGAGAAAAAAAAACTGGCAGTACAATACATGTATTTGA
CACACGACAGAGCTAGCTAGATGCTATGTCTGCATTCTGGTCGATCGATTGATTTGACGT
ACGTACCTAGCTACGTGCCAGCTCAATTAACATGATGCGTATGCATGCATGGCGCCAAAA
AACATTTCACACGTACAATCTGTCCTATGCTGTCTATACGCACCAGATCAACTCCTGCTA
CATACATGTGGCCGTCACATCTGCCATCTTGTACTCCACTCTCTCGCCGTCTTTTCCCAG
TAGTTCTAACTTCTATCATGTAATGGATCGATCTGCGCAGCCACCAACAAGTCATCATCG
ACGGCGGCAGCGGCGGTACTCTCGCCGAGCGCCAGCGCCTTCAGCTGTCTGCAGCAGCAG
GAGGAGGAGCTGGACGACAACAACGAAGGCAGCGGCGGGCGACCGACGACGCTGTCGCTC
GCGGCGCCGCCGCAGAGGGACTACGGCGGCGCCCTGGACACCATGCTTCTGATGCATCAT
CATCATCATCAGGCAGCAGCGGCCGTCGTGCAAGACGACGACCAGGAGCAGGACTTCATG
ATGACCTCTCTCGTGCAATCCCATCATCATCATCAGCAGCAGCAGCAGCAAGGCGAAGTC
AGTGGCAACATCCTGTCGTGCTCGCCGACGGCGTCGGATCAGCGTCGTCAGAATCACCAG
CAGATGCAGATCAACGACGGCGGCGACGACAGCGGCTGCTGCAATAACAGCGGCATGCAG
CAGCAGCAGCATTTCTTCGAGGTGGACTTCATGTAGTATAGCCTGCCTGCCTGATGAATG
AATGAATGAACCGGCCGGGGCCTGGCCGGGGGCCTAGCCTAGCTGTAGCTAGAGGTAGTT
TGACTCCATTATCGTGAGGTATGTGTCAAAGGGGTGTGTGCGTGTGTGTGTGTGTG
AGTGCATGAAGTGAAAGAAAGGATTCCACGAGCTGCATGCAGGGGTAGCTAGCTAGCTGG
CAGGCTGCTGCTGTGTGTTAGTGCGTGGCTAGCTAGGGTAGGGCGTGGCGGCTAGCATGC
ATGCAACATGCAGCCTTTCCTTTCCAGGAATTTCTTGACTTTTTTTCTCTCTTTACTGTG
TCACTTGTACTTTTCTCTCGAATGTCGTTGTTACCTTCTTAACCAGATGAATAATTTGTA
TGTACGTGTATCATGTATTTTTCTTCCCTTCTTGGTAAAAAATGTATATTATGCGGAGTA
GTATGCATTATATATATGTATACTGTTGACCACTTTTTTGTGTATAAGTCATTTTCTGGC
GATTTTTGTTCACTGAAAAAACTTGAAAACTTTCAATTCTAGCTGCTAGATTTTAAGTAA
CTGGATCAGCTGAAAGGTGGAACATCTAATCATTTA
>BRADI3G40030
GAACCGGCAGCCTTTTCTTTCCCATCATATATACTCGTACATACGGGCACTTCCCCGGTT
CTCCTGTTCTGCGCTACGTACATAGCACTCCCACGCGCGCTCGTGCTCGTGCAGCCGTTT
TATTACCCAGCATGGTAAGAGGAGTCTCCTCTGCTATCGTATCGTATCTCCATCCCCTTG
GCTTTCTCATCAATCAAGATCAGCTACTGCTAGCTAGCAAAGCAAAGCAAAAGGTGCTGC
TGCTACTGGTAACCGTAGCTAGCTAAGCCACTGTAGTGTGTAGAGTATAGCATGTCAAGT
TGGGGGAAGAGAGAAAGAGTGAGTGAGAGACCCACACTAGGCTGTCGCAAGCACAGTACT
GCTGAGGACAAAGACACAGGAGAGAAGGGGGGTTTGAGTTTATTCCAAGGACGAAGCTAA
AGGCTGGCTGGTTTTTGGTTACCCGTGTGTAGGAGTGTTTGTTTCCCCGGTCGCCGCCTC
CCGCTCTCTCTTTCCCCTCTCGAGGGAGAGAGAGGAGAAGAGAGGGGGGCCAAGAAAGAAA
CAAGAAACCACCTGTGCAGACATCGCTTGCTAGCTAGTAGTAGCTCACGAAGGGCCGGCA
ATGGAGATTGGCAGCGGCGGTGGCGGAGGGAGCGCCGTTGCCGGAGGTGGCGATGGCGGC
GGCGGAGGGGACGACCAGCTCCGGCACGGGCTCCAGTTCGGCAAGAAGATCTACTTCGAG
GACTCCTCCAGTGGCGGGAGCAGCAGCGGCGGCGGCGGTGCGAATGCGGCGTCGTCGTCT
TCGAAGCCGGCGGCAAGCGGCGGCGGGAAGAAGGGCAAGGGCTCGGCGGCGCCGCCGCGG
TGCCAGGTGGAAGGGTGCGAAGTGGATCTCACGGCCTCCAAGGGCTACTACTGCCGCCAC
AAGGTGTGCTCCATGCACGCCAAGTCGCCCCGCGTCGTCGTCGCCGGCCTCGAGCAGCGC
TTCTGCCAGCAGTGCAGCAGGTCACTCCCATACCCGTCATTGCCTCCACCCCATTGATTG

CTCCTCCTACTCCTCTCTCTTGCTGCATTCTGCTCCGTCACCAATTTCTCGCCATTTTCT
GCATATGCATGTGTGTTTTTTTCTGGTCAAGCTAAAATGTGTTCTTGGGTTCAGTTTGAT
TTGATATGCTACTGGAGTATTATATTTGAGTGGCGGTCATGAGAATTCTCGAGTTTGGTA
GGTTGATTGGTTCATGTTTCAGTGATGTGACAACTCTGCCTGCCTGTTGCTTTGCATAAG
ATTCCTCTAGCTAGCATATATACACACTATACTATAGTCAATAGTAGCAGTTGTTGATTA
GTGGTAGTCTTAAAGAGATTTCATTTGTTGTATAATGAATTAATTTTCCCATGGTTGAAT
AAGAAAGAACTAGCATCTGCTTCATCACAAGTTGGTACTAGTCTGAATTTTGTCTAGTCT
GGGCCAGTACTCACCTGATGATCTTCAGTCCAAGCTCCTCCCCTCAGTTGCCTCACTACA
TATGCTTCATGTAGCTTCATACTTGATATTTATTTCTTGTTTTGACCAGTTCTATTAAGA
ACCACGGAGATGGCTTTCTAGCTGTTTTATGTGTGCTGTGTGTACTGCTGCTTTGCCTTT
TATGAAGTTGATAAAAGTTTGTTGATTTTCTCAAGAAATATTGAAAGGGTGCTTACAAAA
CTTTTGATATTTGAAATTGATAATGATTTTTAAGAAAGTAGAGCAAAATAACTTGTATAT
ATTCTCTGGGTGAGATTGCTGTAGGTTTCTGGCCAGACAGTTGTTATCTACTTTTTGGGT
CATTTTGAATCTTTTGCCATTTCTTTTACTTGTAAGGTTACACCAACCACAGAGATCCAT
GAAACTGTCAAAATAGTAATTATGTTTGAATTGCGCCAATCTCCAATTCTCCAGTGTCAC
AGAAATGTGGCATATATAATCAGAAAGCTACGGTAAAAGGACATCTCCCTCCACATTTGG
TGGATAACCCCCTTGGTGAACGTGCATTATTGATTATATTAAGAAGCCAGACATCTCACA
TCTGTATGAATGATTACAATCTTCTTTACACATGGTTCCTTTGTCCCTTTCTCTTTGGAG
ATTTGTTTTTCCATGCATGGATGCAATTATGCAGGGCAGTATGATAGTGTTATTTAATAG
AATGTGTAGTACAACCTCCGTCCAACAATACGGGATGTATCAGCTTTTCTTTGACAAATG
TTTTGACCACAAATTATTCTATTAATATATAATTATATGACATAAATTCATATTCGTAAT
ATTCCTACTCTAGGATATTTGTTTATGGTTATGATTGTGATCACATTAGTCACATATTCA
TGAAGTAATTTGTGGTTAAAGGCTTGATCAACCGAACTCTAATATGCCCCATATTGTTGG
ACGGATGGAGTACTATATTAAGAAGAGAGGCATTGTAAGCCCTGCAATAGCCTTTTGACC
AGACATGTTGTTTTCCAATTATGCTTGTTGCAAACCAATATACATATAATAGGTAGTACT
TGAGAAGCTTACTATATCTTTGTGAAATATGTGCTCCTTCAACTTCTACTAAAACATTTT
ATAACAACGAGCACTTTGTTCCATGAACTTTGCCTACTTGTTTTTCATTTCTACTCATGT
AAAACATATTTATACGATCATACACAATAAAAGATTGGAAGATCCTGTAATAAGCTTCTA
TTATGTCTAAAATTCACTCACCGTGTGCCTCTCGCACCACTGATTCTCCGTTTTGCATTA
TCGCAGGTTCCACCAGTTGCCTGAATTTGATCAAGGAAAACGCAGCTGCCGCAGACGCCT
CGCAGGCCACAATGAGCGCCGCAGGAGGCCACCCGCTGGCCCTCTTGCGTCACGCTACGG
CCGGCTTGCTGCATCCTTTGGTGATAGCAAACGCTCCTTTCCTGTTTTATCGCAATCTTT
TGTGTTCATTGACCACTACCAATTAAACTGTATTTAGTGATTTATAGTCTTCACATTCTT
GGTGCAGAAGAATCCGGCAGGTTCAGGAGCTATCTGCTGGATTTCTCATACCCACGTGTT
CCGAGCAGTGTGAGGGATGCGTGGCCAGCTGTCCGACCAGGTTACCGGATGCCCAGCGAA
GTTCAGTGGCAAGGGAACTTAGAGCCTCGTGCTCAGTCAGGTGCAGCCATGGGTTACGGC
GGCCATGCATACAGCAGCCATGGCTTCCCCAGCCCAGGGCTCCCTCCAGGCGGGTGTCTT
GCAGGGGTCGCTGCCGACTCCAGCTGTGCTCTCTCTCTTCTGTCAACTCAGCCATGGGAT
ACTACCACCCACGGTGCCAGCCACGACCACCGGTCCGCGGCCATGTCTGCCGCTGCCGCG
AGTTTTGATGGCAACCCTGTGGCGGTGGCCCCGTCCGTCATGGCGGGTAACTACCTGCCG
CCACCGGCGAACCCTTGGAGTGGCTCCAGGGGCCATGAAGGTGGGCGGAACGTACCTCCT
GACCCTCAGCTGCCACATGACGTCCCGCTCCATGAGGTGCACCCTGCAGGCTCTAGCCAG
CAGGGCCACTTCTCAGGTGAGCTCGAGCTCGCTCTGCAGGGTAACAGGCCTGCAGCACCT
GGACCACGCTATGGCGCCGGCCGCAGCACATTCGACCACCCTGGCAGCTCGACGAACTGG
TCTCAGTAGGGATCACTTCCACTACTACCCTCTGCCGTAGTAGTCCGCGCAAAGAGACAT
GGAACTGAAGAAGAGCCTGATATTGCCTACTGTTTGCTACCTTCTCATGTCCAGTACTAT
TTGAAGGAAACTCTGAAGTGGCTGGCGGTTTATCTTCTCAGTGATGCATAACATCCATGA
ACTCCTAACTATTTGCGCCCATGTAAACCTCCATGAATTTCAAGTATCAGTAATTCAGTA
TGCTTGTCGAGGACTTTTACTCGGCATATTATTCCCTGGTATTGCCACTGTAGCATTGCT
ATGACTTACGAATCAGTTCTTGTTCATGCAAAAGTGAGACCACACGCACAGCGTGGCTTT

ACAGCATCCTCCCTGCACCAGTTATGTCCTGTTGAAGGGTTGGCTTGGTGAGATACCGAA
ACCAATACTACAATACAACACCTTCTTCAGTCCAAAAGATTAGACCAGTGGTGAGAACTC
CGGCTATATCCTGCCCCGAACGTCAGCGTTTGCTTTCTTGTGAGAGATACTGCCATGGAG
AATGTGTTCCCTTTGGGATTCTGTTTCAAACCCTCTTGTCCTCTTGATTTGGCTATAACG
TAAAGACCGGGAGTTTTGATGTTTGCGATGAGACCTCCAGGTCCCAAACAGAGTCAGGGA
GCCCACATTTGGAAGGAGAATAAGGAATTCGGTGACACATGCATCAGCTCTGAATCAGCC
ATTATAGCATAACATTTGAACTCATATGCATAATCTTGTGGGCTGTGGCCACATGTTAGC
CTGTCAGGCTGCGATCAACCAACAGTGATCATGGATAGTTCGCATG
>BRADI3G40240
TGCCACGAGGAAACGCGCCTCCCTTGCCGCGCCACCACGCTACGCGCTCGCTGCGCCTTG
GTGTCTAGCAATTTAGCAATCAATCCCACGCGACCCGAACGAACCCCGTGCGCTAGCTGC
TGCTGCTCCGCTTCGCTTCCCTTCACACCCGGCTCGATTCGTCCCCCCTTTCCCTCCTCC
TCCTCCTCCTCCTCGGTCTTCGTCTCCTTCTCCCCCCCCTCTCTCTCTCATCACGCCTTT
CGTCACCGCCGCCCTGCCCCTTCCCCGGCCCCGGCTTCTGCCGTTTGTTCCGCGGGGGGA
GCTGCTCCTCCCTTTTGTTTGTTGTTTATGGGGGCGAGGTGAGGTTGTGAGGTGGTTCCT
GTGGGCGGAGACTTTTTTTTTGGTTGAGGTGCCGTGTGATCCGAGGTAACCTACTGCTCG
AATCGAATCCCCCCCAACTGCTGTTCGTTCGTCTTGGTTTCCTGATCGGTCTCGTTCTGA
TCCGTGTGCTGTGTGTTGTGCGCAGATCCAAAGAGCGAGTGATCCCGTGTGTAGCGGCCG
GCCGGGGGAGCGCCTTAAGCGGCGGCGGAGCGGGGGAGAGGGTCCTTGGGTGGGAGGGAG
ATGCAGAGGGAGGTGGGCCCGCAGGTGGCCTCCCCGCTGTACCTGCACCACCAGATCCAG
CCGCTGCCTCCCCATGCGGTGGCGGCGGCGCCCAAAAAGCGCGGGAACCCGTGGCCCGCC
TCCGCGGAAGGCGCGGCGGCGGGGTCTGCCGGCGCGGGGAACTGGAACCCCGCGATGTGG
GACTGGGACAGCCGCGCCTTCACCGCCAGGCCCTCCTCCGACGCGCTACGCCTCGGGGCC
GGCGCCCAGAATCATCATCATCATAACCACCACCAGCAGCAGCAGCGGCAACCGGCGGCG
ATGGCTGCTGAGGCGCAGCAGCGGCAGGGCCCCGGTGGGCTGAGTCTTCAGCTGGCCACG
CGGGAGGAGGCGTCGGTGGCGATGGATGTCAGCCCAACGGCTATCATGTCTTCCTCGCCT
TCTCCGCCAGCAGCGCCGGCGCATGAGCAGGCCGCTCGGCCTAGCAAGAAGGTCCGGTCT
GAATCGCCGGGGACCGGTTCTGGAGGCGGCGGCAACGGAGGTGGAGGAAGCAGCGGCAAC
GGAGGTGGGAGCTACCCGATGTGCCAGGTGGACGACTGCCGCGCGGATCTGACGAGCGCC
AAGGACTACCACCGGAGGCACAAGGTCTGCGAGATCCACAGCAAGACCACCAAGGCGGTG
GTTGGCCACCAGATGCAACGCTTCTGCCAGCAGTGTAGTAGGTAATCATAGTAGCACCCG
CATCCCCTTCGCAAAATTTTACTGGCATGATTCGCTGTTATGATGCTGCCGCTTTTAAGG
GGATTTGTGTTTATGATGCGAATGGTTGCTATGGTGTGTGTATTTGGAAGGAGAAGTGGT
TCTCTATCCAATGGTTGGCTGAGTGCATTTAGTTGCTACTAAAAGTCGGTTCTTCTGGTC
AATGGTTTAGATTTCACCCCCTCTCGGAGTTCGATGAGGGTAAGAGGAGCTGCAGGCGAA
GGCTCGCCGGGCACAACCGGCGACGGAGAAAAACCCAGCCCACAGATGTTGCTTCACAGT
TGCTGCTACCTGATAACCAAGAAAATGCCGGAAATAGGACACAAGATATTGTCAATCTGA
TCACGGTTATTGCGCGCTTGCAAGGTACTAAATTCACTTGCTACTACTGAACATAGATAC
ACCCTTACACTCCCCTGTGATTTTAAACAGCTTCCATTTTTTCTCTCTGCAATGACAGGT
GGTAATGTTGGTAAACTACCTAGCATCCCTCCTATTCCAGATAAAGATAATCTGGTCCAA
ATTATTAGTAAAATAAACTCAATAAATACGGCAAATGCTCTGGGAAAGTCTCCTCCATCG
GAAGTCATTGATTTGAATGCCTCCCACGGGCAGCAACAGGATGCTGTTCAGAAGGCAACA
AATGTAATTGACAAGCAAGCTGTGCCATCAACCATGGACTTGCTAACAGTTCTATCAGGT
GGCAACGGTGCTTCTACCCCTGAAACCAATACGTCCCAGTCCCAAGGGAGCAGTGACAGC
AGTGGTAATAACAAGAGCAAGAGTCATTCAACGGAGCCAGCTTATGTTGTAAATTCCCAT
GAGAAATCAATCCGAGCTTTTCCTGCAGCTGGTGTGATAAGGAGCAACAGCCCCCATGAC
AGTCCACCTGAAATGTACAAGCAGCCAGACCGAGATGCCCGCCCATTCTTGTCACTGCAG
TTGTTTGGTAGCACCTATGATGATATCCCTGCTAAGATGGATACCGCAAATAAGTACTTG
TCATCTGAGAGCAGTAATCCTATGGACGAGAGATCTCCATCATCCTCTCCACCTGTAACC
CACACATTTTTCCCCATCCGTTCAGCAAACGATGGCATCACGCATCCTCGTGCTGGAGAC

TATGGAGAAGATGCTGCAACAGTCGAGAATAGTACCACTCGGGCATGGTGTGCACCACCA
CTTGAACTTTTCAAAGATTCAGAGCGGCCAACAGAAAATGGGTCACCACCAAACCTCACA
TATCAATCATGTTATGCCTCAACTTCTGGTTCTGACCATTCACCATCAACATCGAATTCA
GATGGACAGGTAATTTGTGTTGTAGCTTCCTGTTTCAATTTGTTATTATGCACGTTAGCT
AAGATGTCATTTATTATTCCAGCCATGCCTTCAATCTTATCTTTGCTTAATTTGCCATTT
TACAGGACCGTACTGGTAGGATTATATTTAAGCTTTTTGGCAAGGAACCTGGCTCAATCC
CTGGGAACCTTCGTGATGAGGTATGCTAATGAAACTGACAAAATGTTTTGAAGCCTGCTG
CAGCCCCTTTTCCAGCTCTTTTCAGTCAACATATAATTAAAATATCATTGTTTCTTGCTC
AGGTTGTAAATTGGCTCAAACACAGCCCCACTGAAATGGAGGGTTACATTCGCCCTGGTT
GCCTTGTACTATCCATGTATTTGTCTATGCCAGCTATTGCATGGGATGAAGTAAGTCTGC
CGCTAAACTAGCGAAATCAAAATTATGCAATAAAATTGGTCCTGCATTATCTAATGTCGG
ATCATGCTATGTCAATGCAGCTTGAAGAGAATCTTCTCCACCGGGTAAACACATTAATTC
AGGGTTCTGATTCTGATTTCTGGAGAAACGGAAGGTTTTTAGTTCGAAGTGACAATCAGT
TGGTGTCATACAAAGATGGTACACATGACTCTTTTGTTGCTAATTTTCTTCTCTATTGCT
GTTCATCAGAGGTCATTCTTTGTTCTGGTTTGATTATTAGTTTGTTTGGTTGATATCAAA
TTTGTATTCCAGGAACGACTCGCTTATCGAAATCATGGAGGACATGGAATACCCCTGAGT
TGACCCTTGTGACACCAATTGCTGTTGTTGGTGGGAGAAAGAGCTCCCTCATTCTTAAGG
GCCGTAATCTAACGATTCCTGGCACCCAGTGAGTTCCCTGTTGTAAAATAATTGCTGACC
TGACAATTATATCCTGAATATTTTTGACTCACCATGAACTGTCGGTCTTTGCAGGATCCA
CTGTACCACTGAAGGGAAGTATATATCAAAAGAGGTACTATGCTCGGCGTATCCAGGTAC
CATATATGATGATTCAGGTGTCGAGACCTTTAATTTACCGGGAGAACCAAATCTCATTCT
TGGGCGTTGCTTTATTGAGGTTTGTCTGCCTGTTATATCTCTGCTGTTGATCTTCATAAG
TAGAGATTAGCTCAAGGAAGGTGATTACAATTTGCAATTATAATGTCCATACATGCAGGT
CGAAAACAGGTTCAGAGGAAACAGCTTCCCTGTTATATTTGCCAATTCAAGCATTTGTCA
GGAGTTGAGGAACCTTGAAGCTGAGCTTGAAGATTCGCGATTTCCTGATGTCTCTTCAGA
AGATCAGGTTGATGATACTAGGCGGTTAAAGCCAAGGGATCAAGTTCTGCATTTTCTTAA
TGAACTTGGCTGGCTCTTCCAGAAGGCTGCTGCTTGTATACCCTCCACCAAATCCGATGT
TTCTGATTCGGAGTTGATTCAATTCTCAACTGCACGATTCAGATACCTTTTGTTGTTTTC
AAATGAGCGGGACTGGTGTTCTCTTACAAAAACACTACTCGATATTCTTTCCAAGAGAAG
TTTGGTCAGTGACGAACTATCACAGGAGACTCTGGAGATGCTCTCAGAGATTCATCTCCT
GAACAGAGCAGTGAAAAGGAAGAGCCGCCGCATGGTGCATTTGCTCGTGCAGTTTGTCGT
AATTTGCCCTGATAATTCCAAACTGTATCCCTTCCTTCCCAATTATCCTGGCCCAGGTGG
TTTGACTCCATTACATCTTGCTGCATCCATTGATGATGCAGAGGGTGTAGTTGATGCCTT
GACAGATGATCCTCAACAGGTAAGCACTTTAAATTTAAACCTGGCCTAGTCTTGCTTATT
TCACATCGATAATTCTTCTGATTTTTTTAAACTTAAATCAATCCAGATTGGTTTGAACTG
CTGGCACTCAGTGTTGGATGATGATGGCCAATCTCCTGAAGCCTATGCGAAGTTCAGGAA
CAACGATTCGTACAATGAACTCGTGGCACAAAAGCTTGTGGACAAGAAGAATAGCCAGGT
TACCATTGTACTCAACAAAGGAGAAATTTGTATGGATCAACCTGGGAATGGTGGAGGGAA
TAATGCATCTGGGATCCAAGCAATGGGGATAAAATCTTGCAGCCAGTGTGCCATTTTGGA
GTCTGGCTTGTTAAGCCGCCCTATGCATTCAAGGGGATTGCTTGCACGCCCTTATATCCA
CTCGATGCTTGCCATAGCAGCAGTTTGTGTCTGTGTCTGTGTATTCATGCGAGCCTTGCT
GCGGTTTAATTCTGGCAGGTCCTTCAAGTGGGAGAGGCTGGATTTCGGTACATCCTAGAC
TTTGCAGGACATCTTATTGCTTAGCCGCATGGTATTATTAAGGCAAACACCTTACAAGAA
CCAGAGAGTATGGGACAAGTTAGCTGTTAACTGTTTTACCTTTCCATGGAACTCTGTCCA
TGGATATGGCTGGTCATGTGCAATACTTATACTGATAAAACACTCCCGGAAAAGAAGTGT
TGATGGGTTGGGATGGCTGCAACACTTGATGGGAGTTGAGAATCAAGAAAAAAAAAGGAAT
CAGGCAAACCCTGTCAAAAGGTGAACTTGGGCAGAGTTGGTACATAGTTTTTGAGACGAG
GAACGGATTGCTGCTCTCCTACAAGATGAGATGGAGGCAGCAAATAGTTCTGGGCTAAAC
GAGTAAGTAACACCCTTGGTTTCCTTTTTTGCTGGTGTAAGTGTGGATCTGCTATGTTTC
ATGTATCATAAGATGGTTGTTTATTTATCTTGGCCTTTGTTTCTGCTAAATTTTTTGTGT

ATCAAATACAGTGCCTAGCATGGAACTGTTTTTCTGTTGTCTCTTAGATGGTAGCTGGGG
GTATAAATGGGAGAGGCGTCGTGGATAGTTGTTGTCTGTAGCTAAAATAGAAAATGAGGC
AGATTTACATGTGTATCCGGATCTATTGTTTTATTTGATTTTATTTCAAAACTCTGGAGT
ACCCGTTTTATTAGAAATCTTCGGCATTTTTTATTTTTCTTCGCACACTTATTTTTTCTC
TCGTTATAACTATTAATGCCTGCTATGCTGATTCTATTCCTTGCACACATCTCTCCGTCT
GTCCATGTGTAAATTTGTTAGAGAAAATCACATTTGTTGCCAATTTATCGATTTATTTTT
TAGTCGGCATATCCGAATATTATGAGATAAAGAAAGTCCTTGTCTGATGAAACAAACACT
ACACGAAAACAGTGTGGCAAGCAACTACTATGCGTGTGTGGTGGGGGGGCCTGGGGGCAT
CAGGGCAACCGTGTCTCGCAAAAGGCTGCATGCCTTGTGCCCTCGTGTTGCGAATGGGAT
GCCTTGCCTTCCTTTCCCGGTTCCCGCTCGCTGTGTGTGTGGATCGGCGCTAGGACACGC
CGTCCAGATTCTGGCGAGCGAACTGAGCGCATCCCATCCCGGGCAAGCGTGACCTGCACT
GCTCTCTGGGGTAGGAAACGATGCCTTTCTTCTCTTTTCCCGGGGGTGCAACATGCACTT
GCAAGCAAGCGAAGAAGACGCAAACGCGTTCTACGGGACGGCCCCCGTGTGCGTCCCGTG
GCCGATGGCTTTCCGGCTGTCGGACTGGACGCTCCCGTTGTTCGTGGCCACACGTTAGTA
CGGAGTACTTCCCAGGAAGTGTGTATCCCGTCC
>BRADI3G41250
CTCGCTCAAATGGGAGTAATTTTTATGAACATATTGTCCTAAAGAAACCTAGCACACCAT
GTATTATTGCTGCTGCGTATCTTTGTTGCTCCCTCTAAAATCTATGTACCTCTCTCTCTG
TAGACCCTGGATAAGATTTTAGAAGCTCCACTGGTCTCTCAGTCAGTCTCTCTCTAGTCT
GTAGCACTGCCATCATCCAATTGTTTGTTGCATCTGCCTGCGGGTGTTATTATTCGACAT
CTTTGGTTTGGTGCGGCAATTAATTAATGAACCGTCCGTGGTGGATCCGGTAGTACGTGC
AGCTGGCTGGCCCGGCCACACGTACCAGTAGGAGCTTACTGCTGCTGCTACTACTAGGTG
GGAACAGCGAGAGACAAGAGCACCCGCGGAATGAGAGAAGCCGATGGAGCTCGGACAGCG
ACCGGAGCAGAGCTTCTTTGGGCTTGCGTGTGAGTCTCGTCTGTGTGAGTGAGAGAGACA
GAGACAGAGTGGGAGATGAAGGAAAGGGAAAGGTCGGGAGCTTAGGCGATACTACTGTAG
TAGTAGGAACTAGCTTAAGATTAGATTAGCTTAGCTTAGCGGGGGGCTCCGGCGGTCGGC
ATGGACTGGGATCTCAAGATGCCCGCGGCCGGCGCCGGCGCCGCGTGGGACCTCGCCGAG
CTGGAGCAAGGCGGCGGAGGCGGCGGGCCGTCGGCGGATGGCATTGCGGCTCCTGCTGCT
GCTGCGGGCGGTGGTGGACGGGCCGAGTGCTCCGTGGACCTGAAGCTCGGCGGGCTGGGC
GAGTCCGGCGGCGGCCAGGCCCAGGCCCAGGCCCAGGACAGCAGCACCCGCGGGGGGAAG
GCGCCGGTCGCGGCGTCGGCGCCGGCGCCGGGGAAGAGGCCGCGCGCGGCGGCGGGGTCG
TCGTCGAGCAGCGGCGGCGGCGGCGGCGGCGGGGGGCAGGGGCAGCAGTGCCCGTCGTGC
GCGGTGGACGGGTGCAGGGCGGACCTGAGCCGGTGCCGCGACTACCACCGGCGTCACAAG
GTCTGCGAGGCGCACTCCAAGACCCCCGTCGTCGCCGTCGCCGGCCGCGACATGCGCTTC
TGCCAGCAGTGCAGCAGGTAACAGGAATCATCTCGGCAATCCAATCCAGATTATCCAATC
CAGTTCTTCGCCCGCCAAATTCTAGGCCAATGTTTGTTTGATTGCCATATTCAACCAACG
AACTAACTGACACCACTATACTGCTCCAGCATATAGTATAGTACTAGTAAATCAAAATCA
TACCCACTTCAATTGTCCTCTTCATCTTGCAATTCCAGCCCTGTTCATGGTTTCTGTTCA
TCAATTACTTCCTCACACTATCAGTTTTTAAGCCCCACCTTCTTTTTCCTTTTCTTTCAG
TCTCTGTGCATGTTTCTGCCTGTCTCATTTCAAGGATGCACAGCTTTACCTTTTCCGGCA
CCAAGCCTGTTTCAGCATCTCATTATTTTTTTTCTTCTTCTTCTTCCCATACCAAATTCT
CATTTTTCTNNNNNNNNNNNNNNNNNNNNNNNNNNNNNNNNNNNNNNNNNNNNNNNNNNN
NNNNNNNNNNNNNNNNNNNNNNNNNNNNNNNNNNNNNNNNNNNNNNNNNNCGGTCTCCGCA
TAAGCAAATATTGTGGTTTTTTCTTCTTCCCCCCCCGAAATTTTTATTTTCCTCTTTTCA
CCCTTATTATAATGGCCCCCAGATACCAGAATTTATGCCGGGTTTTTCTTTTTTTTCCCC
CCAGGGTTTTTTAATTGCCCCCCCCCCCCCCCCCCCCCTCACTCACTGAATCATGACCCCAT
CCATGAGAATAATTCAGGATCGGAAAAAAAATGAGAGGTCATATCTGCACTCCATCTGGC
AATCACACAGAGGCACCACCCAATGATCATTAATCGACCGGCCATCGTGCCCAATTTGCA
AGATGGCATGTATGTTTAGTCAGTTCTGAAGGCCAATCATGCCTTTGATCAGCCATGTTT
CTCTCTGTTTGCGACACTTATGATACCGGTTCCGGTGACGCGACACGCGGTTGATGGTAG

GCCGGACGTCCGACATGATTTGCCGGTGACTCCTTTTCCATGTGCAACTAGCTAGAAATA
GGTGGATCCATGTGCCTTGTTATGCCTGTCCCCTGTGGATTTCTTTGCTGCAAAAATGTT
TTGCGTGGGACTGTCAGTTCCTTAAATATCTGTGAACCTTCTGTTTGAAAGGAAAATGCG
ATATGCCGATATGAGCGCCCAACCCTAGAAACCTTTCAGTGCCATGAAGTATGTAGGGTT
TACAGATACACGTTGATTCCTCAGGTTGGTCAGGCTTGCCCCCCTTCCATGGGCATTAAA
CCTTTCCATGTTTTTACTTCATTAAAAGGTTCATGCTAGTGGCAACTGACTGATGTACCT
TAAATATTAAAAGGCAATACCAACCTATACAGGGTGCTCTAATGATTTGGTTGGTGACCA
TGGTAAAGAAAGAGATCTCTAGCATCCGTATTTGGACTTAATATCACAAAAGCCAGAACT
ATGTTGTGAGGACTGCATTTTTAGGTGGTCCTCGGAAAAGAAAACAAAGACGAGTATCAT
ATCGTGACAATATCTGATTTTTAAACATTAGTGCCAGCATACTTAATAATAGATGATAAT
GTGCTTCTCTCATGTGCTCTTGGACCACTTGAACCTGTTTTGTCTAAGTTTCACTTTTGC
AATTGCCCTGTGAAGATAGCTTCCCTATCTTTTTGTGCATAATTTCACCATAAACTGAGT
AAATCACATCGTGTGAACTGAGCTTTACGGATCAAGTTTATCACACTAAGATTTCCAGAA
ATTACCACAACGGCTTAAACTTAGAAATTAATATAGACGTACCGAGTATCCTCTATCCTC
AACCTGAGCCATCCATTATTATCTGTCAATTAATATTATTATTTTAAGTTTTCGGCCTCT
AGAATTATTTCTTACATTTGTAATTTTGTTGTATCTTGGTCAACTTTAAAGTATAGTCAT
GGTTGTGCCCATTTCTATCATGCTGAACAAAGCAGACGATTCATTTTTTTTACGAGCAAG
GCAGGAGCTCTGCCGATTCATTTAAGAAGAAAGAAGATACAACTGCTCATCGTCCGACAA
CAGAAAGCAGACGATTCTATTCTACCTTCACGCCTTTATAACTCGATAGGATGCTAACCA
TCATCATATTAGTTCTTTCCACACAAGAATATCTTACTCAGTGGGGACAGTTTTTCCAAT
GATTGATTGCTTCGTCTGTTGGTACAGATTTATAAATTTGGTGACCCCAATGTCCCAATG
AACTTTCATTCTTCTAAAAAATATAGTTTCATATACAGTATACTGTGGTCCAAGTTCAAC
AGTTGTTACAATATTTTATTTTTCTGTCATTCACAAATCTTCGAAGGCGCCCAATCTCTG
AAAGGGATAATTGACAGATTATGTGGTCCCTTGTGACATTACCTATTCCAAGAGTATTCT
GTAACTAGTTTCGTGTTTTTTTTATTGGGAAGCATCTTTTTATACTTCGGATCATGTGCC
TAACTACCTAGGAGCATCTTTTGTCATTACAGAATCAACCTAGGACCATGAATATCTCAT
TTAGTTTTTGCAAAAAGTCTAAGAGTATCTTGTATCTTTAGAAAATAAAATCATTCCAGA
TTGTCCCAATGGATTAGGTTAATATTACTTCTGTAGTTGCTGTGTTGCAGATTATTTTTC
TAAGTTAAATGCCATTGACTGATCATGAATTGTTTGTTTCTTTAGGTTTCACTTGCTTAC
AGAGTTTGATGAGACCAAGCGTAGCTGTAGAAAACGTCTTGATGGGCACAACCGTCGCCG
CAGGAAGCCACAGCCAGATCCCATGAATTCTGCAAGTTTTATGACAAGTCAACAAGGTCT
TTTACTTTTTACTACTGCTTTATTTTTAACAGTCAATACATGGTTCGAAACACGAAATAG
TTTGTCAGACATCTTGTAACTCATGGCTCACCTAGTATGTTTGACTTATTAAATTCACCA
GCTTCAGCATCTTGCAGCTCAGCAACTCACCCGGTAGCCTGTTACAGTGTTACTCCACCT
TGTTGCCCATTTGTACCTTATCCAGCAATTGTCATCCTGTAAATTAGTCAGTCCCAGAGG
TGGTTAGTACTCACAAAGGCATAGATAGCACCATATTTTACTCATAAACATACCCAGTTC
ATAGAGGTTGGATATGGTGTAAATAGTTATCTCATTCCACTTGTCACACAGGTCAAAGTT
CAGTTCTGTGGGACAAATAAATTCTTTACTCTCTGCACAGTTAAGAGTCCTTGTGTCTGG
CCTAAGGTCTTACTAAGTCCTAAGTCGTGTTAAGATCATATCAATCCCTGCCAAGAGTAG
ATGTTGACTTACAATTTCAGTGCGCTTGTTTTTTTTTGACGATGTGCTTGTTAGATATTG
ATATTAATCACTTATTGTAGTGCTAAATTCATTTTAAAATTAACTCTACTATCATATGAG
GTTTCCGTATCAACCTCATTCTTTGTAGCATTAAATGGTTGGATAATACTATGACTTTAC
TCATTTGTATTGCCATATGTATGTTCACCACTTTCATTACCTTCTTATTTATCGCAACCA
ATTGACATTGTTCTCAAACTCAGGAACAAGGTTTTCACCATTTCCAAATCCAAGACCGGA
GCAAAGCTGGCCAGGGATCATCAAAACTGAGGAGAGCCCATATTACGCGCATCAAATCCC
TATAGGTATCAGCAACAGGCAGCATTTTGGTGGATCTACATCTACTTACGCCAAAGAAGG
CCGGCGCTTTCCTTTCCTACAGGAAGGCGAAATAAACTTCGCCACAGGGGTGACCCTTGA
GCCTTCAGTGTGCCAACCACACCCGAGGACGGCAGCTCCTCCCGATAGCAGCGGCAGCAG
CAAGATGTTCTCTGATGGGCTGACTCCTGTGCTCGACTCAGATTGTGCTCTCTCTCTTCT
GTCAGCTCCAGCAAACTCCTCTGGTATTGATGTCGGCCGGATGGTCCAAGTCCAACAGAA

CGAACACATCCCCATTGCTCAGCCTCTAGTCTCCAGCCTACAGTTCAGCAGCTCGTCCTG
GTTTGCGCGCTCCCAGGCTGCCACCGGTGCCGTCCCACCGACCGGATTTTCCTGCCCTGT
TGTGGAAAACGAGCAGCTCAACACTGTCCTGAGCTCGGATAACAATGAGATGAACTACAG
CGGGATATTCCATGTCGGCGGCGAAGGCTCCTCAGACGGCGCCCCGCCATCTCTACCCTT
CACCTGGCAGTAGTAGTTCCCAGTAGCTGTTCTTGGCGTGCTTCACAGTAGAGCTCTTCC
CTTCTCCGGTTCTCCCTTTCCCTAGTCCCTAGTGATCAACACTGTTTCTGTTCACTCTGC
ATCATTCCAATTTTTTCTCCTGTATTCATTTCACTCAGCAAGCATTCGCGGTCAGCAAAT
TCATCATAAAAGTCAGTTCCTCGAAAATCTTTTTCTCATCATGTTGTCAAGTACTCCCTC
TATTTCACAAAGGTTGGCGTATTTTGTTTCCTTAAGACAAGGCTTTGATCAATGATAACT
CTATTAATATGTGTTTTTACATAAGGGAGTATATTATTGATCGTCAAGTGCCAACTGTAG
AATTATTATGCTCCTGATCTTTTCTCGTTGATTATTTCCTAATGCCGATCTTCTCTTTGA
CTATTATTACGAAAACATTGCACTGCATCTAACTGCAAACGGCAGCAGTGAGCTCCACAT
GCCCGTTTGGCATGCAGAGTCAACTGAACAGATTTGCAACAGCATCCTAATCTGACTTCT
TTCTAAAACGAAAACTGCAAATCAAATAAGCTATAAAGGAAATTTTCCTTAGGAATAGAA
TCCTCCAAATTTTTTACTAGTGCATATGATCAATGAAGATAAAATTAATTGAATTGGACA
ATAAATTTAGAGCGGTAGATCAATGATACTTATCCACTCGCAGACCACCTGCACGGTCTA
GTAGTGGCCATCCGCCGGGCAACCGAACCAGCGGCAAAGCGCAGCGATGTGGCTGATGAT
CATTAGGGTGCTCCCAACGATCAGTAAGCTTCACGGCCGTACACGCGCGTTGTTGCACAA
CAAATCAGAACGAGTAAGTACGTTGGAGATATTATATGTCCGAACTAGTTGTGCCAACAC
CGAATTGAATTAATTAGC
>BRADI4G33770
CCGCCTGGCACTGGCAAACCTGCGTCTGGGAGGTGCGAAACCCCACGCCCACGCACGCGG
CCGCCCGCCCGCGTATGCACTCTTTCAACCCCCATACAGTTCAAATACGCACCACGTACT
TACTTCTATACCCCTACTCGAGAACTCCCGGAGTAGGAAAGGTGTGGAGTAGTTGATGAC
TCGTGGCATGAAAAGAGGGAAGCAACAGAATCTGGCTGGGGGAGGAAGTGGGAGTGCGTG
AACTACTTACTGAACGGGGAAGTAAAGAAGCAGAGCACAGAAACGGGCACACAACGCACG
CACGCACGCACTGCACCACACCGGCGGAAAAAAGCTTTATTTTAAGCGCGGCGCCACCAT
TGCTGTCGTCCTCCTATGCCGCTTTCCATCTTTTCGCCCGCTCTCTCTCTCTCTCATCTC
CACCTTCTTTCCTCTCTCCAGCCAGAGCGGAAGAGGAGCGGGGCCGGCCAACAAAGCCAG
CCGCCACTATTTATCAGTTCCCTGCCTACACCACTCCAGCTACCAGTAGCTAGCAAGCCC
CGACTATAATACTACTACGCGTGTTCTCGCAAAAGCAGCGGACGAAACGCCGGCGCGGCA
ATGGAGACCGGCAGCAGCGGCGGCGGCGGCGGTGGGGGAGGCGACGACTTCCACGGGCTC
AAGTTCGGCCAGAAGATCTACTTCGAGCAGGACGTGGCCAGCGGCAGCGGGGCGACGACT
TCCGCGGCGGGCGGCGGCGCTGGGGCCGGAGGAGAAGGAGCAGCAGCAGCAGCCTCTGCC
GCCGCTGCGGCCGGCAACGGCGCCGCGCACGCTCAGGCGCAGCCGCCGAGGTGCCAGGTG
GAAGGGTGCGGCGTGGATCTGAGCGGCGACAAGACCTACTACTGCCGCCACAAGGTGTGC
TCCATGCACTCCAAGGCGCCGCTCGTCGTCGTCGCCGGCATCCAGCAGCGCTTCTGCCAA
CAGTGCAGCAGGTAATACAGTATGTATGTATGAAAACACTCGCCCCTACTTGTTAATTTG
AGCTCCTAGCAAATTTGAGCTCCAGCAACATGCAATTTGAGCTCGCGCCTCCAATTAATC
CAGTTTTAAATCTGTTTCGTCCCGGCCTTCTGGACTTTGTTTGCCGCTTTTATTCCTCTG
CTTCCATCTACTAGCTACTGTTGCACTCGTGACACTGCCCTACTCCTCGAAGCCAGCGGA
GCTGTGGATGGTTGCTGGCAAATGGCAATTGTTTTTTCTTCAGAAATCCGCATGTTTGAT
TCGATGTTCGAGTTCCTTGGGTGGGTTGCTCCAGCATGTGGCTGCTGTCAGCATGCAAGC
AAAGGATCCTCAGCAATTGGCCCCAATCAGGACAAGAACACAGTCTTGCAATTGGCCAGA
ATAACTACTAGTACTGCTTCTTTTATTCGCAAAGAAAGTACTGCTTCTTTGCTTTGCCAC
ATTCAGAAATTCAAGTGCTACTTGAAAAAGAAAAAGAAGTTCAACGGTTTGGTTCATATG
GTGACGCATGGGTGTAGTGTAGTACTGCGTGTGTTCACTTTATTTGGTTTGGTTAAAATG
AAGCAACCAATGCAACATTTGGGCAGGTGGAATCTGACCAGAATAGTAAGGGACAGAAAA
GTGTACACCCAGGAATTCTTGTCCGAGTGGCATATATAGTAAGCCTAGATTTCTTTTTCT
TCTTCTTCTTCTTCTTCTTATTCCACTGTATGGTAGGATGAATGCTCGTACGTTGGGTAG

TTTTTTTTGGGATAATGGTACATTGGTAGTACGAGTACTGTATACTATTTAATGCCCTAG
TGTTAAGTTCGATAGGAGAGCAATTTGGTTGGGACAAATGAAATGGAGTCCCAAAGATTT
CCTGCTTGTGCTGATGTTCAAATAATTACTACAGAAAACTTAGAATGCACTATCAAATAC
TCTTTTTGTCCTGTATTAAGTGTCGCAACTTTGTCTAAACACGTGCGTTTTAGTGTGTAT
ATATATGGATATCCAGACAAAGTTGCGACACTTCATACGAGGGAGAACCGTAGTTTTTTT
AAAAAGCTGACTGAAGTAAAAAAAAACTCCCGTGCACTACAGCACATTGATGGATGAACC
TTTGTAACTTGTAAGTACACAAATTTGAACCGGTAGTTCACAATTTTCCGGTGGCTTTGT
GTGGTCTTATGACTGTTGTTGTACTATAAGCCTATGTGCATTCAACTGTCTAGGAATGAT
TACAGGTGTTCTATCCACATGTCCTTTTTTTTCCTGGACGTGATGCGTGGATCTAATCAT
GAAAGGCGTTCCTTTGGTTACAGTTTTAGTCCAGGATTAAAACAAATGGTGTACTTGCTT
CGATGCAGCTTTGCACATGGCATGTGATTAGCTCTGTTTAAGTATTCATTTGATGCATGC
ATGGTATATTAAGCTACAGTATCTGGTTCTCGATTACAGAGTTAGCATTTTCCCTCTTTC
CATGTGCATGCAGATTTTTATACTCATAAACGATATGAGATGCAATTGATATACGAATGC
TACTTGCTCTGGTTTTGTTGTCTGAATTTGACTGTTGTTGTTCATTTTCTGCTTACTTTT
TGTATGACGTTCTTCCTTATGGTGACTCTTTTATCCTTTGTTAATTTGACAAAATCACCA
TCTGGACTTAGTATGTTGGATATGTAAACCTTTCTGCACCTAAAATCGCAGGTTCCACCA
GTTACCTGAATTCGACCAAGGAAAACGAAGTTGCCGCAGGCGCCTAGCGGGTCACAATGA
GCGCCGGAGGAAGCCCCCACCTGGTCCTCTGTCTTCGCGCTATGGCCGGCTTGCCGCGTC
CTTCCATGGTAACTAGTGCCTCATGCAGTCAACAGTTTTAGCACGAATTAGTCACACACT
TGCAATTTAAACGAAACGTTAATGTTTTTGCAAATTTTGTTTCAGAAGATCCCGGCAGGT
TCAGAAGCTTTCTGCTGGATTTCTCGTACCCAAGGGTTCCAAGCAGCGTGAGGGATGCGT
GGCCAACGGCTCATCCCGGCGAACACCACCGGATGCCCGGCACGGCAGCCCAGTGGCAAG
GGAGCCATGAACTCCATCATCCTCACCGCAGCACAGTTGCCGGATACGGCGTCGACCACC
ACGCTTACGCCGGCCAGGGTAGCTCGTCGGGTGGCGCCGCGCCGATGTACCCAAGCTTCG
AGCTTCCCGCGGCCGAATGTATCGCAGGAGTCACGGCCGCCGACTCCAGCTGTGCTCTCT
CTCTTCTGTCAACTCAGCCATGGGATCATAGTGCCCACAGCGCCAGCCACAACCGGCCCC
CGGCAATGTCGACGGCCAGCGCCTTCCAGGTCAGCCCGGTGGCGCCATCCGTCATGGCCA
GCGACTACATGGCGGCGGCAAGCAACAGCGCCTGGGCTAGCACCCGGGGCAGGAACATGC
AGCAGCACCAGCAGCAGCAGCATCACCATCATCATGACGCTGTGATGAGCGATGTCCATC
CAGGCTCGGTTCACCAGCATGGCGGTCAGTTCGGGGAGCTCGAGCTCGCGCTGCAGCAGG
GGAGGGCCGGGGCGCCGAACACGCCGCACGCCGAGCATGGGTCTTCCGGCGCCGGCGCCT
TTGGCCACCACTCCAGCAACGCCATGAACTGGTCTCTGTAGGGGATCGACGAGCTTCTCT
CCGGCCGGCCGGGCCGGACGGCATTCGCATTGTCACGCCAAAGAGACATGGGACATTGGG
AGGCCTGAGTTTTGTGCGCCTTCGACTTGCCGATTTGGATTTCAGGATGAGGAGGAGATT
CTTAGCTGGTAGACCTGGAATTTCTCCCGTGGAGCCCAAGGTGTACATGTACTCGACAGT
TTGATCCCACTGTGTAAAACTCCATAGCTTTAATTCGCTTCTCGAGGACTCTTTAACGTA
TTGATCGATCTCGTTGCCGTCATCCTCATCTCGTCGTTGTTTCTGTTCATCTACTAGTAC
TCCCATAAACGATCTGAGTTGGTGCTGATGGTTAGTTCACACTCAAGTCGTACCGATCAT
TTGATTCGCTACCGTACCCATGGCTGGCCGAGTTGGAGATGATTTGCTCACAAACGAATC
TAGTTCACATTGAAGAGCAGATTCTCCTTTGAAGCACCCGCTTTTTTTTGTTTCACTTCG
AATCCGATCTTTTTTTCAGATCTTGCAAACACGTTTTAGTACTCCCTCAGTCTAACAAAA
AATGTCTCAAGTTTTGTAAAAATTTGGATGTATCTAGATGT
>BRADI4G34667
GAGATTGTTATTTTGAGCTCTATTATTTTTTTACACCTCAATTCCTATGGCCCTTCTCCT
CCTCCTCCCCTACTATAGGGCCTCCTGCCCACTAGCTCCATGGCTGTCCGTCCTCCATCG
CGCACACCTGCACTTGCCTCTGCAAGCAAGTACTGACTGCTACTCCCCAGTCCCTTGGCA
TTTTTGTTGTGTTGCCTGTCTCTCTCTGAGCCTCTGTGTGAGTGCGTGCTCTTGCTGCTG
CCCATTCCCAGTCCCTTCTTCACCAGCTCGCTCGCTCTCTCTCATCCCTATCAGGATAAG
CTGCCCCTCCTCTCCCGCTCTAGCTCCCTGCCGATCTGGTGCAATTGCTCGCGCTTCGAT
TTGTTGGCGTCTGGGATTGGGGGCGAGCTGTGGTCACCATGCAATGATTCTTGGTTGCTA

GCTACTTGCCGCCCTCGTCGCGGCATGCCTGTGGAGTGACAGGGACCGGAGAAAGAGAAC
AAACCGGCAAGCAGTGACCTGACCGGGAGGGAAAGAGAGAGAGATAGCTCCATGGATTGG
GATCTCAAGATGCCGGTTTCCTGGGACCTGGCCGAGCTGGAGCACGACGCCGTGCCCGCC
ATGGCCGCGCCCGCCGCGGTGGCCGCGCCGGGCATTGCTGCCGCGGCGCCGCGCGGGCCG
GAGTGCTCGGTCGACCTCAAGCTCGGCGGGCTGGGGGACTTCGGCGCGGCGGCCGACGCG
ATGAAGGAGACCTCGGCGGCGAAGGCCCCGGCGGTGCCGTCGGCGTCGGCGGCGGCGGTG
GTGCCGTCGGCTAGCCCGCTGAAGCGGCCGCGCCCGGGCGGCGGCGGCGGGGGCCAGTGC
CCGTCGTGCGCGGTGGACGGCTGCAAGGCCGACCTGAGCAAGTGCCGCGACTACCACCGG
CGGCACAAGGTCTGCGAGGCGCACTCCAAGACCCCGGTGGTCGTCGTGGCCGGCCGCGAG
ATGCGCTTCTGCCAGCAGTGCAGCAGGTAATAAATACATCCACACGGCCAAACAGCAAAA
TTCTAATTCTGCAGTTCTGTTCCATATCACTGGTCAATTTTCCAACCATACTGCGCAATT
CAGCCCAATTCGCTTTTAAGTTCTGCGCGACCGCAGTCGCATCGATTTGATTGCGATTTG
CGGAAAGACCATTTGCTCCAATTGTTGCAGTGGGAAAAAATAGGACTGATTGGCTTCAGT
CATTTTCCTTTTACTGCAGGTTATTCCCTTTGTTAAAAGTGAGTATAGGCCAGACTAGTT
CTTTCCTTGGCCCTATGGTCCTGGTTTTTCCCTTTCTCTTGGCTCAATTGTTCTTAGTCC
AAGCAGAGCTCAGCAGGGTGAGAATGAGATGAGCATATGTCTTCATGTCTTGTATCAAGA
ATACTCGGCTTTACCTTTTTCTGGCACTAACTTTTCAGCCTTTTCATATGGATGTACCCC
CAAGATTTTACTTTAGCAACATGTATACCTGAATCATAACCCTCTTCTTTTTCTACTTTT
GCACTTGTAACTTTCTAACCCATCGGTGTGAATTATTACCAGTGAAAAGTGACAGATGCA
CTCCATGTGCCAAAGATCAGGTGGAGCAGTCCTTTTTATTTAGCTGCTACATGTTCAAAC
TTTCCCCTGGATAGCCTTCTTTCGGGTTTTTCTTTTCTTTGGTTTGAAATTGAAGAATGG
ACTAGAATTTTTGTATCTTGTAAACATTGTGAAGAAATTGTTCTTGATTCGGCAAATAAA
AGTGCTTATGTGCCAGACTGTAGAATCCCCCCCGACAGCTGAGATTTTGGAGGAAATAGG
GAGTAGATAAGTTAACCTGTCTAACTTATGGTTAACCATATGAATTTTAAGATCAACCCA
TTGTTTTCCCCTTGAGGGGATATGTACCCGGAATGGTAAGGCCTATCTCTGACATGATTG
CCATAATGGTCTTCCCTTGCTGAGTTGGTAATCAGATCTATACGAATTCAAACACTTTTC
TTGTCGATTCTTGAGTGCGGCAGTATTTGTTGTGTGGGACTGTTCATTCTTAAAGATGTT
GCTGAACACCTGACCTGCTTTATTTATTTTTTGGCAGAAACTGGCCTACCACATCTGCCT
TCATTGCTTCGTGTTATTAGCCTTGTTTTTCATATGAGATGCTGGGACCTAGAAAACCAT
TATTTGAAACACCATTTTTTAATACGATCTGCATAATGCATTTTTGCGTAGCCTGTTACC
TTGGACTCTTAGTAACCGATCCGAAAGCCCAACAAGTTTTACTTTTCTTCAGTTAGCTGA
ACTATTAAGTAATTTGCAATTAAGGTTGGCGCACAACAAAATTTACATTTAACTTTCAAA
ATCTGACTCGTGTCTTTTTTTAAAGACTCCGCGAGGACCTTGTTGTTCAATTATTGAACT
GAAAGAAAATAGTTGTTCTTGGTATGTTCATTCCTTTCTAGCAGTACTATTCATTTATTT
TTTCCTCTTGGATTCTTAACTCATAAAGAGGTTGTAATGAATAATATCTGGCTATATGCA
TCCTGTATGCAGAGGCCGGGGTAACATTTTCTAAAAAAGAAGAAACATGTTCATTTTATC
ATCTGAACTCGTCTTAAATCCTATGTTACTAATTTACATCTGATTGATGTTTTGTTATAA
TTTTTATATAGATCTTCTGCTATAATTCTACTCCACTAAAAAAGTGAGATATATTTGTCA
GATATATGGGAATTCGTACCCTCTGAGCAAAGTCGTGCTCGTGCCTCCAATTTGACATGC
TTTTGCTATATATTTAATCTAGGAATTTCATATCGCAGTAGCACTGGAGAAGCTGAGATC
TTATAGGTTTAACTCTAGTTCAGTTGAGTTGGTGTCTTTCGTATTGTTGAGCTCTGATCA
ATTAACTCTCCATCGTGTGCTTAAAGTTCAAACAATACAAATATTATCCATCAATCATTT
GCTCTCATTCTCCAAATTTTATATTCGCGGGGGCACATTTCCAATGATTGCTCACCAAGT
GGAGCAAAATTTGGATCATCATACTGCTTGGCAGACTTTATCGGTGTTCCAGCAGCTGGT
GCCATACTGCCATATACCTGTTGGAACCTATACTGTGGTCCAAATGCCCAACAGGAGTTG
CACTAATTTTCTGTGCTTTACAGGACCAGTATAAAACTTTTATGTAGTGAAGCCCCATGC
ACTGAAATTGTTAACTGACGCATTGAAATCTGAGTGGTCCAGCTGTGTACTATATTTCCT
CAGGGAATCTGTGTGGTATTTTAATAATGATCAAATAAATATTTGGATCATTCTGTTTGG
CTGAGTAGCATGTTAGTCCTTTGTACTAGCTTTTAATTATTCACGTGCCTTAGTGCTAAG
CTCGTACCATTGTTAAGGAAATAATTCATCAAAAGGTTGTGTGTTTTCATGCAAATTCTC

TCTGATCTCATATGCCATACTACAGTCTTGAAATCTTGATCTATATGCTCCATGATATCA
GGCTGTTATGTACTTATCTGTGTTTTGAGCGTGACAAGCAAGAGTTATATATTTCTTATG
TCAAGGTGCAATGTTCAATAGCCAACGGTCTTCAAGTGTGCTCTGATGTTCTGTGTCTCT
TCTTGCTACATATGTTTACTATGCCATTGACAGTGCTAGTATTCCATTAAAGATCGTCGA
TTAGTACACATTGAGAAACGCGCAGCTCTTCATACATGTTTGTATTTTTTTTCCGAATGG
AAGCTTATATTGATCTCAACCGTATTTTTTTCCGTATATGTTTGTATTGCAGCTCTTTTT
TTCACTTCATTGTTTGTGTTTCTTATCATGATTTTTTTTTGTTTCTTTAGGTTTCACTTG
CTTGCGGAGTTTGATGAGGCTAAGCGTAGCTGTAGAAAGCGCCTTGATGGGCACAACCGA
CGCCGCAGGAAGCCTCAGGTAGAAAGCATGAGTTCTGGGAGCTTTATGACAAGCCAACAA
GGTATTATTCTTTTTTCACTACCTTTTTTTGTCAGCCTGCCAGTTTTTACTTGCTGCAGC
TCAAACATACAAACTACGTAGTGATCCCTCTTGCTAGAATTGGAAATTTACGTGATAGAG
TACATTAATGCTAACGCAAACAAATCACAAATGCTCTTGTCACCAGTAGTTGTTCATAAA
ATCTACTCCCTCCGTTTCATAATCTTGTCGAAATCTTACATGTATCTAGACACTTTTTAG
GAATTGATACATCCATTTTTAGGCAAATTTGAGACAAGAATTATGGAACGGAGGGAGTAT
CTGTAAAACTAAGGTTTTGTAACCTAGTCAATACATAGCTACATATTTTCTCCTATGCAC
TATGTAACTGATAATAAGCTTTACCTGTATTTTACATGGATTTAGAGTTGTTTTTCCTCT
GTATCTAATTCTGTGTGTCCACACAAGGCACCGACAACCAATGGCTGTATATACCTTCCT
AATACTCCATCTAGTAGTATCTAATTTGTTGCATACTGACGATGTTGAGTAACATGGTGT
TTTATTCACTAATATTATCAAAGAATCATATTTGAAGATGAAATGTATGGGGTACTCTTC
TTTTTGTAAACATATGAGCTCAACGCAATCTGAATATTCAAACCAGCTGAACGATTGCTC
TATGATGCATTTTCTATCTTTTGCACGGTTAGGTTTGATTAGTACTCCCTCCGTCCCATA
AAAATTGGCACGGAGTTCAAATCCGTGCCAATCTTTGTGGGACGGAGGGAGTAGTATCAT
GCTTGTTTCATGACGGACATTATTTCCAAATGCAGGGACGAGGTTCGCGTCATTTCCTGT
TCCAAGGCCAGAGCCAAGTTGGTCTGGGATGATCAAATCCGAGGACAGCAGTCCATACTA
CACCCATCAGGTCCTCAACAACAACAACAGGCCACATTTCGCCGGCTCCACGTCGACGTA
CTCCAAAGAAGGCCGGCGCTTCCCCTTCCTCCACGAAGGAGACCAAATGAGTTTCAGCAC
AGGCGTCGAGATCCCCGTGTGCCAGCCTCTCCTGAAGTCCGTCGTCGCTCCGCCGCCGCC
CGAGAGCAGCAGCAGCAACAACAACAAGATGTTCTCCGACGGACAGCTGACTCACGCGCT
CGACTCCGATTGTGCTCTCTCTCTTCTGTCATCCCCGGGCAACTCCTCCAGCGTCGACGT
CAGCCGGATGGTCCGTCCAACGGAGCATATACCCCCCGTGTCCCAGCACCTCGTCCCCAA
CCTGCAGTTCGCGAGCTCCTCCTGGTTCGCCTGCTCCCAGGCCTCCAGCGGCGGTGGAGT
CTCTGCCGCCGCAGGGTTCGCCTTCCCCAGCATGGATAGCACGCAGCTCAACACCGCCGG
CCTGGTCCCGAACTCCAACGACCACGAGATGAACTGCCACGGGATCTTCCATGTGGGTGC
CGAGGGCTCCTCGGACGGGACGTCGCCTCCACTCCCTTTCTCATGGCAGCAGTAGTCTTC
AGTAAACATCGGAGAAGCTGTTCTTGGGTTGTTTGATTAGTAGCTAAGCTGTGCCCTTTT
AGTGATCAGGTGTTATCTTCTTTCTGTGTTTATTCCCACCCTTTTTTTTCTCATGTTTAT
CTGTCTTGACTTCTTCTCTAAGCATCAGAGTGAATAACTTTGCATGATTCAGAAGCAAAG
CTCTTTCAAATATGCTTCTGAATTGCATGTCTCTCTTCATGGATGTGGCGTGGGTGCTCA
ACTATTTGAGTCCCGGATCTCGTGCTTGCAACGAGCATCCTCGAGCAAAGTAGTTCTCTC
GTTAATCTCGTGCAGAAACGCAGCATGGTTACGTAAATTCTTCTCGGTCAGGTTTTCTTA
TCACCTCTGGGCAGTCTGAACTCTGAAGTGTTGAGCCGTTGACAGACCCCACGAAGCCTT
GAAGCGGCCGTCGTCGTCCCTGCACACCTCTTCTACTGTCGCAGCAGGCCAGCAGCTGTG
GCCCAGCTCCTATTCTCCCGTTTCGCGACAGAGAGATAAACACAGCTTCCCCGAGTCAAT
TGGAAACCTAGAATTGCAACCTGTGCCCAGTTTAATCCTCACCAACCAAAGCCAAGTGTC
AACAACAGCATTCGCAAATTTTGCAAACATCCGAGGACTCCATACAACTTCTCTTTTCCT
TTTTCTATTTTTGCAACAAGACTCCAAGCAGAAACACGATTTTTTTTCTTTAAGCGATCC
CATGACTATATTCTACCAGAACTGGGGCGATGCCTCGGCCAATCAGGCCCAGAATTGAGG
CGGGCCACTTTGGTCGGACTGTATATTT
>BRADI5G17720
GGATAACGTGGCCCCATTTGTCTGTGTAAGGACGCGCGGCGGTGAGCGGGGTTCAATGCT

GCGGCAGTCATGTCATGGCACGGCACAGAGCCCGGTCCCGCGTCAGATTGAACCCTTCAA
TGACGCCCATCCATGTGGCGTCGGGCCCGCATGTCACGAGCCCCGCAGCGCTCGGGGTCG
TACAGGGTACACAGCCTACAGCTACTACTATTCGCGCCAGCTACCTCTGACGGGAGATCC
CACCTGTCAGTACGGCACTGCCGTGCCTTCCAGTACCCAGTCAGCTCCTCCCTGGGGAGC
TCGCTTCGCCTTCGCCCCTCTCGGGAACGACTTTCCCTATCCTTTATATGCGCGCCTGCG
CCAGTGGGATAGCCTACCGCTGCCTGTCCTTGCAGCAGCAGCCTCCGCCGCCTTTTCCAC
TTTCTAGCTGAGTTTGCCACCACCGCAACGTATTTTCGCGTAATAACACGGAGCATGGAA
GGTGACGGTGGCGCTGGGCACAGAGCGACGGCGCCATGGGATCTCGGGATGCAGTGGGCG
CCTCCTCCCGCCACGTCAGCATACCCGCAGCACTTCATGCCGCCGCCGCCTCCCGCAGCC
ATGGCTAGCGCCCAGCGTCAGCAGCAGGAGCTCACGAGCCTCAAGCTGGGGAAGCGGCCT
TGCTACCTGCCCGGGTGGCGGGACGGCCAGCTGGCGCAGGTGGGGGCGGCGGGCCACGTG
GACGTCAACGGCGGCCGGCGTGCTGTGGCGGCGCCGGAGGGCAAGAGGAAGGAGAAGGCG
GCGGCGGCAACGGCGACGGCGGCCGTGGCGAGGTGCCAGGTGGAAGGGTGCCACCTGGCG
CTGGCGGGGGCCAAGGAGTACCACCGGCGGCACAAGGTGTGCGAGGCGCACTCCAAGGCG
CCCAGGGTCGTCGTGCACGGCGCCGAGCAGCGCTTCTGCCAGCAATGCAGCCGGTCAGTA
AACTTTTGCCCGCACCAAGAGCAGCATCCACTTTCGTTTCGTGCATGTGTAGTTTACTTA
ATTCGTACGTGCAGTGCGCACGGCGTCACAGTTTATATGATAGTACGGAGTATGAGAGTG
GCCGGGTGTGTGTGTAAGTGCAGGTTCCACGCGATGTCCGAGTTCGACGACGCCAAGCGG
AGCTGCCGGCGGCGGCTCGCGGGGCACAACGAGCGGCGGCGGAAGAGCAACGCCAGCGAG
GCCATGGCCAGGGGATCCGCGCACACACACGGTAAACCGTGCCGCTAATCCCCGTTTTCA
TCCATTAGTGCTCTGTTCAAGTTGTTGGACGCCAGGATGAGAGTTAAGTCGAAAAACCAG
TGAGCGAGCGAGAGAGAGAGTATTATTGGATTGAGGCTGTCTGACAGAGTGATAGATACA
GGAGCGAAGTTTGTAAAGCGAGAGAGCGGATAGGTGGTTTGATGCCGGGAAACAAGGAGA
GATCTGCAGGCGCGCGCGTAGGGACATCAAAAGGTTCTAGGGATGCGTGTGAGACGGGCC
AAAGCACTGAGTTTAATGTGTGCATTAATGTGCCCTGCCTGCTTTGAGAGTCCGTGCTAG
TAGCTTAACGTGGGCAGGCCGACCGTCGGACCGTGCATATGATCGCTTTCAACTCGTTTG
GTGACCAATAATTCATTTTATTTGATTAAAATGAAATGAAATTCAAGTTTTGATAGGATT
TTATTTGGTTGAATTTGAATTTCGAGTTAAAAAATTATATTTGTCTACCACATAGAATTG
AAGAGTGAGTGACAATTCTAAAGAAATCATGACTTTTAAAAAGGAATTGAGGTTAATTAT
AGTGTGATTTCATGAAATTTGAGATTGAATTCCTATGACTAATTCCGT
>BRADI5G24670
TCCTCCCTCCATCGATAGGTCGATAGATACATCCTCTCTCTCTCTCTCTCTCTCTCTCTC
TCTCTCTCTCTCTCTCTCTTTCTTCCCTCAGCACAAGAGCGACATCGACATCGATATAAC
CCGGAACGCACTCCACCCTTGTTCCTCCACCACTCGTTCCTTCCTCCCGTTCGTTCGTTC
ATGGAACCCTAGCTAGCCAGCCCCAAAAGTACACCGATCTTACTTACCCCTCCATCAAAA
AGAAGCTGGACAGGGGAGAGAGCTATAGCTACTCATCGAGCTCGCCCTTGTAGTGCTGCT
GGTCGCCCGGGCGCATACATCGTCGTGTGCCCCCTCTCAATCTGTCGTTCATCGCTATCA
TATATACCACCTCCCCCCATGGCACGCGCAATTTGTCCCATGAATAATAGGATCATCAGC
TCCACCCCCCTGTCGAATCCATAGCTTCCCCCAGCTGCCCGTGATCCAATTCATCCATCG
AGGAAGCTAGCCGCGTCCCCGGTGTCCTTGCATCGCGTCCTCGATCTCGAACCCCCTTTC
CAATCCGTGCTCTTACGGCCAAAAAGCTCGGCCGGCCGGGACCGGGATCGATCGAGGACG
ATGATGAACTTGCCAGCCTCCGCGAGCTCCTGCGACGACTTCATCGGCGTCTATGGAGCT
CCTAGCAACAACCCAAGCCCCAATAACCCGCCTCCACAGCAGCCAGCCTCCTCGCTCTTC
CCGCTCATGGATCACCAGGAACAGCATCGAGATCACCACCACCTGGGCTACAACCTGGAG
CCCAATTCACTGGCCCTTCTCCCCCCATCCAACGCTCACCACCACCATGGCGCCACAATC
GCCGCCCACAGCGCGCACGACATCCTCCAGTTCTACCCGACAGGCGCCACGCACCACCAC
TACCTTGCCGCTGCCGCGGCAGGAAATAACCCCTACTCGGGCCACTTCTCTGGCGCCGGC
AGCACCTTCCAGTCCTCCTACTACGGACAGCAGCAGCAGGGGCCGGAGTACAGCTACTTC
CCGGCGCTGGTGAGCTCGGCGGAGGAGAACATGGCGAGCTTCGCGGCCACGCAGCTCGGG
CTCAACCTTGGGTACCGGACTTACTTCCCGCCGAGGGGCCATGGCGGGTACGCCTACGGG

CACCATCCGCCAAGGTGCCAGGCGGAAGGGTGCAAGGCTGATCTTTCTGGAGCTAAGAGG
TACCACCGGCGCCATAAGGTCTGCGAGCACCACTCCAAGGCCCCCGTCGTCGTCACCGCC
GGTGGCCTGCACCAGAGGTTCTGCCAGCAGTGCAGCAGGTTAGTACTACTAATAGTAGAT
TAGCTCTAGCTAAGCAAGATTCTGAATTCGTCGATGCTTCTTTTCTGAAATTAGTTGATT
GATTGGGGTGTGACTTGTGCATGTCTTGTTCTGATCGATTGATTTGTGTTGGGGTAAACA
GAGATGGATCATGGATTGGTCTTGCTGCCTGTAGTTGTAGAAACTGTTCAGTTGGGTAAA
TTTAGTTATCTCCCTTTCTTCCTTGATCAATCATGAGGTGTCATTTAAGTGATCGATGAT
CGATCTGCAAGTTGCATTCAGTTCAGTTCAATAGGTTGGAGCTTGAAACAACCCACTCAT
CCTCCCGGCGTACGCCGGCCTCACAAAATTAGCCTCATTATAAGCTTGGATTTGTCAGGA
ACTTTTGGGATCTTGGTGATTTGATCAAGCTCGATCCCGTAGTTGGAATGATTGACATTT
TGCCACATTTTGTCTGACCCTTTTTAAGCTGGATCTAGCAATAATCGTTCAGCAGTAGAA
CGTTTGATCTAGCTAGATTTCTGTTCCATGGATGGTTGTTTCCTACAGGACTTAACCGTT
GCTTAACTGCTAGCTAGATGGGAACAAAATTATTTCTTCTGAAGCCAAATTTTTGCATGC
TGTTCTCCCACATTAAATTCCCAAGGAAGCACTACTGAGCAAACTACGCACTGTTGTACT
GCAGTGCACGTACAGCTAGCGCTGCAGAGCAAACTCCTCAATTAGTGCAAGTGGAAAATT
TTGCGACGAACAATTTGTCAATTTCAGCCCTTTCGAGGCCGCGTTTCGACAGGTTATCTT
ATGATAATTTCATTTGCTTAATCTGCTTTGGCTTCTTCTATCGTTTGGGTAGTCTACCTT
TTCTAATTTTGCAGCTGTTTTTTCACATCGTCAAAACGCAGTTCAGCATCCCCCAACTGG
CCGGACTATTGAGTTTAATTTAATTTGGGTATGGGTGCCCTATATATATGATAGAATGTA
TCACATGTACGTAGGGAACATACGTATGCAGACATCTCGTCCTCTAGTTGCTTCTTTCCC
CCCTCTCCTTTTGCTTCTTCTCATTGCCATATATTTTGCTTCACTTCTCCTTCTCATGTT
CGATCGAGTAGTTGCTTCAAACAGTGTGGGCTGTCTGTCTGTACATGCAATTCTGCTGGT
GCCCTCCCAAAATCAGACACTGGGCGACTTTCTTGCTAAGTATACTGCAATAAGTTTGTA
CAGCCCATGTACGAACGTACGTATGTACGTCGACGTACGTACCAGCACAGCAGGTTGCCA
TTGCAGACCAGCAAGCATCCCTTCCTCTCTCTCTAGCTAGTAGCTCTAGCTCCTGCTGTC
CTGCATTGCTGCATGTGCCTGCAGGTACGTAGTAGCAATGGGTTTTGAAGATCCATTGCA
GGACATCGTATATACACGGGCGTACGTGTCTACAGGGTTATTTTACCTAGCTGGCTGCTG
TCGATCGTCACACTGAGATCTATATATCTATATATGAATGGCCAGGCCGGCCTCCCGATC
TCTGGTGTGAAGCATACACGTGTGTGAAGTGAGTGACCATCTCTCTAGCAATGCATTGAA
GGGGAAACAGTGGGATCTCTTGCAGTGTGACACACCATATGGAGAGTTGGAGACCACACT
CCATATGAGTCAGCTGCAAAGCTCCAAGCTGTCTGTGGGATCGATTCGATAGGAACAGTT
TGGATATATGCTTCTCATGCATCATGCATGCATGTTCAGGGTCATCATGAGCTTTGCTAC
TGCAATGTTATTGGTCTGGTCCCCCGGCCCGGCCGGCCAGAATGTTCGATCTTTCAGCCC
AGAACTGAACTCTGATCATGCACTAATATGTGTCTCTCTCGCCTAGGATTACCATTTTAA
AGCCACACCATGCATATCTTTGTTACTATTGGGAGACATTAGTATTTCATGCACTAACAT
GACAAGTTTTTTTTTTCTTTTCTTTTGCTGTTCAGATTCCATTTGCTTGATGAGTTCGAC
GACGCCAAGAAGAGCTGCAGGAAGCGCCTCGCGGATCACAACCGGCGTCGGAGGAAGTCA
AAGCCGTCAGATGCCGATGCTGCGGACAAGAAAAGGTCGACACAGGCCAGCAAAACCGCA
AGTACCAAAGGCAGTACGTGAAATACAATATATATATCGTATATATGATCCATTCTCTAT
CTAATTTATCACACCATCGTTTCTGAAATCATGCATTACCAATTTACCATGCATATGCGC
ACATCTAGTAGCTAATAGCTATCGTTAGCTAGCTTTGTGCATAATTACCAGGAAGTTAAT
TAACACTTCTTAACTAAAATCCAACAAAATTCTCAACAGAAGCAGCTGGAAGCAGCAGTA
AGAGCACCGGTACTGGAGACGGGATGGATATACAGGTGGTGGGGGTTGCAGACCTGTCTA
AAGATCAGGATGAAACCATGGGTCTTGGAGAGGTAGTCAAGGAAATGCAGGTGGATCCCA
AAGGAAAAGCATCAATGCAGCAGCAGCAAGGACACCATGGACTTCACCAGCAGCAGCAGA
GCCACCATGGCTTCCATTTTCCTTCGTCCTCTGCGGGCTCCTGCTTCCCTCACCAGAGCC
AAGCTGTGTCGAGCTCTGACAACACATCAAATATCGCTCAAGTGCAAGAACCAAGCCTGG
GGTTCCATCAGCAGCACCACCACCAGCAGCACAACAACATCCTTCAGCTCGGACAGGCCA
TGTTTGATCTTGACTTCGATCACTAGCCACTATATATAATTAGTAATATTTAATGCTGTC
GATCACTCGTACGTGCATGTGTGTACGTTGCAGCATTTCTTTCCATCTTGTCTGTATTAA

TTACTGTAATATGGATATAATAATGAAAGCTTGTGCATTGGCAGCAATCTTCTACTCCCA
TATATAATTGATTAGAGTTGCTTCTATGCTCACAAATTCAAAAGGTTGTCTTGAATTTGC
CACCCGTACTATTGCAGCTATGCGCGCTCTTTCGAGAAGCTCGTTAATGAGTTGGTGTGT
GATCAAATCGTTTGAGTAAGATATGAACCTGTTCAGGTCACCATTGAGGAAATCTGTAGA
TCACCTCCATGTAATGATGTTCTGTCTTCATTGTGGAATAAAAACCCTGGTAGTGAGCGC
GTGATACCGCAATGAGGTATGACACACATACCGGCCATGGCAGCAAAGGTGCACATTTGC
GGAGAGTGTGACCATATATAGCACTCTGCAGATAAATAGGCTGTTATATGTAGTCAATTT
ATGTGGATTACCATGACGATTGACGGGCACGAGAGATTTAATTACATTCGAGTTTACATG
TGGTTCTATTGACCGTCAACAAATAA
>BRADI4G18890
GCGATTGGATTAAGAACGGTTTTCCTTGTAAAGCTAGGGGATAAGAAAATAGTGTTGGTC
AAAAGAGAAACCTGCAAGGCCTTGAAATCACTCATTAGCCATTGCTTTTGCCTGCCTGTC
TCTGCCTGCCAAGATCGATGCATCCTTTTCCGCCCATATAATGCCACACGGCCACACCCC
ATCAAGCACGGAGAGATACTGTCATACTTCGAGCGATCCGTGCCCTGAGCTGCACACACG
CACGCTCTGTGCTCCCTCCCGACCTGCTCGCGCCTCCGCTCGCCACTGTGCTGCCGCTGT
CGTTGCCCCCCGGCGTCTTCCTCCTACAGTATGTGCCGCTTTTCCTGTCCCCTCTTTCTG
ATCTGCGCTTTGCAGCGACATTTTTCAGGACTCAATTTGCAGCGCTGAGCTGTTCTCGCC
CTGGGAGTGCATGATTTTTCAGCTTTCTTTAGGACCTGTTTTACTGCTTGATTTCGTGCA
AGGATTTCGCGGGATATTTCTTGCACAAATTTTGCCCCAAGATTTTCTAAGTCTTCGCGC
TTTCTTGATTGGAAACAGAGCGGAGGCTCGCGGCGAATCGTGAGATTTTTCTTGGCCCGC
ATGGACTGGGCCGCAGCTCCCGAAGCGGCGTCTTGGGGCGTAGCAGCGGAGGCGGTTGCC
GACCCTGGTCCGACCATGCTGTCCTTCGCCAGTCCGTCGTTCTCCTCCTCTGCCGCCGTG
GCGGCGCAGCTGCAAGACCGCTCTAGGCCAGCGTCGGTAACAGCGAGGAGGGACCGCGCG
CCTGGCAGCGCTGCCGGCACGGTGGCATGCTCGGTGGATGGGTGCCGGTCGGACCTCAGC
CGGTGCCGCGAGTACCACCGGCGGCACAAGGTCTGCGAGGCGCACTCCAAGACGCCGGTC
GTCGTCGTCGCGGGCCAGGAGAAGCGGTTTTGCCAGCAATGCAGCAGGTTAGAAACCATC
CAACCCTTACACCAAGTGATCTCTAGTTACTCCGCTACACACGAAATGTTTGTAGATGAG
GTCATTTGTTAATAATTGTGTGCGCTTGGTCCCCGTTTGGTTGCTAATTGGGGAATTTAG
GGGTTACTTTTTAGGGCATCGTCAGGATCTGATGGAAAAGATTTAGGTCCGGTTTGGTCC
CCAGCTAAATCACTATCTGACTAAACAATTGAGTTGGTAATTTCGTGGCATTTGGGATCT
GATGGCACGGATTGTTTAATCTCTCCAATGCGATATGTCATAATTAAACAATTGGGTTGG
TAAATTTGGTAGCTTTAGGGTCTGATCACACAGATTATTTAATCTTGTGGTTGCTATTTA
AAGCAAGTTGTTGGTCTAGACAACTGACTTCTACTGAGTAGGGTATAGTTTTTCTGTGGG
ATGTTCAGCATTGTGCAGAATCTTTCCTATGATGAAGAGTTTCTGCATCTTCATGCTCAG
AATGGAATAAAATGACAGGCTTCTGTAAAAACTTGTGTGATTGTGTTCCTGAGAAACTGA
CTAGGAAACTCTTCCAGTCAACAAGATCTGATTGAAACAGTTAAGGTTTATTGCGTAATT
TTTGCTGGTCCCCAAACTTAGCATAAAGATATTTCATATGTTACTGTTTGGAGTTGAACC
ACATGTATTTGAAACTGAAAAATTAGGTTATACCTTGGTTTGCTTGATTGTGTTTCCCTT
GAGTTTAAGACGTATTCGATCCCATTTTCCTACTCTCTTACTCTGTACTCTGTTATGCTA
TTTTGTACTTACCTGACAGGACAAGAAAATAAGGAACTACATACATTTTGTGTAGAATTA
TTTGGTTTCTGCAAACCTTTGGGAAATTTTTTAGTGACTTAAAGGAGGTTATCTTACAGA
ATGTATTACAAATTAAAGAAAAAGTACAAACAGATTCCTTCGAGGAGCAAACTCTACTAA
TTTGACTGCATATTGATTGAGAGTTACAATTAAAAAAGTCCAGCATAACATGCACTATCT
GATTTTTACCTTGGTTTCAGAAATAACTCTGAAATATTCGTTAACTATGTTCTCCCAGGA
ATAAATATGTGATTCTAATGCTCTTAACGCCATATTATTTTTCAACCTCTCAAATTCTCA
GTTCGAGAATGACATAAAATGATAAGTACAATTATGTTGCAAAAGAGAAAACTTGTACAT
TTGGTTTTACAACTGCAAGCTTATTGAACAATGCAAGATGTAGAAAGAAGCGCCCTAAGC
TTTTATTGCTGTTGTCTCCAATGCATGTACATATATTCTTCAAAAATCATGCCAAAAACA
GTACTGTACTACTTAGTAATCTGTCATGAACTAGGGTTCATGAGGGTAAATACTGCAATC
TTGAACATTGTTGGCTGCTCTTGAACATTAAGCAATACAACAGGAGCTTGTCTGCAATCT

TGAACATTGTTGAAAAATAAATACTGTACTACTTAGTAATCTGTCATGAACCTGATGTAT
TCATTTTTTAGGATTGGCAAGTGAATGTTGTACTACAAATAGGAGCTTAATTGAGTTTTG
ACCATTATGCACTACCTTGTTTTCCCTATTTCTGTTCTCCTAAGAAGATGAAAATGCACC
ATGCTTCGCCTTTGTTTAATAAAATTGGTAATTTATAAGTGCAATTGTTTTAAAAAACAC
CTAGAGGTATATTACATGCTACAAAATTTGCACTTGCAAAAATCAAGCCAAAATATGACT
ATTTGCACGTATAGTATATGCTCTTCTTTTTTTATCTTTTTAGATTTTTGTATATTCATA
TGGAAGCATTTTATAAGTGTGAGGAAATATGCAACTTGTATTTCCTGTAGGCCAAGGGCC
AGTATATGTACCCGTACAAACGGGTAAAGATTCAGGAAACCCCTTATACAATGGGGAAAA
TACACAACAAATACATAATGCTGTACACAAGTCTAAAAACCCCCCTCAAACTCATAGTCT
ATGCTTCACAGCAAGATTCTTACCGCTGAAAATTTGGAAGCTATTGGTTGGCATCATAAT
GTAATGTGCAAGCTCTGCAACTCTCAACTGGAAACCATCAAACCTTTGCAAGGATTGTGC
CTACACCAAGTCTGTTTGGGCGAT
>BRADI4G18900
CCAGAATGTCACAATTGAAAAGATTAATAAAGTAAGGAATACACAGTTTAGCAATCTGAC
AAGAAACCCAGTCACAGAAGAATTCATTTGGAGATCACGGCCAGAGAATTTCAACACAAT
CACAAAAAAGAAACTATTTGCTTCCATAATCCTCAAATTTCCTTGCACCCAATTCTTATT
CCATATCTAGCATTATTACCCACTGCCCTTGCCCTTCGTTTGTTCTTATCCTGATGTTGG
CCTTAACTATGGCATTGCACGTGAAAGGCATGCATGCATAAGGCAGGATAAACTCTTGCT
TCCGTTGGTTTATATACTAGAGTTCCATCCCTATCAAACAAGCTACTTTAATTATTTAGT
TCAAGTTTAATCTTCCATCAGGTTTAAAACTGGGCAGATAACATATGTGCCTGCAAATTC
TTAATAAGAGAGTGGCGCACAGCGTAGATCAGGTTGTCAAACATAACAGCCTTGGGATAC
ACTTTTGCTTTGTTCAAGGAGCATTTCTTCTATCAGTGTAGGCTGTAGCTGCCCGGTCCA
TCTGAAAGGAGATATTGATGTCATGTGTGTAGTTATCAAAGCCGCAAAAAAATTGACACT
ATGGTGATTACTTCAAGGTTTGTTAAATGATTGGATTGAACTGTAAAAACAAATTAAAAA
GGCTGTGAAAACAAATTGAAGTTGAAATTCGTACTACCAGTTTAAAACACTTGTTCTGAA
AAATGTTGAGTGGCCATGTAATATTATCTACTTGCAGGTTTCACATGCTGGCCGAGTTTG
ACGAGGGGAAGAGAAGTTGCAGAAAACGCCTAGATGGCCATAACAGGCGTAGAAGAAAGC
CACAGTATGATGCAATGAACCCCAGCAGTTTCTTTCCATATCACCAAGGTTTGTATGTTT
GAAACATTGGTAATTTGGTGACCCTATTTACTAATTCTGTCTGTATTCTGTGTTGCAATT
TATGTATGCAGTCATTATAATTGTTGACTCTTTGTTGACACTCCCTCCGCCCAGAAAAAT
AAGGCACACGGTCTTTCTGAAATTTATATTTCACCAAGAATGGGTTCAGTAGTGTGGATT
ACGTGATACGAAATTAATATTTGTCGGATTCGTATTAAAAGCACTTTTCGATGGTATCAC
ATTGGTTAATGTTAAATCTTGGGAAATGTGCGCACACGGAGGGAGTAACAAACAATATCC
AGTTTTGGGTAAAAAACAAACTGCATCCAGCAGATTGCCCACTTATTTTGTTAGGGGAAA
CTGTATGTATTTCTTCACTTAGTCTTGTCTTTTTCATGTTCTTTTGATAGTGCCTTTCTT
CTTGAAGTGTTTTCACAATCAATACACAGGTTTTCTGATATAACATTGTCCTGTCTTACA
GTGAATAAATTTTCTGTCTACCCGCGAACATTTCCTATAGCAGATCCAAATGCGGGGGCC
TCAATGCACCCGCTCGAGCATCATCGACCCTTCTCCATCTCATTCTCGGGAACCACCAAA
GCACCGAAGCACTTCCCATTCTTGCAGGATGGCAGCAGCATCTACAGTACGGCCAGCCCT
ACTCTTCCGCAGCCCTTCTCTTCTCGTGAAGATGGCAACAACACCAGCAGCAGCACATGC
AATGGTCTCTCCAGCGCACTGGACCCTGAGTGTGCTCTCTCTCTTCTGTCATCCTCGCTG
CACCTCTCCCCCATCAGCATTCCCAGTGCAACAGTTCCAGCGCAGTTTGCTTCTTCGCTT
GCTAGGACTGCTGTTGACTCGCAAGCTGCAACCATTGCATTTGCATCAGGTGGAGATTGT
GGTGGTGGCGGCGGCCATGTGTTGGTTGCTGATGCTATGCTTGAAGACCCTTCACAGGGA
CTGCCCTTCTGCTGGCAGGTGTAGATCAAGGTTGTGATTTGCTCTGTTTCCCTTATGCTG
CTGAGTGCTCAGTTGCAAGGCTTTTTGGTGCTTGTGCACCTGTAGTATCAAGTTGATGTA
AATTGTACACGATTAACAATACAAGTCCTCTCTGGTTGATGGCTGTTGTGCAGATTTGTC
GTGTCTTCATTTTTTGGGAGGATGATCTGCATATTGTTTAGCTATTTTGGTTTGTTTATA
GTACTTGTGTAAATTGTAATGACCTTGGTGAGCAAAAACAAAAAGGCCAATAAGCAGAGT
ATGGCTCTCCTCGCAAAAAAAAAAAGGCTCCCAAACCAGGAAATTGACCCCCTGCTGCTT

GTACTGCAGATTGTGAAGCAGGATGCTAAGAATTAGAATAACGGCAGTTGTTTGCCACCC
CACATGGGTGATGGTTTGCTTTGGGCTCAGTTTGGCATCGTTGAACTGTACTGCGTTGAA
CTTATTTTTTAATCTGCTGTGAAAAAATATACACGAAAGTAGGAAAAATTTGGTTAGCCA
AACACGAAAATAGTGAAAAGCGGGTGAGAAAAACGGGATTATGAGAATCCACCGCAATAC
CAAACGCAGCTTAGAGTGACTCGTAGGTACGTTTGGCCTTGGCTGTAACTGTTCTGTTTC
ATCGCAACGCCTGTTTGTTTTGCTTTTGACAACCGCTGCGATCGGAAAATGGTTAGGTTC
GCTTGTTGGGTCATTTGGGACGAATCGAGAGGTCACAGAATAGTACAACATAGAATTCTA
AATTCTTCCACTCACTAGCTACCATCCAACACAGAAGAAATAAAATTGAATGCATAAAAA
TATGGTGTATGAATGATGGTAAGGCTGCCCTCTTTTGGCAGGCATTCAAAGAAAGACCGG
GTAAATGAGAGGGG

Table S2. Coding DNA sequence of BdSBP genes.

>BRADI1G02760 cds:protein_coding
ATGGAGGCGGCCGGGGTCGGGTCGCAGAGCCGCCGGCTGTACGGCGGCGGGTTAGGTGAG
CCTGCCCAGGACATGCGTGGGAAGAGGCTGTTTGGCTGGGACCTCAATGACTGGAGCTGG
GACAGCGAGCGCTTTGTCGCCACCCCGGCGCCTGCAGCAGAAAAGGCAAATGGCCTGTCA
CTGAATAGTTCGCCGTCTTCCTCTGAGGAAGCGGACGTCGAGGTGGCTAGGAGTGGTAAC
GTGAGAGGTGATTCTGATAAGAGGAAGCGGGTGGTGGTCATTGATGATGACGGTGATGAC
CAGAAGGATGAGGACCCTGTGGATAACAATGGCAGGGTGCTCAGCTTGAGAATTGGGGGA
GACACTACTGTTGCCGGGGGAGCGGTGGAGGGTGGTGCCGTTAATGAGGAGGATAGAAAT
GGCAAGAAGATCAGGGTGCAGGGGGGAAGCTCAAGTGGCCCAGCTTGTCAGGTGGAGGGC
TGCTGCGCGGACCTTAGTGCGGCGAAGGATTACCATCGCCGGCACAAGGTCTGCGAGATG
CATGCTAAGGCCAACACTGCGGTGGTCGGAAATACTGTCCAGCGCTTCTGCCAGCAATGC
AGTAGATTTCACCTTCTTCAAGAATTTGATGAAGGAAAGCGAAGCTGTCGTCGGCGTTTA
GCAGGTCATAATAGACGGAGGAGGAAAACCCGCCCTGAAATTGCTGTTGGTGGGACTCCT
ATTGAGGATAAAGTTGGCAGTTATTTAGTGTTGAGTCTTCTTGGAATATGCGCCAATTTG
AACTCTGAGAATGCTGAGCATTTACAAGGTCAGGAGTTGCTATCCAATCTTTGGAGAAAC
CTGGGGACTGTTGCTAAATCATTGGATCCAAAAGAACTCTGTAAACTCCTGGAGACATGT
CAGAGCATGCAAAATGGATCAAATACTGGGACCTCTGAAGCAGCTAACGCTTTGGTGAAT
TCTGCTGCAGTAGAGGCTGCAGGACCATCTAATTCTAAGGCGCCTTTTACGAATGGTGGT
CAACGTGAGCAAACATCATCTGCTGTTATACCGCTACAGTCAAATGCTACCGTGGTGGCG
ACTCCTGAGACTCCAGCATGCAGGATTAGGAATTTTGATTTAAATGACACTTGTAATGAT
ATGGAAGGCTTTGAGGATGGTTCTAATTGCCCATCTGTACAGCAAGATTCTACTCAAAGC
CCACCACAGACTAGTGGTAATTCAGATTCAACGTCAGCTCAGTCATTGTCAAGCTCAAAT
GGAGATGCTCAGTGTCGGACTGATAAAATTGTATTCAAGCTTTTTGACAAAGTTCCTAGT
GATTTACCTCCAATTTTGCGATCACAGATTCTTGGTTGGTTGTCCAGTAGCCCTACTGAT
ATAGAGAGCTATATTAGACCTGGCTGTATTATCCTGACAGTATATCTTCGATTAGTTGAC
TCTGCATGGAGAGAGCTCTCTGAGAATATGAGCTTATACCTGGATAAGCTTTTAAGTAGT
TCCACTGATAACTTTTGGGCATCTAGTTTGGTATTTGTGATGGTACGGCATCAAATTGTT
TTTATGCACAATGGTCAAGTTATGTTGGACAGACCACTGGCACCTAATTCTCATCATTAC
TGCAAGGTTTTATGTGTTAGTCCAGTTGCTGCTCCTTCTTCAGCGACAGTTAATTTCAGG
GTGGAAGGCTTTAACCTAGTCAGTGCTTCCTCGAGGCTAATTTGTTCATTTGAAGGGCGA
TGCATATTCCAGGAAGATACAGCTATTGTGGATGATGCTGCTGAGCATGAAGATATTGAG
TGTCTCAACATTTGTTGTTCCCTCCCTGGTTCAAGAGGAAGAGGATTCATAGAGGTTGAA
GATAGTGGATTTAGTAATGGATTCTTCCCCTTCATAGTTGCTGAGCAGGATGTATGCTCG
GAGGTTTGTGAGCTGGAGAGCATATTTAAGTCATCCAGTCATGAACAGGCAGACAATGAC
AATGCCAGGAGTCAAGCTTTAGAGTTTCTAAATGAGCTGGGGTGGCTTCTTCATAGAGCA
AACATAATTTCTAAGCATGATAAAGTGGAGCTGCCTCTAGCTGCATTTAACCTGCTGAGA
TTTAGAAACCTTGGTATATTCGCCATGGAGCGGGAATGGTGTGCTGTGACCAAAGTGCTG
TTAGATTTATTATTTGATGGATTTGTTGATGTTGGGTTGCAGTCACCGAAAGAGGTGGTA
TTATCAGAAAATTTGCTGCACACTGCTGTGCGAGGGAAATCTGTCCGAATGGTTAGATTT
CTGCTGAGATACAAGCCAAGTAAAGACCAGAAGGAAATTGCAGAGTCATACCTATTCCGA
CCTGATGCTCGGGGCCCTTCTACATTTACACCCCTCCATATAGCAGCTGCTACTAGTGAT
GCAGAGGATGTATTGGATGCACTGACTAGTGACCCTGGACTGGTTGGACTCAACGCGTGG
AAAAATGCGCGAGATGAGACAGGCTTCACCCCTGAAGATTACGCTCGCCAGAGAGGCAAT
GATGCATACATGGATCTGGTCCAGAAAAAGATTGATAAGAATCTTGGCGAAGGTCATGTT
GTCCTTGGTGTTCCCAGCAGCATGTGCCCTGTACTAACTGATGGGGCGAAGCCTGGTGAT
ATTAGCCTCGAGATCTGCAAAAGCATGCCAATGGCTCCACAACCTGTGTCGAGGTGCAAC
ATCTGCAGTCGTCAGGCTCGGATGTACCCCAGTTCCTTTGCAAATACTTTCCTGTACAGG
CCAGCAATGTTCACCGTGATGGGCGTTGCCGTGATCTGCGTCTGTGTCGGCATACTCCTC

CATACCCTCCCCAAGGTTTATGCAGCACCAAATTTCAGATGGGAACTGTTAGAGCGTGGA
CCAATGTGA
>BRADI1G26720 cds:protein_coding
ATGGACCGGAAGGACAAGTCCCGGAAGTCCTCCTCCTCGGCGGCGTCCATGGCCGCGCTC
GCCGCCGCCGGAGGAGACAGGATGGCGCCGCCTTCTTCCGGCGATGAGGACCAGAAGCCG
AACCTGGTGAACGTGCCCGTGGTCGCCACCGGCGCCTCGAGCTCCTCTGCTGCCGCGGTG
AGGAGGGGCGGCGGCGGCGGTGCTGCTGGTGGGCCCGTCGCGGTGGGCGGGGCCGGCGCG
GGCGGGCCCAGCTGCCAGGCCGAGAGGTGCCCCGCCGACCTCACCGAGGCGAAGCGGTAC
CACCGGAGGCACAAGGTGTGCGAGGCGCACGCCAAGGCCGCCGTCGTGCTCGTCGCCGGC
CTCCGCCAGCGCTTCTGCCAGCAATGCAGCCGGTTCCACGAACTTCTGGAGTTCGACGAC
ACCAAGCGCAGCTGCCGCCGGCGCTTGGCCGGTCACAACGAGCGCCGGAGGAAGAGCTCG
GCGGACGCCAATGGTGGGGATGGCTGCCGCCACGTGGACCAGGACGGCCGGAGCAACCCG
GGGAACCCGCCGCCGCTGAACCACTTCCAGATCAGATAA
>BRADI1G31390 cds:protein_coding
ATGATGAGCGGCAGGATGAACAACTCCACGGGGAGCGACGACTTCCCCTTCGCCCCAACG
CCGCCGCCATCCTACGGCGGCTTCGAGCAGCGAGCACTGTACGACAGCTTCGACTTCGCC
GCCGCCTTCCAGTTCCAGCACCAGCAAGAACACCACCAAATGCTCTCACTCCCCCCCAAC
GCCAACACAAGCAACCTACTCCACCACCCCATGGCTCCTCCCCCACCCCCAGCAGCCATG
TCAATGCAGCTCCCAATCCCCATGCCCCAAATGCACGGCCACGGCGGAGACGCCATGATT
TACCCAGCGCTGGGGATGGCCGTGAAGCGGGAGGGAGAAGTGGCCGAGGGAAGGAACATC
GGGCTGAACCTGGGCCGGCGGACCTACTTCTCCCCCGGGGACATGATGGCCGTGGACCGG
CTGCTGATGCGGTCCAGGCTGGGCGGGGTGTTCGGGCTGGGCTTCGGTGGGCCGGGGGGC
CATGGGCACCACCACCAGCCGCCCCGGTGCCAGGCGGAGGGATGCAAGGCGGATCTCTCC
GGCGCCAAGCACTACCACCGGCGCCACAAGGTCTGCGAGTACCACGCCAAGGCCTCCCTC
GTCTCCGCCGGCGGCAAGCACCAGCGCTTCTGCCAGCAATGCAGCAGGTTCCACGTGCTC
ACTGAGTTCGACGAGGCCAAGAGGAGCTGCCGGAAGCGGCTGGCAGAGCACAACCGTCGC
CGGAGGAAGCCGGCGACGACCAATGGCACGGCGACATCGGCGGCCAAGGACTCGGCGACG
CCACCTTCTTCCAAGAAACCCAACAACGGCGCCGGTGGTGCCATCATCGGTTCCTACACT
GTCGACAACAAGAGTAAGACCTACACCTTATTTTGCTGCAGCTTTGAGCGCCGCCAAGTC
GTCGACCATCTCCTCCAACACCAGCGGCATCAGCTGCCTGCAGCAGCAGCAGCAGGACCA
AAGCAAGGCGGCGGCAGCGGCGGCGCTCACCCTCGGCGGATCGCCGCAGGATCAGAGCAA
CGCCGTGCACCAGCTCGCCGGCCACGGCCACGGCCACCATCATCAGGAGCAGCACTTCAT
CACCTCACTCTTGCACAACAACAACAACAACAACAACGGCAACAACAACAACATCCTGTC
GTGCTCCTCGGTGTGCTCCAACGCAATGCCGCCGCCTGCGACAGCGAACAACGGCGGTGG
CGAGGTCTCCGACCAGAACAACCACCACGGCAACAACAACAGCAACAACATGCATCTGTT
CGAGGTAATTAA
>BRADI2G11240 cds:protein_coding
ATGGAAGCCGGTTTCCGGCGAGAAGGCGGCCAAACTGGTTTGGCTAATACAATGTCTAGT
GAGCTCAGCAAGAACAAGAAGAAGAAGGGCCTCGAGTGGGATTTGAACGACTGGAGATGG
GATGGCAACCTGTTCCTGGCCACACCGTCGTCGAATGCCGTCGCGGCGCCATCGGGTTGT
GGCAGCAGGGAGCTGGGCCGATCTGAGGATGGGATAGATTTGAGTGTTGCTGACAAGAGG
AGGAGAGTTTCACCAGTGGATAACCATGGGGAGTGCAGCAATGCTGCGATTGCAAATGGA
GATCATGACAGGGTTGTTGGTCGGAGAGGGCAGAGCAGCAGAGAAGGGAGACATGCAAAT
GCAACAGGTGCATATTCCACTTCTGCTCCATATTGCCAAGTTGACGGCTGCCACGCGGAT
CTCCGTGACAGCAGGGACTACCATAAGAGGCACAAGGTGTGCGAAGTACATACCAAGTCC
ACTGTGGTTCGTATAAAAAGCATAGAGCATCGGTTTTGTCAGCAGTGCAGCAGGTTTCAT
CTTCTTCCAGAATTTGATGAAGGGAAGAAGAGCTGTCGCTCACGTCTAGCAAAACATAAT
GGTAGGAGGAGGAAAGCCCCAGCCCAGGCTGGTGCGGCTGGGAATACCTCAAGTGAAAAT
CAGTCTTTAACCAACACCTTACTCCTCTTGTTGAAACAACTTTCAGGGCAAGATCCTGGA
AGCTCATCTGAGCAAAGCAATGGTCCCAACTTTTTGGTTAATCTTTTGAAGAACCTTGCT

GCCATTGCTGGCACACAGGCGTATCAAGATATGCTTAAGAATGCAAATTCTGCTTCAATA
TCATCAAATGATGCTAACTACGTTGTAAATGGCTTCACAAACGAGCCAACCAGATCACCA
ATTCCTGTAGGAACTGAATCCTCAGCAGAGCATACAGTGAAACAGCATGTGCAGAATTTT
GATCTGAATGATGCTTACGTTGAAGAAGATGAGAGCCGAACAGATAAAATTGTCTTCAAG
CTCTTTGGGAAAGAACCAAAGGATTTTCCTGTTGATCTACGTGCTCAGATGCTAAACTGG
TTGTCACATTACCCAAGTGATATGGAAAGCCATATTAGGCCTGGTTGTGTCATTCTAACT
ATTTACCTTCGTCTTCCTAATTGGATGTGGGATAAGCTTAAAGTCAATCCAGCTCCTTGG
ATAGAAAACCTTATTAGCATATCCACTGATGGCTTCTGGGAAACAGGATGGTTGTATACT
AGGTTACAGGACCGCCTGGCATTGAGTTGCAATGGTAGGCTTATGTTAGTGTCTCCCTGG
CAACCCTTAATAGGTGACAAGCATCAGATATTATGTGTAACTCCGATTGCAACTGCTTGT
TCTTCAACAGCAAACTTCTCAGTGAAAGGTTTCAACATAGTTCAACCAACCACAAAGTTG
AAGATTATGACCAAAGCAGCCTTTCCTTTCCCATTGTTGTCGCTGAAGAATCTTTATGTT
CTGAGATTCGAATGGTGTTCTGTTGTGAAGAAGCTTCTGGATACATTGTTCCAGGGTAAT
ATTGATGTAGATGTCCGCTCACCATTTGAATTTGTCCTAGGAGAAGGTTTAGTATTCACC
GCGGTCAACAAGCGGGCGAAGCGTTTGGTTGAGTTCCTATTACGATACACAACAAATTCT
GCACTTGTGGCCCGTGGAGCCGTGTCACCAGTTCGGTTCTTGTTCACACCTGATATAACT
GGTTCATCAAATATTACACCTCTTCATATTGCAGCCAGTATGAGTGATGGTGCTGGTGTT
TTAGATGCTTTAACTGATGATCCTCAACAGCTTGGAATCAAAGCATGGAAGCATGCTCGT
GACACTACTGGGTACACTCCAGAGGATTATGCTCAGAAGAGAGGTCACATATCCTACATC
CAGATGGTCGAGAACAAAATCAACAGCAGGTTGCCTAAAGCTCATGTGTCTGTTTCCATG
ACTATTAGTCCATCCACCACAGATATCTCTGAAAAGCACGCAGGTCGATCAAAGTCTACG
AATCAAACCACATTGGATATTGAGAAAAGCCAAAGGAGCGACAAGAGACCACCAAGCTGC
AGACAGTGCGTCCAGCTCCAGCACATTGCTTACCATCCCCGTCCGAACAGGTTTCTGTCG
AACAGGCCTGCGGTGCTCTCCTTGGTCGCCATTGGTGCAGTCTGTGTCTGCGTAGGATTG
ATCATGCAGAGCCCGCCGACTGTCGGCGTTGGCATGACGGGTCCTTTCCTCTGGAGTTCC
CTGAATTACGGTCCCATCTGA
>BRADI2G25580 cds:protein_coding
ATGGACGCCTCCGATTCAGGCGGGGCCAGCGCAGCGCCGGACGCCGGCGAGCCAGATTGG
GACTGGAACCACATCCTCGAATTCGCCGTCCGGGGAGACGACTCCCTAATCCTCCCGTGG
GATGACACCCTCGGCACCGCCGAGGCTGGTCCCGCCGAAGGGGCGTTTCTCCCTGCCCCG
TCTCCGGCTCTGCCAGTGGAGGCGGAGCCGGTGGCGCCGCCGCCACCTGTTGAGGCTGGA
GGAAGCAGGTCCGGCGTGAGGAAGCGGGACCCGCGGCTGGTGTGCCCGAACTACCTCGCC
GGGATTGTGCCGTGCGCGTGCCCCGAGTTGGACGAGATGGCGGCTGCTGCGGAGGCCGAG
GAAGTTGCCTCGGAGATGCTGGCTGGCCCGAGGAAGAAGTCGAGGCCTGCCAGCCGGGGC
AACGGAGTGGCAGCCGGAGGTGGTGGTGGTGGTAGTGGAGTAGCTGGCCGTGGGGGAGCA
GTAGAAATGAAGTGCCAAGTCCCAGGTTGCGAGGCAGATATTCGGGAGCTGAAAGGGTAC
CACAAGCGGCACCGGGTGTGTTTGCGTTGCGCGCACGCTACGGCTGTCATGCTCGATGGC
GTCCAGCAGCGCTACTGCCAGCAGTGCGGCAAGTTCCATGTTTTACTTGATTTTGATGAA
GACAAAAGGAGTTGTAGAAGAAAGCTGGAGCGGCACAACAAAAGAAGACGAAGAAAACCT
GATTCAAAAGGAGCATTTGAGAAAGAGGTAGATGAACAATTGGATTTGTCAGCAGATGGT
AGCGGTGGCTGTGAACTAAGAGAAGAGAATACAGATGGAACTACCTGCGAGATGGTTGAG
ACTGTCCTTAGTAATAAGGTTTTGGACAGAGAAACACCTGTGGGGTCTGAAGATGTGCTA
AGTGCTCCAACCTGTACACAGCCCAGCTTGCAAAATGAACAAAGTAAAAGCGTAGTGACT
TTTGCAGCTTCTGTTGAAGGCTGCCTTGGTACAGAACAGGAAAATGCCAATATTACCAAT
TCTTCGATGCATGACACCAAGAGTGTTTATTCATCCTCGTGTCCCACAGGACGCATTTCA
TTCAAGTTGTATGACTGGAATCCCGCAGAATTTCCTCGACGTCTACGTAACCAAATATTT
GAGTGGCTGTCTAGTATGCCGGTAGAATTGGAGGGCTACATTCGTCCTGGATGTACAATT
TTAACTGTGTTTATTGCAATGCCACAACATATGTGGGACCAGTTATCAGAGGATGCAGCA
AATCTTGTCAGAGACTTGGTAAATGCTCCGAGTAGTCTTCTGTTGGGTAAAGGGGCTTTC
TTCGTGCATGTCAATAACATGATATTTCAAGTATTGAAAGATGGGGCCACATTGATGAGT

ACCAGATTGGAGGTACAAGCCCCCAGGATCCATTATGTTCATCCAACATGGTTTGAAGCA
GGAAAGCCTGTTGAGCTCCTCCTCTGTGGAAGTTCCCTTGACCATCCCAAATTCAGATCA
CTTTTGTCATTTGATGGGGAGTACTTGAAGCATGATTGTTGCCGTTTAACGTCTCACGAG
ACCATTGCTTGCGTAAAAAATGCCGCTGCACTTGATTCTCAACATGAAATTTTCCGGATA
AACATCACTCAAACAAAGGCGGATACTCATGGACCTGGGTTTGTGGAAGTGGAAAACATG
ATTGGGCTATCAAATTTTGTCCCTGTCCTTTTCGGTAGCAAACAGTTGTGTTCTGAACTA
GAGAGGATACAAGATGCTCTATGCGGTTCTAATGAAAAGTACAAGAGTGTATTTGGAGAG
GTTCCTGGTGCCACTTCTGACCTGTGCGGGCGTCTGGAACTTAAACAAACTGCAATGTCG
GGATTCCTAATAGAGATTGGATGGCTAATTAGGAAGTCTTCTCCAGATGAACTAAAAAAT
TTACTGAGTTCAGCAAATATCAAGAGATGGACATCTGTGTTGAAGTTCTTGATACAGAAC
GATTTTATCAATGTTCTGGAAATAATTGTCAAGTCTTCAGACAACATCATAGGCTCTGAG
ATTCTTTCTAACTTGGAAAGAGGGAGGTTAGAACATCATGTCACGACATTTCTTGGATAT
GTACGGCATGCTCGAAACATTGTTGAAGACAGAGCTAAATATGACAAGCAAACACAGCTT
GAAACAAGGTGGTGTGGTGATAGTGCTTCAAACCAGCCAAACTTGGGCACTTCTGTCCCA
TTCGCTAAAGAAAATACTGGTGATGGCAGTGAATATGATTTGCACCCAACCAATGTTGAG
TGTAAAGAGGAAGAAAGGATGCTACTTGTGAGTCCTAAAGCTGTCTCACACAGGCAATGC
TGCAGCCCGGAGATGAATGCTAGATGGCTTAATCCCACCTTGGGTGCACCTTTTCCAGGT
GGTGCCATGAGAACGCGACTTGTCAAGACTGTGGTAGTGGCTGCTGTATTGTGCTTCGCT
GCTTGTGTTGTCGTTTTCCACCCAGATAGAGTAGGGGTGCTTGCAGCGCCAGTAAAGAGG
TTCTTATTTAGCGACTCCCCATCTAGTTGA
>BRADI2G59110 cds:protein_coding
ATGGATTGGGAGGCCAAGATGCCTTCATGGGACCTGGGCACGGTGGTGGGGCCAAGCGGC
GGCGGGGGCGGGGGGCTGGACCTAAAGCTCGGGGGCCCGACGAGCTGGAGGCCGGTCCCG
GCGGCGACAGCGGCGCCGGTGGCGCCGTCGTCTTCTCCGACGCCGGCGAAGCGGGCGCGG
GCGGGGGCGCCGGCGCCGGCGTGCTCGGTGGAGGGCTGCGCCGCCGACCTGTCGCGGTGC
CGCGAGTACCACCGGCGGCACAAGGTGTGCGAGGCGCACTCCAAGACCCCCGTCGTCTCC
GTCGCCGGCCAGCAGCAGCGCTTCTGCCAGCAGTGCAGCAGGTTCCATCTGCTCGTTGAG
TTCGACGAGGTGAAGAGGAGCTGCAGGAAGCGGCTCGACGGCCACAACAGGCGCCGGAGG
AAGCAGCAGCCGGACCCCCTCGGCCCTGCCGGCCTCTTCGCTAATCACCACGGAGGAGGA
ACAAGATTTGTGTCGTACCCGCAAATCTTCTCCACGACGACATCCATGGCGCCGGAGCAC
AAGTGGCCTGGCAGCATCGCCGTCAAGACGGAAGCCGACGCGTTCCAGGTCGAGCAGTAC
TACTCGTCACTCCAACTCAACGGTGCCGCCAACGCCGCCGCCTCGCTCTTCCATGGCAAG
GAGAGATCGAAGCGCTTCCCCTTCCTGTCCGATCACCATGGCGACACGGGATTCCAGCAG
CAGCCATTTACCATCACACCTTCCTCAGAGAGCAGCAGCAACAGCAGCAGCAGGCACAGC
AACGGCAACAAGATGTTTGCCACCAACGACGGGGGCCCGGATCACAACTGTGCTCTCTCT
CTTCTGTCAGACAGCCCGACGACACAGGCGGCGCACACCATGATCACCGCAGCGGCGCAG
CACCTCGGTGGCGCAAGGATACATTACAACGTTGGAGGCGATGGTGGCGGCGGTGTCTCG
CTCACTGGGCTGTCTTACGCCAGTGCCATCATGGGAGACAACAAGGGCAGCGCACAGGCC
GCCATGGCCTCATCTACCAGGCACGCTGGAGCCGTTACTACAGCCCCCTCTGTTGCTGCC
CAGCTACAGCAATACCATGGCTACTACCAGCACCAGGTGAATGGTGATCAGGGGAACTCA
GCTGATGCTGGTGCCTCCATGCAGGCCCTCCCTTTCTCATCATGGTAG
>BRADI3G03510 cds:protein_coding
ATGGGCTCATTTGGGATGGACTGGAATCAGAAGAGCTCAGTATTGTGGGATTGGGAGAAT
TTACTGCCGGCAGGCACTAATGCGGCTGAGAACCCCAAGAATGGAATGCAGGCTGAGCAA
AGGTTTGCAAGTGTAGTGGCTGCCATGGCTAATGAATCACGACATTCTTCCGGTAGCAGC
GGTACTTTCTCTTCCAACTCGGAGATGGGATATGGTTCATCCAAGAGCTCCCTATCCGCT
TCAATTGATTCTTCATCCAAGGTAGGGAACAGCATGGAATTCAGATTTGCAGCTGCCGCA
AACCCTGATAGCAACAACAGCAAAAATACTGAGTTGGGTAAAGTTGATGACACCGGAGCC
GGAACATCTCCCTCATCGGTGATAGCAGTGAGCAGTGGAGAGCCAGTCATTGGCCTGAAA
CTTGGCAAGAGAACTTACTTTGAAGATGCCTGCGGAGCACAGAATGTCAAGAACTCACCA

TTGGGTGCGGGTGCACCAAACCCATCTCCTGCTTCGGTCAAGAAGGCAAAGGTGGATCAA
CAGAAGCCACATAATTCATACTGTCAAGTTGAAGGCTGCAGAGTTGATCTCTCTTCTGCT
AAAGATTACCATCGAAAGCACAGAGTCTGTGAAGTTCATTCTAAGACTCCCAAAGTTGTC
GTCGCTGGTCTGGAGCGACGCTTTTGCCAACAGTGTAGCAGGTTTCATGCTTTAGCTGAG
TTTGACCAGATAAAGCGAAGCTGCCGTAGGCGTCTCAACGATCATAATCACCGCAGACGG
AAGCCACAGCCAGAAGCAATTTCTTTCAGTACATCAAGGCTGTCTACGATGTTTTATGAT
GCAAGGCAACAGACAAGTCTTCTATTCAGTGAGGCTCCGTATGTTCAAATGAGAAACTGT
GCAAGTTCTTCATGGGATGACCCAGGACCAGGAGGCTTCAAGTTCACAGAAACAAAAGCT
CCTTGGTTAAAGCCAACAACTGCTGCAGGTGTTGATGCGATGCATTTGTCTAGCCAGCAG
GTATCGAACAGTATTATGCCACACGGTGCACATCATGGTTTTGATGGGTTCATGTCATTC
AAGGGAACTGGTATGAAGTTCCTTAATCAAGGCGTCGAAGCTTCCACTGTTGTTTCCGAC
ACCAGTGGAGCCCCAGACCTTCAGCATGCTCTCTCTCTTCTGTCAAACAACCCAGCAGGT
GCTGGCAATCTCCAGCCAAGTCCCCAGATGCATTCTGGAACTACTGCAGACATCGCCGGC
ACTTCAAACCCTGCGATGCATGTGCTGGGCTCGTCGCCAGGGCTCTGGCTAGACGGCCCG
CCCCTCGACAATCATCACCCACGGTTTCAGGCTTTTGATCTCTTGGGGGGCCACGACAGC
ACCATGCCGCACGAGCTCCAGCTCCCAAAGCCTTCATATGACCACCACGCCGCCTCCCAC
TTCGACCGGATGCACTGA
>BRADI3G05510 cds:protein_coding
ATGGAGTGGACGGCCCCGAAGCCCGCCACGTCCCCCCCGACCCACCTCCTCTGGGACTGG
GGCGACTCCGCCGCGCCGGGCTCCTCCGGCGACGCGGCGGGGAGGCGCGGGAAGGAGAAG
CGGGCGAAGGGGGAGGAGGGCGGCGGCGGCGGCGGAGGAGGAGGAGGAGCGGTGGTGAGG
TGCCAGGTGGAGGGCTGCGGGGTGGAGCTCCGCGCCGCCAAGGATTACCACCGGAAGCAC
CGCGTCTGCGAGGCCCACACCAAGTGCCCCCGCGTCGTCGTCGCCGGCCAGGAGCGCCGC
TTCTGCCAGCAGTGCAGCCGGTTCCATGCCCTGTCGGAGTTTGATGAGAAGAAGAGGAGC
TGCCGGAGGCGACTGTCTGATCACAACGCTCGACGACGAAAGCAGCAGCCAGATGCATTC
TCCTTCGCACCTGCAAGGCTGCCATCAACGTTGATATTTGATGATAGACGACAAATAAGT
TTTGTCTGGAATAAAGGTCCACCTAGCCATGTAAGGCCTTTCGCATGTTCTCCATGGGAA
AGCCCATCTGAATTCAAGCTCTCACAAGTGAAGAACACGAGAGGAGTGTCAACCAATGGA
CAAGTTCATCTGGACAAATCTCATCTATCGAATGCTGTTCCAACACTGAGTCATGACATG
GATGAGCTGTTCCCAGTAAAAGGTCCCGATGCATCTTTAGCTGCTTCAAAATTAGATGGA
GCACCGGATCTTCAGCATGCTCTCTCTCTTCTGTCAGCTAGTTCTCGTGGATTACCTGAT
CCTGCACAGCAAGCATCTTGTCTTGTCCAATTCTCCGGTGCCAGCCAAAACAGCCGGGGC
CTTCATTCATCACATGGAGTGAGCTCTGCGTCGGCACCCTGTGCTGAAGGACAGCCTATG
GCGCCGTCGCCTCACCTCGTCCGTTTTACCGTGGATGGCACCAGCAGTGGCTATGACACC
ACATTCTTCGGTCTAAACAAGATAAATTAA
>BRADI3G05720 cds:protein_coding
ATGAGCAGCAGGCAGCAGCTGAGCCCCGGCACTGGTACAATGCCACCCATCTCCGGCGAC
GCTGACTTCGGCAGCTGCTACACCAGTACTCATCATCCCTACGCCGCCTTCGACGGCAGT
CCCAGCGCCGCCGTCGACCACCGCCCGCCGCTGCTCCACCACCACCACCAGCAGCTCTAC
GACACCACCGGCCTCGACTACGCCGCATTATTCCCCTTCGCCCCCCAGCAAGATAATAAC
CCCCCCGCTCATCTCTTCCCCAACCAGCTGCCGCCCTTCACCGCCAACAGCACCACGATG
CTTCTCCAGCCGCCGATGCTGACGCCACTCCCCGGCCTGCCGACGTCATCCCCGCCGCCG
GCCCCCGGGGACGCGTACCAGCTGCACCACCCCTTCGGCGGCTTCCAGCTGAAGCGGGAG
AACGAGGGCGGCCTCTTCCCCTTTTCCGACGCCATGGCCGCCTCCGGCGTTAGCGGCGTT
GGCGGAGGCAGCGGAGGGAGGATCGGGCTCAACCTGGGCCGCAGGACCTACTTCTCGCCG
GCGGACGTGCTGGCCGTGGACCGGCTGCTGATGCGGACACGCGGGGGACTGGGCGGCGGC
GGCATGGGGGTTCTGGGGCTGGGGCTGGGAGGCGGCATTATGCAGCAGCCGCCGCGGTGC
CAGGCGGAAGGGTGCAAGGCGGACCTGTCGGCGGCCAAGCACTACCACCGCCGCCACAAG
GTCTGCGAGTACCACGCCAAGGCCGCCGCCGTCGCCGCCAACGGCAAGCAGCAGCGATTC
TGCCAGCAATGCAGCCGGTTTCACGTGCTTGCGGAGTTTGACGAGGCCAAGAGGAGCTGC

CGGAAGCGGCTCACGGAGCACAACCGCCGCCGCCGGAAGCCCGTCGGTGTTCAGGGCAAG
GACTCGCCCCCGCCGCCGCCTTCCAAGAAATTAGAAGCCGGCATCACCACCAGCTCATAC
GCCGGGGATCACACTACCACCAACAAGTCATCATCGACGGCGGCAGCGGCGGTACTCTCG
CCGAGCGCCAGCGCCTTCAGCTGTCTGCAGCAGCAGGAGGAGGAGCTGGACGACAACAAC
GAAGGCAGCGGCGGGCGACCGACGACGCTGTCGCTCGCGGCGCCGCCGCAGAGGGACTAC
GGCGGCGCCCTGGACACCATGCTTCTGATGCATCATCATCATCATCAGGCAGCAGCGGCC
GTCGTGCAAGACGACGACCAGGAGCAGGACTTCATGATGACCTCTCTCGTGCAATCCCAT
CATCATCATCAGCAGCAGCAGCAGCAAGGCGAAGTCAGTGGCAACATCCTGTCGTGCTCG
CCGACGGCGTCGGATCAGCGTCGTCAGAATCACCAGCAGATGCAGATCAACGACGGCGGC
GACGACAGCGGCTGCTGCAATAACAGCGGCATGCAGCAGCAGCAGCATTTCTTCGAGGTG
GACTTCATGTAG
>BRADI3G40030 cds:protein_coding
ATGGAGATTGGCAGCGGCGGTGGCGGAGGGAGCGCCGTTGCCGGAGGTGGCGATGGCGGC
GGCGGAGGGGACGACCAGCTCCGGCACGGGCTCCAGTTCGGCAAGAAGATCTACTTCGAG
GACTCCTCCAGTGGCGGGAGCAGCAGCGGCGGCGGCGGTGCGAATGCGGCGTCGTCGTCT
TCGAAGCCGGCGGCAAGCGGCGGCGGGAAGAAGGGCAAGGGCTCGGCGGCGCCGCCGCGG
TGCCAGGTGGAAGGGTGCGAAGTGGATCTCACGGCCTCCAAGGGCTACTACTGCCGCCAC
AAGGTGTGCTCCATGCACGCCAAGTCGCCCCGCGTCGTCGTCGCCGGCCTCGAGCAGCGC
TTCTGCCAGCAGTGCAGCAGGTTCCACCAGTTGCCTGAATTTGATCAAGGAAAACGCAGC
TGCCGCAGACGCCTCGCAGGCCACAATGAGCGCCGCAGGAGGCCACCCGCTGGCCCTCTT
GCGTCACGCTACGGCCGGCTTGCTGCATCCTTTGAAGAATCCGGCAGGTTCAGGAGCTAT
CTGCTGGATTTCTCATACCCACGTGTTCCGAGCAGTGTGAGGGATGCGTGGCCAGCTGTC
CGACCAGGTTACCGGATGCCCAGCGAAGTTCAGTGGCAAGGGAACTTAGAGCCTCGTGCT
CAGTCAGGTGCAGCCATGGGTTACGGCGGCCATGCATACAGCAGCCATGGCTTCCCCAGC
CCAGGGCTCCCTCCAGGCGGGTGTCTTGCAGGGGTCGCTGCCGACTCCAGCTGTGCTCTC
TCTCTTCTGTCAACTCAGCCATGGGATACTACCACCCACGGTGCCAGCCACGACCACCGG
TCCGCGGCCATGTCTGCCGCTGCCGCGAGTTTTGATGGCAACCCTGTGGCGGTGGCCCCG
TCCGTCATGGCGGGTAACTACCTGCCGCCACCGGCGAACCCTTGGAGTGGCTCCAGGGGC
CATGAAGGTGGGCGGAACGTACCTCCTGACCCTCAGCTGCCACATGACGTCCCGCTCCAT
GAGGTGCACCCTGCAGGCTCTAGCCAGCAGGGCCACTTCTCAGGTGAGCTCGAGCTCGCT
CTGCAGGGTAACAGGCCTGCAGCACCTGGACCACGCTATGGCGCCGGCCGCAGCACATTC
GACCACCCTGGCAGCTCGACGAACTGGTCTCAGTAG
>BRADI3G40240 cds:protein_coding
ATGCAGAGGGAGGTGGGCCCGCAGGTGGCCTCCCCGCTGTACCTGCACCACCAGATCCAG
CCGCTGCCTCCCCATGCGGTGGCGGCGGCGCCCAAAAAGCGCGGGAACCCGTGGCCCGCC
TCCGCGGAAGGCGCGGCGGCGGGGTCTGCCGGCGCGGGGAACTGGAACCCCGCGATGTGG
GACTGGGACAGCCGCGCCTTCACCGCCAGGCCCTCCTCCGACGCGCTACGCCTCGGGGCC
GGCGCCCAGAATCATCATCATCATAACCACCACCAGCAGCAGCAGCGGCAACCGGCGGCG
ATGGCTGCTGAGGCGCAGCAGCGGCAGGGCCCCGGTGGGCTGAGTCTTCAGCTGGCCACG
CGGGAGGAGGCGTCGGTGGCGATGGATGTCAGCCCAACGGCTATCATGTCTTCCTCGCCT
TCTCCGCCAGCAGCGCCGGCGCATGAGCAGGCCGCTCGGCCTAGCAAGAAGGTCCGGTCT
GAATCGCCGGGGACCGGTTCTGGAGGCGGCGGCAACGGAGGTGGAGGAAGCAGCGGCAAC
GGAGGTGGGAGCTACCCGATGTGCCAGGTGGACGACTGCCGCGCGGATCTGACGAGCGCC
AAGGACTACCACCGGAGGCACAAGGTCTGCGAGATCCACAGCAAGACCACCAAGGCGGTG
GTTGGCCACCAGATGCAACGCTTCTGCCAGCAGTGTAGTAGATTTCACCCCCTCTCGGAG
TTCGATGAGGGTAAGAGGAGCTGCAGGCGAAGGCTCGCCGGGCACAACCGGCGACGGAGA
AAAACCCAGCCCACAGATGTTGCTTCACAGTTGCTGCTACCTGATAACCAAGAAAATGCC
GGAAATAGGACACAAGATATTGTCAATCTGATCACGGTTATTGCGCGCTTGCAAGGTGGT
AATGTTGGTAAACTACCTAGCATCCCTCCTATTCCAGATAAAGATAATCTGGTCCAAATT
ATTAGTAAAATAAACTCAATAAATACGGCAAATGCTCTGGGAAAGTCTCCTCCATCGGAA

GTCATTGATTTGAATGCCTCCCACGGGCAGCAACAGGATGCTGTTCAGAAGGCAACAAAT
GTAATTGACAAGCAAGCTGTGCCATCAACCATGGACTTGCTAACAGTTCTATCAGGTGGC
AACGGTGCTTCTACCCCTGAAACCAATACGTCCCAGTCCCAAGGGAGCAGTGACAGCAGT
GGTAATAACAAGAGCAAGAGTCATTCAACGGAGCCAGCTTATGTTGTAAATTCCCATGAG
AAATCAATCCGAGCTTTTCCTGCAGCTGGTGTGATAAGGAGCAACAGCCCCCATGACAGT
CCACCTGAAATGTACAAGCAGCCAGACCGAGATGCCCGCCCATTCTTGTCACTGCAGTTG
TTTGGTAGCACCTATGATGATATCCCTGCTAAGATGGATACCGCAAATAAGTACTTGTCA
TCTGAGAGCAGTAATCCTATGGACGAGAGATCTCCATCATCCTCTCCACCTGTAACCCAC
ACATTTTTCCCCATCCGTTCAGCAAACGATGGCATCACGCATCCTCGTGCTGGAGACTAT
GGAGAAGATGCTGCAACAGTCGAGAATAGTACCACTCGGGCATGGTGTGCACCACCACTT
GAACTTTTCAAAGATTCAGAGCGGCCAACAGAAAATGGGTCACCACCAAACCTCACATAT
CAATCATGTTATGCCTCAACTTCTGGTTCTGACCATTCACCATCAACATCGAATTCAGAT
GGACAGGACCGTACTGGTAGGATTATATTTAAGCTTTTTGGCAAGGAACCTGGCTCAATC
CCTGGGAACCTTCGTGATGAGGTTGTAAATTGGCTCAAACACAGCCCCACTGAAATGGAG
GGTTACATTCGCCCTGGTTGCCTTGTACTATCCATGTATTTGTCTATGCCAGCTATTGCA
TGGGATGAACTTGAAGAGAATCTTCTCCACCGGGTAAACACATTAATTCAGGGTTCTGAT
TCTGATTTCTGGAGAAACGGAAGGTTTTTAGTTCGAAGTGACAATCAGTTGGTGTCATAC
AAAGATGGAACGACTCGCTTATCGAAATCATGGAGGACATGGAATACCCCTGAGTTGACC
CTTGTGACACCAATTGCTGTTGTTGGTGGGAGAAAGAGCTCCCTCATTCTTAAGGGCCGT
AATCTAACGATTCCTGGCACCCAGATCCACTGTACCACTGAAGGGAAGTATATATCAAAA
GAGGTACTATGCTCGGCGTATCCAGGTACCATATATGATGATTCAGGTGTCGAGACCTTT
AATTTACCGGGAGAACCAAATCTCATTCTTGGGCGTTGCTTTATTGAGGTCGAAAACAGG
TTCAGAGGAAACAGCTTCCCTGTTATATTTGCCAATTCAAGCATTTGTCAGGAGTTGAGG
AACCTTGAAGCTGAGCTTGAAGATTCGCGATTTCCTGATGTCTCTTCAGAAGATCAGGTT
GATGATACTAGGCGGTTAAAGCCAAGGGATCAAGTTCTGCATTTTCTTAATGAACTTGGC
TGGCTCTTCCAGAAGGCTGCTGCTTGTATACCCTCCACCAAATCCGATGTTTCTGATTCG
GAGTTGATTCAATTCTCAACTGCACGATTCAGATACCTTTTGTTGTTTTCAAATGAGCGG
GACTGGTGTTCTCTTACAAAAACACTACTCGATATTCTTTCCAAGAGAAGTTTGGTCAGT
GACGAACTATCACAGGAGACTCTGGAGATGCTCTCAGAGATTCATCTCCTGAACAGAGCA
GTGAAAAGGAAGAGCCGCCGCATGGTGCATTTGCTCGTGCAGTTTGTCGTAATTTGCCCT
GATAATTCCAAACTGTATCCCTTCCTTCCCAATTATCCTGGCCCAGGTGGTTTGACTCCA
TTACATCTTGCTGCATCCATTGATGATGCAGAGGGTGTAGTTGATGCCTTGACAGATGAT
CCTCAACAGATTGGTTTGAACTGCTGGCACTCAGTGTTGGATGATGATGGCCAATCTCCT
GAAGCCTATGCGAAGTTCAGGAACAACGATTCGTACAATGAACTCGTGGCACAAAAGCTT
GTGGACAAGAAGAATAGCCAGGTTACCATTGTACTCAACAAAGGAGAAATTTGTATGGAT
CAACCTGGGAATGGTGGAGGGAATAATGCATCTGGGATCCAAGCAATGGGGATAAAATCT
TGCAGCCAGTGTGCCATTTTGGAGTCTGGCTTGTTAAGCCGCCCTATGCATTCAAGGGGA
TTGCTTGCACGCCCTTATATCCACTCGATGCTTGCCATAGCAGCAGTTTGTGTCTGTGTC
TGTGTATTCATGCGAGCCTTGCTGCGGTTTAATTCTGGCAGGTCCTTCAAGTGGGAGAGG
CTGGATTTCGGTACATCCTAG
>BRADI3G41250 cds:protein_coding
ATGGACTGGGATCTCAAGATGCCCGCGGCCGGCGCCGGCGCCGCGTGGGACCTCGCCGAG
CTGGAGCAAGGCGGCGGAGGCGGCGGGCCGTCGGCGGATGGCATTGCGGCTCCTGCTGCT
GCTGCGGGCGGTGGTGGACGGGCCGAGTGCTCCGTGGACCTGAAGCTCGGCGGGCTGGGC
GAGTCCGGCGGCGGCCAGGCCCAGGCCCAGGCCCAGGACAGCAGCACCCGCGGGGGGAAG
GCGCCGGTCGCGGCGTCGGCGCCGGCGCCGGGGAAGAGGCCGCGCGCGGCGGCGGGGTCG
TCGTCGAGCAGCGGCGGCGGCGGCGGCGGCGGGGGGCAGGGGCAGCAGTGCCCGTCGTGC
GCGGTGGACGGGTGCAGGGCGGACCTGAGCCGGTGCCGCGACTACCACCGGCGTCACAAG
GTCTGCGAGGCGCACTCCAAGACCCCCGTCGTCGCCGTCGCCGGCCGCGACATGCGCTTC
TGCCAGCAGTGCAGCAGGTTTCACTTGCTTACAGAGTTTGATGAGACCAAGCGTAGCTGT

AGAAAACGTCTTGATGGGCACAACCGTCGCCGCAGGAAGCCACAGCCAGATCCCATGAAT
TCTGCAAGTTTTATGACAAGTCAACAAGGAACAAGGTTTTCACCATTTCCAAATCCAAGA
CCGGAGCAAAGCTGGCCAGGGATCATCAAAACTGAGGAGAGCCCATATTACGCGCATCAA
ATCCCTATAGGTATCAGCAACAGGCAGCATTTTGGTGGATCTACATCTACTTACGCCAAA
GAAGGCCGGCGCTTTCCTTTCCTACAGGAAGGCGAAATAAACTTCGCCACAGGGGTGACC
CTTGAGCCTTCAGTGTGCCAACCACACCCGAGGACGGCAGCTCCTCCCGATAGCAGCGGC
AGCAGCAAGATGTTCTCTGATGGGCTGACTCCTGTGCTCGACTCAGATTGTGCTCTCTCT
CTTCTGTCAGCTCCAGCAAACTCCTCTGGTATTGATGTCGGCCGGATGGTCCAAGTCCAA
CAGAACGAACACATCCCCATTGCTCAGCCTCTAGTCTCCAGCCTACAGTTCAGCAGCTCG
TCCTGGTTTGCGCGCTCCCAGGCTGCCACCGGTGCCGTCCCACCGACCGGATTTTCCTGC
CCTGTTGTGGAAAACGAGCAGCTCAACACTGTCCTGAGCTCGGATAACAATGAGATGAAC
TACAGCGGGATATTCCATGTCGGCGGCGAAGGCTCCTCAGACGGCGCCCCGCCATCTCTA
CCCTTCACCTGGCAGTAG
>BRADI4G33770 cds:protein_coding
ATGGAGACCGGCAGCAGCGGCGGCGGCGGCGGTGGGGGAGGCGACGACTTCCACGGGCTC
AAGTTCGGCCAGAAGATCTACTTCGAGCAGGACGTGGCCAGCGGCAGCGGGGCGACGACT
TCCGCGGCGGGCGGCGGCGCTGGGGCCGGAGGAGAAGGAGCAGCAGCAGCAGCCTCTGCC
GCCGCTGCGGCCGGCAACGGCGCCGCGCACGCTCAGGCGCAGCCGCCGAGGTGCCAGGTG
GAAGGGTGCGGCGTGGATCTGAGCGGCGACAAGACCTACTACTGCCGCCACAAGGTGTGC
TCCATGCACTCCAAGGCGCCGCTCGTCGTCGTCGCCGGCATCCAGCAGCGCTTCTGCCAA
CAGTGCAGCAGGTTCCACCAGTTACCTGAATTCGACCAAGGAAAACGAAGTTGCCGCAGG
CGCCTAGCGGGTCACAATGAGCGCCGGAGGAAGCCCCCACCTGGTCCTCTGTCTTCGCGC
TATGGCCGGCTTGCCGCGTCCTTCCATGAAGATCCCGGCAGGTTCAGAAGCTTTCTGCTG
GATTTCTCGTACCCAAGGGTTCCAAGCAGCGTGAGGGATGCGTGGCCAACGGCTCATCCC
GGCGAACACCACCGGATGCCCGGCACGGCAGCCCAGTGGCAAGGGAGCCATGAACTCCAT
CATCCTCACCGCAGCACAGTTGCCGGATACGGCGTCGACCACCACGCTTACGCCGGCCAG
GGTAGCTCGTCGGGTGGCGCCGCGCCGATGTACCCAAGCTTCGAGCTTCCCGCGGCCGAA
TGTATCGCAGGAGTCACGGCCGCCGACTCCAGCTGTGCTCTCTCTCTTCTGTCAACTCAG
CCATGGGATCATAGTGCCCACAGCGCCAGCCACAACCGGCCCCCGGCAATGTCGACGGCC
AGCGCCTTCCAGGTCAGCCCGGTGGCGCCATCCGTCATGGCCAGCGACTACATGGCGGCG
GCAAGCAACAGCGCCTGGGCTAGCACCCGGGGCAGGAACATGCAGCAGCACCAGCAGCAG
CAGCATCACCATCATCATGACGCTGTGATGAGCGATGTCCATCCAGGCTCGGTTCACCAG
CATGGCGGTCAGTTCGGGGAGCTCGAGCTCGCGCTGCAGCAGGGGAGGGCCGGGGCGCCG
AACACGCCGCACGCCGAGCATGGGTCTTCCGGCGCCGGCGCCTTTGGCCACCACTCCAGC
AACGCCATGAACTGGTCTCTGTAG
>BRADI4G34667 cds:protein_coding
ATGGCCGCGCCCGCCGCGGTGGCCGCGCCGGGCATTGCTGCCGCGGCGCCGCGCGGGCCG
GAGTGCTCGGTCGACCTCAAGCTCGGCGGGCTGGGGGACTTCGGCGCGGCGGCCGACGCG
ATGAAGGAGACCTCGGCGGCGAAGGCCCCGGCGGTGCCGTCGGCGTCGGCGGCGGCGGTG
GTGCCGTCGGCTAGCCCGCTGAAGCGGCCGCGCCCGGGCGGCGGCGGCGGGGGCCAGTGC
CCGTCGTGCGCGGTGGACGGCTGCAAGGCCGACCTGAGCAAGTGCCGCGACTACCACCGG
CGGCACAAGGTCTGCGAGGCGCACTCCAAGACCCCGGTGGTCGTCGTGGCCGGCCGCGAG
ATGCGCTTCTGCCAGCAGTGCAGCAGGTTTCACTTGCTTGCGGAGTTTGATGAGGCTAAG
CGTAGCTGTAGAAAGCGCCTTGATGGGCACAACCGACGCCGCAGGAAGCCTCAGGTAGAA
AGCATGAGTTCTGGGAGCTTTATGACAAGCCAACAAGGGACGAGGTTCGCGTCATTTCCT
GTTCCAAGGCCAGAGCCAAGTTGGTCTGGGATGATCAAATCCGAGGACAGCAGTCCATAC
TACACCCATCAGGTCCTCAACAACAACAACAGGCCACATTTCGCCGGCTCCACGTCGACG
TACTCCAAAGAAGGCCGGCGCTTCCCCTTCCTCCACGAAGGAGACCAAATGAGTTTCAGC
ACAGGCGTCGAGATCCCCGTGTGCCAGCCTCTCCTGAAGTCCGTCGTCGCTCCGCCGCCG
CCCGAGAGCAGCAGCAGCAACAACAACAAGATGTTCTCCGACGGACAGCTGACTCACGCG

CTCGACTCCGATTGTGCTCTCTCTCTTCTGTCATCCCCGGGCAACTCCTCCAGCGTCGAC
GTCAGCCGGATGGTCCGTCCAACGGAGCATATACCCCCCGTGTCCCAGCACCTCGTCCCC
AACCTGCAGTTCGCGAGCTCCTCCTGGTTCGCCTGCTCCCAGGCCTCCAGCGGCGGTGGA
GTCTCTGCCGCCGCAGGGTTCGCCTTCCCCAGCATGGATAGCACGCAGCTCAACACCGCC
GGCCTGGTCCCGAACTCCAACGACCACGAGATGAACTGCCACGGGATCTTCCATGTGGGT
GCCGAGGGCTCCTCGGACGGGACGTCGCCTCCACTCCCTTTCTCATGGCAGCAGTAG
>BRADI5G17720 cds:protein_coding
ATGGCTAGCGCCCAGCGTCAGCAGCAGGAGCTCACGAGCCTCAAGCTGGGGAAGCGGCCT
TGCTACCTGCCCGGGTGGCGGGACGGCCAGCTGGCGCAGGTGGGGGCGGCGGGCCACGTG
GACGTCAACGGCGGCCGGCGTGCTGTGGCGGCGCCGGAGGGCAAGAGGAAGGAGAAGGCG
GCGGCGGCAACGGCGACGGCGGCCGTGGCGAGGTGCCAGGTGGAAGGGTGCCACCTGGCG
CTGGCGGGGGCCAAGGAGTACCACCGGCGGCACAAGGTGTGCGAGGCGCACTCCAAGGCG
CCCAGGGTCGTCGTGCACGGCGCCGAGCAGCGCTTCTGCCAGCAATGCAGCCGTGCGCAC
GGCGTCACAGTTTATATGATAGTACGGAGTATGAGAGTGGCCGGGTGTGTGTGTAAGTGC
AGGTTCCACGCGATGTCCGAGTTCGACGACGCCAAGCGGAGCTGCCGGCGGCGGCTCGCG
GGGCACAACGAGCGGCGGCGGAAGAGCAACGCCAGCGAGGCCATGGCCAGGGGATCCGCG
CACACACACGGTAAACCGTGCCGCTAA
>BRADI5G24670 cds:protein_coding
ATGATGAACTTGCCAGCCTCCGCGAGCTCCTGCGACGACTTCATCGGCGTCTATGGAGCT
CCTAGCAACAACCCAAGCCCCAATAACCCGCCTCCACAGCAGCCAGCCTCCTCGCTCTTC
CCGCTCATGGATCACCAGGAACAGCATCGAGATCACCACCACCTGGGCTACAACCTGGAG
CCCAATTCACTGGCCCTTCTCCCCCCATCCAACGCTCACCACCACCATGGCGCCACAATC
GCCGCCCACAGCGCGCACGACATCCTCCAGTTCTACCCGACAGGCGCCACGCACCACCAC
TACCTTGCCGCTGCCGCGGCAGGAAATAACCCCTACTCGGGCCACTTCTCTGGCGCCGGC
AGCACCTTCCAGTCCTCCTACTACGGACAGCAGCAGCAGGGGCCGGAGTACAGCTACTTC
CCGGCGCTGGTGAGCTCGGCGGAGGAGAACATGGCGAGCTTCGCGGCCACGCAGCTCGGG
CTCAACCTTGGGTACCGGACTTACTTCCCGCCGAGGGGCCATGGCGGGTACGCCTACGGG
CACCATCCGCCAAGGTGCCAGGCGGAAGGGTGCAAGGCTGATCTTTCTGGAGCTAAGAGG
TACCACCGGCGCCATAAGGTCTGCGAGCACCACTCCAAGGCCCCCGTCGTCGTCACCGCC
GGTGGCCTGCACCAGAGGTTCTGCCAGCAGTGCAGCAGATTCCATTTGCTTGATGAGTTC
GACGACGCCAAGAAGAGCTGCAGGAAGCGCCTCGCGGATCACAACCGGCGTCGGAGGAAG
TCAAAGCCGTCAGATGCCGATGCTGCGGACAAGAAAAGGTCGACACAGGCCAGCAAAACC
GCAAGTACCAAAGGCAAAGCAGCTGGAAGCAGCAGTAAGAGCACCGGTACTGGAGACGGG
ATGGATATACAGGTGGTGGGGGTTGCAGACCTGTCTAAAGATCAGGATGAAACCATGGGT
CTTGGAGAGGTAGTCAAGGAAATGCAGGTGGATCCCAAAGGAAAAGCATCAATGCAGCAG
CAGCAAGGACACCATGGACTTCACCAGCAGCAGCAGAGCCACCATGGCTTCCATTTTCCT
TCGTCCTCTGCGGGCTCCTGCTTCCCTCACCAGAGCCAAGCTGTGTCGAGCTCTGACAAC
ACATCAAATATCGCTCAAGTGCAAGAACCAAGCCTGGGGTTCCATCAGCAGCACCACCAC
CAGCAGCACAACAACATCCTTCAGCTCGGACAGGCCATGTTTGATCTTGACTTCGATCAC
TAG
>BRADI4G18890 cds:protein_coding
ATGGACTGGGCCGCAGCTCCCGAAGCGGCGTCTTGGGGCGTAGCAGCGGAGGCGGTTGCC
GACCCTGGTCCGACCATGCTGTCCTTCGCCAGTCCGTCGTTCTCCTCCTCTGCCGCCGTG
GCGGCGCAGCTGCAAGACCGCTCTAGGCCAGCGTCGGTAACAGCGAGGAGGGACCGCGCG
CCTGGCAGCGCTGCCGGCACGGTGGCATGCTCGGTGGATGGGTGCCGGTCGGACCTCAGC
CGGTGCCGCGAGTACCACCGGCGGCACAAGGTCTGCGAGGCGCACTCCAAGACGCCGGTC
GTCGTCGTCGCGGGCCAGGAGAAGCGGTTTTGCCAGCAATGCAGCAGGATTGGCAAGTGA
>BRADI4G18900 cds:protein_coding
ATGGTGATTACTTCAAGGTTTCACATGCTGGCCGAGTTTGACGAGGGGAAGAGAAGTTGC
AGAAAACGCCTAGATGGCCATAACAGGCGTAGAAGAAAGCCACAGTATGATGCAATGAAC

CCCAGCAGTTTCTTTCCATATCACCAAGTGAATAAATTTTCTGTCTACCCGCGAACATTT
CCTATAGCAGATCCAAATGCGGGGGCCTCAATGCACCCGCTCGAGCATCATCGACCCTTC
TCCATCTCATTCTCGGGAACCACCAAAGCACCGAAGCACTTCCCATTCTTGCAGGATGGC
AGCAGCATCTACAGTACGGCCAGCCCTACTCTTCCGCAGCCCTTCTCTTCTCGTGAAGAT
GGCAACAACACCAGCAGCAGCACATGCAATGGTCTCTCCAGCGCACTGGACCCTGAGTGT
GCTCTCTCTCTTCTGTCATCCTCGCTGCACCTCTCCCCCATCAGCATTCCCAGTGCAACA
GTTCCAGCGCAGTTTGCTTCTTCGCTTGCTAGGACTGCTGTTGACTCGCAAGCTGCAACC
ATTGCATTTGCATCAGGTGGAGATTGTGGTGGTGGCGGCGGCCATGTGTTGGTTGCTGAT
GCTATGCTTGAAGACCCTTCACAGGGACTGCCCTTCTGCTGGCAGGTGTAG

Table S3. Protein sequence of BdSBP genes.

>BRADI1G02760.1 peptide: BRADI1G02760.1 pep:protein_coding
MEAAGVGSQSRRLYGGGLGEPAQDMRGKRLFGWDLNDWSWDSERFVATPAPAAEKANGLS
LNSSPSSSEEADVEVARSGNVRGDSDKRKRVVVIDDDGDDQKDEDPVDNNGRVLSLRIGG
DTTVAGGAVEGGAVNEEDRNGKKIRVQGGSSSGPACQVEGCCADLSAAKDYHRRHKVCEM
HAKANTAVVGNTVQRFCQQCSRFHLLQEFDEGKRSCRRRLAGHNRRRRKTRPEIAVGGTP
IEDKVGSYLVLSLLGICANLNSENAEHLQGQELLSNLWRNLGTVAKSLDPKELCKLLETC
QSMQNGSNTGTSEAANALVNSAAVEAAGPSNSKAPFTNGGQREQTSSAVIPLQSNATVVA
TPETPACRIRNFDLNDTCNDMEGFEDGSNCPSVQQDSTQSPPQTSGNSDSTSAQSLSSSN
GDAQCRTDKIVFKLFDKVPSDLPPILRSQILGWLSSSPTDIESYIRPGCIILTVYLRLVD
SAWRELSENMSLYLDKLLSSSTDNFWASSLVFVMVRHQIVFMHNGQVMLDRPLAPNSHHY
CKVLCVSPVAAPSSATVNFRVEGFNLVSASSRLICSFEGRCIFQEDTAIVDDAAEHEDIE
CLNICCSLPGSRGRGFIEVEDSGFSNGFFPFIVAEQDVCSEVCELESIFKSSSHEQADND
NARSQALEFLNELGWLLHRANIISKHDKVELPLAAFNLLRFRNLGIFAMEREWCAVTKVL
LDLLFDGFVDVGLQSPKEVVLSENLLHTAVRGKSVRMVRFLLRYKPSKDQKEIAESYLFR
PDARGPSTFTPLHIAAATSDAEDVLDALTSDPGLVGLNAWKNARDETGFTPEDYARQRGN
DAYMDLVQKKIDKNLGEGHVVLGVPSSMCPVLTDGAKPGDISLEICKSMPMAPQPVSRCN
ICSRQARMYPSSFANTFLYRPAMFTVMGVAVICVCVGILLHTLPKVYAAPNFRWELLERG
PM
>BRADI1G26720.1 peptide: BRADI1G26720.1 pep:protein_coding
MDRKDKSRKSSSSAASMAALAAAGGDRMAPPSSGDEDQKPNLVNVPVVATGASSSSAAAV
RRGGGGGAAGGPVAVGGAGAGGPSCQAERCPADLTEAKRYHRRHKVCEAHAKAAVVLVAG
LRQRFCQQCSRFHELLEFDDTKRSCRRRLAGHNERRRKSSADANGGDGCRHVDQDGRSNP
GNPPPLNHFQIR
>BRADI1G31390.1 peptide: BRADI1G31390.1 pep:protein_coding
MMSGRMNNSTGSDDFPFAPTPPPSYGGFEQRALYDSFDFAAAFQFQHQQEHHQMLSLPPN
ANTSNLLHHPMAPPPPPAAMSMQLPIPMPQMHGHGGDAMIYPALGMAVKREGEVAEGRNI
GLNLGRRTYFSPGDMMAVDRLLMRSRLGGVFGLGFGGPGGHGHHHQPPRCQAEGCKADLS
GAKHYHRRHKVCEYHAKASLVSAGGKHQRFCQQCSRFHVLTEFDEAKRSCRKRLAEHNRR
RRKPATTNGTATSAAKDSATPPSSKKPNNGAGGAIIGSYTVDNKSKTYTLFCCSFERRQV
VDHLLQHQRHQLPAAAAAGPKQGGGSGGAHPRRIAAGSEQRRAPARRPRPRPPSSGAALH
HLTLAQQQQQQQRQQQQHPVVLLGVLQRNAAACDSEQRRWRGLRPEQPPRQQQQQQHASV
RGN

>BRADI2G11240.1 peptide: BRADI2G11240.1 pep:protein_coding
MEAGFRREGGQTGLANTMSSELSKNKKKKGLEWDLNDWRWDGNLFLATPSSNAVAAPSGC
GSRELGRSEDGIDLSVADKRRRVSPVDNHGECSNAAIANGDHDRVVGRRGQSSREGRHAN
ATGAYSTSAPYCQVDGCHADLRDSRDYHKRHKVCEVHTKSTVVRIKSIEHRFCQQCSRFH
LLPEFDEGKKSCRSRLAKHNGRRRKAPAQAGAAGNTSSENQSLTNTLLLLLKQLSGQDPG
SSSEQSNGPNFLVNLLKNLAAIAGTQAYQDMLKNANSASISSNDANYVVNGFTNEPTRSP
IPVGTESSAEHTVKQHVQNFDLNDAYVEEDESRTDKIVFKLFGKEPKDFPVDLRAQMLNW
LSHYPSDMESHIRPGCVILTIYLRLPNWMWDKLKVNPAPWIENLISISTDGFWETGWLYT
RLQDRLALSCNGRLMLVSPWQPLIGDKHQILCVTPIATACSSTANFSVKGFNIVQPTTKL
KIMTKAAFPFPLLSLKNLYVLRFEWCSVVKKLLDTLFQGNIDVDVRSPFEFVLGEGLVFT
AVNKRAKRLVEFLLRYTTNSALVARGAVSPVRFLFTPDITGSSNITPLHIAASMSDGAGV
LDALTDDPQQLGIKAWKHARDTTGYTPEDYAQKRGHISYIQMVENKINSRLPKAHVSVSM
TISPSTTDISEKHAGRSKSTNQTTLDIEKSQRSDKRPPSCRQCVQLQHIAYHPRPNRFLS
NRPAVLSLVAIGAVCVCVGLIMQSPPTVGVGMTGPFLWSSLNYGPI

>BRADI2G25580.1 peptide: BRADI2G25580.1 pep:protein_coding
MDASDSGGASAAPDAGEPDWDWNHILEFAVRGDDSLILPWDDTLGTAEAGPAEGAFLPAP
SPALPVEAEPVAPPPPVEAGGSRSGVRKRDPRLVCPNYLAGIVPCACPELDEMAAAAEAE
EVASEMLAGPRKKSRPASRGNGVAAGGGGGGSGVAGRGGAVEMKCQVPGCEADIRELKGY
HKRHRVCLRCAHATAVMLDGVQQRYCQQCGKFHVLLDFDEDKRSCRRKLERHNKRRRRKP
DSKGAFEKEVDEQLDLSADGSGGCELREENTDGTTCEMVETVLSNKVLDRETPVGSEDVL
SAPTCTQPSLQNEQSKSVVTFAASVEGCLGTEQENANITNSSMHDTKSVYSSSCPTGRIS
FKLYDWNPAEFPRRLRNQIFEWLSSMPVELEGYIRPGCTILTVFIAMPQHMWDQLSEDAA
NLVRDLVNAPSSLLLGKGAFFVHVNNMIFQVLKDGATLMSTRLEVQAPRIHYVHPTWFEA
GKPVELLLCGSSLDHPKFRSLLSFDGEYLKHDCCRLTSHETIACVKNAAALDSQHEIFRI
NITQTKADTHGPGFVEVENMIGLSNFVPVLFGSKQLCSELERIQDALCGSNEKYKSVFGE
VPGATSDLCGRLELKQTAMSGFLIEIGWLIRKSSPDELKNLLSSANIKRWTSVLKFLIQN
DFINVLEIIVKSSDNIIGSEILSNLERGRLEHHVTTFLGYVRHARNIVEDRAKYDKQTQL
ETRWCGDSASNQPNLGTSVPFAKENTGDGSEYDLHPTNVECKEEERMLLVSPKAVSHRQC
CSPEMNARWLNPTLGAPFPGGAMRTRLVKTVVVAAVLCFAACVVVFHPDRVGVLAAPVKR
FLFSDSPSS

>BRADI2G59110.1 peptide: BRADI2G59110.1 pep:protein_coding
MDWEAKMPSWDLGTVVGPSGGGGGGLDLKLGGPTSWRPVPAATAAPVAPSSSPTPAKRAR
AGAPAPACSVEGCAADLSRCREYHRRHKVCEAHSKTPVVSVAGQQQRFCQQCSRFHLLVE
FDEVKRSCRKRLDGHNRRRRKQQPDPLGPAGLFANHHGGGTRFVSYPQIFSTTTSMAPEH
KWPGSIAVKTEADAFQVEQYYSSLQLNGAANAAASLFHGKERSKRFPFLSDHHGDTGFQQ
QPFTITPSSESSSNSSSRHSNGNKMFATNDGGPDHNCALSLLSDSPTTQAAHTMITAAAQ
HLGGARIHYNVGGDGGGGVSLTGLSYASAIMGDNKGSAQAAMASSTRHAGAVTTAPSVAA
QLQQYHGYYQHQVNGDQGNSADAGASMQALPFSSW

>BRADI3G03510.1 peptide: BRADI3G03510.1 pep:protein_coding
MGSFGMDWNQKSSVLWDWENLLPAGTNAAENPKNGMQAEQRFASVVAAMANESRHSSGSS
GTFSSNSEMGYGSSKSSLSASIDSSSKVGNSMEFRFAAAANPDSNNSKNTELGKVDDTGA
GTSPSSVIAVSSGEPVIGLKLGKRTYFEDACGAQNVKNSPLGAGAPNPSPASVKKAKVDQ
QKPHNSYCQVEGCRVDLSSAKDYHRKHRVCEVHSKTPKVVVAGLERRFCQQCSRFHALAE
FDQIKRSCRRRLNDHNHRRRKPQPEAISFSTSRLSTMFYDARQQTSLLFSEAPYVQMRNC
ASSSWDDPGPGGFKFTETKAPWLKPTTAAGVDAMHLSSQQVSNSIMPHGAHHGFDGFMSF
KGTGMKFLNQGVEASTVVSDTSGAPDLQHALSLLSNNPAGAGNLQPSPQMHSGTTADIAG
TSNPAMHVLGSSPGLWLDGPPLDNHHPRFQAFDLLGGHDSTMPHELQLPKPSYDHHAASH
FDRMH

>BRADI3G05510.1 peptide: BRADI3G05510.1 pep:protein_coding
MEWTAPKPATSPPTHLLWDWGDSAAPGSSGDAAGRRGKEKRAKGEEGGGGGGGGGAVVR
CQVEGCGVELRAAKDYHRKHRVCEAHTKCPRVVVAGQERRFCQQCSRFHALSEFDEKKRS
CRRRLSDHNARRRKQQPDAFSFAPARLPSTLIFDDRRQISFVWNKGPPSHVRPFACSPWE
SPSEFKLSQVKNTRGVSTNGQVHLDKSHLSNAVPTLSHDMDELFPVKGPDASLAASKLDG
APDLQHALSLLSASSRGLPDPAQQASCLVQFSGASQNSRGLHSSHGVSSASAPCAEGQPM
APSPHLVRFTVDGTSSGYDTTFFGLNKIN

>BRADI3G05720.1 peptide: BRADI3G05720.1 pep:protein_coding
MSSRQQLSPGTGTMPPISGDADFGSCYTSTHHPYAAFDGSPSAAVDHRPPLLHHHHQQLY
DTTGLDYAALFPFAPQQDNNPPAHLFPNQLPPFTANSTTMLLQPPMLTPLPGLPTSSPPP
APGDAYQLHHPFGGFQLKRENEGGLFPFSDAMAASGVSGVGGGSGGRIGLNLGRRTYFSP
ADVLAVDRLLMRTRGGLGGGGMGVLGLGLGGGIMQQPPRCQAEGCKADLSAAKHYHRRHK

VCEYHAKAAAVAANGKQQRFCQQCSRFHVLAEFDEAKRSCRKRLTEHNRRRRKPVGVQGK
DSPPPPPSKKLEAGITTSSYAGDHTTTNKSSSTAAAAVLSPSASAFSCLQQQEEELDDNN
EGSGGRPTTLSLAAPPQRDYGGALDTMLLMHHHHHQAAAAVVQDDDQEQDFMMTSLVQSH
HHHQQQQQQGEVSGNILSCSPTASDQRRQNHQQMQINDGGDDSGCCNNSGMQQQQHFFEV
DFM

>BRADI3G40030.1 peptide: BRADI3G40030.1 pep:protein_coding
MEIGSGGGGGSAVAGGGDGGGGGDDQLRHGLQFGKKIYFEDSSSGGSSSGGGGANAASSS
SKPAASGGGKKGKGSAAPPRCQVEGCEVDLTASKGYYCRHKVCSMHAKSPRVVVAGLEQR
FCQQCSRFHQLPEFDQGKRSCRRRLAGHNERRRRPPAGPLASRYGRLAASFEESGRFRSY
LLDFSYPRVPSSVRDAWPAVRPGYRMPSEVQWQGNLEPRAQSGAAMGYGGHAYSSHGFPS
PGLPPGGCLAGVAADSSCALSLLSTQPWDTTTHGASHDHRSAAMSAAAASFDGNPVAVAP
SVMAGNYLPPPANPWSGSRGHEGGRNVPPDPQLPHDVPLHEVHPAGSSQQGHFSGELELA
LQGNRPAAPGPRYGAGRSTFDHPGSSTNWSQ

>BRADI3G40240.1 peptide: BRADI3G40240.1 pep:protein_coding
MQREVGPQVASPLYLHHQIQPLPPHAVAAAPKKRGNPWPASAEGAAAGSAGAGNWNPAMW
DWDSRAFTARPSSDALRLGAGAQNHHHHNHHQQQQRQPAAMAAEAQQRQGPGGLSLQLAT
REEASVAMDVSPTAIMSSSPSPPAAPAHEQAARPSKKVRSESPGTGSGGGGNGGGGSSGN
GGGSYPMCQVDDCRADLTSAKDYHRRHKVCEIHSKTTKAVVGHQMQRFCQQCSRFHPLSE
FDEGKRSCRRRLAGHNRRRRKTQPTDVASQLLLPDNQENAGNRTQDIVNLITVIARLQGG
NVGKLPSIPPIPDKDNLVQIISKINSINTANALGKSPPSEVIDLNASHGQQQDAVQKATN
VIDKQAVPSTMDLLTVLSGGNGASTPETNTSQSQGSSDSSGNNKSKSHSTEPAYVVNSHE
KSIRAFPAAGVIRSNSPHDSPPEMYKQPDRDARPFLSLQLFGSTYDDIPAKMDTANKYLS
SESSNPMDERSPSSSPPVTHTFFPIRSANDGITHPRAGDYGEDAATVENSTTRAWCAPPL
ELFKDSERPTENGSPPNLTYQSCYASTSGSDHSPSTSNSDGQDRTGRIIFKLFGKEPGSI
PGNLRDEVVNWLKHSPTEMEGYIRPGCLVLSMYLSMPAIAWDELEENLLHRVNTLIQGSD
SDFWRNGRFLVRSDNQLVSYKDGTTRLSKSWRTWNTPELTLVTPIAVVGGRKSSLILKGR
NLTIPGTQIHCTTEGKYISKEVLCSAYPGTIYDDSGVETFNLPGEPNLILGRCFIEVENR
FRGNSFPVIFANSSICQELRNLEAELEDSRFPDVSSEDQVDDTRRLKPRDQVLHFLNELG
WLFQKAAACIPSTKSDVSDSELIQFSTARFRYLLLFSNERDWCSLTKTLLDILSKRSLVS
DELSQETLEMLSEIHLLNRAVKRKSRRMVHLLVQFVVICPDNSKLYPFLPNYPGPGGLTP
LHLAASIDDAEGVVDALTDDPQQIGLNCWHSVLDDDGQSPEAYAKFRNNDSYNELVAQKL
VDKKNSQVTIVLNKGEICMDQPGNGGGNNASGIQAMGIKSCSQCAILESGLLSRPMHSRG
LLARPYIHSMLAIAAVCVCVCVFMRALLRFNSGRSFKWERLDFGTS

>BRADI3G41250.1 peptide: BRADI3G41250.1 pep:protein_coding
MDWDLKMPAAGAGAAWDLAELEQGGGGGGPSADGIAAPAAAAGGGGRAECSVDLKLGGLG
ESGGGQAQAQAQDSSTRGGKAPVAASAPAPGKRPRAAAGSSSSSGGGGGGGGQGQQCPSC
AVDGCRADLSRCRDYHRRHKVCEAHSKTPVVAVAGRDMRFCQQCSRFHLLTEFDETKRSC
RKRLDGHNRRRRKPQPDPMNSASFMTSQQGTRFSPFPNPRPEQSWPGIIKTEESPYYAHQ
IPIGISNRQHFGGSTSTYAKEGRRFPFLQEGEINFATGVTLEPSVCQPHPRTAAPPDSSG
SSKMFSDGLTPVLDSDCALSLLSAPANSSGIDVGRMVQVQQNEHIPIAQPLVSSLQFSSS
SWFARSQAATGAVPPTGFSCPVVENEQLNTVLSSDNNEMNYSGIFHVGGEGSSDGAPPSL
PFTWQ

>BRADI4G33770.1 peptide: BRADI4G33770.1 pep:protein_coding
METGSSGGGGGGGGDDFHGLKFGQKIYFEQDVASGSGATTSAAGGGAGAGGEGAAAAASA
AAAAGNGAAHAQAQPPRCQVEGCGVDLSGDKTYYCRHKVCSMHSKAPLVVVAGIQQRFCQ
QCSRFHQLPEFDQGKRSCRRRLAGHNERRRKPPPGPLSSRYGRLAASFHEDPGRFRSFLL

DFSYPRVPSSVRDAWPTAHPGEHHRMPGTAAQWQGSHELHHPHRSTVAGYGVDHHAYAGQ
GSSSGGAAPMYPSFELPAAECIAGVTAADSSCALSLLSTQPWDHSAHSASHNRPPAMSTA
SAFQVSPVAPSVMASDYMAAASNSAWASTRGRNMQQHQQQQHHHHHDAVMSDVHPGSVHQ
HGGQFGELELALQQGRAGAPNTPHAEHGSSGAGAFGHHSSNAMNWSL

>BRADI4G34667.1 peptide: BRADI4G34667.1 pep:protein_coding
MAAPAAVAAPGIAAAAPRGPECSVDLKLGGLGDFGAAADAMKETSAAKAPAVPSASAAAV
VPSASPLKRPRPGGGGGGQCPSCAVDGCKADLSKCRDYHRRHKVCEAHSKTPVVVVAGRE
MRFCQQCSRFHLLAEFDEAKRSCRKRLDGHNRRRRKPQVESMSSGSFMTSQQGTRFASFP
VPRPEPSWSGMIKSEDSSPYYTHQVLNNNNRPHFAGSTSTYSKEGRRFPFLHEGDQMSFS
TGVEIPVCQPLLKSVVAPPPPESSSSNNNKMFSDGQLTHALDSDCALSLLSSPGNSSSVD
VSRMVRPTEHIPPVSQHLVPNLQFASSSWFACSQASSGGGVSAAAGFAFPSMDSTQLNTA
GLVPNSNDHEMNCHGIFHVGAEGSSDGTSPPLPFSWQQ

>BRADI5G17720.1 peptide: BRADI5G17720.1 pep:protein_coding
MASAQRQQQELTSLKLGKRPCYLPGWRDGQLAQVGAAGHVDVNGGRRAVAAPEGKRKEKA
AAATATAAVARCQVEGCHLALAGAKEYHRRHKVCEAHSKAPRVVVHGAEQRFCQQCSRAH
GVTVYMIVRSMRVAGCVCKCRFHAMSEFDDAKRSCRRRLAGHNERRRKSNASEAMARGSA
HTHGKPCR

>BRADI5G24670.1 peptide: BRADI5G24670.1 pep:protein_coding
MMNLPASASSCDDFIGVYGAPSNNPSPNNPPPQQPASSLFPLMDHQEQHRDHHHLGYNLE
PNSLALLPPSNAHHHHGATIAAHSAHDILQFYPTGATHHHYLAAAAAGNNPYSGHFSGAG
STFQSSYYGQQQQGPEYSYFPALVSSAEENMASFAATQLGLNLGYRTYFPPRGHGGYAYG
HHPPRCQAEGCKADLSGAKRYHRRHKVCEHHSKAPVVVTAGGLHQRFCQQCSRFHLLDEF
DDAKKSCRKRLADHNRRRRKSKPSDADAADKKRSTQASKTASTKGKAAGSSSKSTGTGDG
MDIQVVGVADLSKDQDETMGLGEVVKEMQVDPKGKASMQQQQGHHGLHQQQQSHHGFHFP
SSSAGSCFPHQSQAVSSSDNTSNIAQVQEPSLGFHQQHHHQQHNNILQLGQAMFDLDFDH

>BRADI4G18890.1 peptide: BRADI4G18890.1 pep:protein_coding
MDWAAAPEAASWGVAAEAVADPGPTMLSFASPSFSSSAAVAAQLQDRSRPASVTARRDRA
PGSAAGTVACSVDGCRSDLSRCREYHRRHKVCEAHSKTPVVVVAGQEKRFCQQCSRIGK

>BRADI4G18900.1 peptide: BRADI4G18900.1 pep:protein_coding
MVITSRFHMLAEFDEGKRSCRKRLDGHNRRRRKPQYDAMNPSSFFPYHQVNKFSVYPRTF
PIADPNAGASMHPLEHHRPFSISFSGTTKAPKHFPFLQDGSSIYSTASPTLPQPFSSRED
GNNTSSSTCNGLSSALDPECALSLLSSSLHLSPISIPSATVPAQFASSLARTAVDSQAAT
IAFASGGDCGGGGGHVLVADAMLEDPSQGLPFCWQV

Table S4. SBP domain sequence of BdSBP proteins.

>BRADI1G02760
ACQVEGCCADLSAAKDYHRRHKVCEMHAKANTAVVGNTVQRFCQQCSRFHLLQEFDEGKRSCRRRLAGHN
RRRRKTRPE
>BRADI1G26720
SCQAERCPADLTEAKRYHRRHKVCEAHAKAAVVLVAGLRQRFCQQCSRFHELLEFDDTKRSCRRRLAGHN
ERRRKSSAD
>BRADI1G31390
RCQAEGCKADLSGAKHYHRRHKVCEYHAKASLVSAGGKHQRFCQQCSRFHVLTEFDEAKRSCRKRLAEHN
RRRRKPATT
>BRADI2G11240
YCQVDGCHADLRDSRDYHKRHKVCEVHTKSTVVRIKSIEHRFCQQCSRFHLLPEFDEGKKSCRSRLAKHN
GRRRKAPAQ
>BRADI2G25580
KCQVPGCEADIRELKGYHKRHRVCLRCAHATAVMLDGVQQRYCQQCGKFHVLLDFDEDKRSCRRKLERHN
KRRRRKPDS
>BRADI2G59110
ACSVEGCAADLSRCREYHRRHKVCEAHSKTPVVSVAGQQQRFCQQCSRFHLLVEFDEVKRSCRKRLDGHN
RRRRKQQPD
>BRADI3G03510
YCQVEGCRVDLSSAKDYHRKHRVCEVHSKTPKVVVAGLERRFCQQCSRFHALAEFDQIKRSCRRRLNDHN
HRRRKPQPE
>BRADI3G05510
RCQVEGCGVELRAAKDYHRKHRVCEAHTKCPRVVVAGQERRFCQQCSRFHALSEFDEKKRSCRRRLSDHN
ARRRKQQPD
>BRADI3G05720
RCQAEGCKADLSAAKHYHRRHKVCEYHAKAAAVAANGKQQRFCQQCSRFHVLAEFDEAKRSCRKRLTEHN
RRRRKPVGV
>BRADI3G40030
RCQVEGCEVDLTASKGYYCRHKVCSMHAKSPRVVVAGLEQRFCQQCSRFHQLPEFDQGKRSCRRRLAGHN
ERRRRPPAG
>BRADI3G40240
MCQVDDCRADLTSAKDYHRRHKVCEIHSKTTKAVVGHQMQRFCQQCSRFHPLSEFDEGKRSCRRRLAGHN
RRRRKTQPT
>BRADI3G41250
SCAVDGCRADLSRCRDYHRRHKVCEAHSKTPVVAVAGRDMRFCQQCSRFHLLTEFDETKRSCRKRLDGHN
RRRRKPQPD
>BRADI4G33770
RCQVEGCGVDLSGDKTYYCRHKVCSMHSKAPLVVVAGIQQRFCQQCSRFHQLPEFDQGKRSCRRRLAGHN
ERRRKPPPG
>BRADI4G34667
SCAVDGCKADLSKCRDYHRRHKVCEAHSKTPVVVVAGREMRFCQQCSRFHLLAEFDEAKRSCRKRLDGHN
RRRRKPQVE
>BRADI5G17720
RCQVEGCHLALAGAKEYHRRHKVCEAHSKAPRVVVHGAEQRFCQQCSRAHGVTVYMIVRSMRTVYMIVRS
MRVAGCVCKCRFHAMSEFDDAKRSCRRRLAGHNERRRKSNAS
>BRADI5G24670
RCQAEGCKADLSGAKRYHRRHKVCEHHSKAPVVVTAGGLHQRFCQQCSRFHLLDEFDDAKKSCRKRLADH
NRRRRKSKPS

>BRADI4G18890
CSVDGCRSDLSRCREYHRRHKVCEAHSKTPVVVVAGQEKRFCQQCSRIGKXXXXXXXXXXXXXXXXXXXX
XXXXXXXXX
>BRADI4G18900
XXXXXXXXXXXXXXXXXXXXXXXXXXXXXXXXXXXXXXXXXXXXXXXXTSRFHMLAEFDEGKRSCRKRLD
GHNRRRRKP
>HvSPL18
SCAVDGCRADLSRCRDYHRRHKVCEAHSKTPVVTVAGREMRFCQQCSRFHLLTEFDEAKRSCRKRLDGHN
RRRRKPQPD
>HvSPL15
MCQVDDCRADLTSAKDYHRRHKVCEIHSKTTKAVVANQMQRFCQQCSRFHPLSEFDEGKRSCRRRLAGHN
RRRRKTQPT
>HvSPL16
SCAVDGCKADLSKCRDYHRRHKVCEAHSKTPLVVVAGREMRFCQQCSRFHMLAEFDEAKRSCRKRLDGHN
RRRRKPQVD
>HvSPL11
RCQVEGCGTELAAAKEYHRKHRVCEAHTKSPRVVVAGQERRFCQQCSRFHGLSEFDQKKRSCRRRLSDHN
ARRRKPQPD
>HvSPL1
SCLVDGCHADLRDGRDYHRRHKVCEVHTKSTLVRIKNIEHRFCQQCSRFHLVQEFDEGKKSCRSRLEKHN
GRRRKAQAQ
>HvSPL17
RCQVEGCGVDLSGGKTYYCRHKVCSMHSKAPLVVVAGIEQRFCQQCSRFHQLPEFDQGKRSCRRRLAGHN
ERRRKPPPG
>HvSPL7A
RCQVEGCHMVLAGAKEYHRRHKVCEAHSKAPRVIVHGAEQRFCQQCSRFHAMAEFDDAKRSCRRRLAGHN
ERRRKSNAN
>HvSPL7
RCQVEGCHMVLAGAKEYHRRHKVCEAHSKAPRVIVHGAEQRFCQQCSRFHAMAEFDDAKRSCRRRLAGHN
ERRRKSNAN
>HvSPL21
RCQAEGCKTDLSAAKHYHRRHKVCEYHAKAATVAASGKQQRFCQQCSRYVVLAEFDEAKRSCRKRLTEHN
RRRRKPAGA
>HvSPL3
YCQVEGCKVDLSSVKEYHRKHRVCELHSKAPKVVVAGLERRFCQQCSRFHALSEFDQKKRSCRRRLNDHN
SRRRKPQPE
>HvSPL13
XXXXERCGADLTEAKRYHRRHKVCEAHSKAAVVVVAGLRQRFCQQCSRFHELLEFDDQKRSCRRRLAGHN
ERRRKSSAE
>HvSPL23
ACSVEGCTADLSRCREYHRRHKVCEAHSKTPVVAVAGQQQRFCQQCSRFHLLGEFDEVKRSCRKRLDGHN
RRRRKPQPD
>HvSPL20
RCQVEDCKADLSGAKHYHRRHKVCEYHAKAALVSTAGKQQRFCQQCSRSHVLMEFDEAKRSCRRRLAEHN
RRRRKPAAG
>HvSPL6
ACQVEGCCADLSAAKDYHRRHKVCEMHAKANTAVVGNTVQRFCQQCSRFHLLQEFDEGKRSCRRRLAGHN
KRRRKTRPE
>HvSPL9
RCQVPGCEADIRELKGYHKRHRVCLRCAHASAVMLDGVQKRYCQQCGKXXXXXXXXXXXXXXXXXXXXXX

```
XXXXXXXXX
>HvSPL8
RCQAEGCKADLSGAKRYHRRHKVCEHHSKAPVVVTAGGLHQRFCQQCSRFHLLDEFDDAKKSCRKRLADH
NRRRRKSKP
>HvSPL22
RCQAEGCKADLSGAKHYHRRHKVCEYHAKASLVAANGKQQRFCQQCSRSEPxxxxxxxxxxxxxxxxxx
xxxxxxxxx
>AtSPL1
VCQVENCEADLSKVKDYHRRHKVCEMHSKATSATVGGILQRFCQQCSRFHLLQEFDEGKRSCRRRLAGHN
KRRRKTNPE
>AtSPL2
HCQVEGCNLDLSSAKDYHRKHRICENHSKFPKVVVSGVERRFCQQCSRFHCLSEFDEKKRSCRRRLSDHN
ARRRKPNPG
>AtSPL3
VCQVESCTADMSKAKQYHKRHKVCQFHAKAPHVRISGLHQRFCQQCSRFHALSEFDEAKRSCRRRLAGHN
ERRRKSTTD
>AtSPL4
LCQVDRCTADMKEAKLYHRRHKVCEVHAKASSVFLSGLNQRFCQQCSRFHDLQEFDEAKRSCRRRLAGHN
ERRRKSSGE
>AtSPL5
LCQVDRCTVNLTEAKQYYRRHRVCEVHAKASAATVAGVRQRFCQQCSRFHELPEFDEAKRSCRRRLAGHN
ERRRKISGD
>AtSPL6
LCQVYGCSKDLSSSKDYHKRHRVCEAHSKTSVVIVNGLEQRFCQQCSRFHFLSEFDDGKRSCRRRLAGHN
ERRRKPAFY
>AtSPL7
RCQVPDCEADISELKGYHKRHRVCLRCATASFVVLDGENKRYCQQCGKFHLLPDFDEGKRSCRRKLERHN
NRRKRKPVD
>AtSPL8
RCQAEGCNADLSHAKHYHRRHKVCEFHSKASTVVAAGLSQRFCQQCSRFHLLSEFDNGKRSCRKRLADHN
RRRRKCHQS
>AtSPL9
RCQVEGCGMDLTNAKGYYSRHRVCGVHSKTPKVTVAGIEQRFCQQCSRFHQLPEFDLEKRSCRRRLAGHN
ERRRKPQPA
>AtSPL10
RCQIDGCELDLSSSKDYHRKHRVCETHSKCPKVVVSGLERRFCQQCSRFHAVSEFDEKKRSCRKRLSHHN
ARRRKPQGV
>AtSPL11
RCQIDGCELDLSSAKGYHRKHKVCEKHSKCPKVSVSGLERRFCQQCSRFHAVSEFDEKKRSCRKRLSHHN
ARRRKPQGV
>AtSPL12
CCQVDNCGADLSKVKDYHRRHKVCEIHSKATTALVGGIMQRFCQQCSRFHVLEEFDEGKRSCRRRLAGHN
KRRRKANPD
>AtSPL13A
ICLVDGCDSDFSNCREYHKRHKVCDVHSKTPVVTINGHKQRFCQQCSRFHALEEFDEGKRSCRKRLDGHN
RRRRKPQPE
>AtSPL14
MCQVDNCTEDLSHAKDYHRRHKVCEVHSKATKALVGKQMQRFCQQCSRFHLLSEFDEGKRSCRRRLAGHN
RRRRKTTQP
>AtSPL15
```

RCQVEGCRMDLSNVKAYYSRHKVCCIHSKSSKVIVSGLHQRFCQQCSRFHQLSEFDLEKRSCRRRLACHN
ERRRKPQPT
>AtSPL16
KCQVDNCKEDLSIAKDYHRRHKVCEVHSKATKALVGKQMQRFCQQCSRFHLLSEFDEGKRSCRRRLDGHN
RRRRKTQPD
>OsSPL1
CCQVDGCTVNLSSARDYNKRHKVCEVHTKSGVVRIKNVEHRFCQQCSRFHFLQEFDEGKKSCRSRLAQHN
RRRRKVQVQ
>OsSPL2
ACSVEGCAADLSKCVRDYHRRHKVCEAHSKTAVVTVAGQQQRFCQQCSRFHLLGEFDEEKRSCRKRLDGH
NKRRRKPQPD
>OsSPL3
HCQVEGCNVDLSSAKPYHRKHRVCEPHSKTLKVIVAGLERRFCQQCSRFHGLAEFDQKKRSCRRRLHDHN
ARRRKPQPE
>OsSPL4
RCQVEGCGVELVGVKDYHRKHRVCEAHSKFPRVVVAGQERRFCQQCSRFHALSEFDQKKRSCRRRLYDHN
ARRRKPQTD
>OsSPL5
RCQAEGCKADLSAAKHYHRRHKVCDFHAKAAAVLAAGKQQRFCQQCSRFHVLAEFDEAKRSCRKRLTEHN
RRRRKPTAG
>OsSPL6
ACQVEGCTADLTGVRDYHRRHKVCEMHAKATTAVVGNTVQRFCQQCSRFHPLQEFDEGKRSCRRRLAGHN
RRRRKTRPE
>OsSPL7
RCQVEGCDITLQGVKEYHRRHKVCEVHAKAPRVVVHGTEQRFCQQCSRFHVLAEFDDAKKSCRRRLAGHN
ERRRRSNAS
>OsSPL8
RCQAEGCKADLSSAKRYHRRHKVCEHHSKAPVVVTAGGLHQRFCQQCSRFHLLDEFDDAKKSCRKRLADH
NRRRRKSKPS
>OsSPL9
KCQVPGCEADIRELKGYHRRHRVCLRCAHAAAVMLDGVQKRYCQQCGKFHILLDFDEDKRSCRRKLERHN
KRRRRKPDS
>OsSPL10
RCQAEGCKADLSGAKHYHRRHKVCEYHAKASVVAASGKQQRFCQQCSRFHVLTEFDEAKRSCRKRLAEHN
RRRRKPAAA
>OsSPL11
RCQVEGCGLELGGYKEYYRKHRVCEPHTKCLRVVVAGQDRRFCQQCSRFHAPSEFDQEKRSCRRRLSDHN
ARRRKPQTD
>OsSPL12
YCQVEGCKVDLSSAREYHRKHKVCEAHSKAPKVIVSGLERRFCQQCSRFHGLAEFDQKKKSCRRRLSDHN
ARRRKPQQE
>OsSPL13
RCQVERCGVDLSEAGRYNRRHKVCQTHSKEPVVLVAGLRQRFCQQCSRFHELTEFDDAKRSCRRRLAGHN
ERRRKSAAD
>OsSPL14
RCQVEGCGADLSGIKNYYCRHKVCFMHSKAPRVVVAGLEQRFCQQCSRFHLLPEFDQGKRSCRRRLAGHN
ERRRRPQTP
>OsSPL15
MCQVDDCRADLTNAKDYHRRHKVCEIHGKTTKALVGNQMQRFCQQCSRFHPLSEFDEGKRSCRRRLAGHN
RRRRKTQPT

>OsSPL16
SCAVDGCKEDLSKCRDYHRRHKVCEAHSKTPLVVVSGREMRFCQQCSRFHLLQEFDEAKRSCRKRLDGHN
RRRRKPQPD
>OsSPL17
RCQVEGCGVDLSGVKPYYCRHKVCYMHAKEPIVVVAGLEQRFCQQCSRCSVHMVRFHQLPEFDQEKKSCR
RRLAGHNERRRKPTPG
>OsSPL18
SCAVDGCKADLSKHRDYHRRHKVCEPHSKTPVVVVSGREMRFCQQCSRFHLLGEFDEAKRSCRKRLDGHN
RRRRKPQAD
>TaSPL1
SCQVDGCHADLSDDRDYHRRHKVCEPHTKSTLVRIKNIEHRFCQQCSRFHLVQEFDEGKKSCRSRLATHN
RRRRKAPAE
>TaSPL3
YCQVEGCKVDLSSVKDYHRKHRVCELHSKAPKVVVAGLERRFCQQCSRFHALAEFDQKKRSCRRRLNDHN
SRRRKPQPE
>TaSPL6
ACQVEGCCADLSAAKDYHRRHKVCEMHAKANTAVVGNTVQRFCQQCSRFHLLQEFDEGKRSCRRRLAGHN
KRRRKTRPE
>TaSPL8
RCQAEGCKADLSGAKRYHRRHKVCEHHSKAPVVVTAGGLHQRFCQQCSRFHLLDEFDDAKKSCRKRLADH
NRRRRKSKPS
>TaSPL15
MCQVDDCRADLTSAKDYHRRHKVCEIHSKTTKAVVGNQMQRFCQQCSRFHPLSEFDEGKRSCRRRLAGHN
RRRRKTQPT
>TaSPL17
RCQVEGCGVDLSGAKQYHSRHKVCSMHTKEPRVVVAGLEQRFCQQCSRFHQLPEFDQGKRSCRRRLAGHN
ERRRKAPPG
>TaSPL20
RCQVXDCXADLSGAKHYHRRHKVCEYHAKAXLVSAAGKQQRFCQQCSRFHVLTEFDEAKRSCRRRLAEHN
RRRRKPAAG
>TaSPL21
RCQAEGCKADLSAAKHYHRRHKVCEYHAKAATVAASGKQQRFCQQCSRFHVLAEFDEAKRSCRKRLTEHN
RRRRKPAGA
>TaSPL22
RCQAEGCKADLSGAKHYHRRHKVCEYHAKASLVAAAGKQQRFCQQCSRFHVLTEFDEAKRSCRKRLAEHN
RRRRKPASS
>TaSPL23
PCSVEGCTADLSRCREYHRRHKVCEAHSKTPVVAVAGQQQRFCQQCSRFHLLGEFDEVKRSCRKRLDGHN
RRRRKPQPD

Table S5: List of *BdSBPs* co-expressed genes.

GeneID	Description
**BRADI3G05720 co-expressed genes**	
bradi3g05720	OsSPL10 - SBP-box gene family member, expressed
bradi2g48700	meiosis 5, putative, expressed
bradi4g34780	nodulin MtN3 family protein, putative, expressed
bradi1g73877	male sterility protein, putative, expressed
**BRADI3G41250 co-expressed genes**	
bradi1g22310	igA FC receptor precursor, putative, expressed
bradi5g06620	BEE 3, putative, expressed
bradi4g05410	stress responsive protein, putative, expressed
bradi4g42770	expressed protein
bradi5g12720	GASR4 - Gibberellin-regulated GASA/GAST/Snakin family protein precursor, expressed
bradi3g18440	Broad Complex BTB domain with Meprin and TRAF Homology MATH domain
bradi3g41250	OsSPL16 - SBP-box gene family member, expressed
**BRADI5G24670 co-expressed genes**	
bradi3g21030	POEI3 - Pollen Ole e I allergen and extensin family protein precursor, expressed
bradi4g06485	haemolysin-III, putative, expressed
bradi3g20980	POEI11 - Pollen Ole e I allergen and extensin family protein precursor, expressed
bradi1g25157	CSLF3 - cellulose synthase-like family F; beta1,3;1,4 glucan synthase, expressed
bradi2g40620	MYB family transcription factor, putative, expressed
bradi2g49340	profilin domain containing protein, expressed
bradi2g60870	expressed protein
bradi3g48800	uncharacterized protein At4g06744 precursor, putative, expressed
bradi1g31390	OsSPL10 - SBP-box gene family member, expressed
bradi2g03807	GDSL-like lipase/acylhydrolase, putative, expressed
bradi1g70420	expressed protein
bradi3g37680	Cupin domain containing protein, expressed
bradi1g63370	POEI7 - Pollen Ole e I allergen and extensin family protein precursor, putative, expressed
bradi1g72180	uroporphyrinogen III synthase, putative, expressed
bradi3g55270	GDSL-like lipase/acylhydrolase, putative, expressed
bradi1g50740	expressed protein
bradi3g21000	POEI11 - Pollen Ole e I allergen and extensin family protein precursor, expressed
bradi3g50200	aldehyde dehydrogenase, putative, expressed
bradi5g17420	fibroin heavy chain precursor, putative, expressed
bradi1g69900	YABBY domain containing protein, putative, expressed
bradi1g68400	POEI16 - Pollen Ole e I allergen and extensin family protein precursor, expressed
bradi3g20970	POEI14 - Pollen Ole e I allergen and extensin family protein precursor, expressed
bradi3g46720	phosphopantothenate--cysteine ligase, putative, expressed
bradi5g24670	OsSPL8 - SBP-box gene family member, expressed
bradi3g42240	OsSAUR36 - Auxin-responsive SAUR gene family member, expressed
bradi3g37670	Cupin domain containing protein, expressed
bradi2g48760	GDSL-like lipase/acylhydrolase, putative, expressed
bradi4g19480	RALFL14 - Rapid ALkalinization Factor RALF family protein precursor, expressed
bradi3g07010	lipoxygenase, putative, expressed
bradi5g15160	GDSL-like lipase/acylhydrolase, putative, expressed
bradi2g03320	expressed protein

bradi3g31527	CCB2, putative, expressed
bradi5g23770	LTPL128 - Protease inhibitor/seed storage/LTP family protein precursor, expressed
bradi3g36510	formin, putative, expressed
bradi3g48770	uncharacterized protein At4g06744 precursor, putative, expressed
bradi3g49590	OsSCP8 - Putative Serine Carboxypeptidase homologue, expressed
bradi1g44010	3-ketoacyl-CoA synthase, putative, expressed
bradi2g60270	classical arabinogalactan protein 26 precursor, putative, expressed
bradi2g12057	pectinacetylesterase domain containing protein, expressed
bradi1g68390	POEI1 - Pollen Ole e I allergen and extensin family protein precursor, expressed
bradi1g04630	polygalacturonase, putative, expressed
bradi3g09590	polygalacturonase, putative, expressed

BRADI3G03510 co-expressed genes

bradi1g77880	expressed protein
bradi2g59047	exostosin family domain containing protein, expressed
bradi5g02330	OsRhmbd10 - Putative Rhomboid homologue, expressed
bradi4g41990	harpin-induced protein 1 domain containing protein, expressed
bradi2g34030	OsIAA15 - Auxin-responsive Aux/IAA gene family member, expressed
bradi4g36730	expressed protein
bradi1g36660	glycosyltransferase family protein, putative, expressed
bradi1g37250	RMD5 homolog A, putative, expressed
bradi1g51250	serine threonine kinase, putative, expressed
bradi4g40182	glutathione S-transferase, putative, expressed
bradi1g01840	protein kinase domain containing protein, expressed
bradi1g50280	expressed protein
bradi4g06680	expressed protein
bradi2g43930	expressed protein
bradi3g33520	WD repeat-containing protein 8, putative, expressed
bradi4g13670	patatin, putative, expressed
bradi3g05010	tubulin/FtsZ domain containing protein, putative, expressed
bradi1g44730	CREG1 precursor, putative, expressed
bradi4g03680	heparanase-like protein precursor, putative, expressed
bradi1g27710	RIN3, putative, expressed
bradi5g17370	GATA zinc finger domain containing protein, expressed
bradi1g20080	sec20 domain containing protein, expressed
bradi1g30510	haemolysin-III, putative, expressed
bradi3g34077	mitochondrial carrier protein, putative, expressed
bradi4g30320	vesicle transport v-SNARE protein, putative, expressed
bradi2g23690	transporter, major facilitator family, putative, expressed
bradi2g13670	natural resistance-associated macrophage protein, putative, expressed
bradi2g52660	domain of unknown function, DUF250 domain containing protein, expressed
bradi1g22050	6-phosphogluconolactonase, putative, expressed
bradi4g44250	harpin-induced protein 1 domain containing protein, expressed
bradi3g60400	para-hydroxybenzoate--polyprenyltransferase, mitochondrial precursor, putative,
bradi3g57320	nuclear transcription factor Y subunit, putative, expressed
bradi3g03510	OsSPL3 - SBP-box gene family member, expressed
bradi1g57137	OsFBLD3 - F-box, LRR and FBD domain containing protein, expressed
bradi5g08630	expressed protein

Bradi4g33770 co-expressed genes

bradi2g61130	eukaryotic aspartyl protease domain containing protein, expressed
bradi1g49400	expressed protein
bradi5g19080	DUF623 domain containing protein, expressed
bradi5g07230	glycosyl hydrolase, putative, expressed
bradi5g16230	OsSCP25 - Putative Serine Carboxypeptidase homologue, expressed
bradi4g35510	3-ketoacyl-CoA synthase precursor, putative, expressed
bradi5g00550	thaumatin, putative, expressed
bradi5g00750	transferase family protein, putative, expressed
bradi4g33237	OsSub58 - Putative Subtilisin homologue, expressed
bradi4g15460	glucan endo-1,3-beta-glucosidase precursor, putative, expressed
bradi1g57890	expressed protein
bradi3g56480	transporter-related, putative, expressed
bradi2g53610	DUF623 domain containing protein, expressed
bradi4g33770	OsSPL17 - SBP-box gene family member, expressed
bradi3g57610	glucan endo-1,3-beta-glucosidase precursor, putative, expressed
bradi1g68607	F-box protein, putative, expressed
bradi1g11790	possible lysine decarboxylase domain containing protein, expressed
bradi1g06460	TCP family transcription factor, putative, expressed
bradi3g31907	hydrolase, alpha/beta fold family domain containing protein, expressed
bradi2g33330	expressed protein
bradi5g16497	expansin precursor, putative, expressed
bradi1g23040	expressed protein
bradi1g53730	GIY-YIG catalytic domain containing protein, putative, expressed
bradi3g38740	expressed protein
bradi3g28657	alcohol oxidase, putative, expressed
bradi3g06550	GDSL-like lipase/acylhydrolase, putative, expressed
bradi1g30460	OsFBX248 - F-box domain containing protein, expressed
bradi2g26063	serine/threonine-protein kinase, putative, expressed
bradi1g71465	membrane associated DUF588 domain containing protein, putative, expressed
bradi3g51270	expressed protein
bradi3g39890	polygalacturonase inhibitor 2 precursor, putative, expressed
bradi4g31080	OsSAUR37 - Auxin-responsive SAUR gene family member, expressed
bradi1g72566	acyl-desaturase, chloroplast precursor, putative, expressed
bradi2g49500	pectinesterase, putative, expressed
bradi1g69690	SEC14 cytosolic factor family protein, putative, expressed
bradi2g54980	ATROPGEF7/ROPGEF7, putative, expressed
bradi2g16800	ATROPGEF7/ROPGEF7, putative, expressed
bradi1g31390	OsSPL10 - SBP-box gene family member, expressed
bradi5g24990	OsSAUR22 - Auxin-responsive SAUR gene family member, expressed
bradi4g12140	plant protein of unknown function domain containing protein, expressed
bradi2g24040	dof zinc finger domain containing protein, putative, expressed

BRADI1G02760 co-expressed genes

bradi4g26810	serine/threonine-protein kinase, putative, expressed
bradi3g06170	homeobox protein knotted-1, putative, expressed
bradi1g62320	expressed protein
bradi1g07540	expressed protein
bradi3g57300	MYB family transcription factor, putative, expressed
bradi3g00530	G-protein alpha subunit, putative, expressed
bradi3g07090	outer membrane protein, OMP85 family protein, expressed

bradi3g48042	vesicle transport protein GOT1B, putative, expressed
bradi1g29020	glutamine-dependent NAD, putative, expressed
bradi3g22907	caffeine-induced death protein 1, putative, expressed
bradi5g17160	diacylglycerol kinase, putative, expressed
bradi1g74210	pattern formation protein EMB30, putative, expressed
bradi2g48657	suppressor of phythchrome A, putative, expressed
bradi4g08870	hhH-GPD superfamily base excision DNA repair protein, expressed
bradi3g11120	conserved oligomeric Golgi complex component 4, related, putative, expressed
bradi1g77087	DCL2, putative, expressed
bradi2g50590	AP003256-AK101847 - NBS/LRR genes that are S-rich,divergent TIR, divergent NBS,
bradi4g25497	expressed protein
bradi3g53020	calpain, putative, expressed
bradi3g22580	FAD binding domain of DNA photolyase domain containing protein, expressed
bradi5g13810	serine/threonine protein phosphatase 2A 59 kDa regulatory subunit Bgamma isoform,
bradi2g46230	DDT domain containing protein, putative, expressed
bradi3g22650	exonuclease, putative, expressed
bradi2g14200	pleckstrin homology domain-containing protein-related taxo, putative, expressed
bradi5g05190	cation efflux family protein, putative, expressed
bradi4g36200	pantothenate kinase, putative, expressed
bradi3g18470	expressed protein
bradi4g16790	lung seven transmembrane domain containing protein, putative, expressed
bradi4g33460	expressed protein
bradi4g35720	UBX domain-containing protein, putative, expressed
bradi1g78090	expressed protein
bradi2g12320	galactosyltransferase family protein, putative, expressed
bradi3g40640	calcium-transporting ATPase, plasma membrane-type, putative, expressed
bradi1g62950	tetratricopeptide repeat domain containing protein, expressed
bradi2g16820	tRNA-binding arm, putative, expressed
bradi2g54850	regulator of chromosome condensation, putative, expressed
bradi1g70460	zinc finger family protein, putative, expressed
bradi3g60200	WD40-like, putative, expressed
bradi4g02900	protein kinase domain containing protein, expressed
bradi2g39207	MLA6 protein, putative, expressed
bradi3g54120	two-component response regulator, putative, expressed
bradi2g14960	dnaJ domain containing protein, expressed
bradi1g11500	dynamin family protein, putative, expressed
bradi3g16120	FAR1 family protein, expressed
bradi3g30560	src homology-3 domain protein 3, putative, expressed
bradi2g26890	TKL_IRAK_CR4L.5 - The CR4L subfamily has homology with Crinkly4, expressed
bradi2g04900	serine/threonine protein phosphatase 2A 59 kDa regulatory subunit Bgamma isoform,
bradi1g32610	glycosyl transferase, group 1 domain containing protein, expressed
bradi2g16330	expressed protein
bradi4g22690	phosphatidylinositol kinase, putative, expressed
bradi2g04537	protein of unknown function domain containing protein, expressed
bradi3g59800	dynamin, putative, expressed
bradi5g14830	expressed protein
bradi1g10340	adenylyl cyclase-associated protein, putative, expressed
bradi1g02000	ion channel DMI1-like, chloroplast precursor, putative, expressed
bradi1g16820	PHD-finger family protein, expressed
bradi1g06250	DUF593 domain containing protein, expressed
bradi1g10530	expressed protein

bradi3g58580	LMBR1 integral membrane protein, putative, expressed
bradi2g23390	DHHC zinc finger domain containing protein, expressed
bradi1g33320	glycosyltransferase family 43 protein, putative, expressed
bradi4g28270	pleiotropic drug resistance protein, putative, expressed
bradi2g38841	disease resistance protein, putative, expressed
bradi3g40350	zinc finger family protein, putative, expressed
bradi4g05580	kelch repeat protein, putative, expressed
bradi2g10910	coatomer subunit beta-1, putative, expressed
bradi3g19470	expressed protein
bradi1g53420	WD domain, G-beta repeat domain containing protein, expressed
bradi2g16840	glycosyltransferase family 43 protein, putative, expressed
bradi2g24290	coiled-coil domain-containing protein 47 precursor, putative, expressed
bradi2g23180	No annotation
bradi3g34800	intracellular protein transport protein USO1-related, putative, expressed
bradi3g60802	cation efflux family protein, putative, expressed
bradi2g10950	coatomer subunit beta-1, putative, expressed
bradi1g51507	kinesin motor domain containing protein, expressed
bradi2g54190	vacuolar ATP synthase 98 kDa subunit, putative, expressed
bradi3g18540	expressed protein
bradi2g62680	PWWP domain containing protein, expressed
bradi1g37770	pollen-specific protein SF21, putative, expressed
bradi1g11300	CASP, putative, expressed
bradi3g56010	protein binding protein, putative, expressed
bradi2g39840	expressed protein
bradi2g16490	zinc finger, C3HC4 type domain containing protein, expressed
bradi4g41777	WD domain, G-beta repeat domain containing protein, expressed
bradi4g31016	AT-rich interaction region, putative, expressed
bradi2g46340	protein kinase domain containing protein, expressed
bradi3g08960	FYVE zinc finger domain containing protein, expressed
bradi4g04390	ATP-grasp domain containing protein, expressed
bradi4g32280	protein transport protein, putative, expressed
bradi3g47860	expressed protein
bradi1g46330	inorganic H+ pyrophosphatase, putative, expressed
bradi1g62640	jmjC domain containing protein, expressed
bradi4g28170	OBP32pep, putative, expressed
bradi5g08510	CBS domain containing membrane protein, putative, expressed
bradi2g50990	expressed protein
bradi2g34040	serine/threonine protein phosphatase 2A 59 kDa regulatory subunit Bgamma isoform,
bradi3g59230	SEC6, putative, expressed
bradi3g27007	KIP1, putative, expressed
bradi2g51630	eukaryotic aspartyl protease domain containing protein, expressed
bradi2g17970	chaperone protein dnaJ 10, putative, expressed
bradi1g02760	OsSPL6 - SBP-box gene family member, expressed
bradi1g26940	expressed protein
bradi3g30070	expressed protein
bradi2g58397	expressed protein
bradi2g38800	NB-ARC domain containing protein, expressed
bradi3g15410	SET domain containing protein, expressed
bradi1g45150	galactosyltransferase family protein, putative, expressed
bradi2g35707	No annotation
bradi4g31640	Spc97 / Spc98 family protein, putative, expressed

bradi1g61590	peptidyl-prolyl isomerase PASTICCINO1, putative, expressed
bradi1g29690	EF hand family protein, putative, expressed
bradi1g09847	BSD domain containing protein, expressed
bradi3g04860	galactosyltransferase, putative, expressed
bradi2g50800	chorismate mutase, chloroplast precursor, putative, expressed
bradi4g16032	No annotation
bradi3g33350	autophagy-related protein 3, putative, expressed
bradi3g45410	expressed protein
bradi1g23980	GDP-fucose protein-O-fucosyltransferase 2, putative, expressed

BRADI3G40240 co-expressed genes

bradi1g55650	transmembrane BAX inhibitor motif-containing protein, putative, expressed
bradi2g04297	proline-rich family protein, putative, expressed
bradi3g00550	MYB family transcription factor, putative, expressed
bradi3g00867	Leucine Rich Repeat family protein, expressed
bradi1g58390	zinc ion binding protein, putative, expressed
bradi1g70000	expressed protein
bradi1g05430	BHLH transcription factor, putative, expressed
bradi4g27747	PFT1, putative, expressed
bradi2g21477	expressed protein
bradi2g58040	zinc finger, RING-type, putative, expressed
bradi1g51330	HAD superfamily phosphatase, putative, expressed
bradi3g39850	AN1-like zinc finger domain containing protein, expressed
bradi3g16670	peptide transporter PTR2, putative, expressed
bradi5g20030	SNARE associated Golgi protein, putative, expressed
bradi2g25677	expressed protein
bradi1g76990	WD repeat-containing protein, putative, expressed
bradi3g29880	vacuolar protein-sorting protein bro1, putative, expressed
bradi4g32460	heat shock protein DnaJ, putative, expressed
bradi2g07560	protein kinase domain containing protein, expressed
bradi1g58730	expressed protein
bradi1g75220	Citrate transporter protein, putative, expressed
bradi4g36460	MYB family transcription factor, putative, expressed
bradi2g56397	proton-dependent oligopeptide transport, putative, expressed
bradi4g42290	expressed protein
bradi3g40240	OsSPL15 - SBP-box gene family member, expressed
bradi2g37950	tetraspanin family protein, putative, expressed
bradi1g52110	expressed protein
bradi1g29240	expressed protein
bradi2g08937	NAK-like ser/thr protein kinase, putative, expressed
bradi1g03050	lipase class 3 family protein, putative, expressed
bradi4g07880	chloride transporter, chloride channel family, putative, expressed
bradi1g11020	protein kinase family protein, putative, expressed
bradi4g29630	malonyl-CoA decarboxylase, mitochondrial precursor, putative, expressed
bradi2g57800	helix-loop-helix DNA-binding domain containing protein, expressed

BRADI2G11240 co-expressed genes

bradi1g43580	protein kinase, putative, expressed
bradi1g34530	histone acetyltransferase HAC1, putative, expressed
bradi1g25600	TKL_IRAK_DUF26-lc.10 - DUF26 kinases have homology to DUF26 containing loci,
bradi1g76280	AMP-binding domain containing protein, expressed

bradi3g01170	OsWAK11 - OsWAK receptor-like protein kinase, expressed
bradi1g72490	SAC domain containing protein, putative, expressed
bradi1g77080	CAMK includes calcium/calmodulin depedent protein kinases, expressed
bradi4g30200	expressed protein
bradi3g51970	CAMK_CAMK_like.15 - CAMK includes calcium/calmodulin depedent protein kinases,
bradi1g49070	IQ calmodulin-binding motif domain containing protein, expressed
bradi5g02780	UDP-glucoronosyl/UDP-glucosyl transferase, putative, expressed
bradi3g37010	DUF1336 domain containing protein, expressed
bradi3g17246	OsFBL41 - F-box domain and LRR containing protein, expressed
bradi2g51727	expressed protein
bradi1g74480	protein kinase domain containing protein, expressed
bradi1g30910	DOMON domain containing protein, expressed
bradi5g16480	transporter family protein, putative, expressed
bradi2g47060	Protein kinase domain containing protein, expressed
bradi3g34270	VHS and GAT domain containing protein, expressed
bradi1g63080	CAMK_KIN1/SNF1/Nim1_like.16 - calcium/calmodulin depedent protein kinases,
bradi4g29600	lipase, putative, expressed
bradi4g20710	expressed protein
bradi1g75390	DENN domain containing protein, expressed
bradi3g13440	synaptobrevin-related family protein, putative, expressed
bradi2g51030	BTBA2 - Bric-a-Brac,Tramtrack, Broad Complex BTB domain with Ankyrin repeat
bradi3g16480	no apical meristem protein, putative, expressed
bradi4g41377	growth regulator related protein, putative, expressed
bradi3g53780	CYPRO4, putative, expressed
bradi2g47410	MDR-like ABC transporter, putative, expressed
bradi1g75140	tyrosine protein kinase domain containing protein, putative, expressed
bradi1g58720	expressed protein
bradi1g63270	expressed protein
bradi4g16000	zinc finger, C3HC4 type domain containing protein, expressed
bradi2g57710	methyltransferase, putative, expressed
bradi2g15520	CAMK_CAMK_like.5 - CAMK includes calcium/calmodulin depedent protein kinases,
bradi1g76227	protein kinase family protein, putative, expressed
bradi4g35460	ankyrin repeat-containing protein, putative, expressed
bradi5g17070	src homology-3 domain protein 3, putative, expressed
bradi2g03290	expressed protein
bradi4g14000	BRASSINOSTEROID INSENSITIVE 1-associated receptor kinase 1 precursor, putative,
bradi4g34022	no apical meristem protein, putative, expressed
bradi5g04686	Sec1 family transport protein, putative, expressed
bradi1g04830	AMP-binding enzyme, putative, expressed
bradi1g71920	expressed protein
bradi4g35317	resistance protein, putative, expressed
bradi3g06050	chorismate mutase, chloroplast precursor, putative, expressed
bradi3g33080	glycosyl transferase 8 domain containing protein, putative, expressed
bradi1g10950	BRASSINOSTEROID INSENSITIVE 1-associated receptor kinase 1 precursor, putative,
bradi3g44630	heparan-alpha-glucosaminide N-acetyltransferase, putative, expressed
bradi1g48630	proline-rich cell wall protein-like, putative, expressed
bradi2g12427	neutral/alkaline invertase, putative, expressed
bradi3g56070	phosphatidylinositol-4-phosphate 5-kinase, putative, expressed
bradi1g77160	expressed protein
bradi2g11240	OsSPL1 - SBP-box gene family member, expressed
bradi1g30520	sucrose-phosphate synthase, putative, expressed

bradi2g02510	Ser/Thr protein kinase, putative, expressed
bradi3g04217	phytosulfokine receptor precursor, putative, expressed
bradi1g75160	expressed protein
bradi3g17252	armadillo/beta-catenin repeat family protein, putative, expressed
bradi3g41900	expressed protein
bradi3g28730	SNF7 domain containing protein, putative, expressed
bradi1g71810	calmodulin-binding transcription activator, putative, expressed
bradi4g30200	expressed protein
bradi5g16810	DTA2, putative, expressed
bradi2g56900	DNA repair metallo-beta-lactamase, putative, expressed
bradi1g03827	expressed protein
bradi3g13177	phosphatidylinositol-4-phosphate 5-Kinase, putative, expressed
bradi2g11010	TsetseEP precursor, putative, expressed
bradi1g23580	lactose permease-related, putative, expressed
bradi3g27890	ras-related protein, putative, expressed
bradi1g34707	serine esterase, putative, expressed
bradi2g02430	OsWAK1 - OsWAK receptor-like cytoplasmic kinase OsWAK-RLCK, expressed
bradi1g21260	GRAM domain containing protein, expressed
bradi1g25540	TKL_IRAK_DUF26-lc.18 - DUF26 kinases have homology to DUF26 containing loci,
bradi5g10670	STE_MEKK_ste11_MAP3K.16 - STE kinases include homologs to sterile 7
bradi2g54340	ankyrin repeat-containing protein, putative, expressed

BRADI2G25580 co-expressed genes

bradi1g66085	No annotation
bradi2g08060	expressed protein
bradi3g07490	expressed protein
bradi2g25580	OsSPL9 - SBP-box gene family member, expressed
bradi2g55760	sorting nexin 1, putative, expressed
bradi2g25520	CUE domain containing protein, expressed
bradi1g64680	No annotation
bradi1g21130	expressed protein
bradi2g12010	expressed protein
bradi3g60210	protein kinase domain containing protein, expressed
bradi5g10810	coiled-coil domain-containing protein 55, putative, expressed
bradi3g17680	GTP-binding protein, putative, expressed
bradi5g21150	zinc finger domain, LSD1 subclass family protein, expressed
bradi3g43180	START domain containing protein, putative, expressed
bradi2g15390	PX domain containing protein, putative, expressed
bradi3g42850	vesicle-associated membrane protein 727, putative, expressed
bradi1g09200	CWF19-like 2, cell cycle control, putative, expressed
bradi3g58197	peptidyl-prolyl cis-trans isomerase CYP40, putative, expressed
bradi3g59870	pre-mRNA-splicing factor ATP-dependent RNA helicase DHX16, putative, expressed
bradi5g14660	cell division control protein 48 homolog B, putative, expressed
bradi2g52955	senescence-induced receptor-like serine/threonine-protein kinase precursor, putative,
bradi1g03800	ThiF family domain containing protein, putative, expressed
bradi1g01990	SWIM zinc finger family protein, putative, expressed
bradi4g27240	Ser/Thr protein phosphatase family protein, putative, expressed
bradi3g54980	translation initiation factor eIF-2B subunit epsilon, putative, expressed
bradi2g13180	NPL4, putative, expressed
bradi3g12830	RecF/RecN/SMC N terminal domain containing protein, expressed
bradi2g35807	serine/threonine-protein phosphatase 2A regulatory subunit B subunitgamma, putative,

bradi1g35110	AAA-type ATPase family protein, putative, expressed
bradi4g37380	nicotinate-nucleotide pyrophosphorylase, putative, expressed
bradi2g36430	signal peptide peptidase-like 2B, putative, expressed
bradi3g55660	tetratricopeptide repeat containing protein, putative, expressed
bradi1g62290	expressed protein
bradi4g09437	expressed protein
bradi1g30140	E2F-related protein, putative, expressed
bradi3g31900	RNA recognition motif, putative, expressed
bradi2g26300	calcium-binding mitochondrial protein anon-60Da, putative, expressed
bradi4g01760	SRP40, C-terminal domain containing protein, expressed
bradi4g08110	expressed protein
bradi1g18910	expressed protein
bradi4g14220	TKL_IRAK_DUF26-la.7 - DUF26 kinases have homology to DUF26 containing loci,
bradi2g07430	smr domain containing protein, expressed
bradi1g27400	dnaJ domain containing protein, expressed
bradi4g36330	XAP5 family protein, putative, expressed
bradi2g53050	expressed protein
bradi1g60040	DEK C terminal domain containing protein, expressed
bradi3g04630	CAMK_KIN1/SNF1 includes calcium/calmodulin depedent protein kinases, expressed
bradi5g23970	BRCA1-associated protein, putative, expressed
bradi5g10432	No annotation
bradi2g49840	hydrolase, acting on carbon-nitrogen, putative, expressed
bradi2g14740	protein phosphatase 2C, putative, expressed
bradi1g10210	fip1 motif family protein, expressed
bradi2g35740	expressed protein
bradi2g33230	zinc finger, C3HC4 type domain containing protein, expressed
bradi3g11480	expressed protein
bradi3g27750	amine oxidase, putative, expressed
bradi3g33900	methyltransferase, putative, expressed
bradi3g54910	ankyrin repeat domain containing protein, expressed
bradi3g53670	serine esterase family protein, putative, expressed
bradi1g17900	chromosome segregation protein sudA, putative, expressed
bradi5g25800	RCD1, putative, expressed
bradi1g72092	expressed protein
bradi2g42210	No annotation
bradi3g14190	pre-mRNA-splicing factor SLU7, putative, expressed
bradi1g15000	bromodomain associated family protein, expressed
bradi3g03680	KIN, antigenic determinant of recA protein, putative, expressed
bradi3g49650	e2f-associated phosphoprotein, putative, expressed
bradi3g23340	pyridoxamine 5\'-phosphate oxidase family protein, putative, expressed
bradi5g22130	OTU-like cysteine protease family protein, putative, expressed
bradi1g53640	SNF7 domain containing protein, putative, expressed
bradi4g22690	phosphatidylinositol kinase, putative, expressed
bradi3g57560	expressed protein
bradi1g29340	expressed protein
bradi4g14050	thioredoxin domain-containing protein 9, putative, expressed
bradi1g08350	expressed protein
bradi1g50497	expressed protein
bradi1g47367	expressed protein
bradi4g41390	splicing factor, putative, expressed
bradi3g24680	vacuolar-sorting receptor precursor, putative, expressed

bradi5g14550	RNA recognition motif containing protein, putative, expressed
bradi2g26120	FAR1 family protein
bradi2g08410	myosin heavy chain-related, putative, expressed
bradi2g56830	powdery mildew resistant protein 5, putative, expressed
bradi4g25177	zinc finger, C3HC4 type domain containing protein, expressed
bradi1g56970	CAMK_CAMK_like.31 - CAMK includes calcium/calmodulin depedent protein kinases,
bradi5g01990	WD domain, G-beta repeat domain containing protein, expressed
bradi5g08610	expressed protein
bradi4g32070	NFKB, putative, expressed
bradi1g72680	sodium/calcium exchanger protein, putative, expressed
bradi3g38200	bZIP transcription factor, putative, expressed
bradi4g24170	No annotation
bradi1g26960	FAR1 family protein, expressed
bradi1g38380	crooked neck, putative, expressed
bradi1g30648	transcription initiation factor TFIID subunit 1, putative, expressed
bradi3g40920	expressed protein
bradi2g23720	periplasmic beta-glucosidase precursor, putative, expressed
bradi2g21780	protein kri1, putative, expressed
bradi2g09246	expressed protein
bradi1g59920	FACT complex subunit SPT16, putative, expressed
bradi4g04800	expressed protein
bradi3g55710	SNARE domain containing protein, putative, expressed
bradi2g42160	coiled-coil domain-containing protein, putative, expressed
bradi3g31947	erythronate-4-phosphate dehydrogenase, putative, expressed
bradi3g47867	EMB2423, putative, expressed
bradi2g56690	RIO1 family protein, expressed
bradi3g59627	nuclear-pore anchor, putative, expressed
bradi1g10000	heat shock protein DnaJ, putative, expressed
bradi1g23930	zinc finger family protein, putative, expressed
bradi3g50560	amino acid permease, putative, expressed
bradi2g48130	AP2 domain containing protein, expressed
bradi1g27990	SNF7 domain containing protein, putative, expressed
bradi2g10200	BSD domain-containing protein, putative, expressed
bradi3g17020	ABC transporter, ATP-binding protein, putative, expressed
bradi1g67770	SWIM zinc finger family protein, putative, expressed
bradi1g71340	proteasome subunit, putative, expressed
bradi1g05950	CK1_CaseinKinase_1a.4 - CK1 includes the casein kinase 1 kinases, expressed
bradi1g47360	X0001, putative, expressed
bradi3g39560	microfibrillar-associated protein 1, putative, expressed
bradi3g46700	OsFBK9 - F-box domain and kelch repeat containing protein, expressed
bradi3g47860	expressed protein
bradi3g17040	expressed protein
bradi4g41960	expressed protein
bradi5g17270	RNA recognition motif containing protein, expressed
bradi1g19340	SNARE associated Golgi protein, putative, expressed
bradi3g11377	GTP binding protein, putative, expressed
bradi5g26340	zinc finger, RING-type, putative, expressed
bradi5g08235	2-oxoglutarate dehydrogenase E1 component, mitochondrial precursor, putative,
bradi3g29980	pre-mRNA-processing factor 6, putative, expressed
bradi4g19990	pre-mRNA-splicing factor ATP-dependent RNA helicase DHX16, putative, expressed
bradi1g01830	expressed protein

bradi3g30080	OsGrx_S17 - glutaredoxin subgroup II, expressed
bradi5g25117	expressed protein
bradi3g51240	tRNA pseudouridine synthase family protein, putative, expressed
bradi1g45737	chaperone protein dnaJ, putative, expressed
bradi4g35301	GRAM domain containing protein, expressed
bradi1g18770	PHD-finger family protein, expressed
bradi3g01750	zinc carboxypeptidase family protein, putative, expressed
bradi1g70690	casein kinase II subunit alpha-2, putative, expressed
bradi2g03617	expressed protein
bradi3g30070	expressed protein
bradi3g45710	tetratricopeptide repeat domain containing protein, expressed
bradi3g45470	FAR1 family protein, expressed
bradi1g11410	ubiquitin-conjugating enzyme, putative, expressed
bradi4g06137	protein binding protein, putative, expressed
bradi1g35230	calcium-dependent protein kinase CPK1 adapter protein 2, putative, expressed
bradi2g55660	expressed protein
bradi1g36700	protein of unknown function domain containing protein, expressed
bradi4g05840	plus-3 domain containing protein, expressed
bradi3g21630	ankyrin repeat domain-containing protein 28, putative, expressed
bradi1g13510	DUF593 domain containing protein, expressed
bradi3g46820	galactosyltransferase family protein, putative, expressed
bradi3g45070	RNA recognition motif containing protein, putative, expressed
bradi2g45437	No annotation

BRADI1G31390 co-expressed genes

bradi3g05880	WAX2, putative, expressed
bradi5g07230	glycosyl hydrolase, putative, expressed
bradi3g28560	3-ketoacyl-CoA synthase precursor, putative, expressed
bradi1g71465	membrane associated DUF588 domain containing protein, putative, expressed
bradi1g04670	expressed protein
bradi2g61247	expressed protein
bradi4g33237	OsSub58 - Putative Subtilisin homologue, expressed
bradi3g50420	transferase family protein, putative, expressed
bradi4g31257	chlorophyll A-B binding protein, putative, expressed
bradi2g40410	3-ketoacyl-CoA synthase, putative, expressed
bradi4g33770	OsSPL17 - SBP-box gene family member, expressed
bradi3g21000	POEI11 - Pollen Ole e I allergen and extensin family protein precursor, expressed
bradi5g15160	GDSL-like lipase/acylhydrolase, putative, expressed
bradi4g38460	male sterility protein, putative, expressed
bradi4g37090	lysM domain-containing GPI-anchored protein precursor, putative, expressed
bradi2g33340	uncharacterized protein At4g06744 precursor, putative, expressed
bradi1g60610	OsSCP14 - Putative Serine Carboxypeptidase homologue, expressed
bradi1g19070	eukaryotic aspartyl protease domain containing protein, expressed
bradi2g19650	GDSL-like lipase/acylhydrolase, putative, expressed
bradi4g07580	POEI19 - Pollen Ole e I allergen and extensin family protein precursor, expressed
bradi2g53420	inactive receptor kinase At2g26730 precursor, putative, expressed
bradi1g54030	NAD dependent epimerase/dehydratase family protein, putative, expressed
bradi2g53610	DUF623 domain containing protein, expressed
bradi2g25620	receptor-like protein kinase precursor, putative, expressed
bradi3g57567	homeobox and START domains containing protein, putative, expressed
bradi2g49500	pectinesterase, putative, expressed

bradi1g33817	glycosyl hydrolases family 16, putative, expressed
bradi2g54980	ATROPGEF7/ROPGEF7, putative, expressed
bradi2g03610	light-induced protein 1-like, putative, expressed
bradi5g14540	expressed protein
bradi5g04640	glycosyl hydrolase family 10 protein, putative, expressed
bradi5g17860	OsSCP56 - Putative Serine Carboxypeptidase homologue, expressed
bradi3g34410	homeobox and START domains containing protein, putative, expressed
bradi5g22907	glycosyl hydrolases family 16 protein, protein, expressed
bradi4g35970	endoglucanase, putative, expressed
bradi1g44030	3-ketoacyl-CoA synthase, putative, expressed
bradi5g16230	OsSCP25 - Putative Serine Carboxypeptidase homologue, expressed
bradi2g09607	transmembrane protein 136, putative, expressed
bradi3g15040	polyphenol oxidase, putative, expressed
bradi4g35510	3-ketoacyl-CoA synthase precursor, putative, expressed
bradi5g26620	Eukaryotic aspartyl protease domain containing protein, expressed
bradi3g38020	oxidoreductase, short chain dehydrogenase/reductase family domain containing protein,
bradi4g22660	peroxidase precursor, putative, expressed
bradi1g12350	GDSL-like lipase/acylhydrolase, putative, expressed
bradi1g68400	POEI16 - Pollen Ole e I allergen and extensin family protein precursor, expressed
bradi1g06460	TCP family transcription factor, putative, expressed
bradi2g25070	GDSL-like lipase/acylhydrolase, putative, expressed
bradi2g53120	LTPL65 - Protease inhibitor/seed storage/LTP family protein precursor, expressed
bradi2g06790	bZIP transcription factor domain containing protein, expressed
bradi5g09120	cytochrome P450, putative, expressed
bradi3g38740	expressed protein
bradi3g12497	transferase family protein, putative, expressed
bradi3g51370	cytochrome P450, putative, expressed
bradi4g30587	aspartic proteinase, putative, expressed
bradi2g06890	TCP family transcription factor, putative, expressed
bradi5g23770	LTPL128 - Protease inhibitor/seed storage/LTP family protein precursor, expressed
bradi3g38417	cyclin, putative, expressed
bradi1g01920	GDSL-like lipase/acylhydrolase, putative, expressed
bradi4g44750	protein phosphatase 2c, putative, expressed
bradi1g08880	transferase family protein, putative, expressed
bradi2g57620	ribonuclease T2 family domain containing protein, expressed
bradi4g04190	expressed protein
bradi1g32810	cytochrome P450, putative, expressed
bradi1g49400	expressed protein
bradi2g00920	TOO MANY MOUTHS precursor, putative, expressed
bradi1g25157	CSLF3 - cellulose synthase-like family F; beta1,3;1,4 glucan synthase, expressed
bradi2g52050	early nodulin 20 precursor, putative, expressed
bradi2g46120	fasciclin domain containing protein, expressed
bradi2g60870	expressed protein
bradi2g16800	ATROPGEF7/ROPGEF7, putative, expressed
bradi1g31390	OsSPL10 - SBP-box gene family member, expressed
bradi2g03807	GDSL-like lipase/acylhydrolase, putative, expressed
bradi3g37680	Cupin domain containing protein, expressed
bradi3g56480	transporter-related, putative, expressed
bradi1g23040	expressed protein
bradi2g45310	chloroplast unusual positioning protein, putative, expressed
bradi5g24670	OsSPL8 - SBP-box gene family member, expressed

bradi3g06550	GDSL-like lipase/acylhydrolase, putative, expressed
bradi1g20797	oxidoreductase, short chain dehydrogenase/reductase family, putative, expressed
bradi2g26063	serine/threonine-protein kinase, putative, expressed
bradi3g01110	cytochrome P450, putative, expressed
bradi1g68590	glycosyl hydrolases family 16, putative, expressed
bradi3g08180	beta-galactosidase precursor, putative, expressed
bradi5g27650	ABC-2 type transporter domain containing protein, expressed
bradi4g34770	POEI18 - Pollen Ole e I allergen and extensin family protein precursor, expressed
bradi2g11860	pectinesterase, putative, expressed
bradi3g53630	SCP-like extracellular protein, expressed
bradi5g17150	expressed protein
bradi2g24220	OsSub47 - Putative Subtilisin homologue, expressed
bradi1g50760	aspartic protease, putative, expressed
bradi2g06980	GDSL-like lipase/acylhydrolase, putative, expressed
bradi1g72110	aspartic proteinase nepenthesin precursor, putative, expressed
bradi3g34950	BURP domain containing protein, expressed
bradi4g35660	MATE domain containing protein, expressed
bradi2g11950	lipase, putative, expressed
bradi3g48800	uncharacterized protein At4g06744 precursor, putative, expressed
bradi5g00750	transferase family protein, putative, expressed
bradi2g21830	LTPL67 - Protease inhibitor/seed storage/LTP family protein precursor, expressed
bradi4g18920	CBS domain-containing protein, putative, expressed
bradi2g45320	proteins of unknown function domain containing protein, expressed
bradi1g65110	receptor-like kinase RHG1, putative, expressed
bradi1g77610	AT-GTL1, putative, expressed
bradi1g40464	ABC-2 type transporter domain containing protein, expressed
bradi5g17110	aldehyde dehydrogenase, putative, expressed
bradi4g15460	glucan endo-1,3-beta-glucosidase precursor, putative, expressed
bradi5g09320	2Fe-2S iron-sulfur cluster binding domain containing protein, expressed
bradi5g18910	OsSub45 - Putative Subtilisin homologue, expressed
bradi3g52220	multidrug resistance protein, putative, expressed
bradi1g11790	possible lysine decarboxylase domain containing protein, expressed
bradi1g76630	fasciclin-like arabinogalactan protein, putative, expressed
bradi1g73910	LTPL82 - Protease inhibitor/seed storage/LTP family protein precursor, expressed
bradi2g11410	beta-D-xylosidase, putative, expressed
bradi3g08930	aquaporin protein, putative, expressed
bradi5g17850	pectinesterase, putative, expressed
bradi3g37670	Cupin domain containing protein, expressed
bradi3g52060	invertase/pectin methylesterase inhibitor family protein, putative, expressed
bradi3g58250	peptidyl-prolyl cis-trans isomerase, FKBP-type, putative, expressed
bradi3g12750	carboxyl-terminal peptidase, putative, expressed
bradi4g19480	RALFL14 - Rapid ALkalinization Factor RALF family protein precursor, expressed
bradi3g55100	WAX2, putative, expressed
bradi3g48770	uncharacterized protein At4g06744 precursor, putative, expressed
bradi1g18150	DUF567 domain containing protein, putative, expressed
bradi3g19360	aspartic proteinase nepenthesin precursor, putative, expressed
bradi3g26827	3-ketoacyl-CoA synthase, putative, expressed

BRADI3G05510 co-expressed genes

bradi2g09980	uncharacterized Cys-rich domain containing protein, putative, expressed
bradi3g52000	OsFBX59 - F-box domain containing protein, expressed

bradi1g57450	WD domain, G-beta repeat domain containing protein, expressed
bradi4g43990	expressed protein
bradi3g48520	enhancer of rudimentary protein, putative, expressed
bradi4g36170	WD repeat-containing protein, putative, expressed
bradi1g45790	plastocyanin-like domain containing protein, putative, expressed
bradi1g24610	secretory carrier-associated membrane protein, putative, expressed
bradi2g48020	green ripe-like, putative, expressed
bradi3g59237	shikimate kinase, putative, expressed
bradi2g23200	expressed protein
bradi2g34270	mitochondrial carrier protein, putative, expressed
bradi1g58920	expressed protein
bradi1g53960	armadillo/beta-catenin-like repeat containing protein, expressed
bradi1g49700	expressed protein
bradi3g05510	OsSPL11 - SBP-box gene family member, expressed
bradi4g37720	OsRCI2-11 - Hydrophobic protein OSR8, expressed
bradi3g59237	shikimate kinase, putative, expressed
bradi1g53800	NADH-ubiquinone oxidoreductase 51 kDa subunit, mitochondrial precursor, putative,
bradi2g13810	heat shock protein DnaJ, putative, expressed
bradi2g44350	CGMC_MAPKCMGC_2.4 - CGMC includes CDA, MAPK, GSK3, and CLKC kinases,
bradi1g75340	ras-related protein, putative, expressed
bradi1g12480	ubiquitin-conjugating enzyme E2 W, putative, expressed
bradi1g55170	targeting protein for Xklp2, putative, expressed
bradi3g56720	expressed protein
bradi3g54870	ABC transporter, ATP-binding protein, putative, expressed
bradi5g21750	peptidase, trypsin-like serine and cysteine proteases, putative, expressed
bradi2g22750	CAMK_CAMK_like.29 - CAMK includes calcium/calmodulin depedent protein kinases,
bradi1g13870	nucleolar protein NOP5, putative, expressed
bradi3g54290	protein phosphatase 2C, putative, expressed
bradi3g03407	auxin response factor 5, putative, expressed
bradi3g13640	expressed protein
bradi1g37020	enoyl-CoA hydratase/isomerase family protein, putative, expressed
bradi1g70300	heparanase-like protein precursor, putative, expressed
bradi2g54770	DHHC zinc finger domain containing protein, expressed
bradi5g07310	CS domain containing protein, putative, expressed
bradi5g13727	steroid nuclear receptor, ligand-binding, putative, expressed
bradi3g55510	DHHC zinc finger domain containing protein, expressed
bradi2g46900	DUF292 domain containing protein, expressed
bradi2g45460	AMP-binding enzyme, putative, expressed
bradi1g66910	oxidoreductase, short chain dehydrogenase/reductase family domain containing protein,
bradi5g12340	RAN guanine nucleotide release factor, putative, expressed
bradi1g33850	DNA-directed RNA polymerases I, II, and III subunit RPABC3, putative, expressed
bradi2g59360	zinc finger, C3HC4 type domain containing protein, expressed
bradi3g29790	Ser/Thr protein phosphatase family protein, putative, expressed
bradi1g03750	actin-depolymerizing factor, putative, expressed
bradi2g13110	glutathione S-transferase, putative, expressed
bradi3g46180	SFT2, putative, expressed
bradi3g30820	fringe-related protein, putative, expressed
bradi2g17687	Divergent PAP2 family domain containing protein, expressed
bradi2g09434	RGH2B, putative, expressed
bradi2g30500	ras-related protein, putative, expressed

BRADI4G34667 co-expressed genes

bradi1g42000	cycloartenol synthase, putative, expressed
bradi4g04590	DUF538 domain containing protein, putative, expressed
bradi1g37390	Cyclopropane-fatty-acyl-phospholipid synthase, putative, expressed
bradi1g66910	oxidoreductase, short chain dehydrogenase/reductase family domain containing protein,
bradi2g44377	glycerol-3-phosphate acyltransferase, putative, expressed
bradi2g13110	glutathione S-transferase, putative, expressed
bradi4g34667	OsSPL18 - SBP-box gene family member, expressed

BRADI2G59110 co-expressed genes

bradi5g16010	lysine-rich arabinogalactan protein 19 precursor, putative, expressed
bradi3g58630	phosphate-induced protein 1 conserved region domain containing protein, expressed
bradi3g12560	zinc finger, C3HC4 type domain containing protein, expressed
bradi1g64790	calmodulin binding protein, putative, expressed
bradi3g58620	phosphate-induced protein 1 conserved region domain containing protein, expressed
bradi2g56890	nodulin MtN3 family protein, putative, expressed
bradi2g44390	X8 domain containing protein, expressed
bradi5g14650	peroxidase precursor, putative, expressed
bradi2g59110	OsSPL2 - SBP-box gene family member, expressed
bradi3g08790	ethylene-responsive transcription factor TINY, putative, expressed
bradi2g02590	reticulon domain containing protein, putative, expressed
bradi1g03760	actin-depolymerizing factor, putative, expressed
bradi2g15532	pex14, putative, expressed
bradi5g04710	expressed protein
bradi3g05380	zinc-binding protein, putative, expressed
bradi3g51707	DUF623 domain containing protein, expressed
bradi2g01480	glycosyltransferase protein, putative, expressed
bradi2g07357	AP2 domain containing protein, expressed
bradi1g33840	glycosyl hydrolases family 16, putative, expressed
bradi4g01190	hsp20/alpha crystallin family protein, putative, expressed

BRADI1G26720 co-expressed genes

bradi2g42477	uncharacterized protein At4g06744 precursor, putative, expressed
bradi2g07357	AP2 domain containing protein, expressed
bradi2g17030	aspartyl protease family protein, putative, expressed
bradi1g26720	OsSPL13 - SBP-box gene family member, expressed
bradi2g31080	expressed protein
bradi1g13240	sodium/calcium exchanger protein, putative, expressed
bradi4g29340	RING-H2 finger protein, putative, expressed
bradi1g53567	expressed protein
bradi4g31740	expressed protein
bradi1g14090	IQ calmodulin-binding motif family protein, putative, expressed
bradi1g38330	expressed protein

Table S6: List of BdSBPs potential interactor proteins.

BRADI2G11240 interactor proteins

#node	domain_summary_url
BRADI2G09990.1	http://smart.embl.de/smart/DD2.cgi?smart=440:COIL(166|201)+Pfam_Sad1_UNC(293|432)+
BRADI2G11240.1	http://smart.embl.de/smart/DD2.cgi?smart=766:Pfam_SBP(131|209)+
BRADI2G44520.1	http://smart.embl.de/smart/DD2.cgi?smart=96:SANT(9|61)+
BRADI3G03680.1	http://smart.embl.de/smart/DD2.cgi?smart=416:ZnF_C2H2(26|50)+Kin17_mid(52|178)+

BRADI2G25580.1 interactor proteins

#node	domain_summary_url
BRADI2G25580.1	http://smart.embl.de/smart/DD2.cgi?smart=849:Pfam_SBP(164|242)+
BRADI2G51210.1	http://smart.embl.de/smart/DD2.cgi?smart=162:Pfam_Ctr(36|84)+Pfam_Ctr(82|136)+
BRADI3G03680.1	http://smart.embl.de/smart/DD2.cgi?smart=416:ZnF_C2H2(26|50)+Kin17_mid(52|178)+

BRADI3G03510.1 interactor proteins

#node	domain_summary_url
BRADI3G03510.1	http://smart.embl.de/smart/DD2.cgi?smart=485:Pfam_SBP(187|265)+
BRADI4G27480.1	http://smart.embl.de/smart/DD2.cgi?smart=318:Pfam_Chloroa_b-bind(114|291)+

BRADI3G40030 interactor proteins

#node	domain_summary_url
BRADI2G33037.2	http://smart.embl.de/smart/DD2.cgi?smart=604:Pfam_Pkinase(39|324)+Pfam_Pkinase_Tyr(39|313)+
BRADI2G47850.1	http://smart.embl.de/smart/DD2.cgi?smart=471:Pfam_Pkinase(9|276)+Pfam_Pkinase_Tyr(9|273)+
BRADI2G49590.1	http://smart.embl.de/smart/DD2.cgi?smart=660:Pfam_Pkinase(100|371)+Pfam_Pkinase_Tyr(100|370)+
BRADI2G51370.1	http://smart.embl.de/smart/DD2.cgi?smart=698:Pfam_Pkinase(138|409)+Pfam_Pkinase_Tyr(138|378)+
BRADI3G10320.2	http://smart.embl.de/smart/DD2.cgi?smart=416:Pfam_Pkinase_Tyr(9|273)+Pfam_Pkinase(9|276)+
BRADI3G28720.1	http://smart.embl.de/smart/DD2.cgi?smart=476:Pfam_Pkinase_Tyr(9|273)+Pfam_Pkinase(9|276)+
BRADI3G40030.1	http://smart.embl.de/smart/DD2.cgi?smart=391:Pfam_SBP(80|158)+
BRADI3G49100.1	http://smart.embl.de/smart/DD2.cgi?smart=466:Pfam_Pkinase(9|283)+Pfam_Pkinase_Tyr(9|273)+
BRADI3G54860.1	http://smart.embl.de/smart/DD2.cgi?smart=468:Pfam_Pkinase(9|277)+Pfam_Pkinase_Tyr(9|273)+
BRADI4G00280.1	http://smart.embl.de/smart/DD2.cgi?smart=644:Pfam_Pkinase(83|371)+Pfam_Pkinase_Tyr(83|358)+
BRADI4G06500.1	http://smart.embl.de/smart/DDt.cgi?smart=386:

BRADI3G40240 interactor proteins

#node	domain_summary_url
BRADI1G46427.1	http://smart.embl.de/smart/DD2.cgi?smart=321:QLQ(16|52)+Pfam_WRC(91|136)+
BRADI3G40240.1	http://smart.embl.de/smart/DD2.cgi?smart=1126:Pfam_SBP(187|265)+TRANS(1086|1108)+

BRADI4G33770.1 interactor proteins

#node	domain_summary_url
BRADI1G05550.1	http://smart.embl.de/smart/DD2.cgi?smart=347:HSF(32|125)+COIL(148|189)+
BRADI1G13910.1	http://smart.embl.de/smart/DD2.cgi?smart=861:HOX(32|98)+COIL(99|132)+START(183|393)
BRADI1G77087.1	http://smart.embl.de/smart/DD2.cgi?smart=1900:DEXDc(266|431)+HELICc(665|751)+Pfam_Dicer_
FTL1	http://smart.embl.de/smart/DD2.cgi?smart=173:Pfam_PBP(23|161)+

BRADI2G53010.1	http://smart.embl.de/smart/DD2.cgi?smart=556:SANT(41|91)+SANT(94|142)+
BRADI3G28720.1	http://smart.embl.de/smart/DD2.cgi?smart=476:Pfam_Pkinase_Tyr(9|273)+Pfam_Pkinase(9|276)+
BRADI3G49100.1	http://smart.embl.de/smart/DD2.cgi?smart=466:Pfam_Pkinase(9|283)+Pfam_Pkinase_Tyr(9|273)+
BRADI3G54860.1	http://smart.embl.de/smart/DD2.cgi?smart=468:Pfam_Pkinase(9|277)+Pfam_Pkinase_Tyr(9|273)+
BRADI4G00280.1	http://smart.embl.de/smart/DD2.cgi?smart=644:Pfam_Pkinase(83|371)+Pfam_Pkinase_Tyr(83|358)+
BRADI4G10171.1	http://smart.embl.de/smart/DD2.cgi?smart=847:Pfam_NB-
BRADI4G33770.1	http://smart.embl.de/smart/DD2.cgi?smart=407:Pfam_SBP(77|155)+

BRADI5G17720 interactor proteins

#node	domain_summary_url
BRADI1G15440.1	http://smart.embl.de/smart/DD2.cgi?smart=1383:DEXDc(23|223)+Pfam_Dicer_dimer(552|628)
BRADI1G21030.1	http://smart.embl.de/smart/DD2.cgi?smart=1219:DEXDc(27|232)+PAZ(642|784)+RIBOc(804|
BRADI2G23187.2	http://smart.embl.de/smart/DD2.cgi?smart=876:PAZ(106|271)+RIBOc(288|464)+RIBOc(497|653
BRADI2G58270.1	http://smart.embl.de/smart/DD2.cgi?smart=1109:PAZ(318|498)+RIBOc(515|691)+RIBOc(7
BRADI5G17720.1	http://smart.embl.de/smart/DD2.cgi?smart=188:Pfam_SBP(71|134)+Pfam_SBP(123|172)+

BRADI5G24670.1 interactor proteins

#node	domain_summary_url
CRNKL1	http://smart.embl.de/smart/DD2.cgi?smart=717:HAT(85|117)+HAT(119|151)+HAT(153|18
BRADI2G11540.1	http://smart.embl.de/smart/DD2.cgi?smart=290:KNOX1(36|80)+KNOX2(81|132)+ELK(17
BRADI2G38390.1	http://smart.embl.de/smart/DD2.cgi?smart=300:KNOX1(40|84)+KNOX2(90|141)+ELK(18
BRADI2G55567.1	http://smart.embl.de/smart/DD2.cgi?smart=544:BRLZ(230|306)+Pfam_DOG1(313|391)+
BRADI3G35130.1	http://smart.embl.de/smart/DD2.cgi?smart=304:TRANS(13|35)+
BRADI5G24670.1	http://smart.embl.de/smart/DD2.cgi?smart=420:Pfam_SBP(185|264)+

BRADI4G18900.1 interactor proteins

#node	domain_summary_url
BRADI1G08550.2	http://smart.embl.de/smart/DD2.cgi?smart=630:SIGNAL(1|22)+Pfam_Glyco_hydro_3(48|376)+
BRADI2G32530.1	http://smart.embl.de/smart/DD2.cgi?smart=497:Pfam_Pyr_redox_2(34|399)+
BRADI3G49747.1	http://smart.embl.de/smart/DD2.cgi?smart=725:Pfam_DUF639(34|720)+
BRADI4G18900.1	http://smart.embl.de/smart/DD2.cgi?smart=216:Pfam_SBP(3|37)+

Supplementary Table S7: Primer sequences used in the study

Primer name	Primer Sequences
Bd41250qF	ACCATTTCCAAATCCAAGACC
Bd41250qR	AATGCTGCCTGTTGCTGATAC
Bd33770qR	TCCAGCAGAAAGCTTCTGAAC
Bd34667qF	ACACCCATCAGGTCCTCAAC
Bd34667qR	GACGACGGACTTCAGGAGAG
Bd59110qF	GACGAGGTGAAGAGGAGCTG
Bd59110qR	AAGATTTGCGGGTACGACAC
BdSPL7FT	GGCACCTAGCAACTGGTACTCG
BdSPL7RT	ATCGAGCATGACAGCCGTAG
Bd3510o	GGAGCGTCTATCATTATCAGTG
Bd3510i	TATCAGTGCATCCGGTCGAAG
Bd667o	AGATCCCGTGGCAGTTCATC
Bd667i	CATCTCGTGGTCGTTGGAG
Bd59110o	TGGTGCTGGTAGTAGCCATG
Bd59110i	GCCATGGTATTGCTGTAGCTG
Bd33770o	GATGGACATCGCTCATCACAG
Bd33770i	ACAGCGTCATGATGATGGTG
BdSPL2qR	CTTCTTGACCGAAGCAGGAG
BdSPL2qF	TCGGTGATAGCAGTGAGCAG
BdSamDC F	TGCTAATCTGCTCCAATGGC
BdSamDC R	GACGCAGCTGACCACCTAGA
BdUBC18 F	GGAGGCACCTCAGGTCATTT
BdUBC18 R	ATAGCGGTCATTGTCTTGCG
T3 T-DNA LB	AGCTGTTTCCTGTGTGAAATTG
R9 T-DNA LB	GATAAGCTGTCAAACATGAGAATTCAG
BdSPL7qF	TCTCACGAGACCATTGCTTG
BdSPL7qR	AGCCCAATCATGTTTTCCAC
BdSPL8qR	CCGTCGATGATGACTTGTTG
BdSPL14qF	GATTTCTGGAGAAACGGAAGG
BdSPL14qR	TCTTTCTCCCACCAACAACAG
BdSPL8qF	GAGTTTGACGAGGCCAAGAG
Hyg Rev	CTATTTCTTTGCCCTCGGACGAGTGC
Hyg Fwd	ATGAAAAAGCCTGAACTCACCGCGAC
